# Mass Spectrometric ITEM-FOUR Analysis Reveals Coding Single-Nucleotide Polymorphisms in Human Cardiac Troponin T That Evade Detection by Sandwich ELISAs Which Use Monoclonal Antibodies M7 and M11.7 from the Elecsys Troponin T^®^ Assay

**DOI:** 10.3390/ijms26104892

**Published:** 2025-05-20

**Authors:** Kristjan Kormann, Manuela Ruß, Claudia Röwer, Cornelia Koy, Michael O. Glocker

**Affiliations:** Proteome Center Rostock University Medicine and Natural Science Faculty, University of Rostock, Schillingallee 69, 18057 Rostock, Germanycornelia.koy@med.uni-rostock.de (C.K.)

**Keywords:** ITEM-FOUR, nano-ESI mass spectrometry, immune complex analysis, myocardial infarction, human troponin T, single-nucleotide polymorphism, single-amino-acid polymorphism

## Abstract

Immunoassays for cardiac troponin, such as the Elecsys^®^ hs-TnT, have become the gold standard for myocardial infarction diagnostics. While various protein/chemical factors affecting the troponin complex and thus its diagnostic accuracy have been investigated, the role of coding single-nucleotide polymorphisms remains underexplored. To evaluate potential cSNP-induced interference with antibody binding in the Elecsys^®^ hs-TnT immunoassay, we applied ITEM-FOUR, a mass spectrometry-based method that quantifies changes in antibody binding upon amino acid substitutions in epitope peptides. Candidate cSNPs were selected from the dbSNP database and were mapped to human cardiac troponin T by molecular modeling. Consuming micromolar antibody concentrations and microliter sample volumes, two wild-type and 17 cSNP-derived variant epitope peptides—six for monoclonal antibody M7 and eleven for monoclonal antibody M11.7—were investigated to reveal the binding motifs “V_131_-K_134_-E_138_-A_142_” for M7 and “E_146_-I_150_-R_154_-E_157_” for M11.7. Loss of binding to M11.7 was observed for substitutions Q148R (rs730880232), R154W (rs483352832), and R154Q (rs745632066), whereas the E138K (rs730881100) exchange disrupted binding of M7. Except for cSNP Q148R, they are associated with cardiomyopathies, placing affected individuals at risk of both underlying heart disease and false-negative hs-TnT assay results in cases of myocardial infarction. Our results highlight the need to account for cSNP-related interferences in antibody-based diagnostics. ITEM-FOUR offers a powerful approach for tackling this challenge, fostering next-generation assay development.

## 1. Introduction

Modern healthcare systems are critically and crucially dependent upon reliable diagnostics [1]. By the year 2000, the European Society of Cardiology (ESC) and the American College of Cardiology (ACC) had established troponin as the biomarker of choice for detection of myocardial infarction (MI) [2]. Cardiac troponin T (cTnT) and cardiac troponin I (cTnI) are highly expressed in cardiomyocytes and are released into the circulation upon acute MI and other causes of cardiomyocyte death [3]. With the high-sensitivity (hs) troponin assay, it is possible to detect even the smallest amounts of troponin within the currently recommend cut-off values [4]. Many properties of the troponin complex constituents have been scrutinized as to whether or not they might potentially be limiting diagnostic accuracy, like epitope masking through protein–protein interactions [5], or the roles of interfering minor compounds, such as biotin present in blood [6]. A blind spot in that regard has to date been the role of coding single-nucleotide polymorphisms (cSNPs) as mutual factors that potentially undermine accurate analysis results of commercialized diagnostic tests [7].

Single-nucleotide polymorphisms (SNPs) are the most common type of genetic variation in the human genome [8]. Their determination can be used as a predictive value and is leveraged to assess disease susceptibility, functional consequences in disease processes, and individual responses to drug therapy [9]. SNPs have been shown to define the risk of an individual’s susceptibility to various illnesses and response to drugs [10]. Mutations in the troponin T gene (*TNNT2*) in particular represent an important subset of known disease-causing mutations [11]. Recent meta-studies have shown that some representatives of the heterogeneous group of cardiomyopathies, namely hypertrophic (HCM), dilative (DCM), and restrictive (RCM), can be caused by cSNPs in the troponin T gene [12,13].

It is self-evident that diagnostic assay performance must be continuously reevaluated, and commercial assays should be updated and refined based on the latest research results to ensure more accurate test results. Following this line of thought, we investigated whether cardiomyopathy-related cSNPs of the human cTnT antigen (hcTnT) were interfering with the binding of the antigen to the anti-hcTnT antibodies M7 and M11.7, which are applied in the commercial Elecsys^®^ hs-TnT immunoassay [14] and whose epitopes are known [15,16,17].

To experimentally verify or falsify postulated cSNP-caused binding interference with the M7 and M11.7 antibodies, we used the recently developed mass spectrometric ITEM-FOUR methodology [18], which has been found capable of determining even subtle differences in binding strengths of epitope peptides to antibodies upon replacing original amino acid residues with unusual amino acid residues. ITEM-FOUR makes use of four key features, which all are provided by modern mass spectrometers: (i) soft ionization, which allows non-covalent protein–peptide complexes to survive intact during ionization and transition from the condensed phase to the gas phase [19,20,21]; (ii) the mass analyzer’s unsurpassed effectiveness of sorting ions with differentiating properties, such as their *m*/*z* values [22,23] and beyond [24,25]; (iii) the mass spectrometer’s ability to perform unimolecular gas phase reactions in a collision cell [26], such as complex dissociation [27]; and (iiii) the high sensitivity of ion detection, which in principle allows even single-ion recording [28,29].

In this study, we investigated the binding strengths of the two wild-type epitope peptides of the two monoclonal antibodies M7 and M11.7 and of 17 aberrant cSNP-derived epitope peptides (6 for M7 and 11 for M11.7). ITEM-FOUR analysis was able to differentiate orthodox binding peptides from unorthodox binding peptides [30], as well as from non-binding peptides. Additionally, bio-computational structure modeling [31] of hcTnT provides evidence that the binding motives are facing outward and orthodox and unorthodox binding peptides should on the protein level provide positive diagnostic assay results, whereas non-binding peptides are indicative of loss of binding also at the protein level, ultimately leading to negative diagnostic assay results.

## 2. Results

### 2.1. Characterization of Antibodies and Epitope Peptides

Since ITEM-FOUR analyses aim at determining the particular roles of amino acid residues at defined positions of a given epitope peptide sequence with respect to the binding strength of an epitope peptide to an antibody under investigation, the starting materials, epitope peptides and antibodies, need to be thoroughly characterized. The mass spectra of the monoclonal antibodies M7, M11.7, and anti-TNFα (negative control) indicated structural homogeneity of the antibodies and that they did not contain side products in measurable amounts. This was judged from the fairly slim multiply charged ion signals and narrow charge state distributions clustered around the 25+ ion signals (Figures S1–S3). The experimentally determined molecular masses of all three antibodies matched the expected values of above 148 kDa (Table S1A–C). 

The homogeneity and high purity of the hcTnT antigen were confirmed through SDS-PAGE analysis, which displayed a single protein band at an apparent molecular mass of approximately 40 kDa (Figure S4). Western blot analyses confirmed that both anti-hcTnT antibodies, M7 and M11.7, successfully recognized recombinant hcTnT as their target antigen, as was expected (Figure S5). 

Since the epitopes of both the M7 and M11.7 antibodies have been previously published, the corresponding 15-mer epitope peptides of the wild-type sequences were chemically synthesized, along with peptides that mimic the in vivo epitope peptide sequences that carry cSNPs (Table 1). Amino acid substitutions were taken from the dbSNP database (https://www.ncbi.nlm.nih.gov/snp/ (accessed on 30 November 2020)). The experimentally determined molecular masses of all synthetic epitope peptides (P11–P17 and P21–P32) matched the calculated values within the limits of experimental accuracy (Table 1). The mass spectra of the epitope peptides showed dominant ion signals corresponding to doubly, triply, and occasionally quadruply protonated peptide ions, with minimal or no significant background ion signals (Figures S6–S24).

Based on the mass spectrometric data and the immuno-analytical characterization results, all starting materials were approved for testing in-solution complex formation as well as for experimental investigation of complex dissociation behavior in the gas phase.

### 2.2. Binding Strength Analysis by ITEM-FOUR Mass Spectrometry

**Figure 1:**
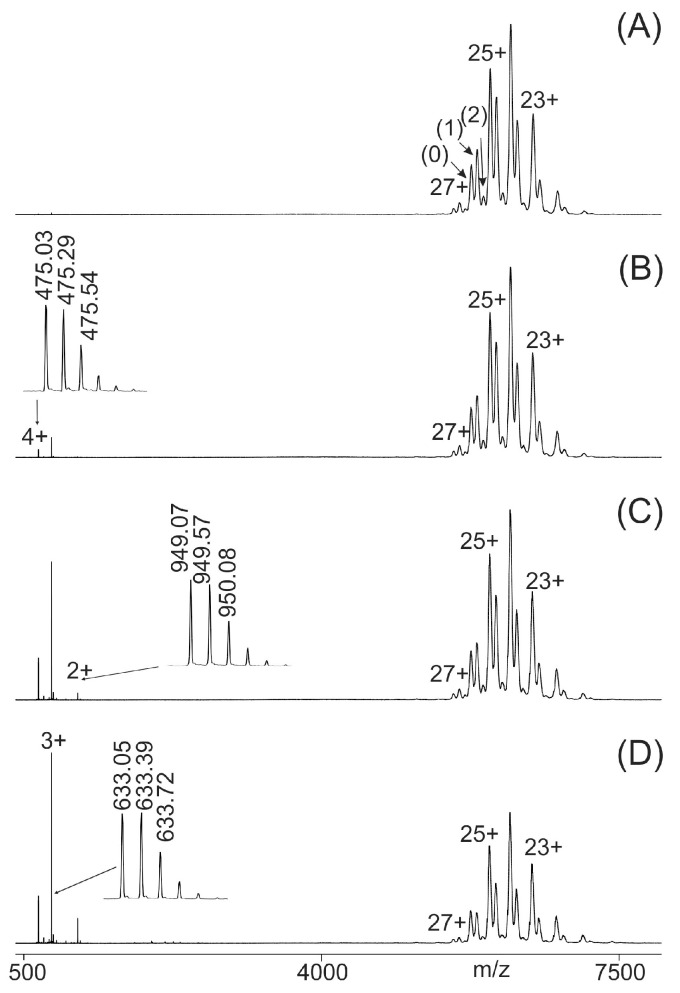
Nano-ESI mass spectra of peptide 11 (LVSLKDRIERRRAER)–M7 antibody mixtures with increasing collision cell voltage differences (ΔCV). (**A**): 2 V, (**B**): 14 V, (**C**): 30 V, (**D**): 52 V. Charge states are given for the ion signals (right ion series) of the antibody (0) and the immune complexes (antibody plus one peptide (1) and antibody plus two peptides (2)). Charge states for peptide ion signals are given on the left. The inlets in (**B**–**D**) show zoom views of the isotopically resolved peptide ion signals and their *m*/*z* values. The quadrupole was set to block transmission of ions < *m*/*z* 3850. The molar ratio of peptide to antibody was 2.1:1. Solvent: 200 mM ammonium acetate, pH 6.7.

Immune complex-containing solutions of a wild-type epitope peptide and its corresponding antibody, e.g., P11 and M7, were prepared by mixing the two components in 200 mM ammonium acetate buffer (pH 6.7), ensuring a molar excess of the epitope peptide over the antibody to achieve binding saturation. After an incubation period of one hour, the entire mixture (~3 µL) was loaded into a nano-ESI emitter and electrosprayed. The mass spectrometer was configured to transmit only ion signals with *m*/*z* values above 3850, effectively eliminating signals from the unbound peptide (Figure 1A).

Since the difference in collision cell voltage (ΔCV) was kept low (2 V), the ion signals in the high *m*/*z* region corresponded to the antibody (0), the intact immune complex with one epitope peptide bound to the antibody (1), and the intact immune complex with two epitope peptides bound to the antibody (2). While the instrument’s resolving power allowed for clear distinction of the (supra)molecular species for each charge state, enabling precise determination of their individual intensities, the added mass increments from the bound epitope peptide(s) were measured with insufficient accuracy. Yet, by increasing ΔCV, the immune complexes began to dissociate, releasing doubly, triply, and quadruply protonated epitope peptides. Their ion signals were recorded with isotopic resolution, enabling precise mass determination and unambiguous definition of their molecular identity. This process is demonstrated for the peptide P11, which underwent collision-induced dissociation from the M7–P11 immune complex (Figure 1B). Further increases in ΔCV produced larger amounts of free epitope peptide ions and free antibody ions (products) while decreasing the ion signal intensities of the complex ions (starting materials, educts). The complex-released epitope peptide ion signals and the ion signals of the free antibody dominated the mass spectra at higher ΔCV settings (Figure 1C,D).

**Figure 2:**
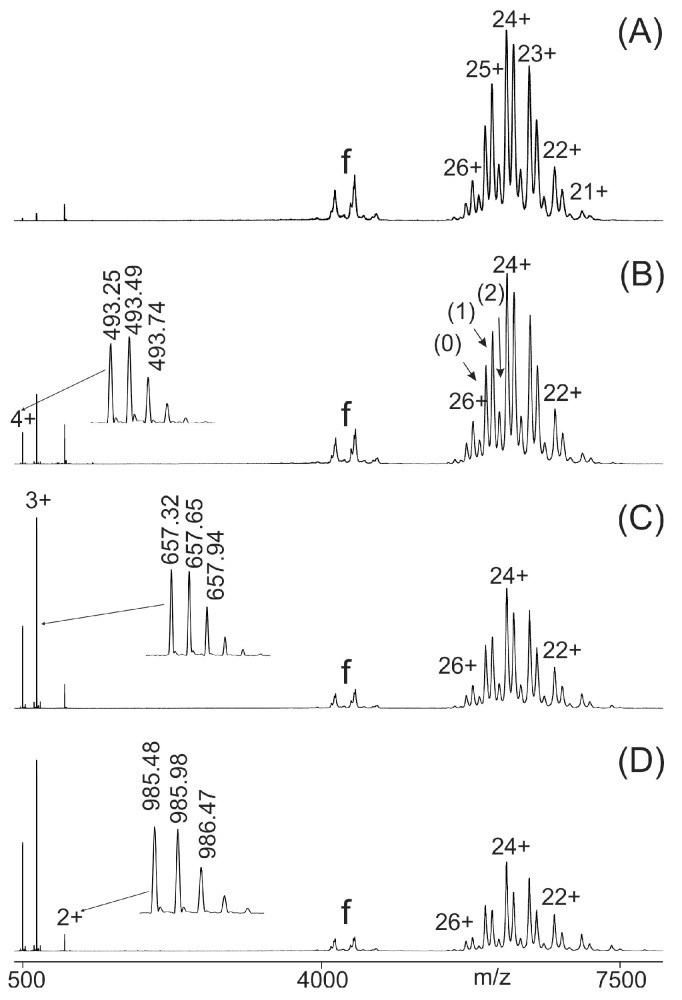
Nano-ESI mass spectra of peptide 21 (AEQQRIRNEREKERQ)–M11.7 antibody mixtures with increasing collision cell voltage differences (ΔCV). (**A**): 2 V, (**B**): 14 V, (**C**): 30 V, (**D**): 52 V. Charge states are given for the ion signals (right ion series) of the antibody (0) and the immune complexes (antibody plus one peptide (1) and antibody plus two peptides (2)). Charge states for peptide ion signals are given on the left. The inlets in (**B**), (**C**), and (**D**) show zoom views of the isotopically resolved peptide ion signals and their *m*/*z* values. Multiply charged antibody fragment ions are labeled f. The quadrupole was set to block transmission of ions < *m*/*z* 3850. The molar ratio of peptide to antibody was 13: 1. Solvent: 200 mM ammonium acetate, pH 6.7.

Very similar mass spectra were recorded for the immune complex consisting of M11.7 and its wild-type epitope peptide P21. At low ΔCV (2 V), the dominating ion signals were those of the M11.7–P21 immune complex (Figure 2A).

Additionally, some multiply charged ion signals from antibody-derived fragments were recorded. These fragment ion signals were not removed by the quadrupole mass filter, but were tolerated, as they did not interfere with ITEM-FOUR analyses. As observed previously, increasing ΔCV in the collision cell (Figure 2B–D) led to the release of epitope peptides, and the ion signal ratio shifted towards the product ions (free epitope peptide plus free antibody) at the expense of the educt ion signals (immune complexes with one and two bound epitope peptides). This phenomenon of ion intensity shifting upon increasing ΔCV was not observed when no immune complex had formed in solution, as was the case with M7, to which the cSNP-derived variant epitope peptide P12 (E138K) had been added. Then, in the high-*m*/*z* range of the mass spectrum, there were only ion signals visible for the free antibody, and blocking of ion signals of excess epitope peptide turned out not to be complete (Figure S25A). Upon increasing ΔCV in the collision cell, the antibody ion signals persisted, but the peptide ion signals started to disappear because of fragmentation (Figure S25B–D).

In all cases where immune complexes had formed with either the M7 or the M11.7 antibody upon adding a cSNP-derived variant epitope peptide in solution, the mass spectra exhibited the same phenomenon as was observed for the wild-type epitope peptides upon increasing ΔCV (Figures S26–S41).

Negative controls were investigated to confirm specificity of in-solution complex formations. As expected, mixing of an unrelated His-tag peptide with either M7 (Figure S42) or M11.7 (Figure S43) did not produce any immune complexes. Similarly, no immune complexes were detected when the wildtype epitope peptide to M11.7 (P21) was added to the M7 antibody (Figure S44) or when the wildtype epitope peptide to M7 (P11) was added to the M11.7 antibody (Figure S45). To further rule out non-specific interactions for epitope peptide P11 or epitope peptide P21, either of them was separately mixed with an unrelated anti-TNFα antibody (Figures S46 and S47). No immune complex signals were observed.

**Figure 3:**
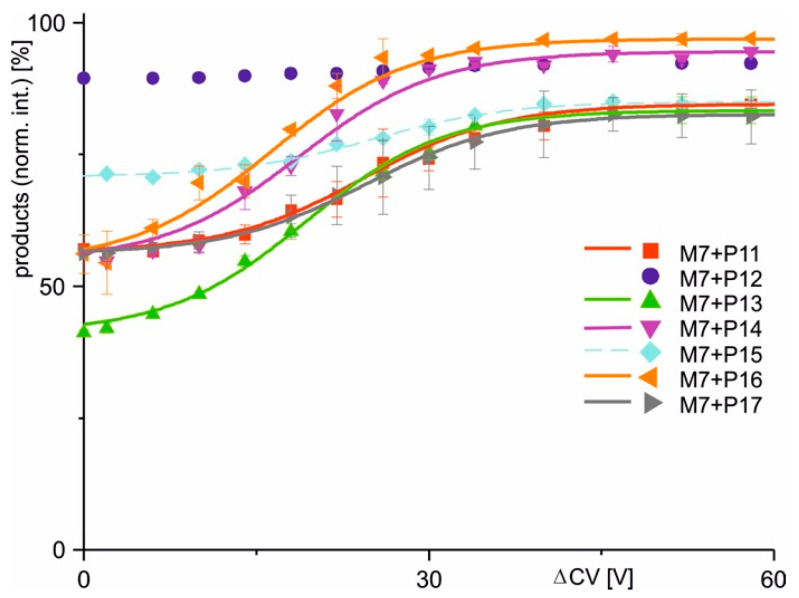
Courses of normalized product ion intensities of M7 antibody–epitope peptide complexes as functions of collision cell voltage differences (ΔCV). The immune complex dissociations with peptides P11 (red square), P12 (violet dot), P13 (green triangle), P14 (violet triangle), P15 (light blue diamond), P16 (orange triangle), and P17 (gray triangle) are shown. Each data point is the mean of two independent measurements (see Table S3). Vertical bars indicate standard deviations. The sigmoidal shaped curves were fitted using a Boltzmann function. Solid lines indicate orthodox binding. Dotted lines indicate unorthodox binding. Curve parameters are given in Table 2.

**Figure 4:**
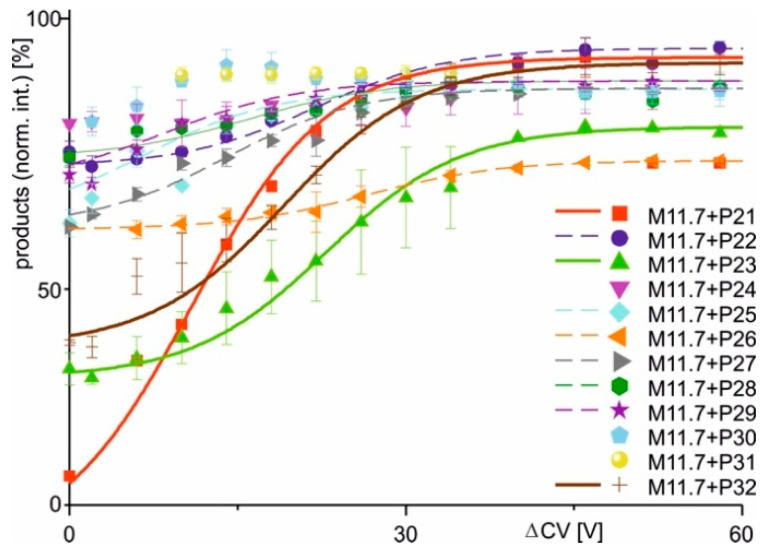
Courses of normalized product ion intensities of M11.7 antibody–epitope peptide complexes as functions of collision cell voltage differences (ΔCV). The immune complex dissociations with peptides P21 (red square), P22 (violet dot), P23 (green triangle), P24 (violet triangle), P25 (light blue diamond), P26 (orange triangle), P27 (gray triangle), P28 (green hexagon), P29 (purple star), P30 (cyan pentagon), P31 (yellow dot), and P32 (brown cross) are shown. Each data point is the mean of two independent measurements (see Table S4). Vertical bars indicate standard deviations. The sigmoidal shaped curves were fitted using a Boltzmann function. Solid lines indicate orthodox binding. Dotted lines indicate unorthodox binding. Curve parameters are given in Table 2.

After measuring all predefined antibody–(variant) epitope peptide mixtures and determining the intensities of all ions corresponding to the respective (variant) epitope peptide, antibody, and immune complex at each ΔCV setting, the mean charge states and the mean intensities of all (supra)molecular species were calculated using Gaussian curve fitting across all charge states for each molecular species (Table S2). Average peak heights and mean charge states were extracted from the apices of the Gaussian curves (Tables S3 and S4). Following normalization, the relative intensity values from two independent measurement series were averaged and plotted as functions of ΔCV (Figure 3 and Figure 4).

The two alternative scenarios, i.e., binding or non-binding, that had already been differentiated from each other when inspecting the mass spectra were substantiated with the courses of normalized product ion intensities. In cases when immune complexes had formed in solution, the dissociation courses, i.e., normalized product ion intensities as functions of increasing ΔCV, followed Boltzmann characteristics.

Closer inspection of the Boltzmann curves revealed two types of complex dissociation behaviors. In group 1, the normalized product intensities started at relatively low levels, but increased sharply around the difference in collision cell voltage required to achieve 50% dissociation (ΔCV_50_), showing a steep rise in the curve. This was observed with M7 + P11, M7 + P13, M7 + P14, M7 + P16, and M7 + P17, as well as with M11.7 + P21, M11.7 + P23, and M11.7 + P32. The differences in normalized product ion intensity values were always greater than 15 percentage points (Figure 3 and Figure 4; Table 2). In group 2, complex dissociation showed Boltzmann-like curves, where the starting points of the normalized product ion intensities remained close to those of the end points, resulting in very shallow rising curves. Differences in normalized product ion intensities were less than 15 percentage points, but greater than 5 percentage points. These conditions were seen with immune complexes M7 + P15, M11.7 + P22, M11.7 + P25, M11.7 + P26, M11.7 + P27, M11.7 + P28, and M11.7 + P29 (Figure 3 and Figure 4; Table 2). Finally, group 3 comprised cases where in-solution complex formation did not occur, resulting in flat normalized product ion intensity curves. This was observed with M7 + P12, M11.7 + P24, M11.7 + P30, and M11.7 + P31. The differences in normalized product intensity values were less than 5 percentage points (Figure 3 and Figure 4; Table 2). Based on these characteristics, each group was assigned a specific binding behavior: epitope peptides in group 1 are termed orthodox binders, those in group 2 are called unorthodox binders, and those in group 3 are classified as non-binders.

Apparent kinetic and pseudo-thermodynamic values were calculated only for group 1 (orthodox binders) epitope peptides (Table 3). This is because the steep slopes of their Boltzmann curves (Table 2) allowed for precise determination of the tangent lines, whereas the shallow slopes in group 2 introduced excessive uncertainty for further calculations that rely on an accurate slope determination of the Boltzmann curves.

Upon translating ΔCV to collision temperature ($T_{\mathrm{coll}}$) and by calculating apparent kinetic values (${\ln\;k}_{\mathrm{mg}}^{\#}$), Arrhenius plots (Figures S48 and S49) and Ellingham diagrams (Figures S50 and S51) were created and apparent kinetic and pseudo thermodynamic values were extrapolated to ambient temperature ($T_{\mathrm{amb}}$) conditions, used to describe the characteristics of gas-phase complex dissociation reactions in a more conventional fashion.

Positive ${\Delta G}_{m0g}^{\#}$ values indicate that dissociation reactions are not spontaneous, and positive ${\Delta H}_{m0g}^{\#}$ values mean that the dissociation reactions consume energy. Positive $T_{\mathrm{amb}}\;{\Delta S}_{m0g}^{\#}$ values show increases of entropy during complex dissociations. It is interesting to note that in these experiments, negative ${\Delta G}_{m0g}^{\#}$ values were only obtained upon charge conversion, i.e., when E residues were converted to K residues (P23 and P32, respectively). Yet, all ${\Delta G}_{m0g}^{\#}$ values were relatively small, i.e., less than 5 kJ/mol. Of particular interest is that in the case of M11.7, ${\Delta H}_{m0g}^{\#}$ values for the cSNP-derived variant epitope peptides P23 and P32 were smaller than those for the wild-type peptide (P21), indicating that less energy was required for the dissociation of the respective complexes. This is explained by a complex-weakening charge conversion, which is associated with the respective amino acid residue exchanges. Interestingly, peptides P13, P14, and P16 bound more strongly to M7 than the wild-type epitope peptide (P11) based on ${\Delta H}_{m0g}^{\#}$ values. This is explained by substitutions of the polar amino acid residue R (P11) with less polar amino acid residue of C in P13, W in P14, and P in P16. Larger non-polar amino acid residues exhibit larger non-polar interactions than smaller amino acid residues. This trend was in fact observed with ${\Delta H}_{m0g}^{\#}$ values of P14 (W) > P16 (P) > P13 (C).

### 2.3. Binding Motif Deduction

**Figure 5:**
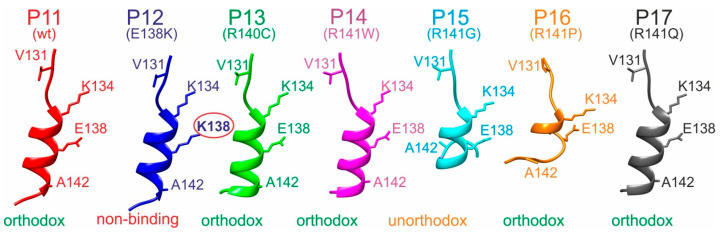
Ribbon cartoons of M7 epitope peptide backbone structure models. Alpha helices of P11 to P17 are shown. Selected amino acid residues are shown (stick model) and labeled (single-letter code). Wild-type (wt) and amino acid exchanges (point mutations) are indicated in parentheses. Amino acid numbering as in the full-length hcTnT protein. Binding modes with the M7 antibody are given at the bottom. Amino acid residues that prevent binding are circled. The “V-K-E-A” binding motif amino acid residues are shown.

The experimentally determined differences of immune complex binding strengths were combined with molecular 3D modeling approaches to deduce epitope-binding motifs. The modeled tertiary structures of all investigated peptides targeted by M7 (P11–P17) revealed a common α-helical core, spanning residues 134–138 (amino acid numbering as in the full-length hcTnT protein), and positioning K134 and E138 adjacent to each other on the same side of the helix (Figure 5 and Figure S52). The N-terminal regions (residues 130–134) were linearly stretched in all peptide models. In the case of the only non-binding peptide, P12 (E138K), in this series, the substitution resulted in K138 being positioned next to K134. Loss of binding is explained by charge conversion at this crucial epitope motif position. All other investigated peptides formed immune complexes with M7 in solution, indicating that the respective amino acid substitutions had occurred at less crucial positions with respect to antibody binding.

The only unorthodox binding peptide of the M7 series, P15, carried the R141G substitution, which led to a loss of helicity at the peptide’s C-terminal end (residues 141–144). This alteration in secondary structure allowed the C-terminal residue R144 to form a salt bridge with E138, thereby interfering with antibody binding. Notably, in peptide P16, the R141P substitution also disrupted C-terminal helicity. However, in none of the models did R144 approach E138, which can be attributed to the “unfavorable” peptide bond angles imposed on the backbone by the proline residue. Consequently, antibody binding was not affected by the R141P substitution.

When aligning the four key residues of the M7 wild-type epitope of the M7-binding 15-mer peptides on the same side of a virtually completed α-helix, a “V_131_-K_134_-E_138_-A_142_” binding motif emerges on the α-helix side where K134 and E138 are positioned. Consequently, V131 and A142 are supposed to play critical roles for binding as well. This “V_131_-K_134_-E_138_-A_142_” motif suggests that the antibody-bound peptide conformations were (nearly) fully α-helical.

**Figure 6:**
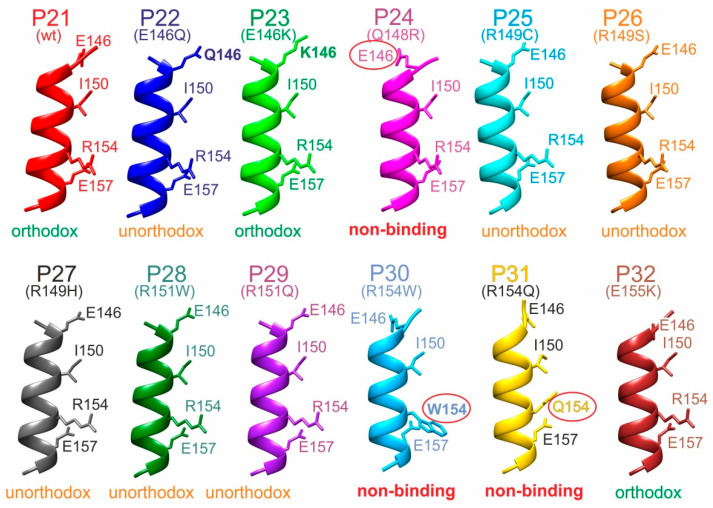
Ribbon cartoons of M11.7 epitope peptide backbone structure models. Alpha helices of P21 to P32 are shown. Selected amino acid residues are shown (stick models) and labeled (single-letter code). Wild-type (wt) and amino acid exchanges (point mutations) are indicated in parentheses. Amino acid numbering as in the full-length hcTnT protein. Binding modes with the M11.7 antibody are given at the bottom. Amino acid residues that prevent binding are circled. The “E-I-R-E” binding motif amino acid residues are shown.

Interestingly, the modeled structures of the M11.7 wild-type epitope peptide (P21) and the M11.7 targeted peptides (P22–P32) consistently formed nearly complete α-helices, with only the very N-terminal residues 145 and 146 slightly deviating from the helix structure (Figure 6 and Figure S53).

The R154W and R154Q substitutions resulted in complete losses of binding, suggesting that this R154 position is critical within the binding motif. Residues 146, 150, and 157 flanked R154 on the same side of the α-helix, forming a deduced “E_146_–I_150_–R_154_–E_157_” binding motif. Interestingly, the Q148R substitution induced bending of E146 away from the motif side due to the formation of a salt bridge between R148 and E146. This spatial displacement of E146 from the binding motif resulted in a complete loss of binding. In contrast, substituting E146 with either glutamine (Q146, P22) or lysine (K146, P23) did not hinder complex formation. Structural models suggest that in these cases, the side chains of the three residues were positioned on the binding motif side of the α-helix. This finding indicates that antibody binding likely depended not on the polar end groups of the side chains at position 146, but rather on the presence of their aliphatic stems.

All other amino acid substitutions of the M11.7 epitope allowed for formation of in-solution immune complexes, indicating that they affected positions within the epitope peptide, which are not crucial for binding.

## 3. Discussion

Typically, antibodies recognize up to about ten amino acid residues when binding to epitopes from which five or fewer “key residues” are the most critical because they are not only making physical contact with the antibody’s paratope surface, but are energetically required for binding [32,33,34]. Epitope sizes of around 1600 Å^2^ have been calculated from the AlphaFold3 structure model of the hcTnT protein (Table S5), matching well with the sizes of published epitope areas [35,36]. Notably, with α-helical epitope peptides, the assembly of up to four key amino acid residues on one and the same side of the helix has been proposed, resulting in the “V_131_–K_134_–E_138_–A_142_” motif for M7 and in the “E_146_–I_150_–R_154_–E_157_” motif for M11.7. Similarly assembled epitope motives consisting of four amino acid residues on one and the same side of the α-helical epitope have been reported [37,38] and were termed “hybrid epitopes” to assign them as falling between “consecutive” (linear) and “assembled” (conformational) epitopes [39].

**Figure 7:**
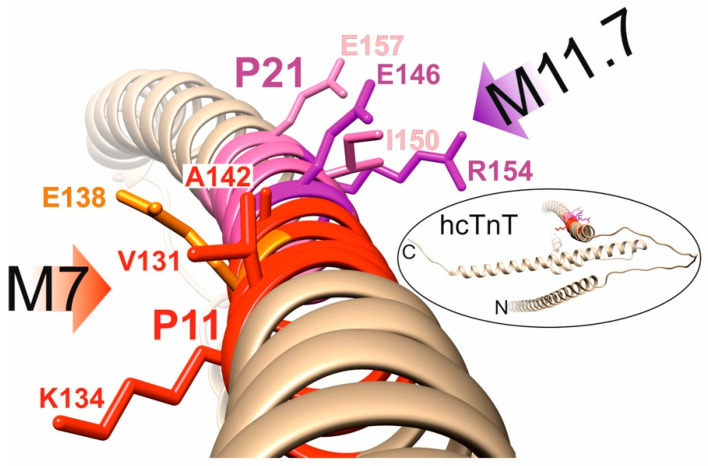
Ribbon cartoon of the hcTnT backbone structure model. The alpha helix that contains the M7 (red) and the M11.7 (pink) epitopes is shown. Selected amino acid residues are shown (stick models) and labeled (single-letter code). Amino acid residues E138 (orange), E146 (purple) and R154 (purple) are required for antibody binding. The orientations of antibody docking are indicated with red (M7) and purple (M11.7) arrows. A ribbon structure model of full-length hcTnT (UniProt accession no. P45379) is shown in the insert (circled). N- and C-termini are labeled.

The AlphaFold3 model of the full-length hcTnT protein (Figure 7 and Figure S54) suggests that both epitope peptides, P11 and P21, adopt fully α-helical secondary structures when bound to the M7 or the M11.7 antibody. The hcTnT protein model further reveals that these epitope sequences are positioned adjacent to each other on one of the three elongated α-helices of hcTnT. Additionally, the model indicates that the two binding motifs are oriented outward, facing the surrounding medium. Since they are positioned on nearly opposite sides relative to each other, this spatial arrangement ensures simultaneous accessibility to both antibodies, a crucial requirement for a sandwich ELISA assay.

For complete loss of antibody binding in some cases, it just needs a single amino acid exchange within an epitope [30,40,41,42,43], whereas in other cases, antibodies maintain binding capability despite multiple amino acid substitutions [44]. In our binding studies of cSNP-derived variant epitope peptides targeted by either the M7 or the M11.7 antibody, we found that single amino acid exchanges E138K within the M7 epitope, as well as Q148R, R154W, and R154Q, within the M11.7 epitope caused complete loss of binding. These four cases were crucial for binding motif deduction, since they singled out particular sides on the otherwise indistinguishable outward-facing partial surfaces of the α-helix.

We introduced the term “orthodox epitope–paratope binding” to describe interactions where attractive forces arise between all complementary sets of amino acid residues on molecular surfaces. These interactions occur when the physicochemical properties of one surface precisely match the positions of crucial key residues on the opposing molecular surface, either naturally or through intentional design [30]. Aberration of this “three-dimensional force code” may lead to either unorthodox binding or to complete loss of binding. With ITEM-FOUR, the distinction between orthodox and unorthodox binding can be made in a straightforward manner, as all in-solution conditions are kept constant across investigations, and mass spectrometer settings—i.e., conditions in the gas phase—are well controlled and are highly reproducible. Thus, the experimentally observed differences in complex dissociation result solely from amino acid sequence variations in the studied epitope peptides, aligning with previous investigations on the scope and limitations of the ITEM method [22,45].

Hypertrophic cardiomyopathy (HCM) is caused by mutations in genes encoding elements of the sarcomere of the cardiomyocytes, with the vast majority of mutations being familial in nature [46]. These genes include troponin T2 (*TNNT2*) [47], and molecular aberrations may lead to abnormal force generation of cardiac muscle cells, causing heart dysfunction. A significant association between SNPs rs3729547 and rs3729843 within *TNNT2* and dilated cardiomyopathy (DCM) has been found in the Chinese Han population [48]. DCM-causing mutations in hcTnT (R141W, R151W, R215L, and ΔK220) showed decreases in ATPase activation [11]. Also, an R144W mutation in the hcTnT protein of an Indian family—not investigated in this study—was associated with DCM [49] that resulted in sudden-cardiac death (SCD). Note that the R141W (rs 74315380; P14—orthodox) and R151W (rs 74315379; P28—unorthodox) amino acid substitutions, which fall within the epitopes of the M7 and the M11.7 antibody, respectively, facilitate binding of their targeted antibodies. Binding is explained by the locations of the substituted amino acid residues that are not part of the assumed antibody-binding motifs (Figure 7). As a consequence, either of the two cSNPs may impair the biological functionality of altered hcTnT, while binding to the diagnostic antibodies remains unaffected. Thus, MI assay results will retain their medically important information despite the presence of these cSNPs. The opposite case is also observed. While the Q148R substitution (rs730880232; P24—non-binding) as of yet does not appear to be associated with any cardiomyopathy, the presence of this cSNP leads to a complete loss of binding to the M11.7 antibody. Consequently, this may result in a false-negative MI assay result for carriers of this variant. The third case may be considered the most concerning, as the loss of aberrant hcTnT binding to a diagnostic antibody due to a cSNP within the epitope coincides with an increased risk of cardiomyopathy. This scenario is observed for the E138K (rs730881100; P12—non-binding), R154W (rs483352832; P30—non-binding), and R154Q (rs745632066; P31—non-binding) variants, a coincidence of unrelated events that places carriers in a double-risk scenario. On the positive side, from a clinical point of view, in carriers of these cSNPs who either have symptomatic relatives or develop symptoms themselves that raise suspicion of an underlying cardiomyopathy, genetic testing is generally recommended [50,51] and should easily be expandable to SNP determination. As a result, the potential risk of diagnostic inaccuracies in the event of a cardiac event, i.e., MI, can be recognized and may be accounted for.

To conclude, when cSNP interferences prove relevant for real-world antibody-based diagnostic assays, it may be advisable to complement point-of-care (POC) assay results with additional SNP analysis to minimize potential cSNP-related diagnostic inaccuracies. However, in clinical practice, this is not always viable due to the cost and more importantly the time required [52,53], an essential factor, particularly in suspected MI cases. Alternatively, the next generation of existing diagnostic kits could incorporate multiple highly specific antibody pairs to account for the most prevalent cSNPs within an antigen’s epitope, aligning with the principles of precision and personalized medicine [54]. For future antibody-based diagnostic kits, we recommend systematically assessing cSNP-related interference in antigen binding, addressing a currently overlooked, yet fundamental challenge in antibody-based POC diagnostics. As demonstrated here, ITEM-FOUR is well suited for fulfilling this task.

## 4. Materials and Methods

### 4.1. Preparation of Solutions with Peptides, Antibodies, and Antibody–Peptide Complexes

The synthetic hcTnT epitope peptides P11–P17, P21–P32, and the His-tag peptide were purchased from Peptides & Elephants (Peptides & Elephants GmbH, Hennigsdorf, Germany). Portions of the lyophilized peptides were weighed individually using a Microscale ME36S balance (Sartorius, Göttingen, Germany). The weighed portions of the lyophilized powders were dissolved in appropriate volumes of 200 mM ammonium acetate (pH 6.7) to yield peptide stock solutions with 1 µg/µL peptide concentrations.

Recombinant hcTnT protein was purchased from Hytest (Hytest Ltd., Turku, Finland) and obtained as lyophilized powder. To prepare hcTnT protein stock solution, 1 mg of the lyophilized hcTnT was dissolved in 500 µL of radioimmunoprecipitation assay (RIPA) buffer (50 mM TRIS-HCl, pH 8.0; 150 mM NaCl; 1% Triton X-100; 0.5% sodium deoxycholate (SDC); 0.1% sodium dodecyl sulfate (SDS)). Determination of protein concentration resulted in a value of 3.5 µg/µL.

To obtain M7 and M11.7 antibody stock solutions, portions of the lyophilized powders, which were provided courtesy of Roche (Roche Diagnostics International, Rotkreuz, Switzerland), were weighed individually. Then, 1.25 mg of lyophilized M7 was dissolved in 600 µL of PBS and 1.7 mg of lyophilized M11.7 was dissolved in 800 µL of PBS. Protein concentrations were 0.5 µg/µL for the M7 and 0.7 µg/µL for the M11.7 stock solutions.

The anti-TNFα antibody stock solution (2 µg/µL) was prepared from lyophilized antibody powder (article number: MAS: 23720, ThermoFisher Scientific) and 200 mM ammonium acetate (pH 6.7).

A secondary antibody stock solution with 1 µg/µL of IRDye 800CW-conjugated polyclonal anti-mouse antibody from goat (isotype: IgG; article number: 926-32210, lot number: C60726-02, LI-COR Biosciences, Lincoln, NE, USA) was prepared by dissolving the lyophilized powder in PBS (140.0 mM NaCl, 10.0 mM Na_2_HPO_4_. 2 H_2_O, 2.7 mM KCl, 1.8 mM KH_2_PO_4_, pH 7.4).

To prepare peptide working solutions, the peptide stock solutions were diluted with 200 mM ammonium acetate (pH 6.7). Peptide working solutions were adjusted to peptide concentrations of 0.1 µg/µL for nano-ESI-MS analyses and to 0.02 µg/µL for ITEM-FOUR experiments by adding the appropriate volumes of 200 mM ammonium acetate, pH 6.7.

To generate antibody working solutions, the antibody stock solutions were rebuffered according to described procedures [55,56]. In brief, 80 μL aliquots of the antibodies M7 and M11.7 were individually pipetted into separate 50 K centrifugal filter devices (Merck Millipore, Carrigtwohill, Ireland), with a cut-off value of 50 kDa, and 400 μL of 200 mM ammonium acetate (pH 6.7) was added to each. After centrifugation at 13,000 rpm for 10 min in an Eppendorf centrifuge (MiniSpin, Eppendorf, Hamburg, Germany), the flowthrough was discarded and the filter was filled again with 400 μL of 200 mM ammonium acetate (pH 6.7). This procedure of centrifugation, discarding, and refilling was repeated eight times. After the final centrifugation step, the filters were placed upside down in a new tube and centrifuged for 5 min at 4500 rpm. Approximately 50 μL of each antibody solution was collected. Protein concentrations were 3.62 µg/µL and 4.6 µg/µL for M7 and M11.7 antibody solutions, respectively. For the anti-TNFα antibody, rebuffering into 200 mM ammonium acetate, pH 6.7, using the procedure described above afforded a protein concentration of 0.8 µg/µL. All antibody working solutions were adjusted to 0.2 µg/µL protein concentration by dilution with 200 mM ammonium acetate (pH 6.7). A volume of 5 µL of hcTnT protein stock solution was diluted with 82.5 µL of RIPA buffer to achieve a concentration of 0.2 µg/µL of the hcTnT working solution.

Antibody–peptide mixture solutions were prepared for ITEM FOUR experiments by mixing 5 µL of one antibody working solution with one assigned peptide working solution to generate antibody to peptide molar ratios of 1:2.1 for all solutions that contained the M7 antibody. For mixture solutions containing the M11.7 or the anti-TNFα antibody, 1:13 molar ratios were prepared. Antibodies were incubated together with peptides for at least one hour at room temperature prior to analysis.

### 4.2. Protein and Peptide Concentration Determination

Protein concentrations of the antibody and peptide stock solutions were determined using the Qubit™ 2.0 fluorometer (Invitrogen by Life Technologies/Thermo Fisher Scientific, Waltham, MA, USA) as described in [22,57]. The protein concentration of the hcTnT solution was determined using the Bradford assay as described in [58,59].

### 4.3. SDS-PAGE Analysis of the hcTnT Antigen

The purity of the hcTnT antigen was assessed by SDS-PAGE analysis [60,61]. For SDS-PAGE analysis, two antigen solutions (antigen solution 1 and antigen solution 2) with final volumes of 12.5 µL each were prepared. Antigen solution 1, containing 1 μg of hcTnT, was prepared by mixing 5 µL of hcTnT working solution with 5 µL of deionized water and 2.5 µL of SDS sample buffer (312.5 mM TRIS-HCl pH 6.8, 10% SDS, 325 mM DTT, 50% glycerol, 0.4% bromophenol blue). Antigen solution 2, containing 2 μg of hcTnT, was prepared by mixing 10 µL of hcTnT working solution with 2.5 µL of SDS sample buffer. Afterwards, antigen solutions 1 and 2 were heated at 95 °C for 5 min, cooled at 4 °C for 10 min and centrifuged at 13.000 rpm for one minute. A 10% SDS gel (82 mm × 68 mm × 1 mm, Thermo Fisher Scientific) was placed in an XCell SureLock^TM^ chamber (Thermo Fisher Scientific), which was filled with 500 mL of 3-morpholinopropane-1-sulfonic acid (MOPS) buffer containing 0.05 M MOPS, 0.05 M TRIS, 3.465 mM SDS, and 0.76% Titriplex III. Finally, 3 μL of PageRuler Prestained Protein Ladder marker solution (Thermo Fisher Scientific, Waltham, MA, USA) were loaded in pocket 1. Antigen solution 1 was loaded in pocket 2 and antigen solution 2 was loaded in pocket 3. The lid was placed on the chamber, the electrodes were connected to the power supply, and electrophoresis was performed at a constant voltage of 200 V for approximately 1 h. Following electrophoresis, the gel was subjected to protein fixation. For fixation, the gel was placed in a dish that contained 50 mL of a solution containing 50% ethanol and 10% acetic acid. The dish was placed on a PROMAX 2020 shaker (Heidolph Scientific Products GmbH, Schwabach, Germany). Incubation lasted for one hour with gentle shaking at room temperature. The fixation solution was removed and 50 mL of staining solution (1.5 l deionized water, 0.4 g Coomassie Brilliant Blue G250, 100 g aluminum sulfate-(14-18)-hydrate, 46 mL 85% ortho-phosphoric acid, adjusted to a total volume of 2 L with deionized water) was added. Again, the gel, which was placed in the dish on the shaker, was incubated for 16 h at room temperature [62]. After staining, the solution was discarded, and the gel was washed three times for 20 min each with 50 mL of destaining solution (10% ethanol (96%), 2.3% ortho-phosphoric acid (85%), deionized water) on a shaker until the background staining faded, leaving only the protein bands visible. An image of the gel was acquired using a ScanMaker 1000XL scanner (Microtek, Hsinchu City, Taiwan) with a resolution of 300 dpi. The image was saved as an RGB 24-bit tif file.

### 4.4. Western Blot Analysis of the Anti-hcTnT Antibodies

To assess the antibodies’ performance, the monoclonal antibodies M7 and M11.7 were investigated as primary antibodies by Western blot analysis [61,63,64]. A volume of 5 µL of hcTnT protein stock solution was diluted with 170 µL of RIPA buffer to achieve a final concentration of 0.1 µg/µL. Afterwards, two antigen solutions were prepared, antigen solutions 1 and 2, both with final volumes of 12.5 µL. hcTnT antigen solution 1 was prepared by mixing 2.5 µL of hcTnT protein working solution with 7.5 µL of deionized water and 2.5 µL of SDS sample buffer. Antigen solution 2 was prepared by mixing 5 µL of hcTnT protein working solution with 5 µL of deionized water and 2.5 µL of SDS sample buffer. Antigen solutions 1 and 2 were heated at 95 °C for 5 min, cooled at 4 °C for 10 min, and centrifuged at 13.000 rpm for one minute. The pockets of a 10% SDS-gel (82 mm × 68 mm × 1 mm, Thermo Fisher Scientific) were loaded as follows: lanes 1, 4, and 7—molecular mass marker (3 µL); lanes 2, 5, and 8—antigen solution 1; lanes 3, 6, and 9—antigen solution 2. The SDS-PAGE was performed as described above. After SDS-PAGE separation, proteins were transferred onto a polyvinylidene difluoride (PVDF) membrane (8 cm width, 6.5 cm length; pore width: 0.45 nm, item number: IPFL00010, lot number: K8PN6324A, Immobilon, Millipore Sigma, Bedford, MA, USA). Filter papers of 8 × 6.5 cm^2^ dimensions (GB 002, item number: 10426694, Schleicher & Schuell, Keene, NH, USA) were used for semidry blotting. In detail, the gel was placed in a dish that contained 50 mL of ε-aminocaproic acid buffer (0.04 M ε-aminocaproic acid, 0.025 M TRIS, 20% methanol, pH 9.4) and incubated with gentle shaking for 10 min at room temperature. The PVDF membrane was wetted first with isopropanol, then with deionized water and kept in a dish that contained 50 mL of low TRIS (LT) buffer (0.025 M TRIS, 20% methanol, pH 10.4) at room temperature for 10 min. Then, the bottom plate of a Pegasus blotting device (Phase, Lübeck, Germany) was wetted with ε-aminocaproic acid buffer. Nine layers of ε-aminocaproic acid buffer-soaked filter papers were placed on the wetted bottom plate, followed by the gel, the wetted PVDF membrane, and three layers of low TRIS (LT) buffer-soaked filter papers. Finally, 6 layers of filter paper soaked with high TRIS (HT) buffer (0.3 M TRIS, 20% methanol, pH 10.4) were added on top. The top plate of the blotting device was wetted with high TRIS (HT) buffer also [65]. Proteins were transferred onto the PVDF membrane under a constant current of 48 mA (1.2 mA/cm^2^) for approximately 1 h. Following the protein transfer, the PVDF membrane was stained with 10 mL of Ponceau S solution (0.2% Ponceau S, 3% trichloro-acetic acid) for 2 min and destained with 20 mL of deionized water three times for 2 min each. The PVDF membrane was cut into three pieces such that each piece contained three lanes, one marker, and two hcTnT antigens. Surfaces were blocked, each in 10 mL of a 1:1 mixture of ready-to-use Intercept Blocking Buffer (lot number: 927-60001, LI-COR) and buffer. The membrane strips were separately incubated for 1 h at room temperature. Then, the blocking buffers were removed from strips 1 and 2 and M7 antibody and M11.7 were added, respectively, whereas the membrane strip 3 was left untouched. For antibody incubation, 8 µg of M7 (from the M7 working solution) and 8 µg of M11.7 (from the M11.7 working) were added to 8 mL of Intercept Blocking Buffer/PBS (1:1 *v*/*v*) with 0.1% (*v*/*v*) Tween-20 each. Primary antibody and mock incubations lasted for 16 h at 4 °C each. Afterwards, the membrane strips were washed four times with 10 mL of washing buffer (140 mM PBS with 0.1% Tween-20) for five minutes each [66]. After washing, secondary antibody incubation was performed with the three membrane strips in parallel. A volume of 2 µL of the secondary antibody stock solution was added to 30 mL of Intercept Blocking Buffer/PBS (1:1 *v*/*v*) with 0.1% (*v*/*v*) Tween-20. Each membrane strip was separately incubated in 8 mL of this secondary antibody solution for 1 h at room temperature in the dark on a shaker. After discarding the secondary antibody-containing solutions, the PVDF membrane strips were washed again with 10 mL washing buffer four times for 5 min each. Then, the strips were washed with 10 mL PBS for 5 min to remove the Tween20. After this, the membrane strips were forwarded to imaging. The detection of antibody-decorated proteins was achieved using an Odyssey Infrared Imaging System (LI-COR) that was set to 800 nm (solid-state diode laser emits at 785 nm) and by applying recording conditions as described [66]. For semiquantitative analysis, the fluorescence intensity of the bands was evaluated using the Image Studio Light software (LI-COR, version no. 5.2). Blot images were stored as tif files and blot membrane strips were discarded after imaging [67].

### 4.5. Mass Calibration of Mass Spectrometry Instruments

The Q-ToF 2 instrument was calibrated with 1% phosphoric acid made from 85% ortho-phosphoric acid in 2,2,2-trifluoro ethanol (item number: 101055731, Sigma Aldrich, Saint Louis, MI, USA)/deionized water (item number: 7343.1, Carl Roth, Karlsruhe, Germany) (1:1 *v*/*v*) [22]. For mass calibration of the Synapt G2S mass spectrometer (Waters MS-Technologies, Manchester, UK) a solution of 1 mg/mL sodium iodide (item number: 71710, Fluka Chemika, Buchs, St. Gallen, Switzerland) and isopropanol/deionized water, 1:1 *v*/*v*) was used [22].

### 4.6. Preparation of Nano-ESI-MS Emitters, Filling and Mounting

Nano-ESI emitters for offline nano-ESI mass spectrometry were made from borosilicate glass capillaries (item number: BF100-50-10, Sutter Instrument Company, Novato, CA, USA) as described in [45]. Two emitter needles were produced from one glass capillary with an inner diameter of 0.5 mm and an outer diameter of 1.0 mm using a P-1000 Flaming/Brown^TM^ Micropipette Puller System (Sutter Instruments). To achieve even lengths of about 5 cm each, the emitter needles were cut at the blunt ends. The emitter needles were subsequently gold-coated using a BalTec SCD 005 (Bal-Tech, Balzers, Liechtenstein) sputter coater. Emitter needles were placed about 5 cm away from the gold target. Under an oxygen-free atmosphere, which was achieved in the coating chamber by first applying a vacuum and secondly an argon gas pressure of about 0.5 mbar, the emitter needles were gold-coated for 150 s using an electric current of 20 mA [22,30,55,68].

Volumes of 3 µL of antibody, peptide, or antibody–peptide mixture solutions were loaded into separate emitter needles using micro-loader pipette tips (item number 5242956.003, Eppendorf, Hamburg, Germany). Filled emitter needles were mounted onto the ion source holder of the Q-ToF 2 instrument or the Synapt G2S mass spectrometer (Waters MS-Technologies, Wilmslow, UK) for performing either mass spectrometric molecular mass determinations or ITEM FOUR experiments [30,68,69].

### 4.7. Q-ToF 2 Instrument Settings and Data Acquisition

Molecular masses of peptides were obtained using the Q-ToF 2 mass spectrometer (Waters MS-Technologies, Wilmslow, UK) [22]. Data acquisition was performed with the following instrumental settings: source temperature, 40 °C; capillary voltage, 1.0 kV; sample cone voltage, 30 V; extractor cone voltage 30 V; collision voltage, 2 V; pusher time, 124 µs. All mass spectra were acquired in positive-ion mode applying a mass window of *m*/*z* 0–2000. The quadrupole analyzer was set to full transmission. Individual scans were integrated to obtain an average spectrum from which ion intensities were extracted. Data were acquired and processed using MassLynx software version 4.1 (Waters MS-Technologies, Wilmslow, UK) [30]. For peptide-mass determinations, 3 smoothing cycles with a window of 2 mass units were applied using the Savitzky–Golay method. All measurements were recorded in duplicate. The mass spectrometric raw data have been deposited via the ProteomeXchange Consortium in the PRIDE partner repository with the dataset identifier PXD058812 [70].

### 4.8. Synapt G2S Instrument Settings and Data Acquisition

Data acquisition for ITEM-FOUR experiments was performed as described in [18] with the Synapt G2S mass spectrometer (Waters MS-Technologies, Wilmslow, UK) and with the following instrumental settings: source temperature, 40 °C; capillary voltage, 1.0–1.3 kV; sample cone voltage, 130 V; source offset voltage, 130 V; trap gas flow (argon), 8.0 mL/min; helium gas: 20 bar. All mass spectra were acquired in positive-ion mode applying a mass window of *m*/*z* 200–8000. The quadrupole analyzer was used to block transmission of lower-molecular-mass ions: M1 = 5000 with dwell time of 25% and ramp time of 25%; M2 = 5000 with dwell time of 25% and ramp time of 25%; M3 = 5000. ΔCV in the TRAP collision cell was increased stepwise as follows: 0, 2, 6, 10, 14, 18, 22, 26, 30, 34, 40, 46, 52 and 58 V. The ion mobility separation mode was not used. All acquired scans during a given collision cell voltage difference setting were integrated to obtain an averaged spectrum from which ion intensities were extracted for ITEM-FOUR calculations. Data were acquired and processed with MassLynx software version 4.1 (Waters MS-Technologies) [71]. For exact time stamps of all measurements, refer to Table S2 in the Supplement (see also the deposited raw data supplement in the PRIDE directory). All mass spectra for each ΔCV setting were smoothed using the Savitzky-Golay method. Spectra of antibody solutions and antibody–peptide mixture solutions were smoothed using 30 cycles with a 15-mass unit window at the high mass end and at the low mass end with 3 cycles and a 2-mass unit window. All measurements were recorded in duplicate. The mass spectrometric raw data have been deposited via the ProteomeXchange Consortium in the PRIDE partner repository with the dataset identifier PXD058812 [70].

### 4.9. ITEM-FOUR Spectral Data Analysis

The Origin Pro 2023b (64-bit, OriginLab Corporation, Northampton, MA, USA) software package with an automation script for processing multiple datasets was used to graphically plot the extracted intensities of the ion signals of all relevant molecular entities (the immune complex with two bound peptides, the immune complex with one bound peptide, free antibodies, and free peptides) against their corresponding charge state for each collision cell voltage difference (ΔCV) [22]. Subsequently, Gaussian curve fittings were performed separately for each molecular entity. Since there are at least five values needed for a Gaussian fit and the number of peptides singly and multiply charged ions did not add up to this number, further values were automatically imputed and checked manually. Iterations were performed for R2 values to be at least 0.91. Apices of Gaussian curves provided the intensities and the average *m*/*z* values for each molecular species. Intensity values of individual molecular entities (educts and products) were summed and normalized. Normalized intensities of educts and products from duplicate measurements were averaged.

### 4.10. ITEM-FOUR Calculations of Apparent Kinetic and Apparent Thermodynamic Values

The number of atoms from rituximab [33] was used because the amino acid sequences of neither the M7 nor the M11.7 antibody were disclosed. Average intensity values of normalized product intensities were plotted against the ΔCV settings together with their standard deviations. Boltzmann curves were fitted to represent the courses of product intensities using the OriginPro software package. All fits reached R2 values of at least 0.96. Boltzmann curve parameters were used for calculating the tangents along the steep parts of the Boltzmann curves. The mathematical procedures for calculations of apparent kinetic and thermodynamic values $k_{m0g}^{\#}$, $K_{D\;m0g}^{\#}$, ${\Delta G}_{m0g}^{\#}$, ${\Delta H}_{m0g}^{\#}$, and $T_{\mathrm{amb}}\;{\Delta S}_{m0g}^{\#}$ were followed as described elsewhere [22,55].

### 4.11. Molecular Modeling of Protein and Peptide Structures

Three-dimensional models of the hcTnT protein and the synthetic peptides P11–P17 and P21–P32 were created using AlphaFold3 (https://alphafold.ebi.ac.uk/entry/P45379, accessed on 13 January 2025) [72] and Pepfold4 (https://bioserv.rpbs.univ-paris-diderot.fr/services/PEP-FOLD/, accessed on 27 October 2024), respectively [73,74]. Based on the AlphaFold3 model of hcTnT (UniProt accession: P45379) the total accessible surface areas of hcTnT as well as of partial accessible epitope surfaces were calculated using the in-house IndyMedSurfacer tool.

## Figures and Tables

**Table 1:** Single-nucleotide polymorphism and molecular information of the anti-hcTnT antibodies’ epitope regions.

Peptide No. (wt or SAP) ^(a,b)^	Amino Acid Sequence (wt or SAP) ^(a,b)^	SNP Entry ^(c)^	SNP ^(c)^	Cardiomyopathy Association ^(c)^	Atom No.	M_r_ (Mono) ^(d)^	MM (Exp.) ^(e)^
11 (wt)	LVSLKDRIERRRAER	n.a.	n.a.	n.a.	278	1896.14	1896.91
12 (E138K)	LVSLKDRI**K**RRRAER	rs 730881100	G > A	hypertrophic	283	1896.27	1896.44
13 (R140C)	LVSLKDRIER**C**RAER	rs 397516463	C > T	hypertrophic/familial restrictive	264	1842.03	1842.09
14 (R141W)	LVSLKDRIERR**W**AER	rs 74315380	C > T	dilated	279	1926.11	1926.36
15 (R141G)	LVSLKDRIERR**G**AER	rs 74315380	C > G	not provided	262	1797.07	1797.19
16 (R141P)	LVSLKDRIERR**P**AER	rs 397516464	G > C	dilated	269	1837.08	1837.43
17 (R141Q)	LVSLKDRIERR**Q**AER	rs 397516464	G > A	dilated	272	1868.09	1868.18
21 (wt)	AEQQRIRNEREKERQ	n.a.	n.a.	n.a.	274	1969.04	1969.67
22 (E146Q)	A**Q**QQRIRNEREKERQ	rs 371142225	G > C	dilated	275	1968.07	1968.78
23 (E146K)	A**K**QQRIRNEREKERQ	rs 371142225	G > A	dilated	279	1968.10	1969.02
24 (Q148R)	AEQ**R**RIRNEREKERQ	rs 730880232	A > G	not provided	280	1997.08	1997.43
25 (R149C)	AEQQ**C**IRNEREKERQ	rs 397516465	C > T	familial dilated	261	1914.94	1915.46
26 (R149S)	AEQQ**S**IRNEREKERQ	rs 397516465	C > A	not provided	262	1899.96	1899.56
27 (R149H)	AEQQ**H**IRNEREKERQ	rs 397516466	G > A	dilated	268	1950.00	1951.24
28 (R151W)	AEQQRI**W**NEREKERQ	rs 74315379	C > T	dilated	275	1999.03	2000.02
29 (R151Q)	AEQQRI**Q**NEREKERQ	rs 730881101	G > A	familial restrictive	268	1941.00	1941.99
30 (R154W)	AEQQRIRNE**W**EKERQ	rs 483352832	C > T	dilated	275	1999.03	2000.95
31 (R154Q)	AEQQRIRNE**Q**EKERQ	rs 745632066	G > A	familial restrictive	268	1941.00	1941.90
32 (E155K)	AEQQRIRNER**K**KERQ	rs 984218824	G > A	familial restrictive	279	1968.10	1969.03

^(a)^ aa130-aa144 and aa145-aa159 from hcTnT (UniProt: P45379), which are the epitope regions of the monoclonal anti-hcTnT antibodies M7 and M11.7, respectively; ^(b)^ amino acid exchanges in peptides are printed in bold; wt: wild type; SAP: single-amino-acid polymorphism. ^(c)^ SNP: single-nucleotide polymorphism; n.a.: not applicable; ^(d)^ mono: monoisotopic mass; ^(e)^ MM: molecular mass.

**Table 2:** Course characteristics of gas phase dissociation reactions of anti-hcTnT antibody–epitope peptide complexes.

Complex ^(a)^	Peptide Sequence	Mean Charge ± SD. ^(b,c)^	Initial (%) ^(b,c,d)^	Final (%) ^(b,c,e)^	Δ (% pts)	ΔCV_50_ (V) ^(b)^	dx (V) ^(b)^	Slope (%/V) ^(b)^	R^2 (b,c)^
M7 + P11	LVSLKDIERRRAER	24.1 ± 0.23	56.43	84.36	27.93	25.27	6.69	1.04	0.995
M7 + P12	LVSLKDI**K**RRRAER	24.5 ± 0.67	89.38	92.28	2.90	n.a.	n.a.	n.a.	n.a.
M7 + P13	LVSLKDIER**C**RAER	24.3 ± 0.29	40.36	83.40	43.04	23.30	6.55	1.64	0.999
M7 + P14	LVSLKDIERR**W**AER	24.1 ± 0.22	54.37	93.86	39.49	18.02	4.33	2.28	0.997
M7 + P15	LVSLKDIERR**G**AER	24.1 ± 0.01	70.69	84.98	14.29	24.89	6.09	0.59	0.995
M7 + P16	LVSLKDIERR**P**AER	24.2 ± 0.15	55.34	96.90	41.56	15.83	5.05	2.06	0.993
M7 + P17	LVSLKDIERR**Q**AER	24.0 ± 0.01	56.18	82.69	26.51	24.61	6.46	1.03	0.999
M11.7 + P21	AEQQRIRNEREKERQ	23.6 ± 0.11	56.91	96.29	39.38	12.77	5.77	1.71	0.996
M11.7 + P22	A**Q**QQRIRNEREKERQ	23.7 ± 0.10	87.08	97.12	10.04	25.18	7.70	0.33	0.970
M11.7 + P23	A**K**QQRIRNEREKERQ	23.3 ± 0.01	67.00	89.77	22.77	22.47	7.29	0.78	0.994
M11.7 + P24	AEQ**R**RIRNEREKERQ	23.3 ± 0.06	90.25	93.57	3.32	n.a.	n.a.	n.a.	n.a.
M11.7 + P25	AEQQ**C**IRNEREKERQ	23.2 ± 0.17	80.75	93.33	12.58	10.06	6.00	0.52	0.962
M11.7 + P26	AEQQ**S**IRNEREKERQ	22.9 ± 0.00	80.48	86.48	6.00	26.67	6.49	0.25	0.996
M11.7 + P27	AEQQ**H**IRNEREKERQ	23.1 ± 0.07	80.22	93.49	13.27	14.04	7.60	0.44	0.987
M11.7 + P28	AEQQRI**W**NEREKERQ	23.4 ± 0.01	86.91	93.90	6.99	13.80	6.90	0.25	0.955
M11.7 + P29	AEQQRI**Q**NEREKERQ	23.2 ± 0.15	84.39	93.92	9.53	9.96	5.17	0.46	0.981
M11.7 + P30	AEQQRIRNE**W**EKERQ	23.5 ± 0.09	90.48	94.03	3.55	n.a.	n.a.	n.a.	n.a.
M11.7 + P31	AEQQRIRNE**Q**EKERQ	23.2 ± 0.02	93.60	93.39	−0.21	n.a.	n.a.	n.a.	n.a.
M11.7 + P32	AEQQRIRNER**K**KERQ	23.1 ± 0.06	69.09	96.03	26.94	17.63	7.32	0.92	0.992

^(a)^ Multiply charged and accelerated complexes. ^(b)^ Averaged from two acquisitions. ^(c)^ Dimensionless number. ^(d)^ Product quantity at the lowest applied ΔCV50 value. ^(e)^ Product quantity at the highest applied ΔCV50 value.

**Table 3:** Apparent kinetic and pseudo thermodynamic values for anti-hcTnT antibody–epitope peptide complex dissociations.

Complex ^(a)^	Peptide Sequence	$k_{m0g}^{\#}\;$ ^(b)^ [1/s]	$K_{D\;m0g}^{\#}\;$ ^(b,c)^ [Ø]	$\Delta G_{m0g}^{\#}$ ^(b)^ [kJ/mol]	$\Delta H_{m0g}^{\#}$ ^(b)^ [kJ/mol]	$T_{amb}\;\Delta S_{m0g}^{\#}$ ^(b)^ [kJ/mol]	Binding Type
M7 + P11	LVSLKDIERRRAER	3.69∙10^12^	0.59	1.28	40.25	38.96	orthodox
M7 + P12	LVSLKDI**K**RRRAER	n.a.	n.a.	n.a.	n.a.	n.a.	non-binding
M7 + P13	LVSLKDIER**C**RAER	1.72∙10^12^	0.28	3.17	55.16	51.99	orthodox
M7 + P14	LVSLKDIERR**W**AER	1.75∙10^12^	0.28	3.13	92.09	88.96	orthodox
M7 + P15	LVSLKDIERR**G**AER	n.a.	n.a.	n.a.	n.a.	n.a.	unorthodox
M7 + P16	LVSLKDIERR**P**AER	2.95∙10^12^	0.48	1.84	85.61	83.77	orthodox
M7 + P17	LVSLKDIERR**Q**AER	3.82∙10^12^	0.62	1.14	38.06	36.92	orthodox
M11.7 + P21	AEQQRIRNEREKERQ	5.70∙10^12^	0.92	0.21	72.53	72.32	orthodox
M11.7 + P22	A**Q**QQRIRNEREKERQ	n.a.	n.a.	n.a.	n.a.	n.a.	unorthodox
M11.7 + P23	A**K**QQRIRNEREKERQ	7.33∙10^12^	1.18	−0.42	37.41	37.81	orthodox
M11.7 + P24	AEQ**R**RIRNEREKERQ	n.a.	n.a.	n.a.	n.a.	n.a.	non-binding
M11.7 + P25	AEQQ**C**IRNEREKERQ	n.a.	n.a.	n.a.	n.a.	n.a.	unorthodox
M11.7 + P26	AEQQ**S**IRNEREKERQ	n.a.	n.a.	n.a.	n.a.	n.a.	unorthodox
M11.7 + P27	AEQQ**H**IRNEREKERQ	n.a.	n.a.	n.a.	n.a.	n.a.	unorthodox
M11.7 + P28	AEQQRI**W**NEREKERQ	n.a.	n.a.	n.a.	n.a.	n.a.	unorthodox
M11.7 + P29	AEQQRI**Q**NEREKERQ	n.a.	n.a.	n.a.	n.a.	n.a.	unorthodox
M11.7 + P30	AEQQRIRNE**W**EKERQ	n.a.	n.a.	n.a.	n.a.	n.a.	non-binding
M11.7 + P31	AEQQRIRNE**Q**EKERQ	n.a.	n.a.	n.a.	n.a.	n.a.	non-binding
M11.7 + P32	AEQQRIRNER**K**KERQ	8.85∙10^12^	1.43	−0.88	50.35	51.23	orthodox

^(a)^ Multiply charged and accelerated complex; ^(b)^ n.a.: not applicable; ^(c)^ dimensionless number.

## Data Availability

The mass spectrometric raw data have been deposited via the ProteomeXchange Consortium in the PRIDE partner repository with the dataset identifier PXD058812.

## Supplementary Materials

The following supporting information can be downloaded at https://www.mdpi.com/article/10.3390/ijms26104892/s1.

## List of Abbreviations

Å	Ångström
ACC	American College of Cardiology
anti-TNFα	anti-tumor necrosis factor alpha
BSA	bovine serum albumin
cSNP	coding single-nucleotide polymorphism
cTnT	cardiac troponin T
DCM	dilative cardiomyopathy
dbSNP	single-nucleotide polymorphism database
ELISA	enzyme-linked immunosorbent assay
ESC	European Society for Cardiology
ESI	electrospray ionization
HCM	hypertrophic cardiomyopathy
hcTNT	human cardiac troponin T
hs	high-sensitivity
ITEM	intact transition epitope mapping
ITEM FOUR	intact transition epitope mapping—force differences between original and unusual residues
kDa	kiloDalton
MI	myocardial infarction
*m*/*z*	mass-to-charge ratio
nano-ESI-MS	nano-electrospray ionization–mass spectrometry
PBS	phosphate-buffered saline
POC	point of care
PRIDE	PRoteomics IDEntification
PVDF	polyvinylidene fluoride
Q-ToF	quadrupole time of flight
RCM	restrictive cardiomyopathy
rpm	revolutions per minute
RIPA	radioimmunoprecipitation assay
SDC	sodium deoxycholate
SDS	sodium dodecyl sulfate
SDS-PAGE	sodium dodecyl sulfate–polyacrylamide gel electrophoresis
SNP	single-nucleotide polymorphism
tif	tagged image file
Tn	troponin
TnI	troponin I
*TNNT2*	troponin T gene
TnT	troponin T
TRIS	tris(hydroxymethyl)aminomethane
ΔCV	voltage difference in collision cell
3D	three-dimensional
