# Supplementary material for: Mass Spectrometric ITEM-FOUR Analysis Reveals Coding Single-Nucleotide Polymorphisms in Human Cardiac Troponin T That Evade Detection by Sandwich ELISAs Which Use Monoclonal Antibodies M7 and M11.7 from the Elecsys Troponin T® Assay
Source: Int J Mol Sci. 2025 May 20;26(10):4892. doi: 10.3390/ijms26104892 (PMC12112476; doi:10.3390/ijms26104892)
Supplement: Supplementary file 1 [file ijms-26-04892-s001.zip › ijms-3608576-supplementary.pdf]

Mass spectrometric ITEM-FOUR analysis reveals coding single nucleotide polymorphisms in human cardiac troponin T that evade detection by sandwich ELISAs which use monoclonal antibodies M7 and M11.7 from the Elecsys Troponin T® assay

Kristjan Kormann, Manuela Ruß, Claudia Röwer, Cornelia Koy, and Michael O. Glocker

Proteome Center Rostock Medical Faculty and Natural Science Faculty, University of Rostock
Schillingallee 69, 18057 Rostock, Germany

Supplement

Supplemental Figures page 2
Supplemental Tables page 33

Supplemental Figures

1) Monoclonal antibodies

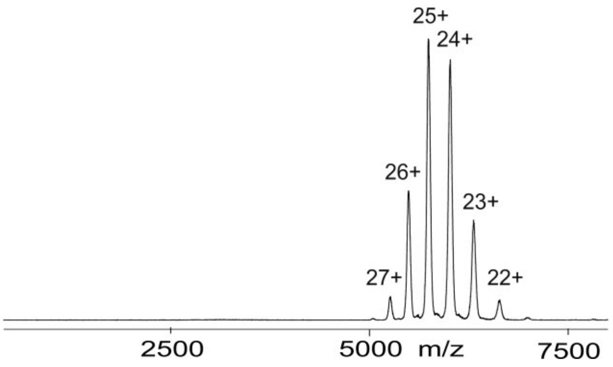

Figure S1: NanoESI mass spectrum of the monoclonal antibody M7. Charge states of ion signals are given. For m/z values see Table S1. Protein concentration: 0.2 µg/µl. Solvent: 200 mM ammonium acetate, pH 6.7.

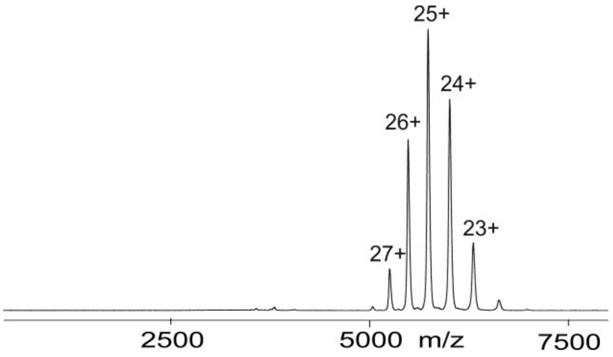

Figure S2: NanoESI mass spectrum of the monoclonal antibody M11.7. Charge states of ion signals are given. For m/z values see Table S1. Protein concentration: 0.2 µg/µl. Solvent: 200 mM ammonium acetate, pH 6.7.

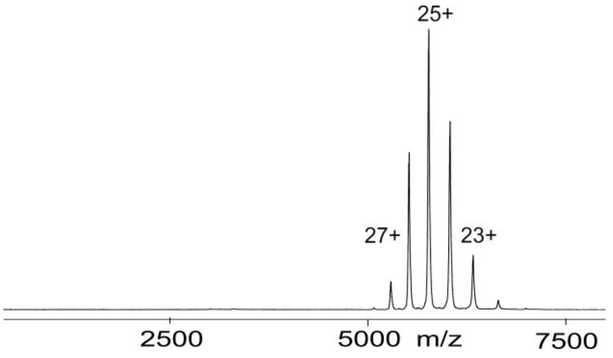

Figure S3: NanoESI mass spectrum of the monoclonal anti-TNFα antibody. Charge states of ion signals are given. For m/z values see Table S1. Protein concentration: 0.2 µg/µl. Solvent: 200 mM ammonium acetate, pH 6.7.

2) hcTnT antigen

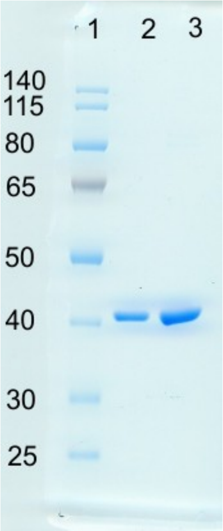

Figure S4: SDS PAGE analysis of hcTnT. Lane 1: Molecular mass marker (PageRuler Prestained Protein Ladder). Apparent molecular masses are shown at the left (in kD). Lane 2: hcTnT (1 µg). Lane 3: hcTnT (2 µg). Proteins were stained with colloidal Coomassie Brilliant Blue.

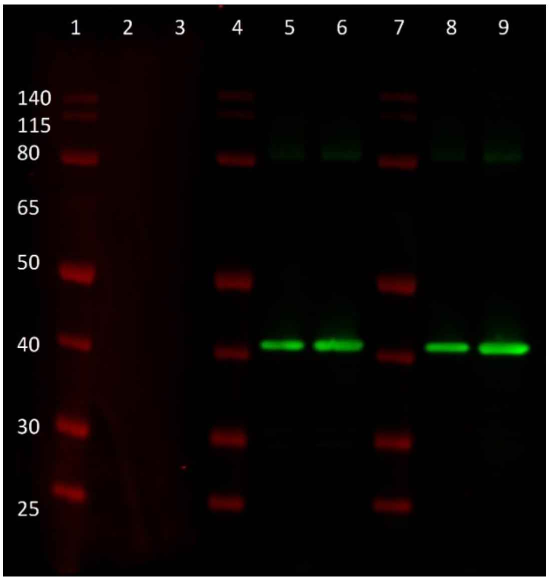

Figure S5: Western blot analysis of hcTnT. Lanes 1, 4, and 7: Molecular mass marker (PageRuler Prestained Protein Ladder). Apparent molecular masses are shown at the left (in kD). Lanes 2, 5 und 8: 0.25 µg hcTnT. Lanes 3, 6, and 9: 0.5 µg hcTnT. Lanes 1, 2, and 3: no primary antibody. Lanes 4, 5, and 6: M11.7 as primary antibody. Lanes 7, 8, and 9: M7 as primary antibody. All lanes: IRDye 800CW-labeled anti-mouse antibody from goat as secondary antibody.

3) Epitope peptides

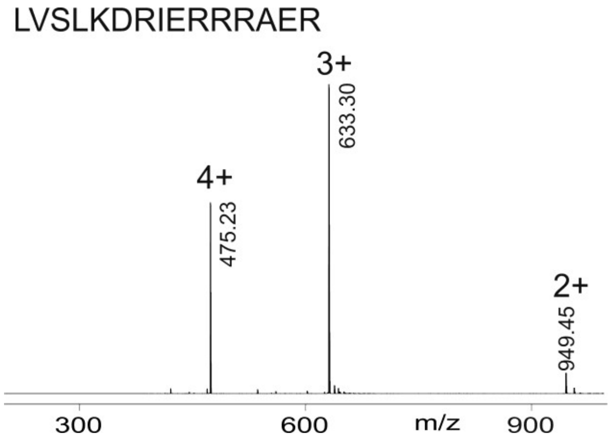

Figure S6: Nano-ESI mass spectrum of peptide P11. The m/z values of the isotopically resolved multiply protonated peptide ion signals and charge states are given. The amino acid sequence is shown in single letter code. Peptide concentration: 0.1 µg/µl. Solvent: 2% acetic acid, 10% methanol (9:1, v/v).

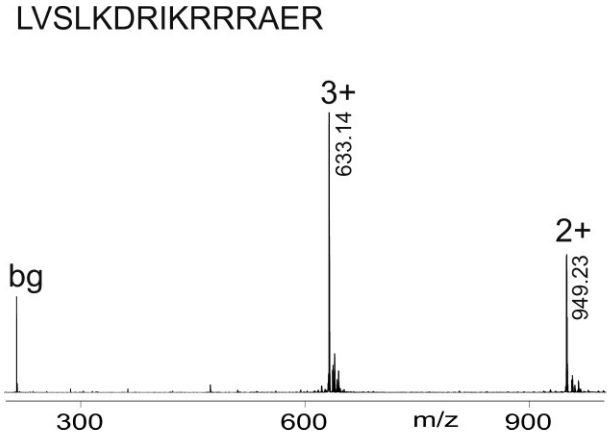

Figure S7: Nano-ESI mass spectrum of peptide P12. The m/z values of the isotopically resolved multiply protonated peptide ion signals and charge states are given. The amino acid sequence is shown in single letter code. Peptide concentration: 0.1 µg/µl. Solvent: 2% acetic acid, 10% methanol (9:1, v/v).

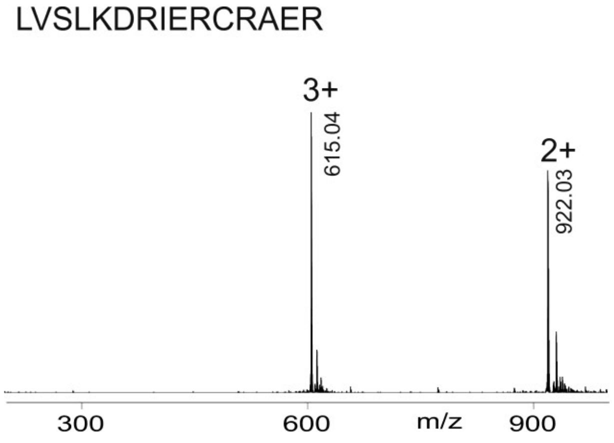

Figure S8. Nano-ESI mass spectrum of peptide P13. The m/z values of the isotopically resolved multiply protonated peptide ion signals and charge states are given. The amino acid sequence is shown in single letter code. Peptide concentration: 0.1 µg/µl. Solvent: 2% acetic acid, 10% methanol (9:1, v/v).

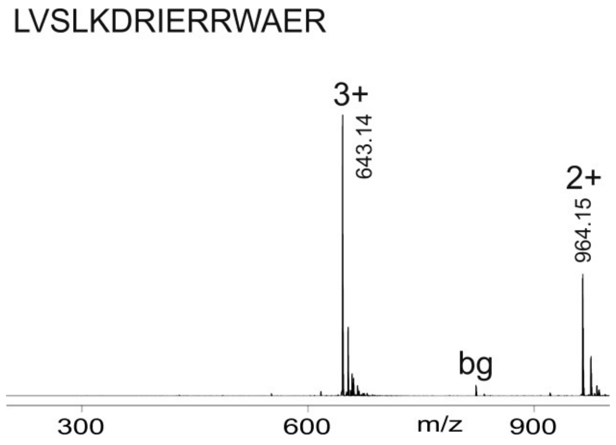

Figure S9. Nano-ESI mass spectrum of peptide P14. The m/z values of the isotopically resolved multiply protonated peptide ion signals and charge states are given. The amino acid sequence is shown in single letter code. bg: background ion signals. Peptide concentration: 0.1 µg/µl. Solvent: 2% acetic acid, 10% methanol (9:1, v/v).

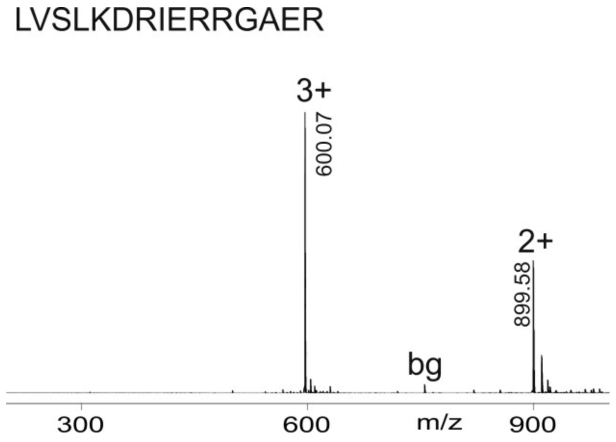

Figure S10. Nano-ESI mass spectrum of peptide P15. The m/z values of the isotopically resolved multiply protonated peptide ion signals and charge states are given. The amino acid sequence is shown in single letter code. bg: background ion signals. Peptide concentration: 0.1 µg/µl. Solvent: 2% acetic acid, 10% methanol (9:1, v/v).

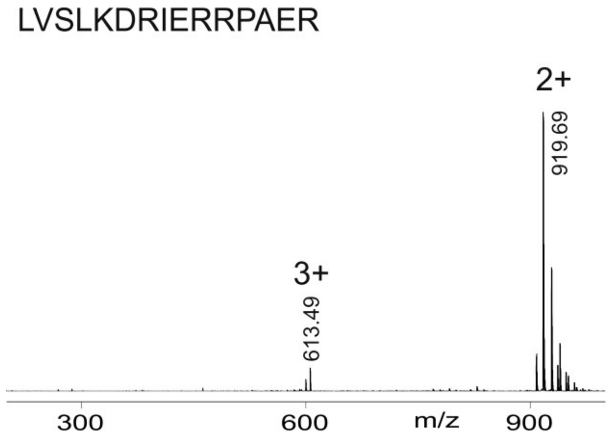

Figure S11. Nano-ESI mass spectrum of peptide P16. The m/z values of the isotopically resolved multiply protonated peptide ion signals and charge states are given. The amino acid sequence is shown in single letter code. Peptide concentration: 0.1 µg/µl. Solvent: 2% acetic acid, 10% methanol (9:1, v/v).

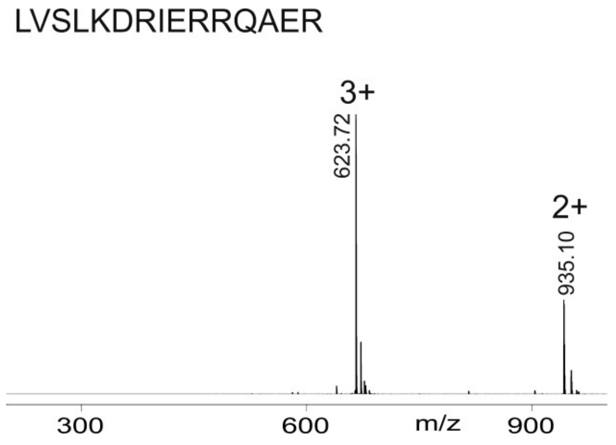

Figure S12. Nano-ESI mass spectrum of peptide P17. The m/z values of the isotopically resolved multiply protonated peptide ion signals and charge states are given. The amino acid sequence is shown in single letter code. Peptide concentration: 0.1 µg/µl. Solvent: 2% acetic acid, 10% methanol (9:1, v/v).

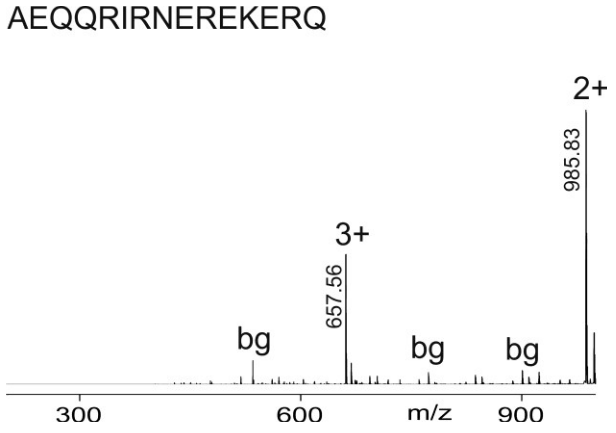

Figure S13. Nano-ESI mass spectrum of peptide P21. The m/z values of the isotopically resolved multiply protonated peptide ion signals and charge states are given. The amino acid sequence is shown in single letter code. bg: background ion signals. Peptide concentration: 0.1 µg/µl. Solvent: 2% acetic acid, 10% methanol (9:1, v/v).

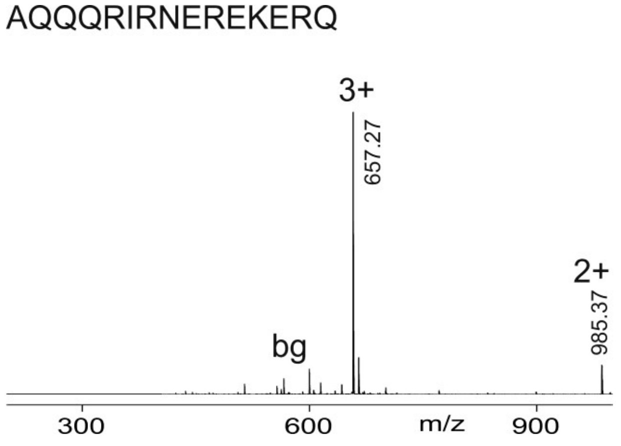

Figure S14. Nano-ESI mass spectrum of peptide P22. The m/z values of the isotopically resolved multiply protonated peptide ion signals and charge states are given. The amino acid sequence is shown in single letter code. bg: background ion signals. Peptide concentration: 0.1 µg/µl. Solvent: 2% acetic acid, 10% methanol (9:1, v/v).

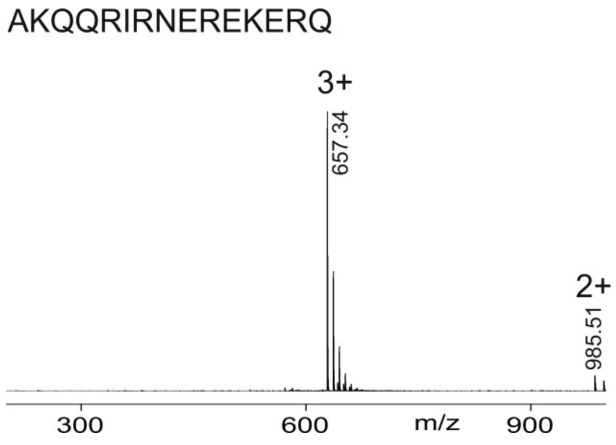

Figure S15. Nano-ESI mass spectrum of peptide P23. The m/z values of the isotopically resolved multiply protonated peptide ion signals and charge states are given. The amino acid sequence is shown in single letter code. Peptide concentration: 0.1 µg/µl. Solvent: 2% acetic acid, 10% methanol (9:1, v/v).

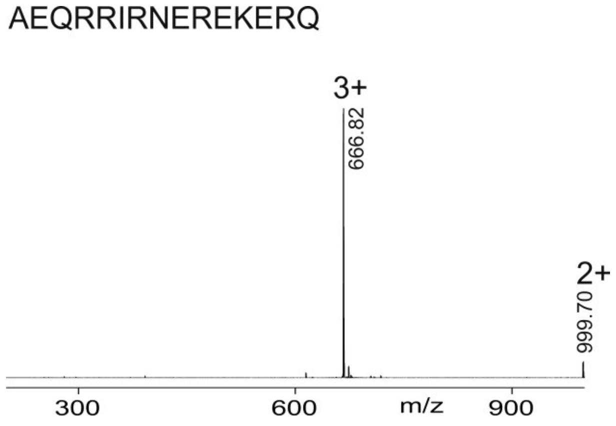

Figure S16. Nano-ESI mass spectrum of peptide P24. The m/z values of the isotopically resolved multiply protonated peptide ion signals and charge states are given. The amino acid sequence is shown in single letter code. Peptide concentration: 0.1 µg/µl. Solvent: 2% acetic acid, 10% methanol (9:1, v/v).

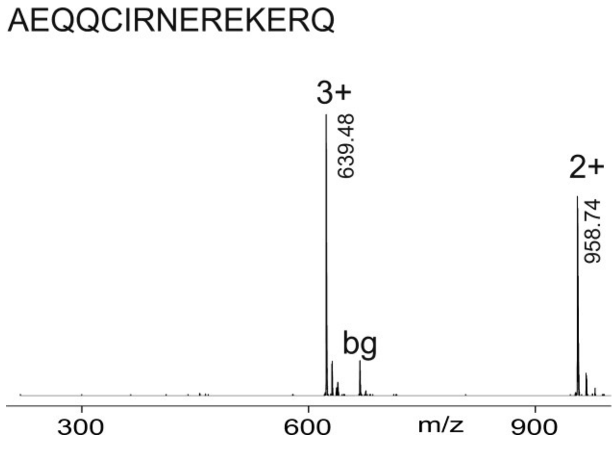

Figure S17. Nano-ESI mass spectrum of peptide P25. The m/z values of the isotopically resolved multiply protonated peptide ion signals and charge states are given. The amino acid sequence is shown in single letter code. bg: background ion signals. Peptide concentration: 0.1 µg/µl. Solvent: 2% acetic acid, 10% methanol (9:1,

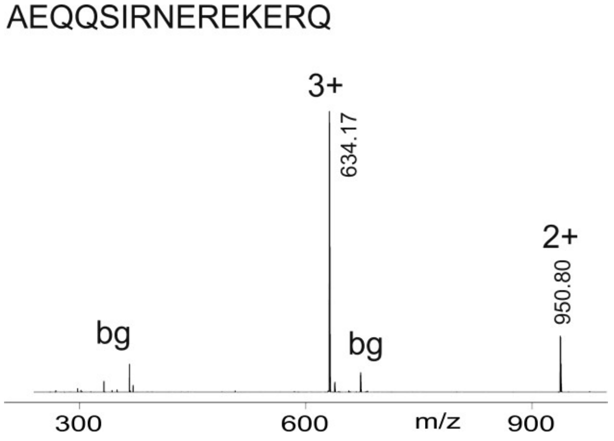

Figure S18. Nano-ESI mass spectrum of peptide P26. The m/z values of the isotopically resolved multiply protonated peptide ion signals and charge states are given. The amino acid sequence is shown in single letter code. bg: background ion signals. Peptide concentration: 0.1 µg/µl. Solvent: 2% acetic acid, 10% methanol (9:1, v/v).

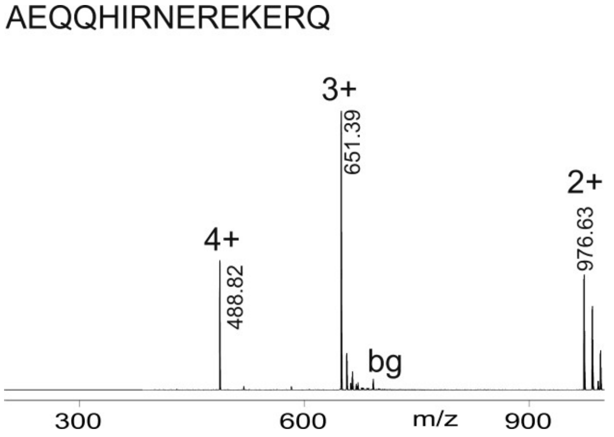

Figure S19. Nano-ESI mass spectrum of peptide P27. The m/z values of the isotopically resolved multiply protonated peptide ion signals and charge states are given. The amino acid sequence is shown in single letter code. bg: background ion signals. Peptide concentration: 0.1 µg/µl. Solvent: 2% acetic acid, 10% methanol (9:1, v/v).

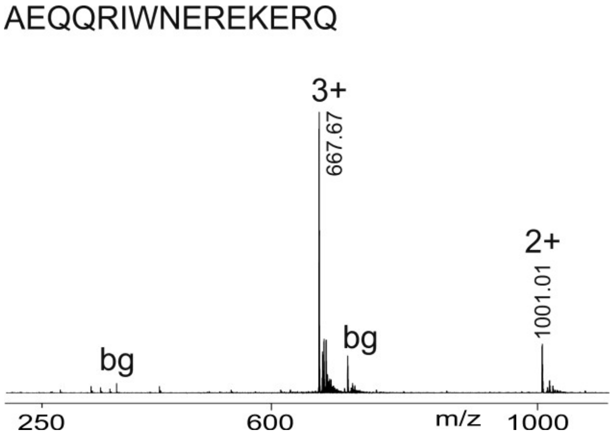

Figure S20. Nano-ESI mass spectrum of peptide P28. The m/z values of the isotopically resolved multiply protonated peptide ion signals and charge states are given. The amino acid sequence is shown in single letter code. bg: background ion signals. Peptide concentration: 0.1 µg/µl. Solvent: 2% acetic acid, 10% methanol (9:1,

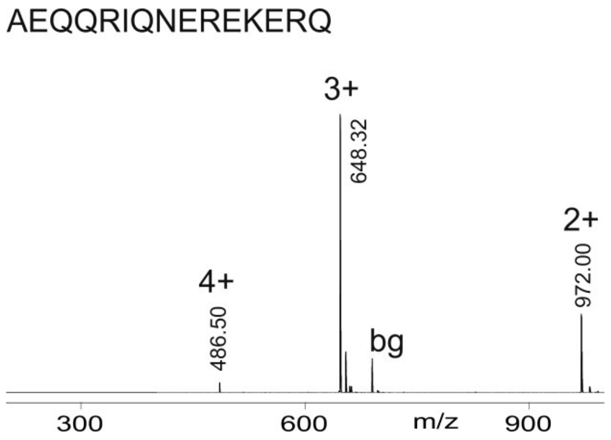

Figure S21. Nano-ESI mass spectrum of peptide P29. The m/z values of the isotopically resolved multiply protonated peptide ion signals and charge states are given. The amino acid sequence is shown in single letter code. bg: background ion signals. Peptide concentration: 0.1 µg/µl. Solvent: 2% acetic acid, 10% methanol (9:1, v/v).

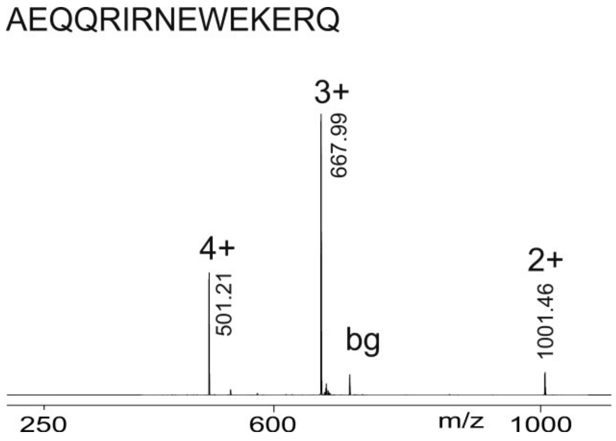

Figure S22. Nano-ESI mass spectrum of peptide P30. The m/z values of the isotopically resolved multiply protonated peptide ion signals and charge states are given. The amino acid sequence is shown in single letter code. bg: background ion signals. Peptide concentration: 0.1 µg/µl. Solvent: 2% acetic acid, 10% methanol (9:1, v/v).

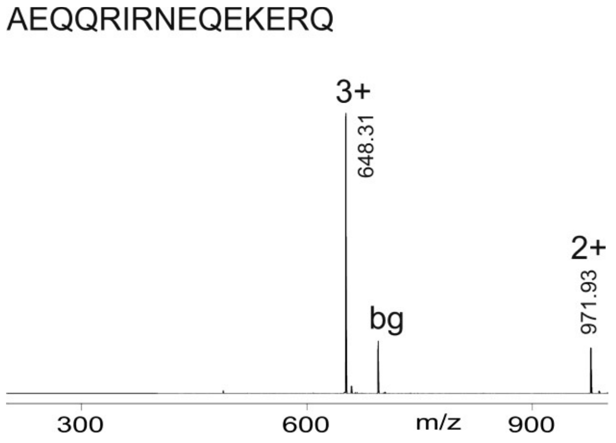

Figure S23. Nano-ESI mass spectrum of peptide P31. The m/z values of the isotopically resolved multiply protonated peptide ion signals and charge states are given. The amino acid sequence is shown in single letter code. bg: background ion signals. Peptide concentration: 0.1 µg/µl. Solvent: 2% acetic acid, 10% methanol (9:1, v/v).

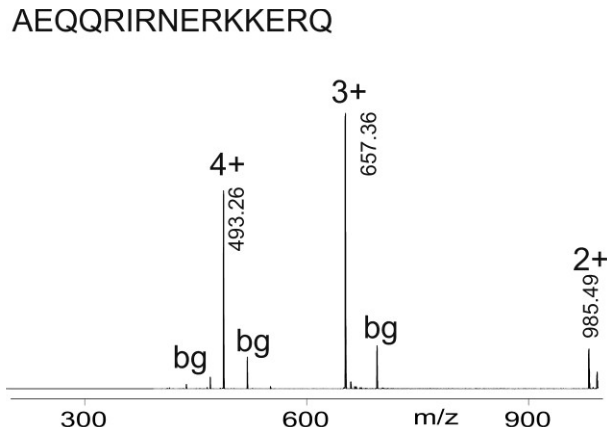

Figure S24. Nano-ESI mass spectrum of peptide P32. The m/z values of the isotopically resolved multiply protonated peptide ion signals and charge states are given. The amino acid sequence is shown in single letter code. bg: background ion signals. Peptide concentration: 0.1 µg/µl. Solvent: 2% acetic acid, 10% methanol (9:1,

4) ITEM-FOUR mass spectrometry

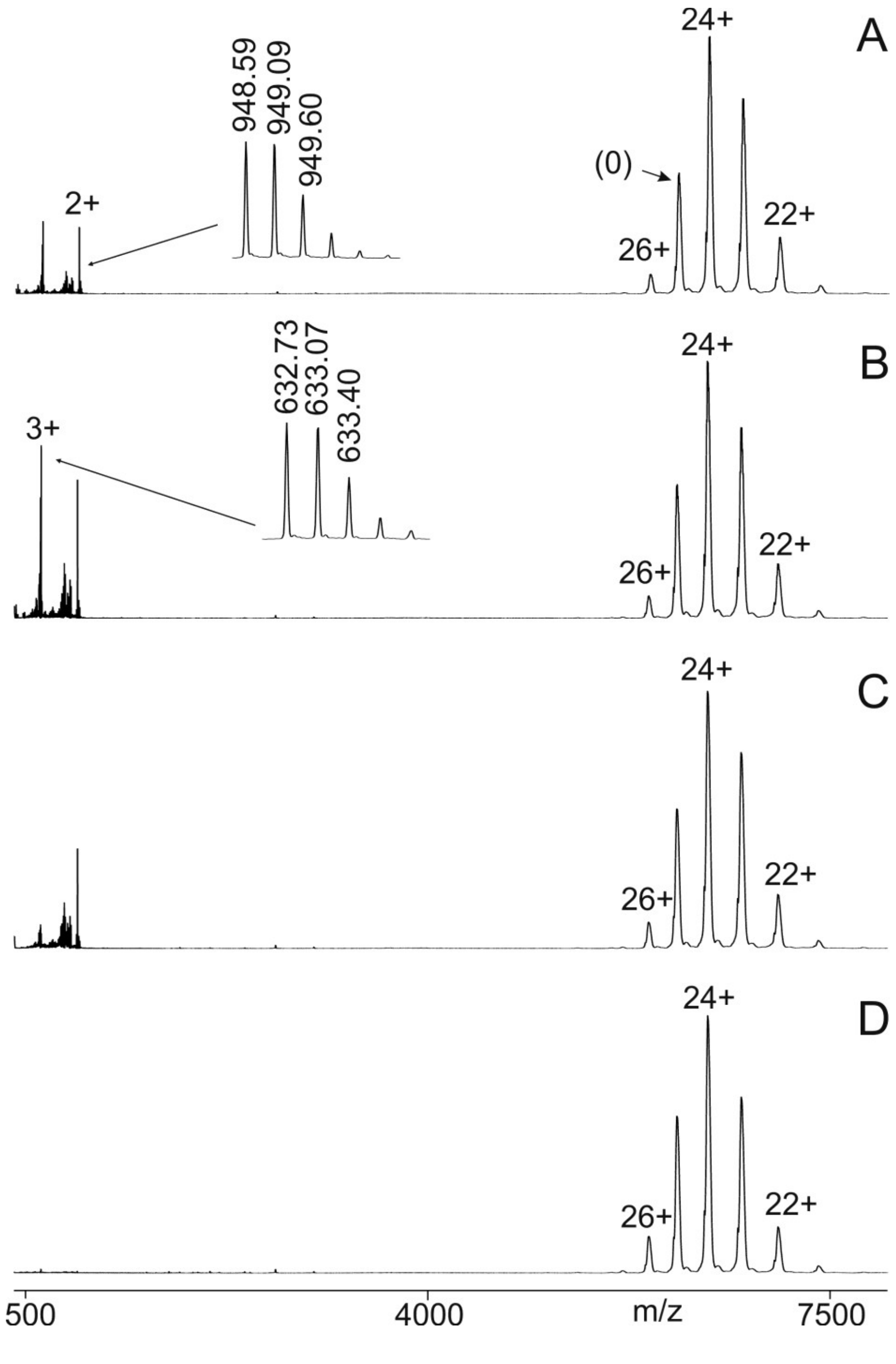

Figure S25: Nano-ESI mass spectra of peptide P12 (LVSLKDRI**K**RRRAER) – M7 antibody mixtures with increasing collision cell voltage differences (ΔCV). A: 2 V, B: 14 V, C: 30 V, D: 52 V. Charge states are given for the ion signals of the antibody (right ion series). Charge states for peptide ion signals are given on the left. The inlets in B and C show zoom views of the isotopically resolved peptide ion signals and their m/z values. The quadrupole was set to block transmission of ions < m/z 3850. Molar ratio of peptide to antibody was 2.1 : 1. Solvent: 200 mM ammonium acetate, pH 6.7.

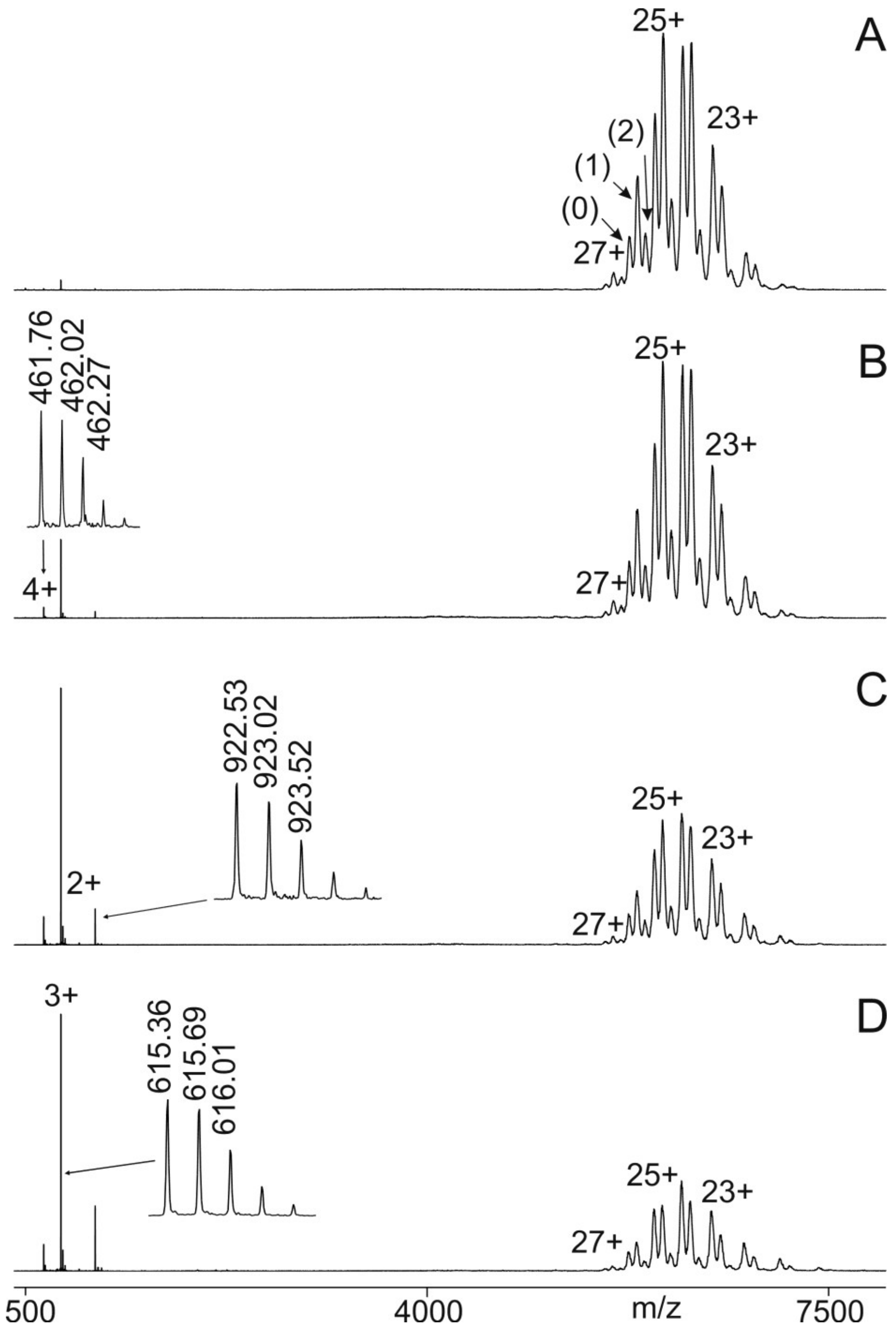

Figure S26: Nano-ESI mass spectra of peptide P13 (LVSLKDRIERCRAER) – M7 antibody mixtures with increasing collision cell voltage differences (ΔCV). A: 2 V, B: 14 V, C: 30 V, D: 52 V. Charge states are given for the ion signals (right ion series) of the antibody (0) and the immune complexes (antibody plus one peptide (1) and antibody plus two peptides (2)). Charge states for peptide ion signals are given on the left. The inlets in B, C, and D show zoom views of the isotopically resolved peptide ion signals and their m/z values. The quadrupole was set to block transmission of ions < m/z 3850. Molar ratio of peptide to antibody was 2.1 : 1. Solvent: 200 mM ammonium acetate, pH 6.7.

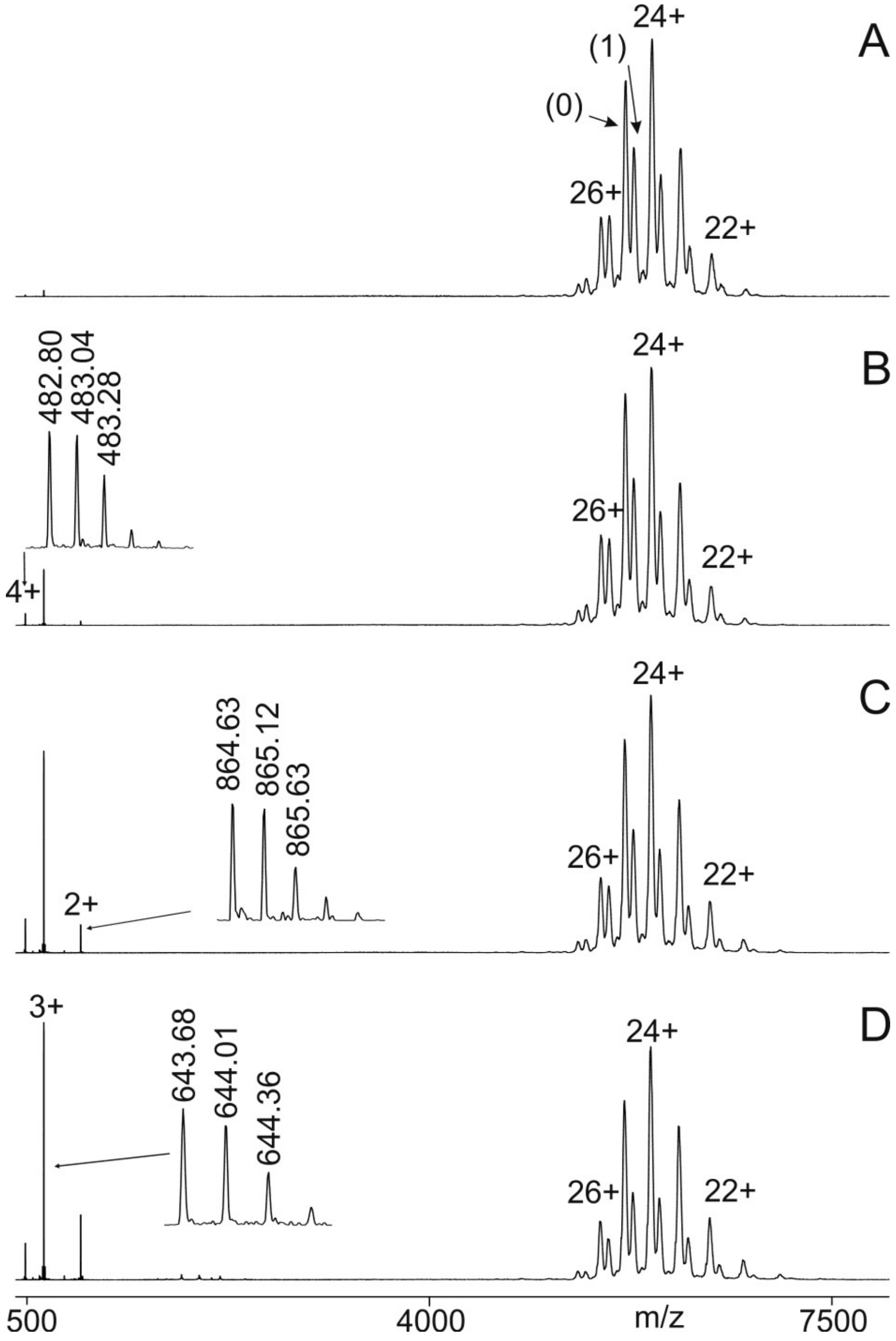

Figure S27: Nano-ESI mass spectra of peptide P14 (LVSLKDRIERR**W**AER) – M7 antibody mixtures with increasing collision cell voltage differences (ΔCV). A: 2 V, B: 14 V, C: 30 V, D: 52 V. Charge states are given for the ion signals (right ion series) of the antibody (0) and the immune complexes (antibody plus one peptide (1) and antibody plus two peptides (2)). Charge states for peptide ion signals are given on the left. The inlets in B, C, and D show zoom views of the isotopically resolved peptide ion signals and their m/z values. The quadrupole was set to block transmission of ions < m/z 3850. Molar ratio of peptide to antibody was 2.1 : 1. Solvent: 200 mM ammonium acetate, pH 6.7.

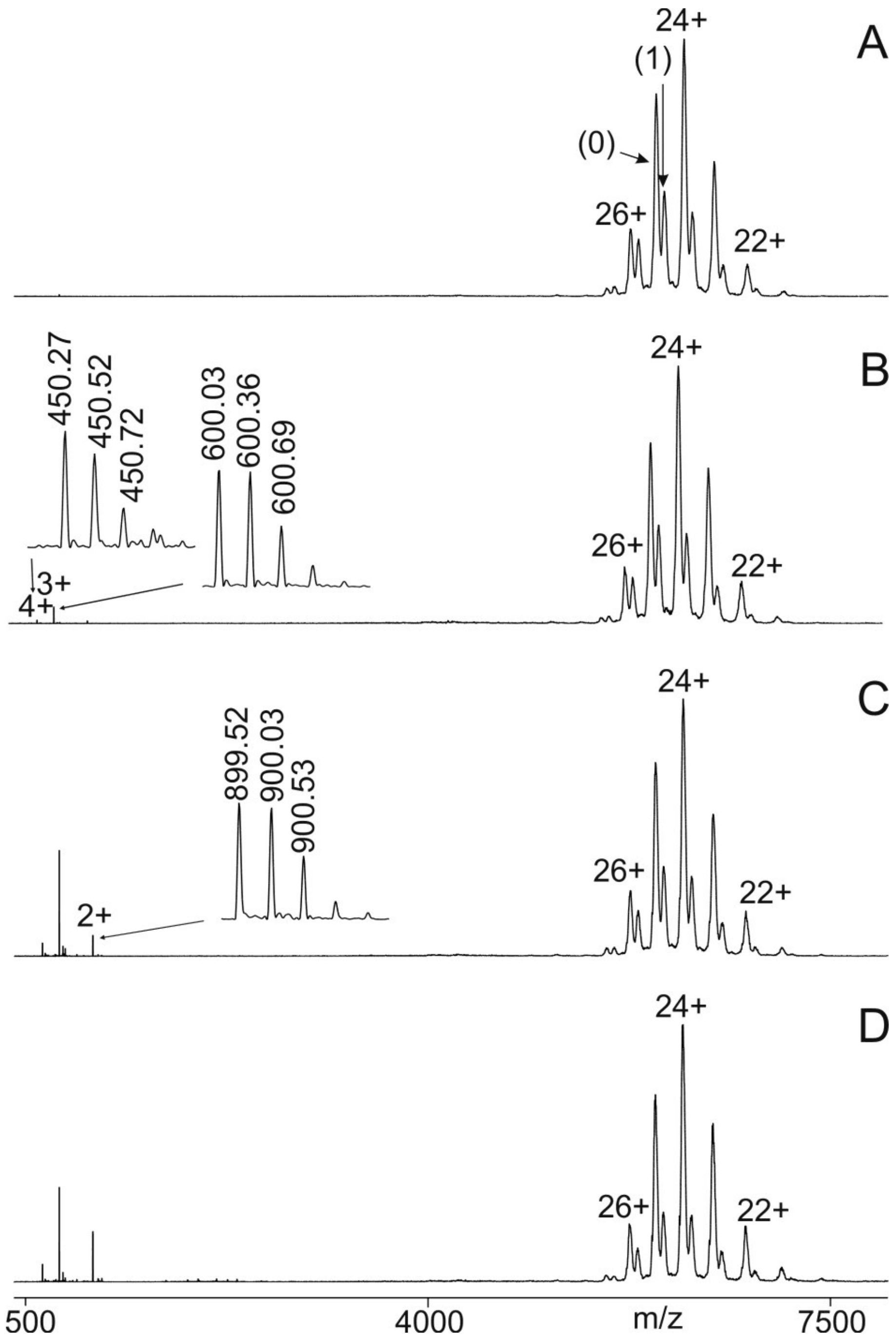

Figure S28: Nano-ESI mass spectra of peptide P15 (LVSLKDRIERRGAER) – M7 antibody mixtures with increasing collision cell voltage differences (ΔCV). A: 2 V, B: 14 V, C: 30 V, D: 52 V. Charge states are given for the ion signals (right ion series) of the antibody (0) and the immune complexes (antibody plus one peptide (1)). Charge states for peptide ion signals are given on the left. The inlets in B and C show zoom views of the isotopically resolved peptide ion signals and their m/z values. The quadrupole was set to block transmission of ions < m/z 3850. Molar ratio of peptide to antibody was 2.1 : 1. Solvent: 200 mM ammonium acetate, pH 6.7.

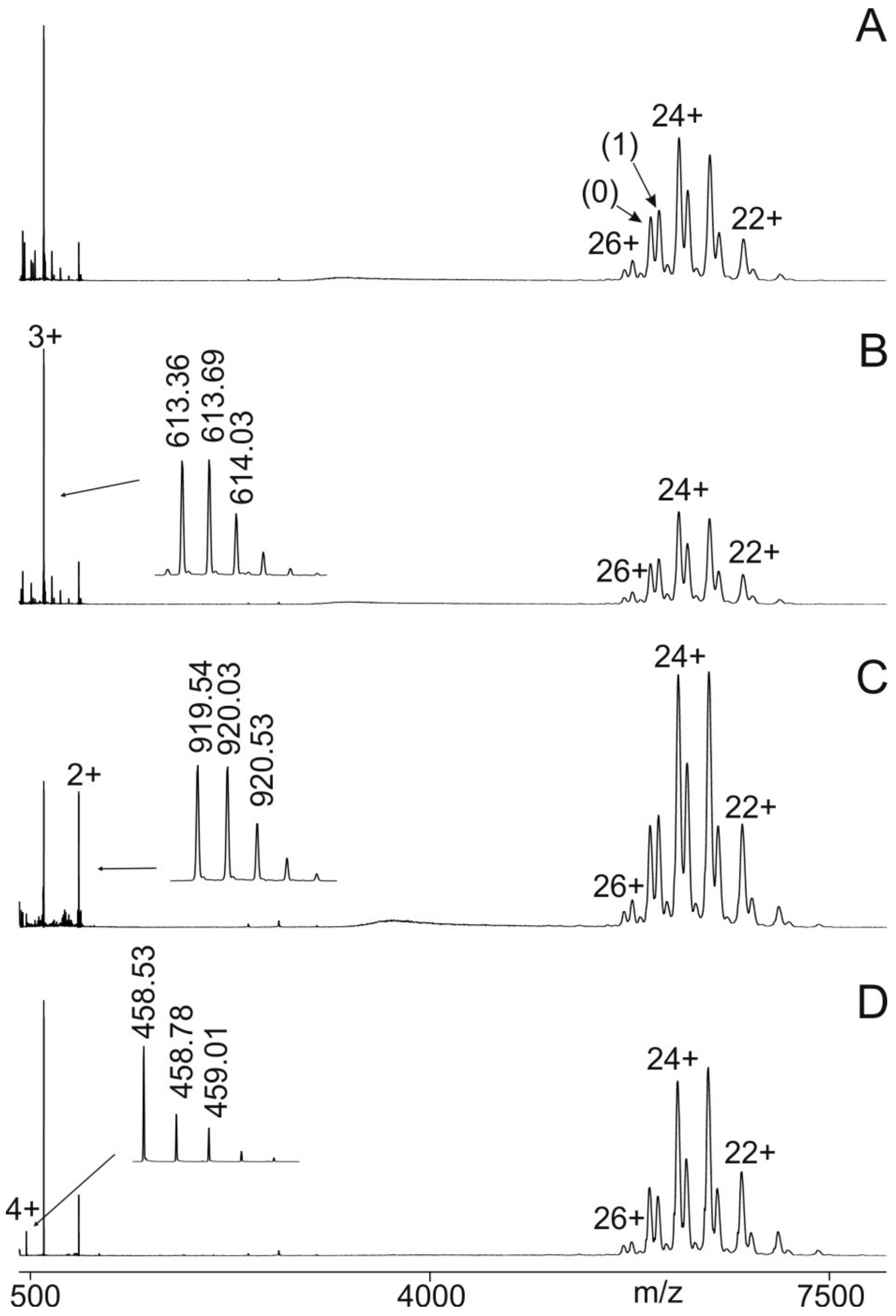

Figure S29: Nano-ESI mass spectra of peptide P16 (LVSLKDRIERRPAER) – M7 antibody mixtures with increasing collision cell voltage differences (ΔCV). A: 2 V, B: 14 V, C: 30 V, D: 52 V. Charge states are given for the ion signals (right ion series) of the antibody (0) and the immune complexes (antibody plus one peptide (1)). Charge states for peptide ion signals are given on the left. The inlets in B, C, and D show zoom views of the isotopically resolved peptide ion signals and their m/z values. The quadrupole was set to block transmission of ions < m/z 3850. Molar ratio of peptide to antibody was 2.1 : 1. Solvent: 200 mM ammonium acetate, pH 6.7.

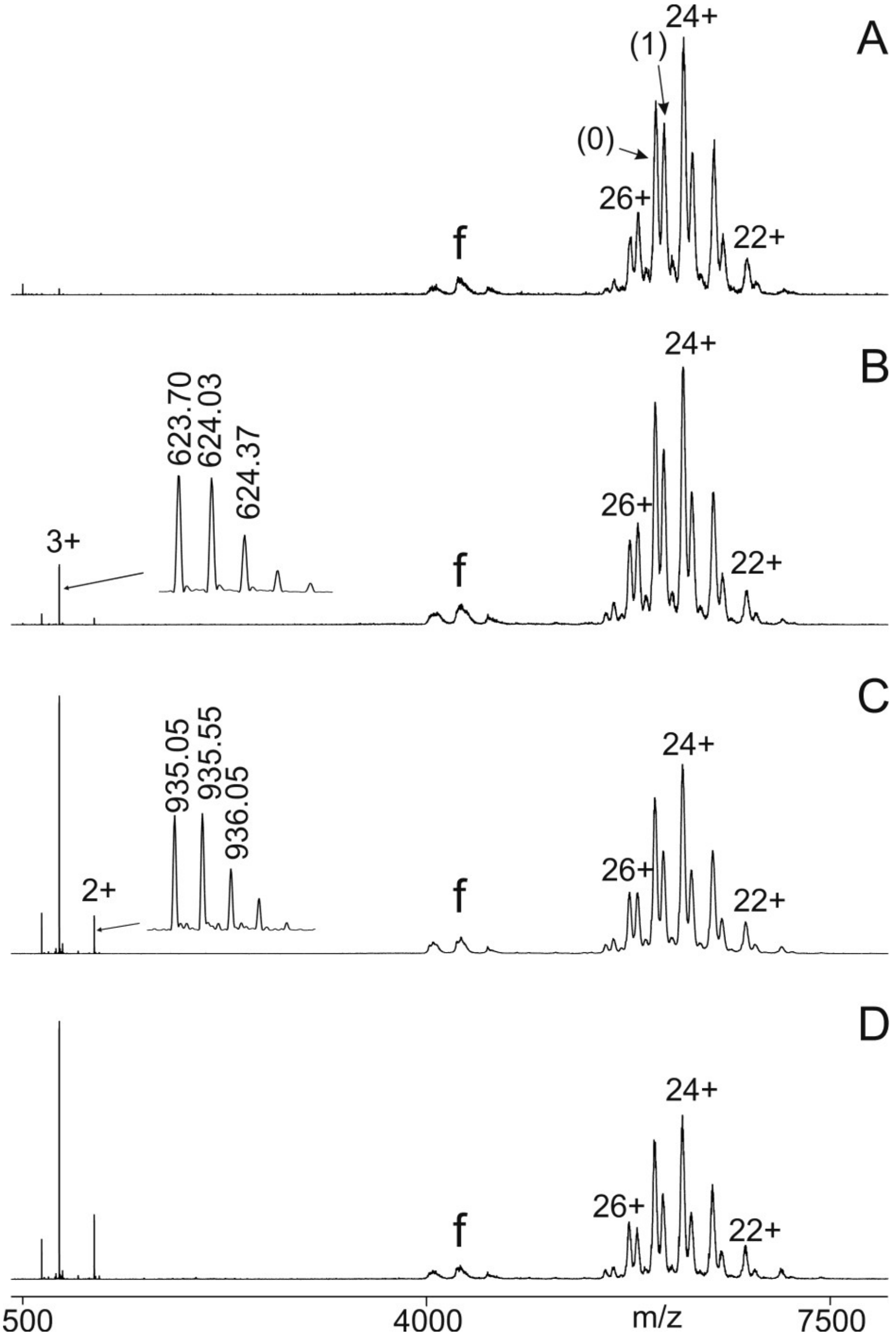

Figure S30: Nano-ESI mass spectra of peptide P17 (LVSLKDRIERR**Q**AER) – M7 antibody mixtures with increasing collision cell voltage differences (ΔCV). A: 2 V, B: 14 V, C: 30 V, D: 52 V. Charge states are given for the ion signals (right ion series) of the antibody (0) and the immune complexes (antibody plus one peptide (1)). Charge states for peptide ion signals are given on the left. The inlets in B and C show zoom views of the isotopically resolved peptide ion signals and their m/z values. Multiply charged antibody fragment ions are labeled f. The quadrupole was set to block transmission of ions < m/z 3850. Molar ratio of peptide to antibody was 2.1 : 1. Solvent: 200 mM ammonium acetate, pH 6.7.

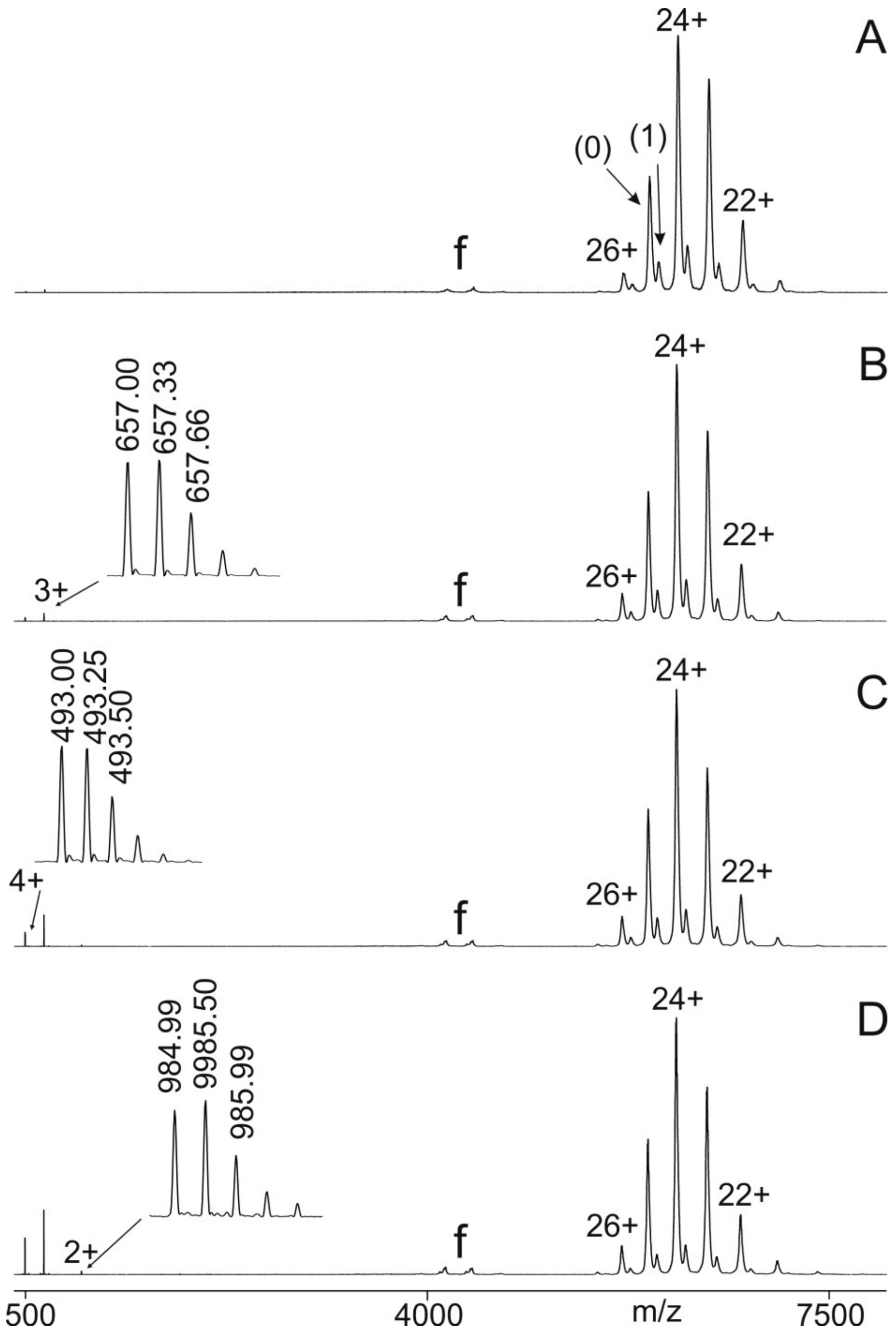

Figure S31: Nano-ESI mass spectra of peptide P22 (AQQQRIRNEREKERQ) – M11.7 antibody mixtures with increasing collision cell voltage differences (ΔCV). A: 2 V, B: 14 V, C: 30 V, D: 52 V. Charge states are given for the ion signals (right ion series) of the antibody (0) and the immune complexes (antibody plus one peptide (1)). Charge states for peptide ion signals are given on the left. The inlets in B, C, and D show zoom views of the isotopically resolved peptide ion signals and their m/z values. Multiply charged antibody fragment ions are labeled f. The quadrupole was set to block transmission of ions < m/z 3850. Molar ratio of peptide to antibody was 13 : 1. Solvent: 200 mM ammonium acetate, pH 6.7.

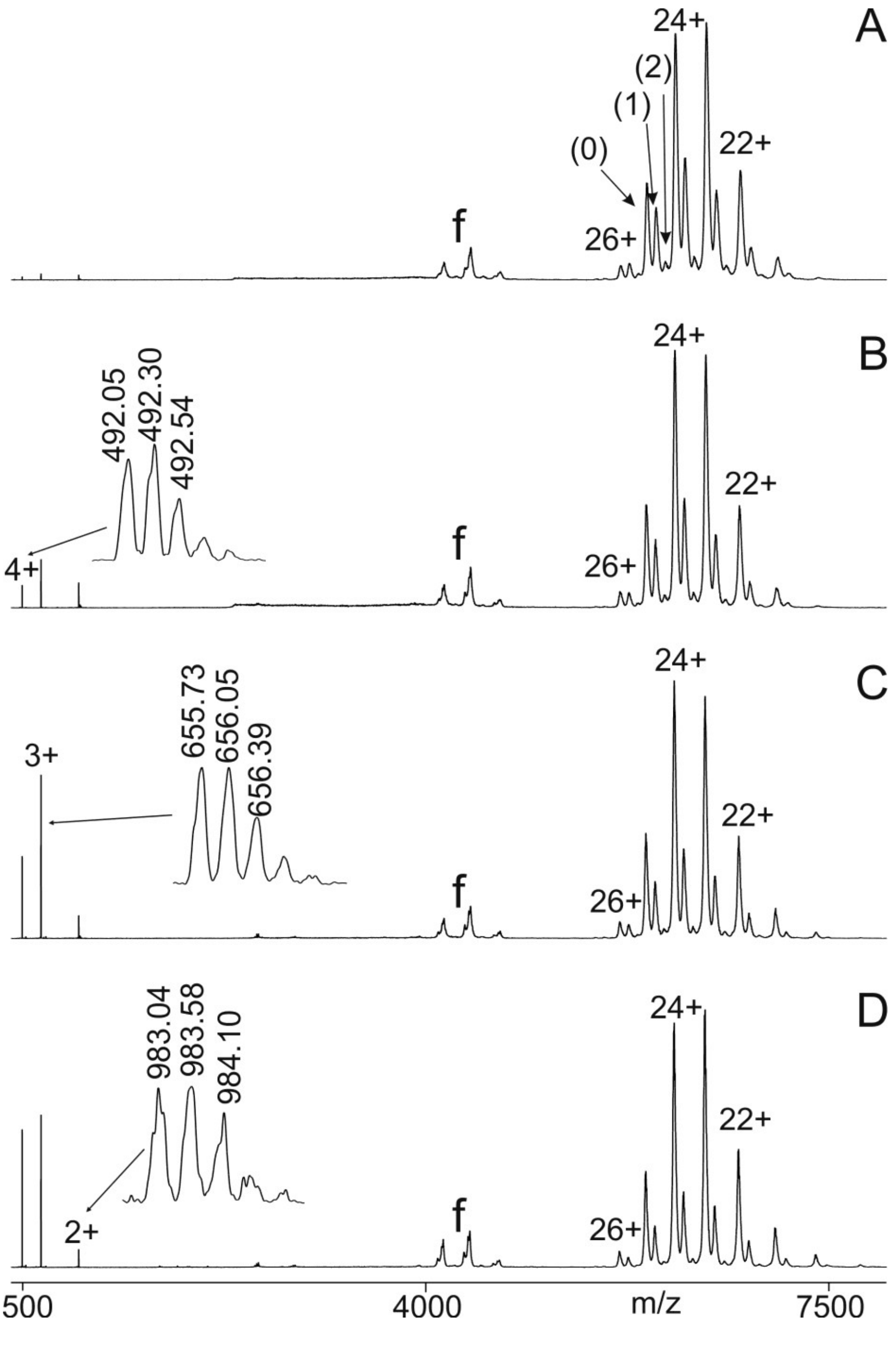

Figure S32: Nano-ESI mass spectra of peptide P23 (AKQQRIRNEREKERQ) – M11.7 antibody mixtures with increasing collision cell voltage differences (ΔCV). A: 2 V, B: 14 V, C: 30 V, D: 52 V. Charge states are given for the ion signals (right ion series) of the antibody (0) and the immune complexes (antibody plus one peptide (1) and antibody plus two peptides (2)). Charge states for peptide ion signals are given on the left. The inlets in B, C, and D show zoom views of the isotopically resolved peptide ion signals and their m/z values. Multiply charged antibody fragment ions are labeled f. The quadrupole was set to block transmission of ions < m/z 3850. Molar ratio of peptide to antibody was 13 : 1. Solvent: 200 mM ammonium acetate, pH 6.7.

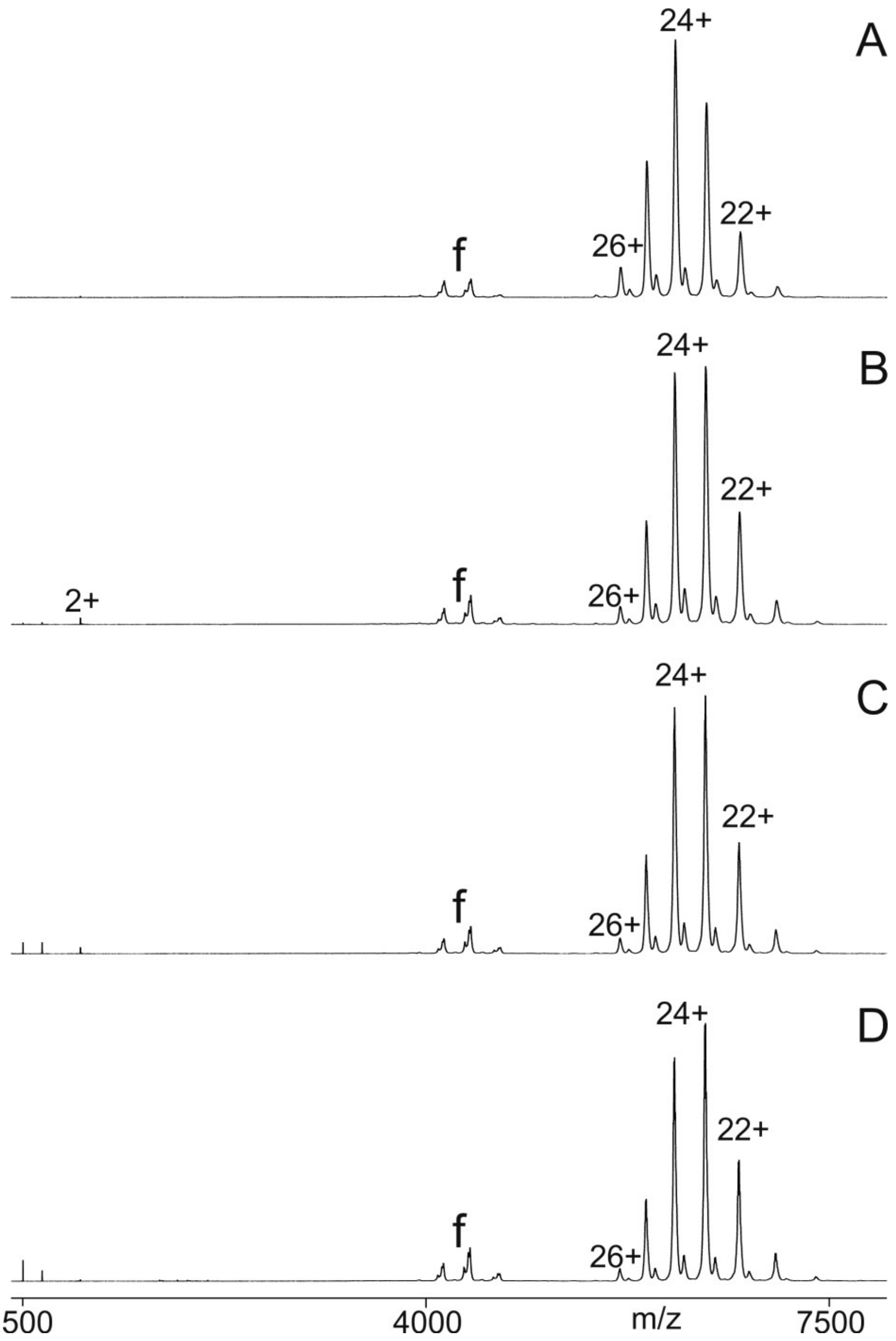

Figure S33: Nano-ESI mass spectra of peptide P24 (AEQ**R**RIRNEREKERQ) – M11.7 antibody mixtures with increasing collision cell voltage differences (ΔCV). A: 2 V, B: 14 V, C: 30 V, D: 52 V. Charge states are given for the ion signals of the antibody (right ion series). Charge states for peptide ion signals are given on the left. Multiply charged antibody fragment ions are labeled f. The quadrupole was set to block transmission of ions < m/z 3850. Molar ratio of peptide to antibody was 13 : 1. Solvent: 200 mM ammonium acetate, pH 6.7.

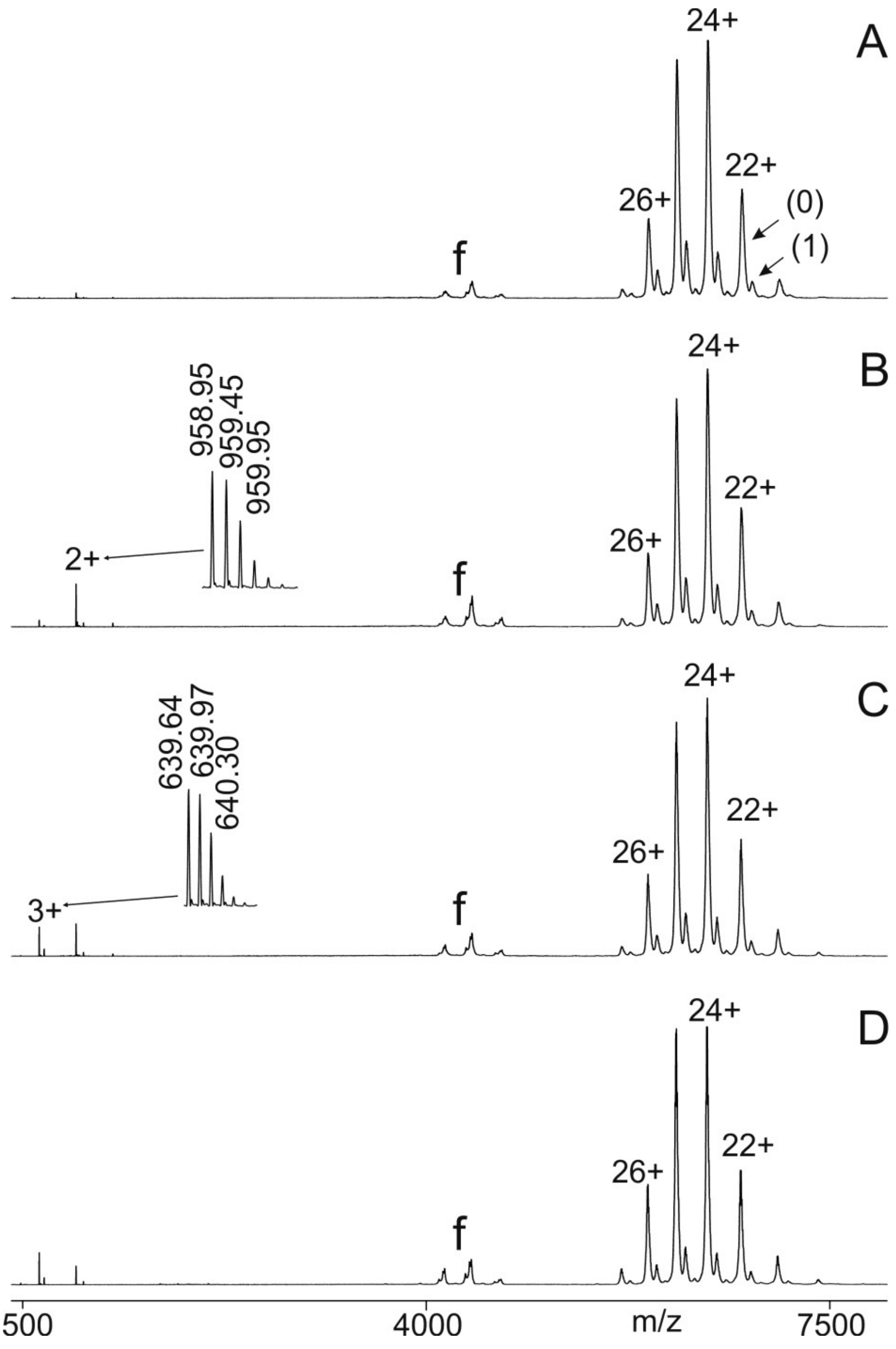

Figure S34: Nano-ESI mass spectra of peptide P25 (AEQQCIRNEREKERQ) – M11.7 antibody mixtures with increasing collision cell voltage differences (∆CV). A: 2 V, B: 14 V, C: 30 V, D: 52 V. Charge states are given for the ion signals (right ion series) of the antibody (0) and the immune complexes (antibody plus one peptide (1)). Charge states for peptide ion signals are given on the left. The inlets in B and C show zoom views of the isotopically resolved peptide ion signals and their m/z values. Multiply charged antibody fragment ions are labeled f. The quadrupole was set to block transmission of ions < m/z 3850. Molar ratio of peptide to antibody was 13 : 1. Solvent: 200 mM ammonium acetate, pH 6.7.

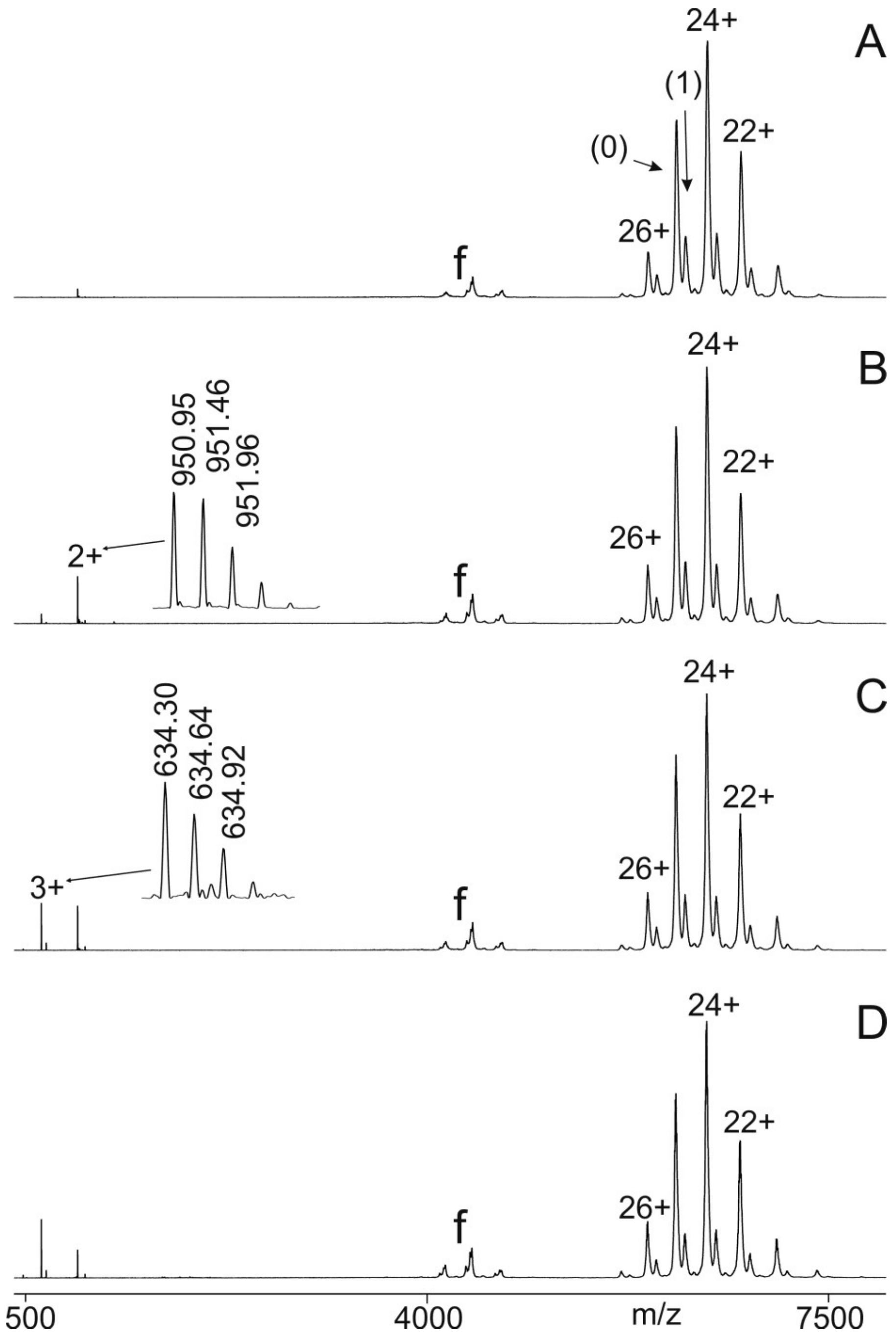

Figure S35: Nano-ESI mass spectra of peptide P26 (AEQQSIRNEREKERQ) – M11.7 antibody mixtures with increasing collision cell voltage differences (ΔCV). A: 2 V, B: 14 V, C: 30 V, D: 52 V. Charge states are given for the ion signals (right ion series) of the antibody (0) and the immune complexes (antibody plus one peptide (1)). Charge states for peptide ion signals are given on the left. The inlets in B and C show zoom views of the isotopically resolved peptide ion signals and their m/z values. Multiply charged antibody fragment ions are labeled f. The quadrupole was set to block transmission of ions < m/z 3850. Molar ratio of peptide to antibody was 13 : 1. Solvent: 200 mM ammonium acetate, pH 6.7.

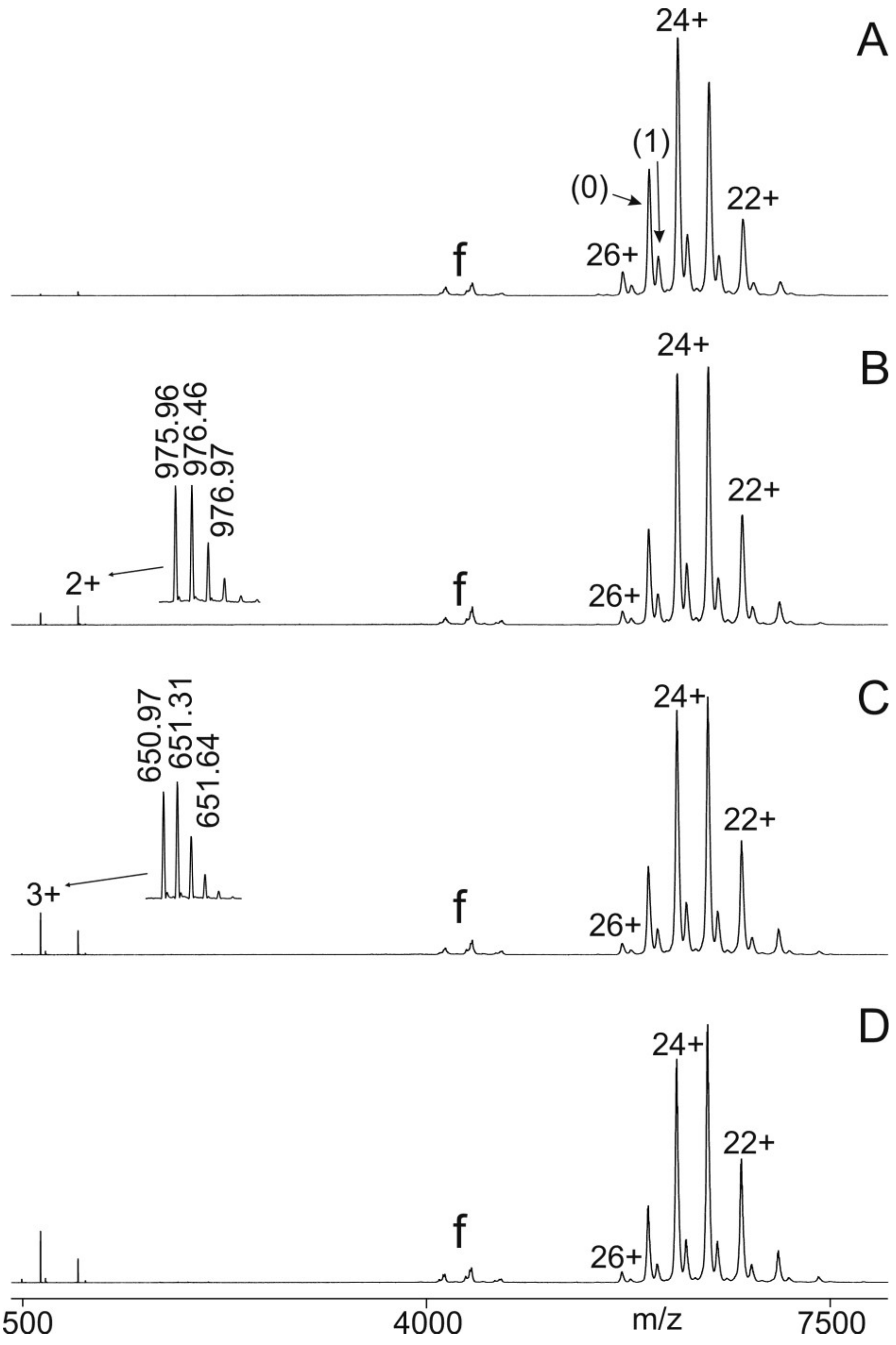

Figure S36: Nano-ESI mass spectra of peptide P27 (AEQQHIRNEREKERQ) – M11.7 antibody mixtures with increasing collision cell voltage differences (ΔCV). A: 2 V, B: 14 V, C: 30 V, D: 52 V. Charge states are given for the ion signals (right ion series) of the antibody (0) and the immune complexes (antibody plus one peptide (1)). Charge states for peptide ion signals are given on the left. The inlets in B and C show zoom views of the isotopically resolved peptide ion signals and their m/z values. Multiply charged antibody fragment ions are labeled f. The quadrupole was set to block transmission of ions < m/z 3850. Molar ratio of peptide to antibody was 13 : 1. Solvent: 200 mM ammonium acetate, pH 6.7.

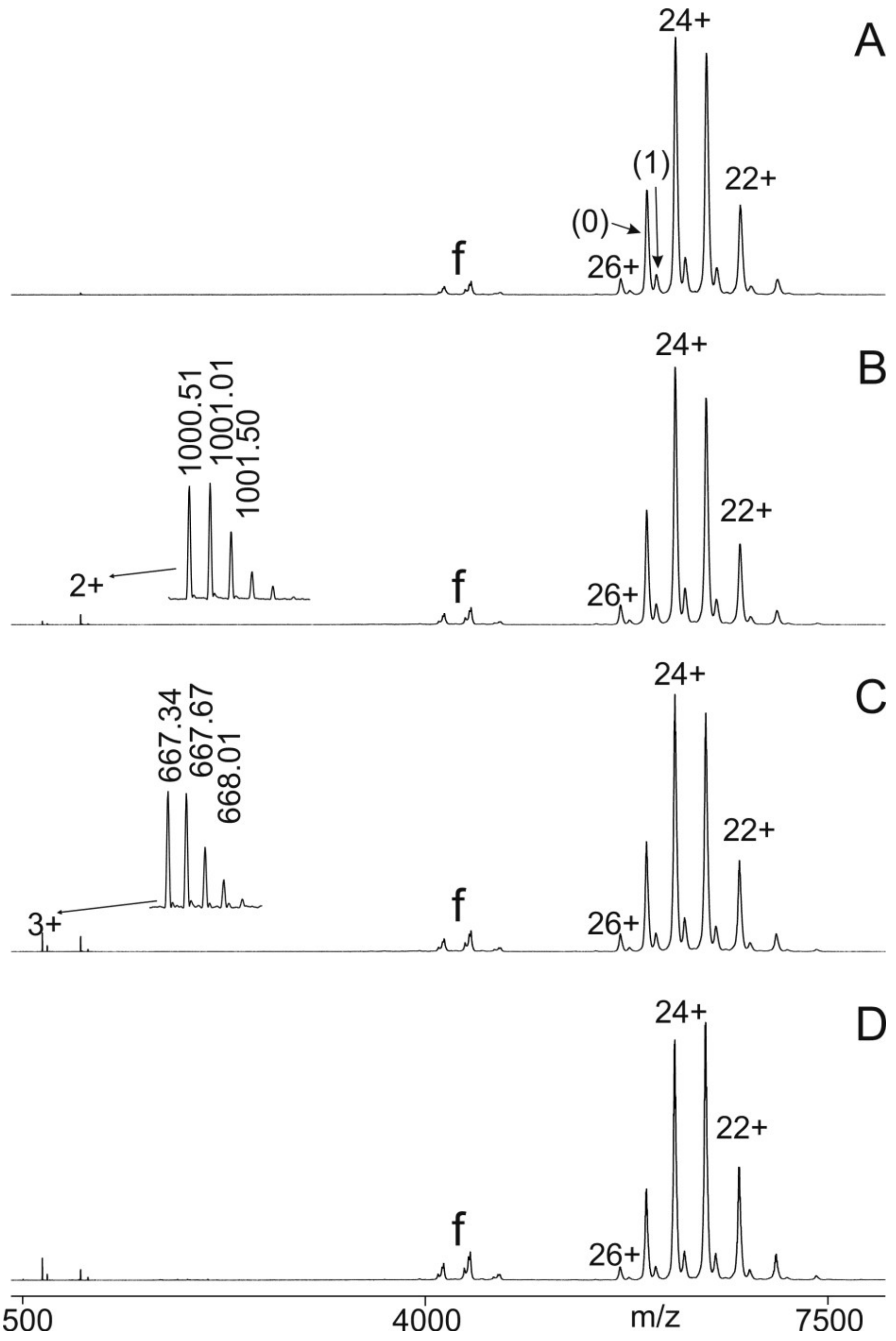

Figure S37: Nano-ESI mass spectra of peptide P28 (AEQQRIWNEREKERQ) – M11.7 antibody mixtures with increasing collision cell voltage differences (ΔCV). A: 2 V, B: 14 V, C: 30 V, D: 52 V. Charge states are given for the ion signals (right ion series) of the antibody (0) and the immune complexes (antibody plus one peptide (1)). Charge states for peptide ion signals are given on the left. The inlets in B and C show zoom views of the isotopically resolved peptide ion signals and their m/z values. Multiply charged antibody fragment ions are labeled f. The quadrupole was set to block transmission of ions < m/z 3850. Molar ratio of peptide to antibody was 13 : 1. Solvent: 200 mM ammonium acetate, pH 6.7.

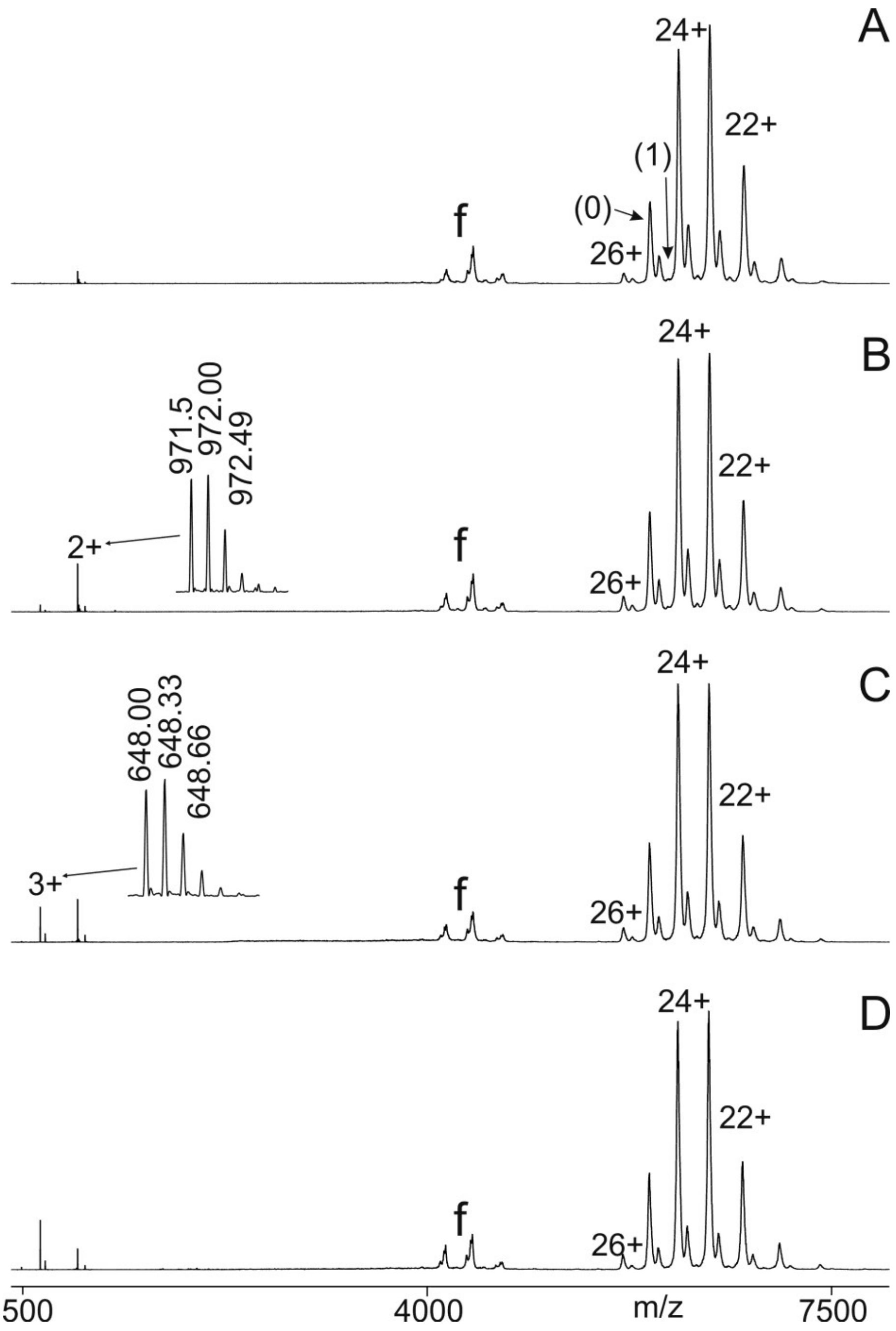

Figure S38: Nano-ESI mass spectra of peptide P29 (AEQQRIQNEREKERQ) – M11.7 antibody mixtures with increasing collision cell voltage differences (ΔCV). A: 2 V, B: 14 V, C: 30 V, D: 52 V. Charge states are given for the ion signals (right ion series) of the antibody (0) and the immune complexes (antibody plus one peptide (1)). Charge states for peptide ion signals are given on the left. The inlets in B and C show zoom views of the isotopically resolved peptide ion signals and their m/z values. Multiply charged antibody fragment ions are labeled f. The quadrupole was set to block transmission of ions < m/z 3850. Molar ratio of peptide to antibody was 13 : 1. Solvent: 200 mM ammonium acetate, pH 6.7.

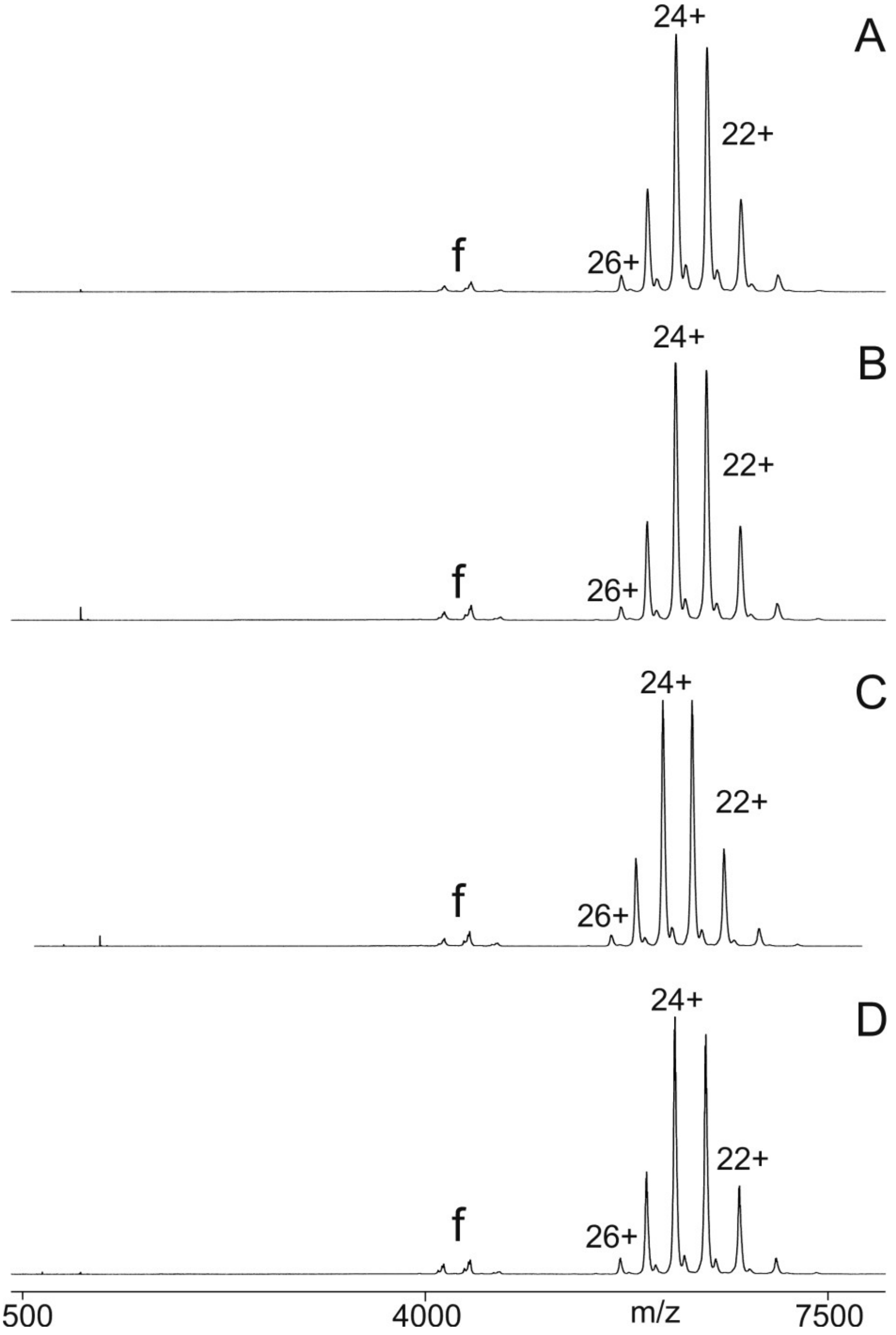

Figure S39: Nano-ESI mass spectra of peptide P30 (AEQQRIRNEWEKERQ) – M11.7 antibody mixtures with increasing collision cell voltage differences (ΔCV). A: 2 V, B: 14 V, C: 30 V, D: 52 V. Charge states are given for the ion signals of the antibody (right ion series). Charge states for peptide ion signals are given on the left. Multiply charged antibody fragment ions are labeled f. The quadrupole was set to block transmission of ions < m/z 3850. Molar ratio of peptide to antibody was 13 : 1. Solvent: 200 mM ammonium acetate, pH 6.7.

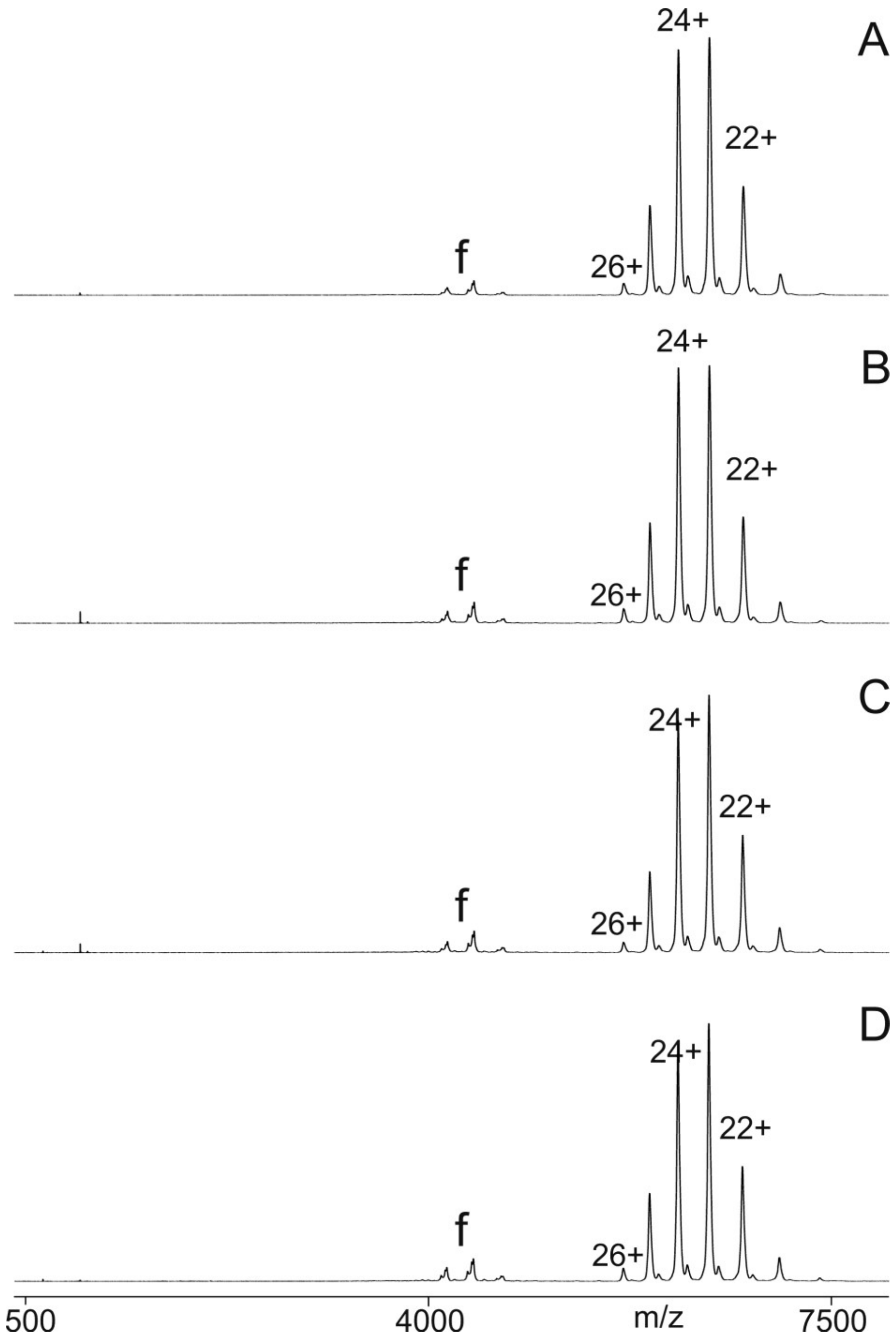

Figure S40: Nano-ESI mass spectra of peptide P31 (AEQQRIRNEQEKERQ) – M11.7 antibody mixtures with increasing collision cell voltage differences (ΔCV). A: 2 V, B: 14 V, C: 30 V, D: 52 V. Charge states are given for the ion signals of the antibody (right ion series). Charge states for peptide ion signals are given on the left. Multiply charged antibody fragment ions are labeled f. The quadrupole was set to block transmission of ions < m/z 3850. Molar ratio of peptide to antibody was 13 : 1. Solvent: 200 mM ammonium acetate, pH 6.7.

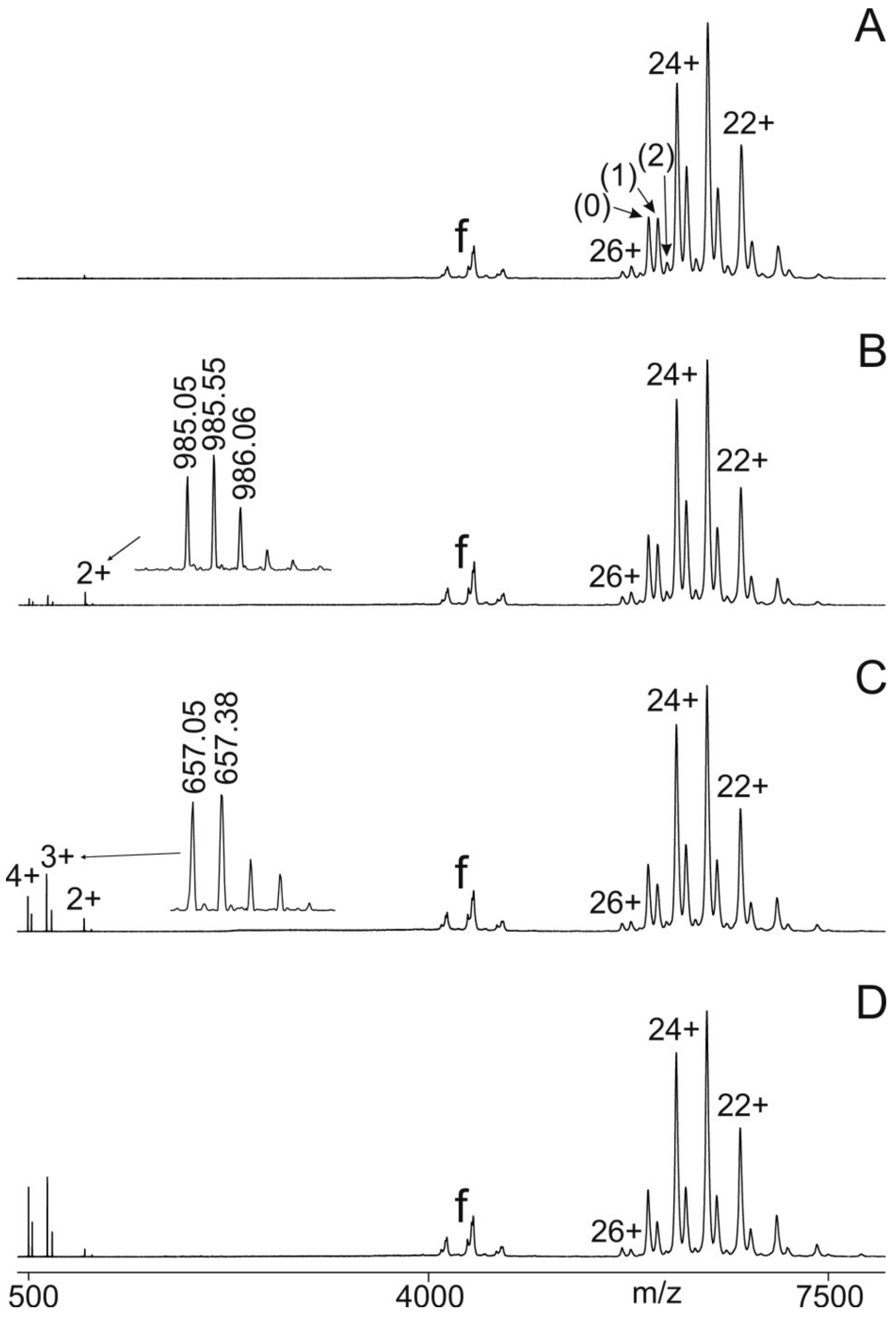

Figure S41: Nano-ESI mass spectra of peptide P32 (AEQQRIRNER**K**KERQ) – M11.7 antibody mixtures with increasing collision cell voltage differences (ΔCV). A: 2 V, B: 14 V, C: 30 V, D: 52 V. Charge states are given for the ion signals (right ion series) of the antibody (0) and the immune complexes (antibody plus one peptide (1) and antibody plus two peptides (2)). Charge states for peptide ion signals are given on the left. The inlets in B and C show zoom views of the isotopically resolved peptide ion signals and their m/z values. Multiply charged antibody fragment ions are labeled f. The quadrupole was set to block transmission of ions < m/z 3850. Molar ratio of peptide to antibody was 13 : 1. Solvent: 200 mM ammonium acetate, pH 6.7.

5) Negative controls

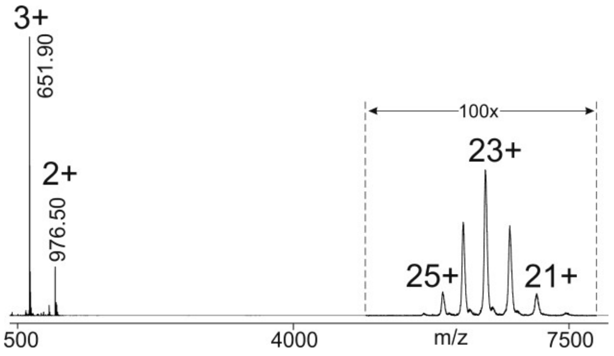

Figure S42: Nano-ESI mass spectrum of His-tag peptide (GPSIVHRKSFHHHHHH) – M7 antibody mixture. Charge states are given for the ion signals of the antibody (right ion series). Magnification factor is shown. Charge states for peptide ion signals and their m/z values are given on the left. Molar ratio of peptide to antibody was 13 : 1. Solvent: 200 mM ammonium acetate, pH 6.7.

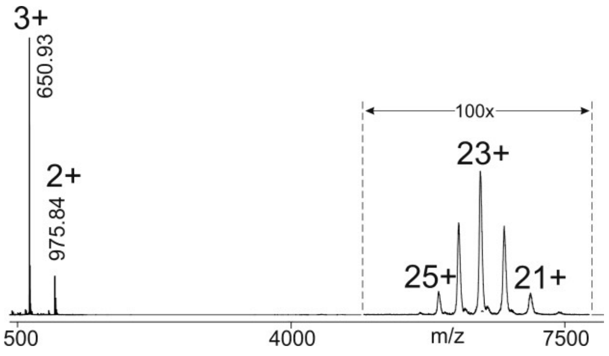

Figure S43: Nano-ESI mass spectrum of His-tag peptide (GPSIVHRKSFHHHHHH) – M11.7 antibody mixture. Charge states are given for the ion signals of the antibody (right ion series). Magnification factor is shown. Charge states for peptide ion signals and their m/z values are given on the left. Molar ratio of peptide to antibody was 13 : 1. Solvent: 200 mM ammonium acetate, pH 6.7.

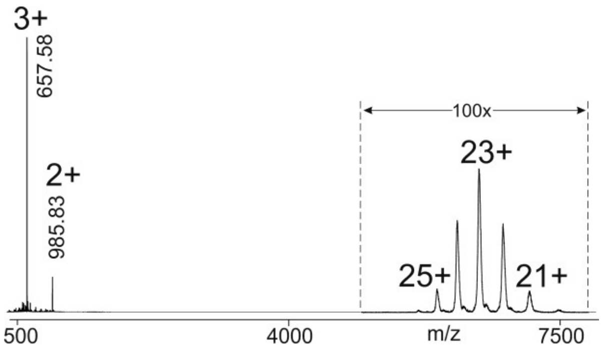

Figure S44: Nano-ESI mass spectrum of peptide 21 (AEQQRIRNEREKERQ) – M7 antibody mixture. Charge states are given for the ion signals of the antibody (right ion series). Magnification factor is shown. Charge states for peptide ion signals and their m/z values are given on the left. Molar ratio of peptide to antibody was 13 : 1. Solvent: 200 mM ammonium acetate, pH 6.7.

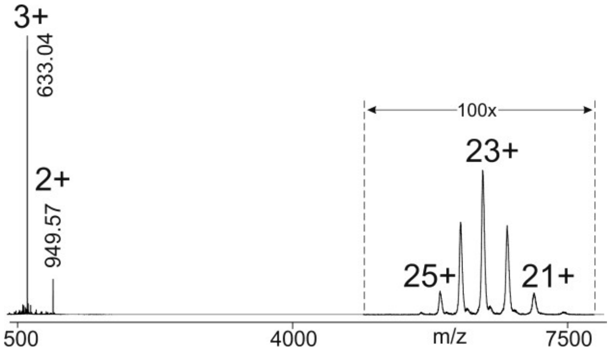

Figure S45: Nano-ESI mass spectrum of peptide 11 (LVSLKDRIERRRAER) – M11.7 antibody mixture. Charge states are given for the ion signals of the antibody (right ion series). Magnification factor is shown. Charge states for peptide ion signals and their m/z values are given on the left. Molar ratio of peptide to antibody was 13 : 1. Solvent: 200 mM ammonium acetate, pH 6.7.

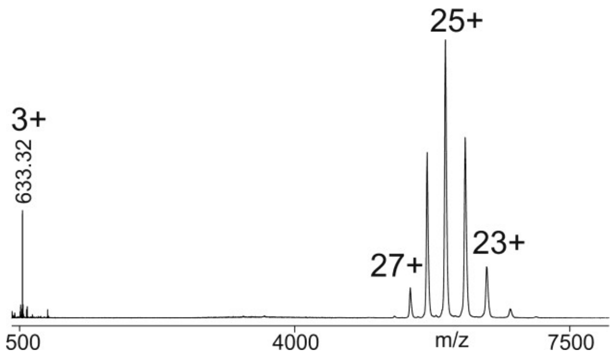

Figure S46: Nano-ESI mass spectrum of peptide 11 (LVSLKDRIERRRAER) – antiTNFα antibody mixture. Charge states are given for the ion signals of the antibody (right ion series). Charge states for the peptide ion signal and its m/z value is given on the left. Molar ratio of peptide to antibody was 13 : 1. Solvent: 200 mM ammonium acetate, pH 6.7.

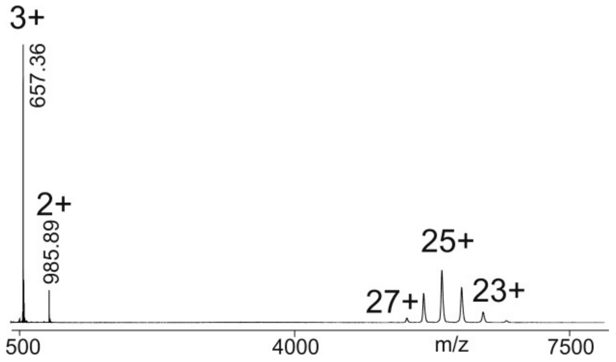

Figure S47: Nano-ESI mass spectrum of peptide 21 (AEQQRIRNEREKERQ) – antiTNFα antibody mixture. Charge states are given for the ion signals of the antibody (right ion series). Charge states for the peptide ion signals and their m/z values are given on the left. Molar ratio of peptide to antibody was 13 : 1. Solvent: 200 mM ammonium acetate, pH 6.7.

6) ITEM-FOUR calculations and data analysis

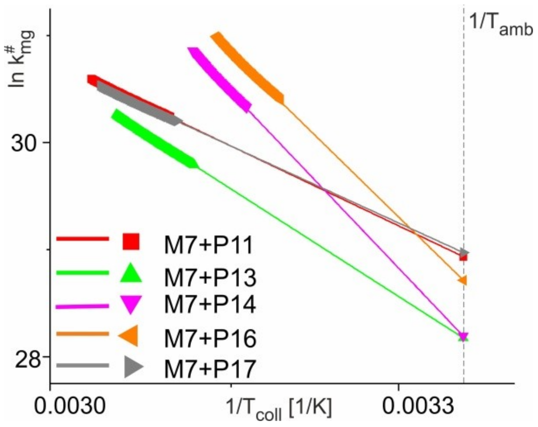

Figure S48: Arrhenius plot for the immune complex dissociations of M7 antibody and epitope peptides P11 (red square), P13 (green triangle), P14 (violet triangle), P16 (orange triangle), and P17 (grey triangle) in the gas phase. Each data point (thickened parts of the lines) has been obtained experimentally and corresponding lines have been extrapolated linearly. The values for $\boldsymbol{ln}\ \mathbf{k}^{\#}_{\mathbf{m0g}}$ are read at $\mathbf{1/T_{amb}}$.

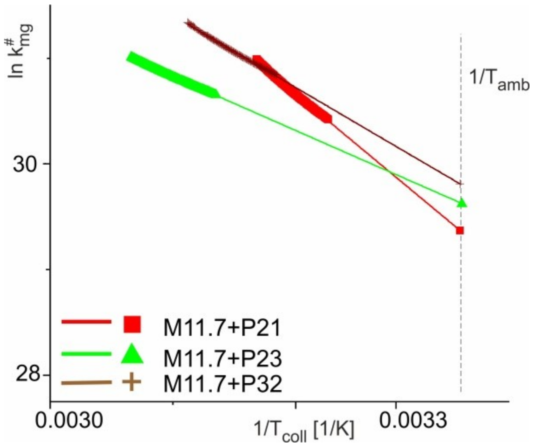

Figure S49: Arrhenius plot for the immune complex dissociations of M11.7 antibody and epitope peptides P21 (red square), P23 (green triangle), and P32 (brown cross) in the gas phase. Each data point (thickened parts of the lines) has been obtained experimentally and corresponding lines have been extrapolated linearly. The values for $\boldsymbol{ln}\ \mathbf{k}^{\#}_{\mathbf{m0g}}$ are read at $\mathbf{1/T_{amb}}$.

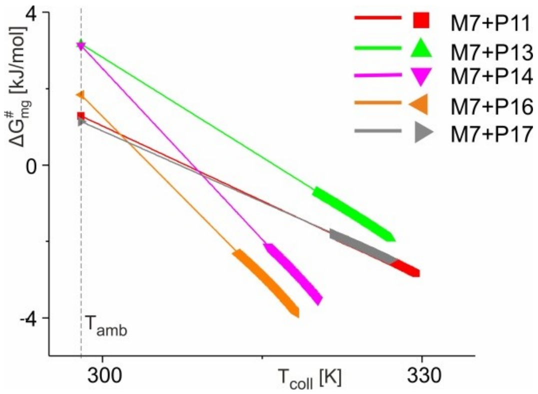

Figure S50: Ellingham diagram for the immune complex dissociations of M7 antibody and epitope peptides P11 (red square), P13 (green triangle), P14 (violet triangle), P16 (orange triangle), and P17 (grey triangle) in the gas phase. Each data point (thickened parts of the lines) has been obtained experimentally and corresponding lines have been extrapolated linearly. The values for $\mathbf{\Delta G^{\#}_{m0g}}$ are read at $\mathbf{T_{amb}}$.

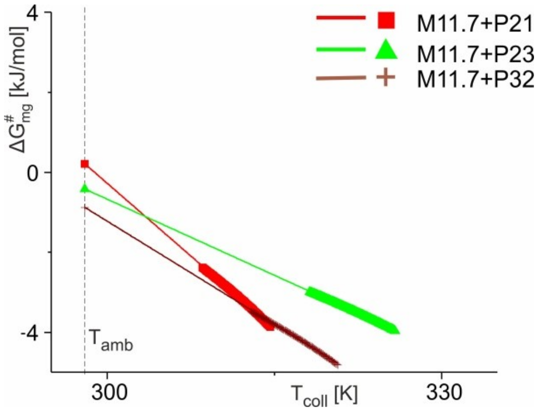

Figure S51: Ellingham diagram for the immune complex dissociations of M11.7 antibody and epitope peptides P21 (red square), P23 (green triangle), and P32 (brown cross) in the gas phase. Each data point (thickened parts of the lines) has been obtained experimentally and corresponding lines have been extrapolated linearly. The values for $\mathbf{\Delta G^{\#}_{m0g}}$ are read at $\mathbf{T_{amb}}$.

7) Molecular modelling

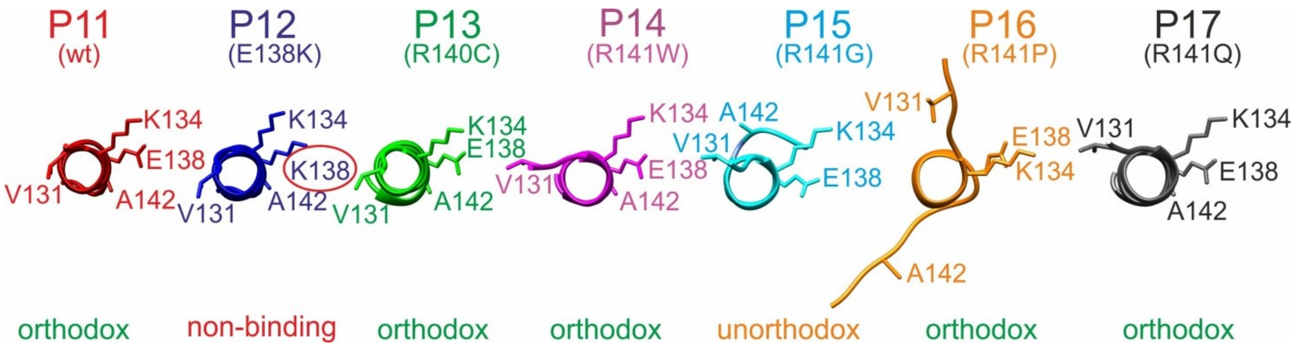

Figure S52: Ribbon cartoons of M7 epitope peptide backbone structure models. Alpha helices of P11 to P17 are shown from top. Selected amino acid residues are shown (stick models) and labeled (single letter code). Wild type (wt) and amino acid exchanges (point mutations) are indicated in parentheses. Amino acid numbering is as in the full-length hcTnT protein. Binding modes with the M7 antibody are given at the bottom. Amino acid residues which prevent binding are circled.

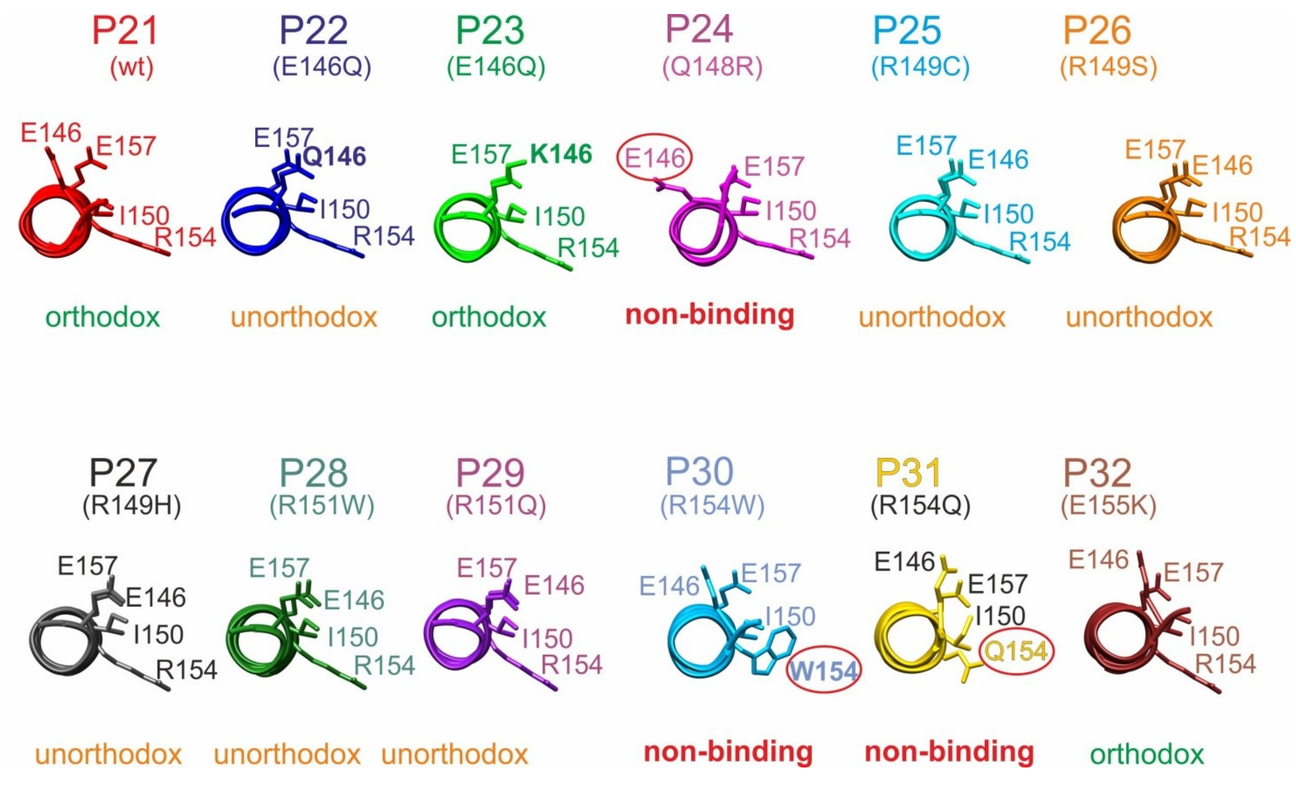

Figure S53: Ribbon cartoons of M11.7 epitope peptide backbone structure models. Alpha helices of P21 to P32 are shown from side Selected amino acid residues are shown (stick models) and labeled (single letter code). Wild type (wt) and amino acid exchanges (point mutations) are indicated in parentheses. Amino acid numbering is as in the full-length hcTnT protein. Binding modes with the M7 antibody are given at the bottom. Amino acid residues which prevent binding are circled.

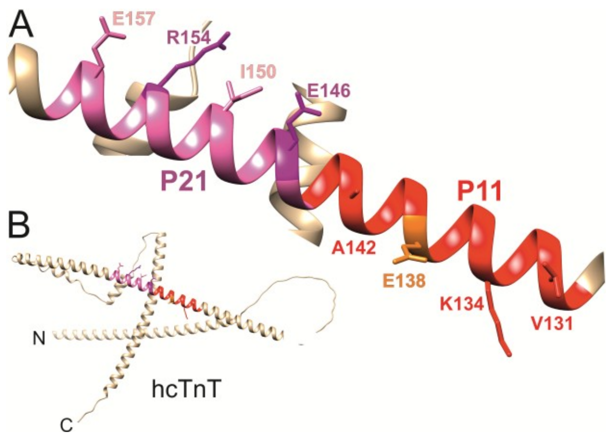

Figure S54: Ribbon cartoons of the hcTnT backbone structure model. A: The alpha helix which contains the M7 (red) and the M11.7 (pink) epitopes is shown. Selected amino acid residues are shown (stick models) and labeled (single letter code). Amino acid residues E138 (orange), E146 (purple) and R154 (purple) are required for antibody binding. B: Structure model of full-length hcTnT (Uniprot accession no.: P45379).

Supplemental Tables

Table S1A: Ion charge states, m/z values, and molecular mass of M7 mAb.

z	m/z	MW (exp.) [Da]
27	5489.23	148209.21
26	5702.98	148277.48
25	5932.81	148320.25
24	6178.71	148289.04
23	6452.13	148398.99
22	6743.48	148356.56
	mean MW	148308.13 ± 66

Table S1B: Ion charge states, m/z values, and molecular mass of M11.7 mAb.

z	m/z	MW (exp.) [Da]
27	5484.87	148091.49
26	5698.09	148150.34
25	5926.84	148171.00
24	6175.73	148217.52
23	6438.91	148094.93
22	6731.95	148102.90
	mean MW	148138.03 ± 51

Table S1C: Ion charge states, m/z values, and molecular mass of anti-TNFα mAb.

z	m/z	MW (exp.) [Da]
27	5503.29	148588.83
26	5714.78	148584.28
25	5943.5	148587.50
24	6191.2	148588.80
23	6460.13	148582.99
22	6754.32	148595.04
	mean MW	148587.91 ± 4

Table S2: Ion species, charge states, m/z values and intensities for anti-Troponin T antibody complexed with Troponin I peptides at measured collision cell voltage differences.

M7+P11, measurement 1

**ion / charge state**	**m/z**	**ΔCV**														
		**2**	**6**	**10**	**14**	**18**	**22**	**26**	**30**	**34**	**40**	**46**	**52**	**58**	**64**	**0**
**peptide 11/ 1+**				0	0	0	0	0	0	0	0	0	0	0		0
**peptide 11/ 2+**	949.38	**n.d.**	**n.d.**	25	90	108	108	228	397	955	1881	1355	1691	2128	**n.d.**	100
**peptide 11/ 3+**	633.298	**n.d.**	**n.d.**	934	1954	3645	3658	6787	10010	15595	22093	12263	13749	13314	**n.d.**	356
**peptide 11/ 4+**	475.26	**n.d.**	**n.d.**	380	807	1311	1318	2429	3380	4628	6152	3223	3586	3367	**n.d.**	166
**peptide 11/ 5+**				0	0	0	0	0	0	0	0	0	0	0		0
**antibody+0 pep/ 28+**		0	0	0	0	0	0	0	0	0	0	0	0	0		0
**antibody+0 pep/ 27+**	5486.38	813	517	538	466	438	424	495	486	421	472	190	228	235	**n.d.**	678
**antibody+0 pep/ 26+**	5697.37	6150	4309	3880	3603	3380	3408	3733	3633	3339	3684	1542	1779	1752	**n.d.**	5551
**antibody+0 pep/ 25+**	5924.91	17211	13714	11135	10186	9802	9868	10565	10274	9671	10613	4750	5464	5290	**n.d.**	16078
**antibody+0 pep/ 24+**	6172.11	22012	18658	14720	13466	13049	13158	13574	13206	13182	14246	6572	7226	7235	**n.d.**	21084
**antibody+0 pep/ 23+**	6440.52	11233	10806	7498	7456	7288	7363	7278	7376	7667	8221	4357	4411	4535	**n.d.**	11229
**antibody+0 pep/ 22+**	6733.86	2651	2930	1840	1892	2004	2019	1991	2003	2350	2649	1584	1502	1628	**n.d.**	2672
**antibody+0 pep/ 21+**	7055.21	391	525	278	295	325	327	344	401	585	737	458	472	501	**n.d.**	442
**antibody+0 pep/ 20+**		0	0	0	0	0	0	0	0	0	0	0	0	0		0
**antibody+1 pep/ 29+**		0	0	0	0	0	0	0	0	0	0	0	0	0		0
**antibody+1 pep/ 28+**		80	80	80	80	80	80	80	80	80	80	80	80	80		80
**antibody+1 pep/ 27+**	5556.98	1532	978	982	885	849	858	897	910	697	714	279	335	319	**n.d.**	100
**antibody+1 pep/ 26+**	5769.6	7548	5746	4920	4384	4082	4111	4337	4137	3619	3735	1540	1652	1674	**n.d.**	1336
**antibody+1 pep/ 25+**	6000.76	13853	11421	8870	8248	7792	7841	7877	7640	6957	7069	3091	3298	3124	**n.d.**	7269
**antibody+1 pep/ 24+**	6429.89	10781	9702	7049	6722	6583	6643	6563	6347	5929	6142	2876	2964	3003	**n.d.**	12919
**antibody+1 pep/ 23+**	6515.06	3912	3980	2546	2576	2665	2690	2560	2581	2549	2671	1464	1417	1485	**n.d.**	10564
**antibody+1 pep/ 22+**	6827.27	849	964	593	614	631	638	627	654	724	2649	473	438	510	**n.d.**	889
**antibody+1 pep/ 21+**		0	0	0	0	0	0	0	0	0	0	0	0			0
**antibody+2 peps/ 29+**		0	0	0	0	0	0	0	0	0	0	0	0			0
**antibody+2 peps/ 28+**		80	80	80	80	80	80	80	80	80	80	80	80			80
**antibody+2 peps/ 27+**	5629.3	743	546	476	410	375	356	384	414	326	303	119	138	127	**n.d.**	671
**antibody+2 peps/ 26+**	5844.44	2152	1743	1400	1241	1181	1191	1195	1085	940	910	375	368	**n.d.**	**n.d.**	2109
**antibody+2 peps/ 25+**	6075.77	2479	2183	1617	1494	1443	1456	1423	1323	1207	1154	490	489	**n.d.**	**n.d.**	2475
**antibody+2 peps/ 24+**	6339.31	1434	1080	888	891	831	839	900	824	758	745	355	301	**n.d.**	**n.d.**	1332
**antibody+2 peps/ 23+**	6613.72	80	80	80	80	80	80	80	80	80	80	80	80	**n.d.**	**n.d.**	466
**antibody+2 peps/ 22+**		80	80	80	80	80	80	80	80	80	80	80	80			80
**antibody+2 peps/ 21+**		0	0	0	0	0	0	0	0	0	0	0	0			0

Table S2: continued

M7 + P11, measurement 2

ion / charge state	m/z	ΔCV 2	6	10	14	18	22	26	30	34	40	46	52	58	64	0
**peptide 11/ 1+**		0	0	0	0	0	0	0	0	0	0	0	0	0		0
**peptide 11/ 2+**	939.395	100	100	20	28	96	213	302	455	759	575	707	922	1533	**n.d.**	20
**peptide 11/ 3+**	588.513	2256	187	606	669	2939	5183	7877	9332	12651	9659	6592	6479	9139	**n.d.**	30
**peptide 11/ 4+**	471.669	537	109	244	262	1141	1825	2682	2989	3776	2850	1722	1718	2398	**n.d.**	80
**peptide 11/ 5+**		0	0	0	0	0	0	0	0	0	0	0	0	0		0
**antibody+0 pep/ 28+**		0	0	0	0	0	0	0	0	0	0	0	0	0		
**antibody+0 pep/ 27+**	5487.281	116	252	299	324	371	422	428	331	375	227	118	94	166	**n.d.**	**n.d.**
**antibody+0 pep/ 26+**	5696.815	793	1798	2249	2367	2785	2921	3091	2490	2688	1607	837	658	1344	**n.d.**	**n.d.**
**antibody+0 pep/ 25+**	5924.263	2406	5306	6515	6734	7579	7752	8106	7031	7805	5052	2552	2199	3977	**n.d.**	**n.d.**
**antibody+0 pep/ 24+**	6171.991	3212	7724	8876	8998	9702	9621	9736	9407	10233	7688	3978	3794	5401	**n.d.**	**n.d.**
**antibody+0 pep/ 23+**	6439.077	1993	4844	5209	5088	5392	5214	5302	5435	5776	5374	2898	3062	3592	**n.d.**	**n.d.**
**antibody+0 pep/ 22+**	6733.554	625	1412	1446	1369	1403	1410	1447	1679	1804	1853	1100	1237	1280	**n.d.**	**n.d.**
**antibody+0 pep/ 21+**	7056.77	145	310	256	242	230	261	307	376	454	470	343	394	100	**n.d.**	**n.d.**
**antibody+0 pep/ 20+**		0	0	0	0	0	0	0	0	0	0	0	0	0		
**antibody+1 pep/ 29+**		0	0	0	0	0	0	0	0	0	0	0	0	0		
**antibody+1 pep/ 28+**		80	80	80	80	80	80	80	80	80	80	80	80	80		
**antibody+1 pep/ 27+**	5556.43	201	445	540	610	688	751	709	533	563	350	168	119	260	**n.d.**	**n.d.**
**antibody+1 pep/ 26+**	5770.53	965	725	2936	2967	3297	3332	3327	2791	2826	1802	8705	663	1193	**n.d.**	**n.d.**
**antibody+1 pep/ 25+**	6004.23	1766	4783	5529	5537	5808	5806	1002	5137	5422	3776	1791	1513	2352	**n.d.**	**n.d.**
**antibody+1 pep/ 24+**	6249.03	1604	4230	4726	4697	4823	4638	4710	4369	4705	3899	1824	1741	2259	**n.d.**	**n.d.**
**antibody+1 pep/ 23+**	6521.76	719	1839	1863	1914	1878	1844	1823	1853	2001	1806	1028	1090	1135	**n.d.**	**n.d.**
**antibody+1 pep/ 22+**	6820.23	196	457	466	440	484	477	501	512	573	577	390	372	385	**n.d.**	**n.d.**
**antibody+1 pep/ 21+**			0	0	0	0	0	0	0	0	0	0	0	0		
**antibody+2 pep/ 29+**			0	0	0	0	0	0	0	0	0	0	0	0		
**antibody+2 pep/ 28+**			80	80	80	80	80	80	80	80	80	80	80	80		
**antibody+2 pep/ 27+**	5630.48	98	128	292	304	304	317	315	265	270	158	72	49	110	**n.d.**	**n.d.**
**antibody+2 pep/ 26+**	5844.52	280	725	871	886	907	908	852	742	777	460	221	185	262	**n.d.**	**n.d.**
**antibody+2 pep/ 25+**	6075.31	323	954	1032	1048	1110	1010	1002	937	933	694	310	250	356	**n.d.**	**n.d.**
**antibody+2 pep/ 24+**	6326.91	100	610	654	644	595	625	603	583	609	512	269	243	267	**n.d.**	**n.d.**
**antibody+2 pep/ 23+**			80	80	80	80	80	80	80	80	80	80	80	80		
**antibody+2 pep/ 22+**			80	80	80	80	80	80	80	80	80	80	80	80		
**antibody+2 pep/ 21+**			0	0	0	0	0	0	0	0	0	0	0	0		

Table S2: continued

M7 + P12, measurement 1

**ion / charge state**	**m/z**	**ΔCV**														
		**2**	**6**	**10**	**14**	**18**	**22**	**26**	**30**	**34**	**40**	**46**	**52**	**58**	**64**	**0**
**peptide 12/ 1+**				0	0	0	0	0	0	0	0	0	0	0		
**peptide 12/ 2+**	948.81	**n.d.**	**n.d.**	133	171	201	121	128	127	134	185	122	133	171	**n.d.**	**n.d.**
**peptide 12/ 3+**	632.98	**n.d.**	**n.d.**	32	256	334	349	557	297	344	451	429	503	337	**n.d.**	**n.d.**
**peptide 12/ 4+**	475.133	**n.d.**	**n.d.**	11	100	100	42	56	27	32	41	46	87	63	**n.d.**	**n.d.**
**peptide 12/ 5+**				0	0	0	0	0	0	0	0	0	0	0		
**antibody+0 pep/ 28+**		0	0	0	0	0	0	0	0	0	0	0	0	0		
**antibody+0 pep/ 27+**	5497.382	99	139	192	393	196	215	226	108	101	111	81	95	89	**n.d.**	**n.d.**
**antibody+0 pep/ 26+**	5707.327	942	1054	1600	3004	1662	1719	1697	844	770	874	656	792	694	**n.d.**	**n.d.**
**antibody+0 pep/ 25+**	5935.533	3014	3623	5346	10221	5474	5590	5408	2650	2651	2880	2352	2734	2382	**n.d.**	**n.d.**
**antibody+0 pep/ 24+**	6184.077	4571	5600	8222	16208	8515	8438	8242	3960	3998	4465	2635	4072	3692	**n.d.**	**n.d.**
**antibody+0 pep/ 23+**	6459.462	2893	3830	5314	10704	5577	5460	5208	2563	2605	2879	2411	2842	2485	**n.d.**	**n.d.**
**antibody+0 pep/ 22+**	6748.52769	881	1179	1554	3229	1641	1639	1529	748	807	918	784	849	839	**n.d.**	**n.d.**
**antibody+0 pep/ 21+**	7072.46308	152	206	256	552	281	279	287	138	160	171	144	199	186	**n.d.**	**n.d.**
**antibody+0 pep/ 20+**		0	0	0	0	0	0	0	0	0	0	0	0	0		
**antibody+1 pep / 28+**		0	0	0	0	0	0	0	0	0	0	0	0	0		
**antibody+1 pep / 27+**		80	80	80	80	80	80	80	80	80	80	80	80	80		
**antibody+1 pep / 26+**	5783.494	211	259	385	719	364	350	360	177	159	173	124	151	122	**n.d.**	**n.d.**
**antibody+1 pep / 25+**	6067.198	500	552	845	1597	839	851	786	387	380	411	305	357	294	**n.d.**	**n.d.**
**antibody+1 pep / 24+**	6295.599	265	671	896	1720	918	890	871	407	437	467	351	405	367	**n.d.**	**n.d.**
**antibody+1 pep / 23+**	6538.841	85	322	467	911	460	450	445	254	235	226	189	217	184	**n.d.**	**n.d.**
**antibody+1 pep / 22+**		80	80	80	80	80	80	80	80	80	80	80	80	80		
**antibody+1 pep / 21+**		80	80	80	80	80	80	80	80	80	80	80	80	80		
**antibody+1 pep / 20+**		0	0	0	0	0	0	0	0	0	0	0	0	0		

Table S2: continued

M7 + P12, measurement 2

ion / charge state	m/z	ΔCV														
		**2**	**6**	**10**	**14**	**18**	**22**	**26**	**30**	**34**	**40**	**46**	**52**	**58**	**64**	**0**
**peptide 12/ 1+**				0	0	0	0	0	0	0	0	0	0	0		
**peptide 12/ 2+**	948.75	**n.d.**	**n.d.**	25	38	19	32	36	53	34	31	37	78	62	**n.d.**	**n.d.**
**peptide 12/ 3+**	632.973	**n.d.**	**n.d.**	41	47	143	272	378	366	295	265	210	301	252	**n.d.**	**n.d.**
**peptide 12/ 4+**	475.022	**n.d.**	**n.d.**	21	24	65	134	160	139	125	87	53	121	95	**n.d.**	**n.d.**
**peptide 12/ 5+**				0	0	0	0	0	0	0	0	0	0	0		
**antibody+0 pep/ 28+**		0	0	0	0	0	0	0	0	0	0	0	0	0		0
**antibody+0 pep/ 27+**	5501.273	95	134	151	96	132	193	185	139	80	67	59	112	100	**n.d.**	42
**antibody+0 pep/ 26+**	5711.047	804	1065	1281	831	1001	1497	1314	1049	570	467	418	760	721	**n.d.**	373
**antibody+0 pep/ 25+**	5937.769	2677	3699	4307	2621	3254	4614	4218	3351	1969	1540	1398	2498	2369	**n.d.**	1407
**antibody+0 pep/ 24+**	6185.637	4313	5741	6651	4245	5195	6703	6083	5077	3068	2306	2295	3915	3802	**n.d.**	2368
**antibody+0 pep/ 23+**	6455.821	2913	4034	4552	2876	3390	4373	3763	3343	2005	1601	1616	2797	2663	**n.d.**	1318
**antibody+0 pep/ 22+**	6750.453	911	1236	1415	940	1039	1303	1172	998	687	502	521	953	908	**n.d.**	584
**antibody+0 pep/ 21+**	7072.845	179	203	260	171	181	220	185	182	130	114	117	186	202	**n.d.**	118
**antibody+0 pep/ 20+**		0	0	0	0	0	0	0	0	0	0	0	0	0		0
**antibody+1 pep / 28+**			0	0	0	0	0	0	0	0	0	0	0	0		0
**antibody+1 pep / 27+**			80	80	80	80	80	80	80	80	80	80	80	80		80
**antibody+1 pep / 26+**	5787.331	205	266	309	205	217	303	280	205	114	77	80	125	121	**n.d.**	103
**antibody+1 pep / 25+**	6015.762	**n.d.**	618	696	414	536	649	562	461	264	194	167	309	329	**n.d.**	230
**antibody+1 pep / 24+**	6265.597	**n.d.**	671	745	475	517	732	651	514	301	250	207	384	353	**n.d.**	277
**antibody+1 pep / 23+**	6542.475	**n.d.**	346	427	269	282	357	323	260	156	124	90	205	189	**n.d.**	164
**antibody+1 pep / 22+**			80	80	80	80	80	80	80	80	80	80	80	80		80
**antibody+1 pep / 21+**			80	80	80	80	80	80	80	80	80	80	80	80		80
**antibody+1 pep / 20+**			0	0	0	0	0	0	0	0	0	0	0	0		0

Table S2: continued

M7+P13, measurement 1

ion / charge state	m/z	ΔCV														
		2	6	10	14	18	22	26	30	34	40	46	52	58	64	0
**peptide 13/ 1+**		0	0	0	0	0	0	0	0	0	0	0	0	0		
**peptide 13/ 2+**	922.77	100	100	100	159	1137	505	1970	2930	4323	5814	7824	5278	8293	**n.d.**	**n.d.**
**peptide 13/ 3+**	615.589	205	300	806	1968	12866	4991	18072	23669	29893	34604	41401	25265	34516	**n.d.**	**n.d.**
**peptide 13/ 4+**	461.1	100	65	132	364	1818	695	2148	2707	3392	3802	4247	2553	3241	**n.d.**	**n.d.**
**peptide 13/ 5+**		0	0	0	0	0	0	0	0	0	0	0	0	0		
**antibody+0 pep/ 28+**		0	0	0	0	0	0	0	0	0	0	0	0	0		
**antibody+0 pep/ 27+**	5490.715	160	188	214	259	889	203	611	646	594	515	488	260	315	**n.d.**	**n.d.**
**antibody+0 pep/ 26+**	5699.64	1255	1412	1618	1820	6621	1564	4149	4063	4042	3222	3256	1719	2222	**n.d.**	**n.d.**
**antibody+0 pep/ 25+**	5926.425	3707	4257	4715	5164	18389	4228	10575	9888	10095	7977	8124	4745	6329	**n.d.**	**n.d.**
**antibody+0 pep/ 24+**	6180.259	4519	4994	5487	5921	20146	4880	11760	11062	11137	9760	9627	5956	8249	**n.d.**	**n.d.**
**antibody+0 pep/ 23+**	6442.542	2270	2471	2662	2871	9652	2541	5988	5665	5799	5427	5689	3720	5204	**n.d.**	**n.d.**
**antibody+0 pep/ 22+**	6735.774	527	549	642	697	2395	686	1826	1861	1990	1998	2225	1679	2244	**n.d.**	**n.d.**
**antibody+0 pep/ 21+**		80	80	80	80	80	80	80	80	80	80	80	80	80		
**antibody+0 pep/ 20+**		0	0	0	0	0	0	0	0	0	0	0	0	0		
**antibody+1 pep/ 28+**		0	0	0	0	0	0	0	0	0	0	0	0	0		
**antibody+1 pep/ 27+**	5556.82	398	483	545	599	2240	483	1430	1394	1373	997	964	451	574	**n.d.**	**n.d.**
**antibody+1 pep/ 26+**	5770.092	2324	2800	2996	3381	11572	2604	6892	6533	6443	4604	4466	2349	2919	**n.d.**	**n.d.**
**antibody+1 pep/ 25+**	6000.773	4957	5484	6010	6476	22409	5106	12364	11242	11087	8551	7874	4752	6060	**n.d.**	**n.d.**
**antibody+1 pep/ 24+**	6250.795	4069	4378	4873	5176	17383	4399	9984	9002	8992	7096	6930	4408	5694	**n.d.**	**n.d.**
**antibody+1 pep/ 23+**	6522.586	1536	1584	1835	1918	6132	1622	4019	3531	3674	3195	3181	2238	2881	**n.d.**	**n.d.**
**antibody+1 pep/ 22+**	6819.632	309	322	355	393	1347	414	1017	1012	1092	1074	1117	795	1098	**n.d.**	**n.d.**
**antibody+1 pep/ 21+**	7149.954	80	71	60	86	241	73	226	218	273	264	375	269	395	**n.d.**	**n.d.**
**antibody+1 pep/ 20+**		0	0	0	0	0	0	0	0	0	0	0	0	0		
**antibody+2 peps/ 29+**		0	0	0	0	0	0	0	0	0	0	0	0	0		
**antibody+2 peps/ 28+**		80	80	80	80	80	80	80	80	80	80	80	80	80		
**antibody+2 peps/ 27+**	5628.916	300	327	349	382	1367	311	870	851	755	518	460	225	282	**n.d.**	**n.d.**
**antibody+2 peps/ 26+**	5841.244	1115	1228	1365	1428	4822	1087	2821	2455	2315	1676	1344	775	937	**n.d.**	**n.d.**
**antibody+2 peps/ 25+**	6075.001	1634	1680	1813	1985	6325	1499	3430	3088	2989	2167	1886	1188	1375	**n.d.**	**n.d.**
**antibody+2 peps/ 24+**	6327.142	905	953	1084	1138	3627	941	2215	1887	1842	1322	1188	754	979	**n.d.**	**n.d.**
**antibody+2 peps/ 23+**	6604.453	308	302	322	346	1073	288	719	636	682	481	486	365	392	**n.d.**	**n.d.**
**antibody+2 peps/ 22+**		80	80	80	80	80	80	80	80	80	80	80	80	80		
**antibody+2 peps/ 21+**		0	0	0	0	0	0	0	0	0	0	0	0	0		

Table S2: continued

M7+P13, measurement 2

ion / charge state	m/z	ΔCV 2	6	10	14	18	22	26	30	34	40	46	52	58	64	0
**peptide 13/ 1+**		0	0	0	0	0	0	0	0	0	0	0	0	0		
**peptide 13/ 2+**	922.846	100	71	90	181	243	646	622	803	1014	1184	1314	1499	1550	**n.d.**	**n.d.**
**peptide 13/ 3+**	615.595	327	811	1241	2185	2856	6257	5767	5832	7162	6106	6225	6342	5800	**n.d.**	**n.d.**
**peptide 13/ 4+**	461.968	100	153	167	324	400	826	677	710	809	704	719	703	676	**n.d.**	**n.d.**
**peptide 13/ 5+**		0	0	0	0	0	0	0	0	0	0	0	0	0		
**antibody+0 pep/ 28+**		0	0	0	0	0	0	0	0	0	0	0	0	0	0	
**antibody+0 pep/ 27+**	5488.023	167	281	192	144	102	135	87	70	94	66	46	47	49	167	**n.d.**
**antibody+0 pep/ 26+**	5699.766	1307	2367	1394	1247	800	1028	656	559	561	375	360	370	382	1307	**n.d.**
**antibody+0 pep/ 25+**	5926.008	4282	6980	4304	3734	2544	3259	2178	1659	1754	1191	11334	1217	1199	4282	**n.d.**
**antibody+0 pep/ 24+**	6180.232	5938	9178	5578	5379	3619	4820	3025	2317	2429	1723	1622	1755	1707	5938	**n.d.**
**antibody+0 pep/ 23+**	6441.003	3534	5285	3389	3267	2206	3060	1913	1581	1579	1137	1177	1179	1124	3534	**n.d.**
**antibody+0 pep/ 22+**	6735.014	923	1407	927	902	663	1077	688	613	602	475	540	558	564	923	**n.d.**
**antibody+0 pep/ 21+**	7056.491	152	239	161	186	160	233	183	184	234	190	221	249	256	152	**n.d.**
**antibody+1 pep/ 20+**		0	0	0	0	0	0	0	0	0	0	0	0	0	0	
**antibody+1 pep/ 28+**		0	0	0	0	0	0	0	0	0	0	0	0	0	0	
**antibody+1 pep/ 27+**	5557.229	453	797	282	393	247	323	199	158	155	112	95	103	106	453	**n.d.**
**antibody+1 pep/ 26+**	5771.236	2767	4702	2735	2313	1521	2053	1245	954	991	655	556	559	565	2767	**n.d.**
**antibody+1 pep/ 25+**	6001.015	6257	10208	5764	5486	3505	4688	32814	2216	2165	1404	1274	1275	1272	6257	**n.d.**
**antibody+1 pep/ 24+**	6251.035	6012	9113	5656	5323	3598	4801	2805	2104	2144	1444	1316	1354	1340	6012	**n.d.**
**antibody+1 pep/ 23+**	6524.334	2551	3858	2382	2458	1601	2309	1416	1122	1032	690	731	699	708	2551	**n.d.**
**antibody+1 pep/ 22+**	6819.992	673	875	546	577	431	639	421	356	366	294	290	295	287	673	**n.d.**
**antibody+1 pep/ 21+**	7168.677	87	175	99	101	92	145	98	99	109	96	101	111	113	87	**n.d.**
**antibody+1 pep/ 20+**		0	0	0	0	0	0	0	0	0	0	0	0	0	0	
		0	0	0	0	0	0	0	0	0	0	0	0	0		
**antibody+2 peps/ 28+**		80	80	80	80	80	80	80	80	80	80	80	80	80		
**antibody+2 peps/ 27+**	5628.059	327	541	371	273	150	216	166	104	119	62	71	60	63	**n.d.**	**n.d.**
**antibody+2 peps/ 26+**	5839.522	1393	2243	1275	1136	703	951	569	448	433	240	209	206	201	**n.d.**	**n.d.**
**antibody+2 peps/ 25+**	6073.686	2250	3422	2023	1906	1172	1568	936	703	637	393	329	255	320	**n.d.**	**n.d.**
**antibody+2 peps/ 24+**	6336.245	1513	2177	1363	1311	888	1228	711	492	470	317	273	263	257	**n.d.**	**n.d.**
**antibody+2 peps/ 23+**	6605.119	511	705	443	460	430	426	255	199	191	126	114	119	112	**n.d.**	**n.d.**
**antibody+2 peps/ 22+**		80	80	80	80	80	80	80	80	80	80	80	80	80		
**antibody+2 peps/ 21+**		0	0	0	0	0	0	0	0	0	0	0	0	0		

Table S2: continued

M7+P14, measurement 1

ion / charge state	m/z	ΔCV 2	6	10	14	18	22	26	30	34	40	46	52	58	64	0
**peptide 14/ 1+**		0	0	0	0	0	0	0	0	0	0	0	0	0		0
**peptide 14/ 2+**	964.62	159	497	1130	3341	2683	11760	11670	10620	21940	74630	44670	45700	25140	n.d.	22
**peptide 14/ 3+**	643.41	706	4570	7061	28730	21710	49140	122500	108700	116600	169100	87890	67700	49440	n.d.	199
**peptide 14/ 4+**		400	400	400	400	400	400	400	400	400	400	400	400	400		100
**peptide 14/ 5+**		0	0	0	0	0	0	0	0	0	0	0	0	0		0
**antibody+0 pep/ 28+**		0	0	0	0	0	0	0	0	0	0	0	0	0		0
**antibody+0 pep/ 27+**	5488.281	160	544	728	690	469	696	797	581	555	1247	632	564	284	n.d.	27
**antibody+0 pep/ 26+**	5698.188	1258	4497	5658	5332	3739	5307	6065	4158	3766	8539	4544	3979	1946	n.d.	208
**antibody+0 pep/ 25+**	5923.732	4632	14080	16190	15720	11180	15600	16760	11710	10570	22470	11200	10120	4842	n.d.	684
**antibody+0 pep/ 24+**	6170.326	6492	17860	19960	19020	13570	18930	18940	13250	11940	25000	12530	11130	5381	n.d.	1079
**antibody+0 pep/ 23+**	6439.477	3742	9222	9803	9789	7123	9706	9669	6842	6054	12840	6748	5832	2836	n.d.	704
**antibody+0 pep/ 22+**	6733.157	1015	2311	2357	2432	1780	2701	2843	2038	1960	4579	2402	2335	1161	n.d.	208
**antibody+0 pep/ 21+**	7055.247	160	324	325	354	327	494	602	477	523	1361	837	841	393	n.d.	49
**antibody+0 pep/ 20+**		0	0	0	0	0	0	0	0	0	0	0	0	0		0
**antibody+1 pep/ 28+**		0	0	0	0	0	0	0	0	0	0	0	0	0		0
**antibody+1 pep/ 27+**	5557.257	312	1305	1587	1598	1104	1571	1709	1155	1015	2184	993	882	427	n.d.	46
**antibody+1 pep/ 26+**	5770.426	2004	7322	8763	8402	5849	7925	8312	5673	4886	9762	4711	3930	1876	n.d.	290
**antibody+1 pep/ 25+**	6000.904	4610	14170	16900	16030	11220	15100	14800	10000	8743	16850	7987	6828	3176	n.d.	721
**antibody+1 pep/ 24+**	6251.466	4204	11570	12780	12720	8899	12100	11420	7431	6340	12750	5961	5180	2447	n.d.	695
**antibody+1 pep/ 23+**	6524.555	1681	4301	4557	4588	3239	4536	4412	2941	2505	5180	2570	2308	1065	n.d.	316
**antibody+1 pep/ 22+**	6822.38	418	1010	1067	1078	801	1186	1200	878	772	1661	860	799	370	n.d.	99
**antibody+1 pep/ 21+**	7147.152	72	139	156	203	148	209	228	211	187	406	255	235	137	n.d.	15
**antibody+1 pep/ 20+**		0	0	0	0	0	0	0	0	0	0	0	0	0		0
**antibody+2 peps/ 29+**		0	0	0	0	0	0	0	0	0	0	0	0	0		0
**antibody+2 peps/ 28+**		80	80	80	80	80	80	80	80	80	80	80	80	80		80
**antibody+2 peps/ 27+**	5632.034	192	739	943	919	610	845	869	600	485	911	441	363	157	n.d.	30
**antibody+2 peps/ 26+**	5846.293	715	2546	3177	2964	2156	2703	2773	1833	1510	2704	1180	974	426	n.d.	119
**antibody+2 peps/ 25+**	6078.716	1037	3325	3809	3644	2661	3332	3140	2008	1656	3029	1281	1079	503	n.d.	164
**antibody+2 peps/ 24+**	6333.555	639	1752	2049	2087	1399	1898	1652	1074	894	1665	814	616	299	n.d.	123
**antibody+2 peps/ 23+**	6606.027	230	601	689	638	466	668	624	434	371	642	332	268	142	n.d.	52
**antibody+2 peps/ 22+**		80	80	80	80	80	80	80	80	80	80	80	80	80		80
**antibody+2 peps/ 21+**		0	0	0	0	0	0	0	0	0	0	0	0	0		0

Table S2: continued

M7+P14, measurement 2

**ion / charge state**	**m/z**	**ΔCV**														
		**2**	**6**	**10**	**14**	**18**	**22**	**26**	**30**	**34**	**40**	**46**	**52**	**58**	**64**	**0**
**peptide 14/ 1+**		0	0	0	0	0	0	0	0	0	0	0	0	0		0
**peptide 14/ 2+**	964.62	274	299	1012	871	3807	7837	4718	11370	12920	11940	18770	22690	16550	**n.d.**	513
**peptide 14/ 3+**	643.41	1429	3662	5668	9461	17500	57870	46220	69540	63770	29150	61420	30490	53300	**n.d.**	2575
**peptide 14/ 4+**		400	400	400	400	400	400	400	400	400	400	400	400	400		400
**peptide 14/ 5+**		0	0	0	0	0	0	0	0	0	0	0	0	0		0
**antibody+0 pep/ 28+**		0	0	0	0	0	0	0	0	0	0	0	0	0		0
**antibody+0 pep/ 27+**	5487.091	542	475	748	486	437	592	337	425	402	280	336	318	304	**n.d.**	47
**antibody+0 pep/ 26+**	5698.635	3621	3169	4748	3474	3248	3933	2248	2996	2603	1642	2001	2109	2173	**n.d.**	280
**antibody+0 pep/ 25+**	5924.017	9368	7961	12010	9372	8828	10170	6194	7710	6506	4022	4899	5095	5596	**n.d.**	828
**antibody+0 pep/ 24+**	6170.686	10720	8838	13310	10420	10100	11370	6845	8331	6954	4379	5339	5515	6188	**n.d.**	947
**antibody+0 pep/ 23+**	6439.411	4936	3912	6189	4983	5155	5624	3406	4075	3385	2287	2906	3067	3476	**n.d.**	500
**antibody+0 pep/ 22+**	6732.806	1202	915	1626	1286	1458	1647	1122	1303	1120	840	1077	1194	1417	**n.d.**	147
**antibody+0 pep/ 21+**	7053.485	178	144	278	229	243	307	277	327	331	247	323	389	443	**n.d.**	34
**antibody+0 pep/ 20+**		0	0	0	0	0	0	0	0	0	0	0	0	0		0
**antibody+0 pep/ 28+**		0	0	0	0	0	0	0	0	0	0	0	0	0		0
**antibody+1 pep/ 27+**	5557.611	1159	1055	1434	1033	951	1062	626	808	687	434	454	461	456	**n.d.**	87
**antibody+1 pep/ 26+**	5770.169	5446	4579	6711	5053	4466	5125	3056	3744	3099	1734	2025	1929	2105	**n.d.**	391
**antibody+1 pep/ 25+**	6001.639	9163	7657	11350	8684	8175	8960	5152	6263	4999	2928	3270	3262	3541	**n.d.**	727
**antibody+1 pep/ 24+**	6249.867	6603	5461	7857	6415	6244	6757	4028	4495	3683	2159	2567	2579	2838	**n.d.**	591
**antibody+1 pep/ 23+**	6523.864	2200	1783	2827	2299	2323	2522	1604	1768	1446	848	1105	1197	1308	**n.d.**	239
**antibody+1 pep/ 22+**	6820.025	501	419	673	547	591	696	457	510	418	328	405	417	507	**n.d.**	71
**antibody+1 pep/ 21+**	7149.066	94	73	117	120	106	152	96	109	99	89	121	123	153	**n.d.**	26
**antibody+0 pep/ 20+**		0	0	0	0	0	0	0	0	0	0	0	0	0		0
**antibody+2 peps/ 29+**		0	0	0	0	0	0	0	0	0	0	0	0	0		0
**antibody+2 peps/ 28+**		80	80	80	80	80	80	80	80	80	80	80	80	80		80
**antibody+2 peps/ 27+**	5632.915	690	526	741	543	509	560	334	375	318	183	187	189	177	**n.d.**	53
**antibody+2 peps/ 26+**	5846.976	1856	1576	2197	1658	1465	1628	911	1092	994	468	494	472	490	**n.d.**	148
**antibody+2 peps/ 25+**	6077.552	2021	1682	2345	1794	1732	1842	1020	1163	898	519	542	523	537	**n.d.**	174
**antibody+2 peps/ 24+**	6331.229	954	829	1267	998	946	1042	576	676	504	319	337	333	348	**n.d.**	115
**antibody+2 peps/ 23+**	6606.367	326	261	378	319	318	365	251	259	216	137	154	147	167	**n.d.**	39
**antibody+2 peps/ 22+**		80	80	80	80	80	80	80	80	80	80	80	80	80		80
**antibody+2 peps/ 21+**		0	0	0	0	0	0	0	0	0	0	0	0	0		0

Table S2: continued

M7+P15, measurement 1

ion / charge state	m/z	ΔCV														
		2	6	10	14	18	22	26	30	34	40	46	52	58	64	0
**peptide 15/ 1+**		0	0	0	0	0	0	0	0	0	0	0	0	0		
**peptide 15/ 2+**	899.836	20	10	35	9	51	42	48	187	497	293	388	638	403	**n.d.**	**n.d.**
**peptide 15/ 3+**	600.243	24	58	178	85	492	322	394	1281	2407	1076	1211	1482	700	**n.d.**	**n.d.**
**peptide 15/ 4+**	450.457	20	20	40	16	76	62	39	176	294	157	209	252	148	**n.d.**	**n.d.**
**peptide 15/ 5+**		0	0	0	0	0	0	0	0	0	0	0	0	0		
**antibody+0 pep/ 28+**		0	0	0	0	0	0	0	0	0	0	0	0	0		
**antibody+0 pep/ 27+**	5475.502	112	117	123	50	98	44	44	92	128	56	55	82	55	**n.d.**	**n.d.**
**antibody+0 pep/ 26+**	5698.731	742	760	885	279	629	324	272	595	983	337	402	662	375	**n.d.**	**n.d.**
**antibody+0 pep/ 25+**	5930.745	2072	2363	2976	752	986	972	742	1767	3043	1042	1201	1921	1214	**n.d.**	**n.d.**
**antibody+0 pep/ 24+**	6173.403	2958	3184	3973	910	2577	1256	951	2159	3891	1393	1581	2763	1684	**n.d.**	**n.d.**
**antibody+0 pep/ 23+**	6440.003	1681	1896	2217	551	1480	678	539	1226	2266	806	1025	1710	980	**n.d.**	**n.d.**
**antibody+0 pep/ 22+**	6733.853	457	495	624	162	417	188	163	356	649	269	330	555	309	**n.d.**	**n.d.**
**antibody+0 pep/ 21+**	7057.203	74	15	124	30	84	53	41	80	161	75	93	169	106	**n.d.**	**n.d.**
**antibody+0 pep/ 20+**		0	0	0	0	0	0	0	0	0	0	0	0	0		
**antibody+1 pep/ 29+**		0	0	0	0	0	0	0	0	0	0	0	0	0		
**antibody+1 pep/ 28+**		80	80	80	80	80	80	80	80	80	80	80	80	80		
**antibody+1 pep/ 27+**	5557.294	126	100	147	46	49	57	43	92	135	49	55	75	48	**n.d.**	**n.d.**
**antibody+1 pep/ 26+**	5765.69	609	633	762	232	511	241	179	397	672	217	255	389	225	**n.d.**	**n.d.**
**antibody+1 pep/ 25+**	5996.891	1134	1358	1511	391	986	447	369	770	1292	444	490	774	447	**n.d.**	**n.d.**
**antibody+1 pep/ 24+**	6244.729	977	1096	1335	320	882	423	328	667	1119	411	442	700	430	**n.d.**	**n.d.**
**antibody+1 pep/ 23+**	6517.929	417	445	578	122	355	172	163	289	477	191	206	336	196	**n.d.**	**n.d.**
**antibody+1 pep/ 22+**	5557.294	126	100	147	46		57	43	92	135	49	55	75	48	**n.d.**	**n.d.**
**antibody+1 pep/ 21+**		0	0	0	0	0	0	0	0	0	0	0	0	0		

Table S2: continued

M7+P15, measurement 2

ion / charge state	m/z	ΔCV 2	6	10	14	18	22	26	30	34	40	46	52	58	64	0
**peptide 15/ 1+**		0	0	0	0	0	0	0	0	0	0	0	0	0		
**peptide 15/ 2+**	899.805	100	22	28	77	101	162	329	453	451	688	883	889	840	**n.d.**	**n.d.**
**peptide 15/ 3+**	600.256	121	168	306	518	736	1112	2168	2536	2167	2804	3006	2201	1665	**n.d.**	**n.d.**
**peptide 15/ 4+**	450.450	100	59	41	111	112	179	344	330	302	387	447	371	326	**n.d.**	**n.d.**
**peptide 15/ 5+**		0	0	0	0	0	0	0	0	0	0	0	0	0		
**antibody+0 pep/ 28+**		0	0	0	0	0	0	0	0	0	0	0	0	0		
**antibody+0 pep/ 27+**	5486.892	215	276	180	126	116	103	172	167	113	156	129	111	93	**n.d.**	**n.d.**
**antibody+0 pep/ 26+**	5697.961	1600	2053	1347	1132	951	881	1645	1173	846	1029	977	866	799	**n.d.**	**n.d.**
**antibody+0 pep/ 25+**	5926.879	4790	5960	4197	3576	3024	2767	4804	3437	2633	2860	2887	2708	2569	**n.d.**	**n.d.**
**antibody+0 pep/ 24+**	6171.878	6058	7737	5549	5176	4208	3778	6348	3611	3339	3800	3806	3600	3549	**n.d.**	**n.d.**
**antibody+0 pep/ 23+**	6439.855	3164	4165	3133	3078	2509	2234	3636	2508	1942	2132	2182	2240	2126	**n.d.**	**n.d.**
**antibody+0 pep/ 22+**	6732.145	777	1049	807	832	703	664	998	827	574	638	719	776	773	**n.d.**	**n.d.**
**antibody+0 pep/ 21+**	7060.829	144	183	130	100	121	118	207	165	136	130	204	205	211	**n.d.**	**n.d.**
**antibody+0 pep/ 20+**		0	0	0	0	0	0	0	0	0	0	0	0	0		
**antibody+1 pep/ 29+**		0	0	0	0	0	0	0	0	0	0	0	0	0		
**antibody+1 pep/ 28+**		80	80	80	80	80	80	80	80	80	80	80	80	80		
**antibody+1 pep/ 27+**	5267.481	251	324	209	163	156	122	224	176	132	123	110	114	83	**n.d.**	**n.d.**
**antibody+1 pep/ 26+**	5845.605	1390	1714	1112	928	790	709	1171	825	584	603	590	509	456	**n.d.**	**n.d.**
**antibody+1 pep/ 25+**	5998.915	2478	3199	2262	1932	1627	1453	2259	1633	1159	1129	1174	1034	946	**n.d.**	**n.d.**
**antibody+1 pep/ 24+**	6254.144	2052	2600	1901	1804	1466	1231	2123	1435	985	1041	1027	1007	899	**n.d.**	**n.d.**
**antibody+1 pep/ 23+**	6518.574	770	979	783	789	609	525	872	607	426	482	445	467	429	**n.d.**	**n.d.**
**antibody+1 pep/ 22+**	6791.418	198	246	186	171	185	156	226	179	132	166	139	165	158	**n.d.**	**n.d.**
**antibody+1 pep/ 21+**		0	0	0	0	0	0	0	0	0	0	0	0	0		

Table S2: continued

M7+P16, measurement 1

ion / charge state	m/z	ΔCV													
		2	6	10	14	18	22	26	30	34	40	46	52	58	64	0
**peptide 16/ 1+**		0	0	0	0	0	0	0	0	0	0	0	0	0		0
**peptide 16/ 2+**	919.54	305	735	1531	3261	7469	13650	24390	59160	91720	94620	110100	91640	78210	**n.d.**	236
**peptide 16/ 3+**	613.36	2019	7486	17800	28920	57270	150400	397400	383200	412400	411800	420500	212100	243400	**n.d.**	1726
**peptide 16/ 4+**	460.27	240	993	2367	5538	9887	17070	23530	48290	26110	21800	23730	18550	25620	**n.d.**	409
**peptide 16/ 5+**		0	0	0	0	0	0	0	0	0	0	0	0	0		0
**antibody+0 pep/ 28+**						0	0	0								
**antibody+0 pep/ 27+**	5489.416	21	35	35	82	146	162	141	146	147	125	133	115	73		14
**antibody+0 pep/ 26+**	5700.217	77	267	381	866	1353	2578	1398	1398	1481	1205	1141	1105	750		61
**antibody+0 pep/ 25+**	6010.617	459	1518	2038	4131	6031	6554	6009	6977	7041	5725	5258	4550	3558		317
**antibody+0 pep/ 24+**	6173.437	1585	3859	4988	8938	12010	12680	12420	16750	17350	13150	12390	10060	7684		994
**antibody+0 pep/ 23+**	6444.4	2195	4450	5449	8356	10380	10810	11510	18380	19070	13340	13070	10350	8003		1498
**antibody+0 pep/ 22+**	6735.813	1257	2193	2518	3324	3964	4362	5012	8939	10020	7074	7100	5387	4272		833
**antibody+0 pep/ 21+**	7060.71	310	454	537	679	880	1038	1375	2830	3360	2668	2610	1994	1585		203
**antibody+0 pep/ 20+**		80	80	80	80	0	0	0	80	80	80	80	80	80		80
**antibody+0 pep/ 19+**		0	0	0	0				0	0	0	0	0	0		0
**antibody+1 pep/ 28+**		0	0	0	0	0	0	0	0	0	0	0	0	0		0
**antibody+1 pep/ 27+**	5559	34	100	106	286	533	573	550	501	530	374	346	327	206		22
**antibody+1 pep/ 26+**	5770.084	250	925	1226	2621	3961	4124	3597	3994	1716	2733	2494	2206	1519		187
**antibody+1 pep/ 25+**	5900.092	1115	3482	4647	8577	11060	11210	10160	12630	11890	8580	7843	6352	4908		757
**antibody+1 pep/ 24+**	6250.73	2171	5464	6871	11350	13640	14420	13350	19620	18990	12360	11860	8898	6871		1585
**antibody+1 pep/ 23+**	6519.835	1855	3792	4519	6332	7550	7880	8118	13890	14450	8885	8063	6114	4824		1435
**antibody+1 pep/ 22+**	6821.912	784	1327	1541	1924	2313	2547	2862	5289	5641	3638	3439	2406	1851		581
**antibody+1 pep/ 21+**	7143.273	223	271	318	399	459	529	693	1290	1605	1260	1195	923	764		150
**antibody+1 pep/ 20+**		0	0	0	0	0	0	0	0	0	0	0	0	0		0
**antibody+2 peps/ 29+**			0	0	0	0	0	0	0	0	0	0	0	0		0
**antibody+2 peps/ 28+**		0	80	80	80	80	80	80	80	80	80	80	80	80		0
**antibody+2 peps/ 27+**	5636.919	42	149	152	324	509	544	487	516	477	310	293	254	206		41
**antibody+2 peps/ 26+**	5840.104	217	781	1013	1784	2360	2308	2055	2388	2074	1373	1175	973	718		198
**antibody+2 peps/ 25+**	6073.542	549	1667	2161	3479	4375	4245	3808	4987	4675	2834	2475	1813	1426		439
**antibody+2 peps/ 24+**	6325.973	635	1613	1937	2858	3247	3332	3194	4921	4534	2631	2413	1684	1208		519
**antibody+2 peps/ 23+**		80	80	80	80	80	80	80	80	80	80	80	80	80		80
**antibody+2 peps/ 22+**		80	80	80	80	80	80	80	80	80	80	80	80	80		80
**antibody+2 peps/ 21+**		80	0	0	0	0	0	0	0	0	0	0	0	0		80

Table S2: continued

M7+P16, measurement 2

ion / charge state	m/z	ΔCV 2	6	10	14	18	22	26	30	34	40	46	52	58	64	0
**peptide 16/ 1+**		0	0	0	0	0	0	0	0	0	0	0	0	0		0
**peptide 16/ 2+**	919.53	339	1719	4092	3367	6376	8863	4307	10930	25760	21220	12860	12480	9298	**n.d.**	979
**peptide 16/ 3+**	613.36	2893	6437	23860	30300	81580	119800	166200	187700	230200	213400	76800	110900	63900	**n.d.**	3246
**peptide 16/ 4+**	460.52	203	300	2982	6333	12390	19010	25770	31230	46140	54120	35150	37870	33410	**n.d.**	237
**peptide 16/ 5+**		0	0	0	0	0	0	0	0	0	0	0	0	0		0
**antibody+0 pep/ 28+**																
**antibody+0 pep/ 27+**	5490.519	30	29	80	107	108	90	95	74	49	55	28	30	22	**n.d.**	26
**antibody+0 pep/ 26+**	5700.93	250	309	778	1067	1096	903	689	522	394	234	104	101	97	**n.d.**	183
**antibody+0 pep/ 25+**	5927.67	1300	1642	3824	4939	5555	4595	3764	2820	2055	1525	612	550	489	**n.d.**	1062
**antibody+0 pep/ 24+**	6174.236	3001	4109	8694	11080	13160	11560	9946	7997	6515	4744	2064	1894	1735	**n.d.**	2975
**antibody+0 pep/ 23+**	6442.638	3900	4358	8604	10740	13630	13260	12110	10330	9326	6994	3157	3060	2772	**n.d.**	3563
**antibody+0 pep/ 22+**	6736.476	1600	1899	3516	4367	6126	6646	6409	6060	5670	4746	2430	2426	2127	**n.d.**	1719
**antibody+0 pep/ 21+**	7058.484	392	425	694	809	1206	1534	1755	1830	2120	1915	1073	1081	968	**n.d.**	379
**antibody+0 pep/ 20+**		80	80	80	80	80	80	80	80	80	80	80	80	80		80
**antibody+0 pep/ 19+**		0	0	0	0	0	0	0	0	0	0	0	0	0		0
**antibody+1 pep/ 28+**		0	0	0	0	0	0	0	0	0	0	0	0	0		0
**antibody+1 pep/ 27+**	5557.359	101	136	411	522	561	389	283	199	133	94	48	48	41	**n.d.**	89
**antibody+1 pep/ 26+**	5770.57	856	1156	2821	3652	3945	2945	2364	1642	1027	636	283	232	248	**n.d.**	713
**antibody+1 pep/ 25+**	6000.76	3140	3899	8821	11130	12190	10140	8092	6027	4400	2857	1110	1083	871	**n.d.**	2620
**antibody+1 pep/ 24+**	6249.272	4891	5681	12050	15250	17860	16070	13950	10750	8640	5776	2473	2377	2133	**n.d.**	4529
**antibody+1 pep/ 23+**	6522.479	3210	3669	7141	9087	11590	11350	10480	8799	7921	5627	2413	2266	2076	**n.d.**	3289
**antibody+1 pep/ 22+**	6818.008	1200	1244	2203	2674	3719	3943	3916	3622	3594	3015	1482	1415	1241	**n.d.**	1241
**antibody+1 pep/ 21+**	7144.279	223	257	430	531	738	879	975	934	1116	992	524	539	528	**n.d.**	257
**antibody+1 pep/ 20+**		0	0	0	0	0	0	0	0	0	0	0	0	0		0
		0	0	0	0	0	0									0
**antibody+2 peps/ 28+**		80	80	80	80	80	80	0	0	0	0	0	0	0		80
**antibody+2 peps/ 27+**	5624.151	190	221	468	613	673	491	385	245	152	113	61	40	32	**n.d.**	154
**antibody+2 peps/ 26+**	5842.094	719	973	2157	2695	2904	2203	1672	1166	774	407	160	160	106	**n.d.**	613
**antibody+2 peps/ 25+**	6074.134	1602	1955	4170	5133	5767	4689	3605	2817	1967	1194	419	386	369	**n.d.**	1526
**antibody+2 peps/ 24+**	6327.046	1550	1664	3289	4226	5194	4661	3953	2867	2461	1524	612	581	494	**n.d.**	1414
**antibody+2 peps/ 23+**	6603.732	808	721	1331	1724	2258	2093	1945	1594	1584	1129	482	438	320	**n.d.**	806
**antibody+2 peps/ 22+**		80	80	80	80	80	80	80	80	80	80	80	80	80		80
**antibody+2 peps/ 21+**		0	0	0	0	0	0	80	80	80	80	80	80	80		0

Table S2: continued

M7+P17, measurement 1

ion / charge state	m/z	ΔCV 2	6	10	14	18	22	26	30	34	40	46	52	58	64	0
**peptide 17/ 1+**		0	0	0	0	0	0	0	0	0	0	0	0	0		
**peptide 17/ 2+**	935.388	20	26	60	48	164	203	221	325	486	664	566	333	293	**n.d.**	**n.d.**
**peptide 17/ 3+**	623.952	46	196	271	261	870	1141	1341	1897	3163	3890	3759	1604	1267	**n.d.**	**n.d.**
**peptide 17/ 4+**	468.218	20	30	41	35	103	120	159	270	472	648	699	566	579	**n.d.**	**n.d.**
**peptide 17/ 5+**		0	0	0	0	0	0	0	0	0	0	0	0	0		
**antibody+0 pep/ 29+**		0	0	0	0	0	0	0	0	0	0	0	0	0		
**antibody+0 pep/ 28+**		80	80	80	80	80	80	80	80	80	80	80	80	80		
**antibody+0 pep/ 27+**	5488.798	425	811	2024	237	1218	999	727	593	1043	1041	795	418	396	**n.d.**	**n.d.**
**antibody+0 pep/ 26+**	5701.299	1430	2424	5247	2334	3194	2697	1931	1795	2510	2732	2059	1118	1049	**n.d.**	**n.d.**
**antibody+0 pep/ 25+**	5926.252	1884	3071	6125	2726	3700	3147	2537	2530	3048	3214	2476	1388	1318	**n.d.**	**n.d.**
**antibody+0 pep/ 24+**	6173.473	1131	1723	3055	1400	1902	1769	1477	1636	1619	1681	1271	796	812	**n.d.**	**n.d.**
**antibody+0 pep/ 23+**	6442.812	270	449	721	365	498	504	453	494	515	516	412	295	309	**n.d.**	**n.d.**
**antibody+0 pep/ 22+**	6736.072	49	89	126	56	93	83	89	130	135	172	140	91	100	**n.d.**	**n.d.**
**antibody+0 pep/ 21+**	7058.108	54	107	321	136	180	149	113	81	145	171	146	89	76	**n.d.**	**n.d.**
**antibody+1 pep/ 29+**		0	0	0	0	0	0	0	0	0	0	0	0	0		
**antibody+1 pep/ 28+**		80	80	80	80	80	80	80	80	80	80	80	80	80		
**antibody+1 pep/ 27+**	5556.476	116	209	605	237	338	249	177	151	252	261	187	94	102	**n.d.**	**n.d.**
**antibody+1 pep/ 26+**	5765.978	602	1093	2505	882	1468	1237	876	769	1043	1118	801	390	383	**n.d.**	**n.d.**
**antibody+1 pep/ 25+**	6001.772	1252	2040	4139	1904	2526	2072	1552	1456	1770	1942	1268	688	627	**n.d.**	**n.d.**
**antibody+1 pep/ 24+**	6251.164	1048	1699	3084	1342	1919	1641	1391	1359	1455	1415	1035	555	518	**n.d.**	**n.d.**
**antibody+1 pep/ 23+**	6521.239	449	704	1125	504	752	685	625	593	582	606	426	260	250	**n.d.**	**n.d.**
**antibody+1 pep/ 22+**	6820.219	106	166	237	110	183	175	149	162	163	209	137	96	94	**n.d.**	**n.d.**
**antibody+1 pep/ 21+**		0	0	0	0	0	0	0	0	0	0	0	0	0		
**antibody+2 peps/ 29+**		0	0	0	0	0	0	0	0	0	0	0	0	0		
**antibody+2 peps/ 28+**		80	80	80	80	80	80	80	80	80	80	80	80	80		
**antibody+2 peps/ 27+**	5628.423	70	121	254	124	159	172	96	68	112	102	81	42	36	**n.d.**	**n.d.**
**antibody+2 peps/ 26+**	5845.034	204	354	702	337	431	352	258	221	300	294	199	97	69	**n.d.**	**n.d.**
**antibody+2 peps/ 25+**	6074.917	296	429	772	373	487	412	333	282	298	336	219	121	133	**n.d.**	**n.d.**
**antibody+2 peps/ 24+**	6329.411	156	258	440	209	288	264	215	206	205	216	133	72	68	**n.d.**	**n.d.**
**antibody+2 peps/ 23+**		80	80	80	80	80	80	80	80	80	80	80	80	80		
**antibody+2 peps/ 22+**		80	80	80	80	80	80	80	80	80	80	80	80	80		
**antibody+2 peps/ 21+**		0	0	0	0	0	0	0	0	0	0	0	0	0		

Table S2: continued

M7+P17, measurement 2

**ion / charge state**	**m/z**	**ΔCV**														
		**2**	**6**	**10**	**14**	**18**	**22**	**26**	**30**	**34**	**40**	**46**	**52**	**58**	**64**	**0**
**peptide 17/ 1+**		0	0	0	0	0	0	0	0	0	0	0	0	0		0
**peptide 17/ 2+**	935.365	100	62	226	145	324	749	1117	3262	1226	3576	2547	3202	3509	**n.d.**	100
**peptide 17/ 3+**	623.945	223	560	2622	1933	3324	5781	8182	21869	7225	17387	9913	10808	11198	**n.d.**	131
**peptide 17/ 4+**	468.214	75	152	500	404	618	998	1352	3476	1028	2380	1394	1540	1429	**n.d.**	39
**peptide 17/ 5+**		0	0	0	0	0	0	0	0	0	0	0	0	0		0
**antibody+0 pep / 28+**		0	0	0	0	0	0	0	0	0	0	0	0	0	0	
**antibody+0 pep / 27+**	5489.636	100	377	542	885	383	267	362	314	809	242	502	257	291	289	**n.d.**
**antibody+0 pep / 26+**	5699.928	899	2578	3431	5844	2477	1993	2287	2249	5252	1476	2883	1632	1854	1781	**n.d.**
**antibody+0 pep / 25+**	5998.826	2963	7547	9519	15887	6945	5592	6202	6017	13276	3692	7588	4039	4556	4804	**n.d.**
**antibody+0 pep / 24+**	6174.297	4576	10027	12090	20304	9124	7956	8008	7532	16096	4356	9216	5034	5636	6038	**n.d.**
**antibody+0 pep / 23+**	6442.589	3023	5649	6508	11049	5313	4930	4476	4368	8777	2357	5303	3016	3419	3581	**n.d.**
**antibody+0 pep / 22+**	6737.393	955	1520	1708	2821	1357	1454	1240	1315	2737	765	1678	1028	1199	1330	**n.d.**
**antibody+0 pep / 21+**	7059.38	173	255	278	708	243	244	260	292	618	184	479	313	388	418	**n.d.**
**antibody+0 pep / 20+**		0	0	0	0	0	0	0	0	0	0	0	0	0	0	
**antibody+1 pep/ 29+**		0	0	0	0	0	0	0	0	0	0	0	0	0	0	
**antibody+1 pep/ 28+**		80	80	80	80	80	80	80	80	80	80	80	80	80	80	
**antibody+1 pep/ 27+**	5557.199	253	712	966	1619	695ß	522	606	524	1306	361	650	336	362	347	**n.d.**
**antibody+1 pep/ 26+**	5771.919	1273	3375	4231	7226	3022	2349	2581	2377	5211	1427	2692	1366	1499	1443	**n.d.**
**antibody+1 pep/ 25+**	6001.441	2663	6250	7583	12655	65574	4495	4597	4185	8759	2359	3369	2364	2534	2579	**n.d.**
**antibody+1 pep/ 24+**	6251.146	2580	5302	6169	10244	4693	4161	3884	3666	7145	1874	3754	2012	2265	2281	**n.d.**
**antibody+1 pep/ 23+**	6525.049	1221	2034	2332	3931	1865	1832	1622	1556	3040	787	1584	896	1004	1061	**n.d.**
**antibody+1 pep/ 22+**	6823.669	331	516	528	947	411	465	432	600	809	245	492	286	349	339	**n.d.**
**antibody+1 pep/ 21+**		0	0	0	0	0	0	0	0	0	0	0	0	0	0	
**antibody+2 peps/ 29+**		0	0	0	0	0	0	0	0	0	0	0	0	0	0	
**antibody+2 peps/ 28+**		80	80	80	80	80	80	80	80	80	80	80	80	80	80	
**antibody+2 peps/ 27+**	5630.288	117	362	484	711	307	261	273	246	517	144	230	111	133	124	**n.d.**
**antibody+2 peps/ 26+**	5843.335	407	1009	1231	2028	895	674	698	624	1239	330	601	282	278	293	**n.d.**
**antibody+2 peps/ 25+**	6077.184	557	1271	1394	2371	1098	950	844	745	1379	385	705	346	366	347	**n.d.**
**antibody+2 peps/ 24+**	6330.331	407	753	808	1390	614	577	558	474	935	230	453	239	248	247	**n.d.**
**antibody+2 peps/ 23+**		80	80	80	80	80	80	80	80	80	80	80	80	80	80	
**antibody+2 peps/ 22+**		80	80	80	80	80	80	80	80	80	80	80	80	80	80	
**antibody+2 peps/ 21+**		0	0	0	0	0	0	0	0	0	0	0	0	0	0	

Table S2: continued

M11+P21, measurement 1

ion / charge state	m/z	ΔCV 2	6	10	14	18	22	26	30	34	40	46	52	58	0
**peptide 21/ 1+**		0	0	0	0	0	0	0	0	0	0	0	0	0	
**peptide 21/ 2+**	985.325	1532	33228	20505	20071	28694	18918	22812	34039	26701	40677	31841	28972	1532	**n.d.**
**peptide 21/ 3+**	657.33	976	9782	15376	34306	79499	92235	148782	249346	226938	401626	357212	321601	976	**n.d.**
**peptide 21/ 4+**	493.25	374	4079	7670	14233	33924	35488	61139	207658	100240	187682	184252	181040	374	**n.d.**
**peptide 21/ 5+**		0	0	0	0	0	0	0	0	0	0	0	0	0	
**antibody+0 pep / 28+**		0	0	0	0	0	0	0	0	0	0	0	0		
**antibody+0 pep / 27+**	5559.266	190	327	294	311	395	285	345	460	333	531	440	384	**n.d.**	**n.d.**
**antibody+0 pep / 26+**	5695.622	828	1521	1288	1398	1821	1297	1720	2196	1740	3007	2590	2233	**n.d.**	**n.d.**
**antibody+0 pep / 25+**	5922.859	4413	7433	6245	6565	8885	6283	7867	10430	8240	13721	11513	10043	**n.d.**	**n.d.**
**antibody+0 pep / 24+**	6169.979	8860	14505	12336	12641	17022	12241	15239	19729	16021	26190	21083	19622	**n.d.**	**n.d.**
**antibody+0 pep / 23+**	6438.132	7240	11743	9857	9869	13689	9685	11771	16153	12871	21992	17259	16098	**n.d.**	**n.d.**
**antibody+0 pep / 22+**	6732.429	2552	4125	3458	3652	5188	3911	4880	6824	5853	9573	8079	8029	**n.d.**	**n.d.**
**antibody+0 pep / 21+**	7053.589	466	776	709	732	1253	1075	1586	2409	2136	3961	3511	3702	**n.d.**	**n.d.**
**antibody+0 pep / 20+**	7405.367	80	93	88	80	170	168	300	573	624	1256	1166	1336	**n.d.**	**n.d.**
**antibody+1 pep/ 28+**		0	0	0	0	0	0	0	0	0	0	0	0		
**antibody+1 pep/ 27+**	5634.053	174	276	264	269	353	225	295	341	234	353	290	282	**n.d.**	**n.d.**
**antibody+1 pep/ 26+**	5771.388	1904	3149	2743	2846	2577	2506	3056	3812	2909	4352	3437	3017	**n.d.**	**n.d.**
**antibody+1 pep/ 25+**	6002.08	6375	10808	8787	8812	11715	7967	9159	11712	9194	13881	10323	8947	**n.d.**	**n.d.**
**antibody+1 pep/ 24+**	6252.143	8280	14025	11652	11396	15471	10446	12140	15668	12064	18942	14295	129622	**n.d.**	**n.d.**
**antibody+1 pep/ 23+**	6524.149	4721	7768	6623	6521	8976	6264	7399	10033	7680	11750	9191	9026	**n.d.**	**n.d.**
**antibody+1 pep/ 22+**	6822.471	1476	2372	2067	2051	2992	2196	2724	3693	3090	5025	4154	2052	**n.d.**	**n.d.**
**antibody+1 pep/ 21+**	7193.476	80	467	411	103	652	513	731	1142	1037	1726	1488	1654	**n.d.**	**n.d.**
**antibody+1 pep/ 20+**	7497.79	0	0	0	0	0	0	0	213	211	457	401	489	**n.d.**	**n.d.**
**antibody+2 peps/ 29+**		0	0	0	0	0	0	0	0	0	0	0	0		
**antibody+2 peps/ 28+**	5484.424	80	89	80	121	145	121	146	172	130	253	204	198	**n.d.**	**n.d.**
**antibody+2 peps/ 27+**		80	80	80	80	80	80	80	80	80	80	80	80		
**antibody+2 peps/ 26+**	5846.876	1228	1956	1699	1618	2154	1424	1680	1991	1416	1955	1412	1180	**n.d.**	**n.d.**
**antibody+2 peps/ 25+**	6080.27	2659	4310	3606	3478	4426	3058	3327	4116	3089	4339	3068	2592	**n.d.**	**n.d.**
**antibody+2 peps/ 24+**	6333.895	2454	3819	3232	3162	4157	2856	3128	3903	2843	4343	3201	2854	**n.d.**	**n.d.**
**antibody+2 peps/ 23+**	6611.645	1156	1756	1472	1426	2077	1387	1626	2037	1616	2424	1777	1704	**n.d.**	**n.d.**
**antibody+2 peps/ 22+**		80	80	80	80	80	80	80	80	80	80	80	80		
**antibody+2 peps/ 21+**		0	0	0	0	0	0	0	0	0	0	0	0		
**antibody+2 peps/ 20+**															

Table S2: continued

M11+P21, measurement 2

ion / charge state	m/z	ΔCV														
		2	6	10	14	18	22	26	30	34	40	46	52	58		0
**peptide 21/ 1+**		0	0	0	0	0	0	0	0	0	0	0	0	0		0
**peptide 21/ 2+**	983.483	2775	15949	19398	19091	23714	18499	20432	29835	34100	29411	30039	43205	37655	**n.d.**	364
**peptide 21/ 3+**	657.33	3317	12194	26708	52897	101648	119493	159664	261854	338234	311279	343180	460919	354861	**n.d.**	1637
**peptide 21/ 4+**	493.25	2189	5778	11713	22155	39613	47728	64393	107639	146393	140365	167076	250877	209810	**n.d.**	1048
**Peptide 21/ 5+**		0	0	0	0	0	0	0	0	0	0	0	0	0		0
**antibody+0 pep/ 28+**		0	0	0	0	0	0	0	0	0	0	0	0	0		0
**antibody+0 pep/ 27+**	5558.549	269	482	513	492	577	396	346	419	395	342	303	412	294	**n.d.**	80
**antibody+0 pep/ 26+**	5696.425	1220	2133	2057	2146	1661	1940	1935	2207	2425	1888	1888	2764	2073	**n.d.**	293
**antibody+0 pep/ 25+**	5922.994	5643	9067	9278	9577	11833	9011	8824	10836	11792	9267	9422	13143	10943	**n.d.**	1994
**antibody+0 pep/ 24+**	6169.928	10419	16271	16711	17493	21636	16447	16628	21304	23851	19424	19289	27365	23433	**n.d.**	5227
**antibody+0 pep/ 23+**	6438.807	7986	11799	12315	13153	16885	12737	12893	16888	19110	16274	17406	24129	21506	**n.d.**	4996
**antibody+0 pep/ 22+**	6733.305	2632	4002	4171	4680	6173	4893	5204	6976	8383	7663	8176	12125	11070	**n.d.**	2056
**antibody+0 pep/ 21+**	7054.246	494	756	823	944	1379	1343	1655	2497	3214	3149	3523	5622	5085	**n.d.**	423
**antibody+0 pep/ 20+**	7405.35	80	92	109	126	190	219	312	587	933	1011	1180	1839	1858	**n.d.**	72
**antibody+1 pep/ 28+**		0	0	0	0	0	0	0	0	0	0	0	0	0		0
**antibody+1 pep/ 27+**	5632.174	248	413	419	435	477	338	317	319	317	208	218	277	202	**n.d.**	80
**antibody+1 pep/ 26+**	5772.152	2509	4333	4175	4305	5248	3668	3395	4036	3944	2745	2655	3592	2854	**n.d.**	715
**antibody+1 pep/ 25+**	6002.501	7754	12333	12340	12571	15215	10778	10330	12512	13090	9744	9216	12298	9954	**n.d.**	3191
**antibody+1 pep/ 24+**	6252.794	9458	14242	14726	15448	18971	13665	13197	16444	17901	14258	13593	18387	15347	**n.d.**	4990
**antibody+1 pep/ 23+**	6525.2	5111	7442	7823	8406	10590	7957	8060	10210	11459	9369	9462	13138	11466	**n.d.**	3258
**antibody+1 pep/ 22+**	6823.417	1546	2170	2327	2637	3366	2679	2873	3944	4511	4019	4255	5957	5497	**n.d.**	1155
**antibody+1 pep/ 21+**	7146.946	302	373	469	498	700	673	775	1152	1520	1406	1505	2392	2305	**n.d.**	253
**antibody+1 pep/ 20+**	7502.548	0	0	75	0	0	0	121	211	314	357	446	688	667	**n.d.**	46
**antibody+2 peps/ 29+**		0	0	0	0	0	0	0	0	0	0	0	0	0		0
**antibody+2 peps/ 28+**	5485.453	80	80	80	80	80	80	80	80	80	80	80	80	80		80
**antibody+2 peps/ 28+**	5485.453	101	159	166	169	238	151	158	163	182	149	170	221	162	**n.d.**	**n.d.**
**antibody+2 peps/ 27+**		80	80	80	80	80	80	80	80	80	80	80	80	80		80
**antibody+2 peps/ 26+**	5848.933	1546	2404	1501	2435	2744	1895	1830	2059	1977	1293	1118	1466	1186	**n.d.**	486
**antibody+2 peps/ 25+**	6077.936	3062	4649	4722	4850	5664	3815	3596	4239	4397	3162	2861	3652	2875	**n.d.**	1428
**antibody+2 peps/ 24+**	6335.19	2543	3791	3916	4164	5062	3556	3348	3982	4268	3311	3182	4086	3307	**n.d.**	1471
**antibody+2 peps/ 23+**	6611.171	1155	1582	1710	1842	2288	1745	1739	2144	2380	1887	1840	2501	2212	**n.d.**	752
**antibody+2 peps/ 22+**	6911.074	346	444	508	583	697	522	599	777	730	790	827	1119	1023	**n.d.**	248
**antibody+2 peps/ 21+**		0	0	0	0	0	0	0	0	0	0	0	0	0		0
**antibody+2 peps/ 20+**																

Table S2: continued

M11+P22, measurement 1

ion / charge state	m/z	ΔCV 2	6	10	14	18	22	26	30	34	40	46	52	58	64	0
**peptide 22/ 1+**		0	0	0	0	0	0	0	0	0	0	0	0	0		
**peptide 22/ 2+**	984.996	400	400	431	664	1292	1776	2626	2500	1959	2898	9007	4291	6339	**n.d.**	1039
**peptide 22/ 3+**	657.001	947	2845	7976	12680	23684	51009	50316	47877	47462	70929	218540	81942	125275	**n.d.**	**n.d.**
**peptide 22/ 4+**	493.001	400	1755	4116	6114	10489	23278	24144	20090	21512	36928	99882	48129	74032	**n.d.**	**n.d.**
**Peptide 22/ 5+**		0	0	0	0	0	0	0	0	0	0	0	0	0		
**antibody+0 pep/ 28+**		0	0	0	0	0	0	0	0	0	0	0	0	0		0
**antibody+0 pep/ 27+**	5484.598	99	303	492	373	460	807	649	450	338	417	417	993	432		80
**antibody+0 pep/ 26+**	5694.696	1192	3912	6710	5280	6334	9838	8346	6067	4288	4966	5441	12548	4966	**n.d.**	837
**antibody+0 pep/ 25+**	5922.679	6601	19488	32260	24304	30038	44280	37413	27404	21031	23503	25459	56269	23354	**n.d.**	3871
**antibody+0 pep/ 24+**	6169.805	14362	39121	64707	48092	58552	83884	70755	51181	41129	45952	50377	107217	44509	**n.d.**	7702
**antibody+0 pep/ 23+**	6438.634	11640	29076	48997	35668	42489	60022	49349	35665	29962	33665	36700	79781	32383	**n.d.**	5998
**antibody+0 pep/ 22+**	6732.172	3869	8738	14834	10723	12734	17784	14331	10349	9145	11018	11185	25732	10333	**n.d.**	1929
**antibody+0 pep/ 21+**	7053.861	624	1351	2280	1750	2094	2981	2480	1836	1711	2606	2216	5526	2453		80
**antibody+0 pep/ 20+**		0	0	0	0	0	0	0	0	0	0	0	0	0		0
**antibody+1 pep/ 28+**		0	0	0	0	0	0	0	0	0	0	0	0	0		0
**antibody+1 pep/ 27+**	5558.692	60	180	242	227	282	426	325	231	154	196	196	348	80	**n.d.**	80
**antibody+1 pep/ 26+**	5771.105	502	1506	2385	1816	2090	3118	2717	1875	1425	1115	1469	2522	1151	**n.d.**	297
**antibody+1 pep/ 25+**	6001.652	1779	5010	7970	5885	6857	9741	7985	5793	4356	3665	4977	8423	3505	**n.d.**	872
**antibody+1 pep/ 24+**	6251.666	2671	6581	10772	7853	9235	12954	10488	7446	5904	5194	6778	12243	5122	**n.d.**	1213
**antibody+1 pep/ 23+**	6523.967	1646	3614	5904	4279	4946	6883	5398	4017	3313	3212	3759	7303	3142	**n.d.**	671
**antibody+1 pep/ 22+**	6822.246	471	1001	1567	1136	1343	1892	1535	1073	968	1073	1083	2317	1002	**n.d.**	200
**antibody+1 pep/ 21+**	7147.366	91	195	292	197	245	341	280	233	205	317	289	589	282	**n.d.**	80
**antibody+1 pep/ 20+**	7408.989	66	125	80	168	196	318	298	258	271	570	398	1031	517	**n.d.**	56

Table S2: continued

M11+P22, measurement 2

**ion / charge state**	**m/z**	**ΔCV**														
		**2**	**6**	**10**	**14**	**18**	**22**	**26**	**30**	**34**	**40**	**46**	**52**	**58**	**64**	**0**
**peptide 22/ 1+**		0	0	0	0	0	0	0	0	0	0	0	0	0		0
**peptide 22/ 2+**	984.989	501	2678	2594	4413	10421	15964	21170	23302	22461	41630	46840	51747	64612	**n.d.**	400
**peptide 22/ 3+**	656.992	1413	4084	5420	10318	21137	33750	48151	52502	50918	81645	91084	85331	88191	**n.d.**	825
**peptide 22/ 4+**	492.998	400	400	400	593	764	1377	1858	1971	1533	3047	4354	4178	5814	**n.d.**	400
**peptide 22/ 5+**		0	0	0	0	0	0	0	0	0	0	0	0	0		0
**antibody+0 pep/ 28+**		0	0	0	0	0	0	0	0	0	0	0	0	0		0
**antibody+0 pep/ 27+**	5484.417	118	643	428	493	691	831	886	992	576	709	563	589	609	**n.d.**	80
**antibody+0 pep/ 26+**	5695.776	1533	85	4898	5189	7470	8992	9461	10538	6110	7803	6755	6247	7051	**n.d.**	517
**antibody+0 pep/ 25+**	5922.911	7620	28422	19777	20890	29347	35082	37333	41221	24853	32528	28482	26085	31129	**n.d.**	3248
**antibody+0 pep/ 24+**	6169.88	14885	48209	32520	34082	48329	57064	60248	66494	42311	56816	52276	46137	56119	**n.d.**	8137
**antibody+0 pep/ 23+**	6438.96	10386	29229	19767	21023	29513	34568	36315	39960	26070	36461	35596	30930	37857	**n.d.**	6918
**antibody+0 pep/ 22+**	6732.371	3079	7398	5122	5521	7689	8990	9575	10549	7189	10292	10811	9492	11943	**n.d.**	2419
**antibody+0 pep/ 21+**	7053.482	521	1051	759	835	1204	1445	1692	1857	1323	2124	2413	2320	2986	**n.d.**	445
**antibody+0 pep/ 20+**	7408.585	0	0	0	0	0	0	0	0	0	0	0	457	679	**n.d.**	49
**antibody+1 pep/ 28+**		0	0	0	0	0	0	0	0	0	0	0	0	0		0
**antibody+1 pep/ 27+**	5561.205	80	332	252	240	343	340	412	469	243	276	217	225	212	**n.d.**	80
**antibody+1 pep/ 26+**	5771.169	626	2396	1662	1640	2338	2813	2808	3122	1687	1957	1485	1281	1538	**n.d.**	246
**antibody+1 pep/ 25+**	6001.696	1864	6590	4472	4712	6324	7350	7501	8287	7405	5860	4799	3941	4469	**n.d.**	964
**antibody+1 pep/ 24+**	6252.206	2437	7545	5099	5276	7300	8388	8386	9282	5657	7541	6434	5237	6004	**n.d.**	1406
**antibody+1 pep/ 23+**	6523.761	1245	3291	2289	2444	3364	3786	3920	4306	2637	3787	3479	2885	3424	**n.d.**	867
**antibody+1 pep/ 22+**	6821.586	394	812	553	585	841	983	1032	1138	789	1040	1059	912	1189	**n.d.**	277
**antibody+1 pep/ 21+**	7145.229	80	80	80	116	157	180	195	210	80	263	299	256	386	**n.d.**	80
**antibody+1 pep/ 20+**		0	0	0	0	0	0	0	0	0	0	0	0	0		0

Table S2: continued

M11+P23, measurement 1

ion / charge state	m/z	ΔCV 2	6	10	14	18	22	26	30	34	40	46	52	58	64	0
**peptide 23/ 1+**		0	0	0	0	0	0	0	0	0	0	0	0			
**peptide 23/ 2+**	983.058	400	1913	2615	2262	1821	3102	1435	1332	1516	3616	2493	3159	2768	**n.d.**	**n.d.**
**peptide 23/ 3+**	655.792	2634	7764	13755	14751	17767	26760	15094	16192	20145	25544	22721	24523	18141	**n.d.**	**n.d.**
**peptide 23/ 4+**	492.035	1296	3590	7121	5792	7962	19209	6595	6927	9141	15059	15626	18520	19162	**n.d.**	**n.d.**
**peptide 23/ 5+**		0	0	0	0	0	0	0	0	0	0	0	0	400	0	0
**Peptide 23/ 6+**														0		
**antibody+0 pep/ 27+**		0	0	0	0	0	0	0	0	0	0	0	0	0		0
**antibody+0 pep/ 26+**	5684.175	1805	7476	5818	3381	2492	4018	1452	1063	1159	735	697	856	749	**n.d.**	113
**antibody+0 pep/ 25+**	5911.605	12541	49533	41973	23088	18011	27380	9268	7639	8247	5400	4587	5718	5455	**n.d.**	819
**antibody+0 pep/ 24+**	6158.406	38046	146260	128771	65534	51615	78340	24181	21083	22076	15742	13573	15868	15806	**n.d.**	2801
**antibody+0 pep/ 23+**	6426.589	44930	158349	150984	71846	57709	85298	24473	22550	23574	17535	15164	18643	18209	**n.d.**	3477
**antibody+0 pep/ 22+**	6719.582	21891	70165	67670	30210	34967	36387	9566	9162	9387	8238	7552	9378	9611	**n.d.**	1704
**antibody+0 pep/ 21+**	7039.503	4351	13858	13124	5670	4875	7378	2416	2210	2232	2612	2242	3289	3225	**n.d.**	427
**antibody+0 pep/ 20+**	7393.987	464	1328	1304	586	561	925	2100	378	479	80	796	1030	1127	**n.d.**	62
**antibody+0 pep/ 19+**		0	0	0	0	0	0	0	0	0	0	0	0	0		0
**antibody+1 pep/ 28+**		0	0	0	0	0	0	0	0	0	0	0	0	0		0
**antibody+1 pep/ 27+**		80	80	80	80	80	80	80	80	80	80	80	80	80		80
**antibody+1 pep/ 26+**	5760.453	2056	8852	6833	3995	2809	4101	1475	1151	1173	595	483	599	502	**n.d.**	105
**antibody+1 pep/ 25+**	5983.934	10059	40159	34203	17712	13248	19796	6120	5387	5377	2944	2178	2576	2475	**n.d.**	656
**antibody+1 pep/ 24+**	6240.281	19905	73453	66819	32985	25515	37067	10883	9412	9664	5550	4531	5149	5019	**n.d.**	1425
**antibody+1 pep/ 23+**	6512.431	16766	57171	52548	24515	19135	27954	7407	6771	7136	4662	3987	4707	4510	**n.d.**	1155
**antibody+1 pep/ 22+**	6815.751	6470	20452	18593	8354	6613	9559	313	2337	2436	2004	1694	2193	2203	**n.d.**	458
**antibody+1 pep/ 21+**	7484.63	1399	3903	3608	1571	1221	1882	495	498	552	632	564	759	735	**n.d.**	123
**antibody+1 pep/ 20+**		0	0	0	0	0	0	0	0	0	0	0	0	0		0
**antibody+2 peps/ 28+**		0	0	0	0	0	0	0	0	0	0	0	0	0		0
**antibody+2 peps/ 27+**		80	80	80	80	80	80	80	80	80	80	80	80	80		80
**antibody+2 peps/ 26+**	5838.141	787	3414	2583	1414	977	1471	460	363	360	80	80	80	80	**n.d.**	80
**antibody+2 peps/ 25+**	6069.634	2667	10276	8721	4288	3129	4459	1286	1082	1156	513	365	401	394	**n.d.**	80
**antibody+2 peps/ 24+**	6322.703	3920	13228	11501	5667	4073	5893	1544	1322	1412	755	553	691	590	**n.d.**	234
**antibody+2 peps/ 23+**	6597.231	2451	8000	7078	3243	2355	3484	862	786	783	538	456	544	515	**n.d.**	168
**antibody+2 peps/ 22+**	6900.539	955	2836	2454	1142	795	1167	956	300	300	247	214	304	253	**n.d.**	80
**antibody+2 peps/ 21+**		80	80	80	80	80	80	80	80	80	80	80	80	80		80
**antibody+2 peps/ 20+**		0	0	0	0	0	0	0	0	0	0	0	0	0		0

Table S2: continued

M11+P23, measurement 2

ion / charge state	m/z	ΔCV														
		2	6	10	14	18	22	26	30	34	40	46	52	58	64	0
**peptide 23/ 0.5+**		0	0	0												
**peptide 23/ 1+**		0	400	400	0	0	0	0	0	0	0	0	0			
**peptide 23/ 2+**	982.985	623	3171	3553	2048	1733	1937	2175	2150	3339	3045	2060	2674	2598	**n.d.**	**n.d.**
**peptide 23/ 3+**	655.719	956	2539	2798	3980	4507	7657	14475	15068	20664	27836	17004	26044	15639	**n.d.**	**n.d.**
**peptide 23/ 4+**	492.099	606	765	1552	1579	2494	3547	6859	7040	11649	17636	14213	21755	19055	**n.d.**	**n.d.**
**peptide 23/ 5+**		0			0	0	0	0	0	0	0	0	0	400		
**peptide 23/ 6+**														0		
**antibody+0 pep/ 28+**																
**antibody+0 pep/ 27+**		0	0	0	0	0	0	0	0	0	0	0	0	0		0
**antibody+0 pep/ 26+**	5684.621	769	1406	880	623	542	602	799	707	955	1086	802	1299	1022	**n.d.**	64
**antibody+0 pep/ 25+**	5911.427	5112	8476	5386	3873	3207	4038	5238	4456	5974	6934	4582	7558	6214	**n.d.**	611
**antibody+0 pep/ 24+**	6158.467	12934	21021	13761	9677	7815	9776	12279	10888	14709	17516	11643	19187	15921	**n.d.**	2228
**antibody+0 pep/ 23+**	6426.746	13524	21384	13611	9500	7511	9282	11410	10248	13872	17246	11767	20209	16617	**n.d.**	2768
**antibody+0 pep/ 22+**	6720.401	5785	8704	5382	3843	3035	3812	4791	4322	5931	7446	5300	9271	7965	**n.d.**	1365
**antibody+0 pep/ 21+**	7040.781	1223	1786	1055	755	633	914	1234	1290	1828	2410	1755	3132	2794	**n.d.**	342
**antibody+0 pep/ 20+**	7396.935	135	190	105	82	83	129	241	80	436	688	531	1014	921	**n.d.**	48
**antibody+0 pep / 19+**		0	0	0	0	0	0	0	0	0	0	0	0	0		0
**antibody+1 pep/ 28+**		0	0	0	0	0	0	0	0	0	0	0	0	0		0
**antibody+1 pep/ 27+**		80	80	80	80	80	80	80	80	80	80	80	80	80		80
**antibody+1 pep/ 26+**	5761.311	887	1440	867	583	519	613	731	598	746	781	541	799	682	**n.d.**	80
**antibody+1 pep/ 25+**	5990.963	1003	1303	741	498	398	444	536	433	517	547	339	472	386	**n.d.**	80
**antibody+1 pep/ 24+**	6240.648	6431	9905	6063	4122	3174	3953	4621	3793	5196	5878	3668	5952	4799	**n.d.**	1155
**antibody+1 pep/ 23+**	6513.155	4744	6953	4023	2760	2229	2728	3244	2652	3670	4387	2840	4827	3831	**n.d.**	895
**antibody+1 pep/ 22+**	6808.696	1745	2497	1307	1010	745	926	1191	1081	1473	1792	1179	2111	1740	**n.d.**	375
**antibody+1 pep/ 21+**	7136.03	367	468	281	212	165	208	300	294	415	521	400	687	615	**n.d.**	93
**antibody+1 pep/ 20+**		0	0	0	0	0	0	0	0	0	0	0	0	0		0
**antibody+2 peps/ 28+**		0	0	0	0	0	0	0	0	0	0	0	0	0		
**antibody+2 peps/ 27+**		80	80	80	80	80	80	80	80	80	80	80	80	80		
**antibody+2 peps/ 26+**	5842.565	369	489	252	172	80	80	80	80	80	80	80	80	80	**n.d.**	**n.d.**
**antibody+2 peps/ 25+**	6069.314	1003	1303	741	498	398	444	536	433	517	547	339	472	386	**n.d.**	**n.d.**
**antibody+2 peps/ 24+**	6321.771	1256	1544	889	607	447	534	563	524	656	755	448	702	553	**n.d.**	**n.d.**
**antibody+2 peps/ 23+**	6599.641	790	931	455	332	227	337	305	305	394	480	338	518	413	**n.d.**	**n.d.**
**antibody+2 peps/ 22+**	6908.773	295	318	180	125	80	80	80	80	80	80	80	80	80	**n.d.**	**n.d.**
**antibody+2 peps/ 21+**		80	80	80	80	80	80	80	80	80	80	80	80	80		
**antibody+2 peps/ 20+**		0	0	0	0	0	0	0	0	0	0	0	0	0		

Table S2: continued

M11+P24, measurement 1

ion / charge state	m/z	ΔCV 2	6	10	14	18	22	26	30	34	40	46	52	58	64	0
**peptide 24/ 0.5+**			0	0	0	0	0									
**peptide 24/ 1+**			400	400	400	400	400	0	0							
**peptide 24/ 2+**	997.539	763	2210	4094	3895	2984	4269	4449	5454	4263	3014	2377	1081	875	**n.d.**	**n.d.**
**peptide 24/ 3+**	665.348	**n.d.**	367	740	2046	1929	3658	5556	9244	11134	9704	10303	6293	4070	**n.d.**	**n.d.**
**peptide 24/ 4+**	499.26	**n.d.**	0	702	1344	1847	3067	5373	10699	12283	13131	16460	15047	8226	**n.d.**	**n.d.**
**peptide 24/ 5+**								0	400	400	400	400	400	400		
**peptide 24/ 6+**									0	0	0	0	0	0		
**antibody+0 pep/ 28+**		0	0	0	0	0	0	0	0	0	0	0	0	0		0
**antibody+0 pep/ 27+**	5473.653	80	364	506	386	371	299	333	363	281	80	80	80	80	**n.d.**	80
**antibody+0 pep/ 26+**	5682.947	4365	8363	6179	6104	4060	2356	1727	1461	1671	1366	1414	1760	1465	**n.d.**	164
**antibody+0 pep/ 25+**	5910.767	24969	42485	31783	31264	20280	12848	10085	8384	10434	8045	8109	100032	8545	**n.d.**	1481
**antibody+0 pep/ 24+**	6157.352	56465	90835	67722	67046	43838	28861	23065	19075	24717	19982	19386	23983	20542	**n.d.**	4340
**antibody+0 pep/ 23+**	6425.702	53064	80472	60221	60245	38874	26697	20814	17103	22451	18497	18483	22901	20049	**n.d.**	4726
**antibody+0 pep/ 22+**	6717.642	20833	30343	23027	23290	14654	10271	7919	6392	8844	7135	7131	9055	8250	**n.d.**	2093
**antibody+0 pep/ 21+**	7038.088	4041	5713	4333	4516	2843	1959	1544	1325	1781	1554	1495	1942	1744	**n.d.**	443
**antibody+0 pep/ 20+**	7390.573	80	695	528	558	316	223	193	163	243	215	259	324	309	**n.d.**	58
**antibody+1 pep/ 28+**		0	0	0	0	0	0	0	0	0	0	0	0	0		0
**antibody+1 pep/ 27+**		80	80	80	80	80	80	80	80	80	80	80	80	80		80
**antibody+1 pep/ 26+**	5760.208	1326	1328	2180	1704	1343	1495	1583	1922	1428	1048	1074	1006	592	**n.d.**	98
**antibody+1 pep/ 25+**	5990.321	3584	3939	7205	6069	4475	5393	5893	7344	5724	4634	4948	4316	2805	**n.d.**	461
**antibody+1 pep/ 24+**	6240.796	4661	5381	11228	10476	7021	9287	10068	13059	10600	9490	10303	8580	5375	**n.d.**	799
**antibody+1 pep/ 23+**	6512.77	2787	3434	8076	8257	5141	7079	7045	10950	8808	8027	9532	7915	4826	**n.d.**	670
**antibody+1 pep/ 22+**	6808.577	972	1132	2881	3120	1881	2552	2974	4045	3582	3305	3394	3277	2148	**n.d.**	300
**antibody+1 pep/ 21+**	7132.333	80	114	636	686	434	628	720	893	843	841	1004	888	602	**n.d.**	89
**antibody+1 pep/ 20+**		0	0	0	0	0	0	0	0	0	0	0	0	0		0

Table S2: continued

M11+P24, measurement 2

ion / charge state	m/z	ΔCV 2	6	10	14	18	22	26	30	34	40	46	52	58	64	0
**peptide 24/ 0.5+**			0	0	0	0	0	0								
**peptide 24/ 1+**			400	400	400	400	400	400								
**peptide 24/ 2+**	999.5	**n.d.**	7047	12723	11135	10546	9253	7233	5269	6582	3523	1735	995	970	**n.d.**	**n.d.**
**peptide 24/ 3+**	666.67	**n.d.**	1108	2082	3628	4743	6727	5575	5914	11329	9974	7712	7309	4416	**n.d.**	**n.d.**
**peptide 24/ 4+**	500.26	**n.d.**	1013	1487	3166	2626	4690	5484	6085	11398	10890	12909	16890	14542	**n.d.**	**n.d.**
**peptide 24/ 5+**						0	0		400	400	400	400	400	400		
**peptide 24/ 6+**									0	0	0	0	0	0		
**antibody+0 pep/ 28+**		0	0	0	0	0	0	0	0	0	0	0	0	0		0
**antibody+0 pep/ 27+**	5482.722	80	464	382	399	264	131	103	102	114	91	85	118	98	**n.d.**	0
**antibody+0 pep/ 26+**	5693.92	4365	8363	6179	6104	4060	2356	1727	1461	1671	1366	1414	1760	1465	**n.d.**	164
**antibody+0 pep/ 25+**	5922.328	24969	42485	31783	31264	20280	12848	10085	8384	10434	8045	8109	100032	8545	**n.d.**	1481
**antibody+0 pep/ 24+**	6168.696	56465	90835	67722	67046	43838	28861	23065	19075	24717	19982	19386	23983	20542	**n.d.**	4340
**antibody+0 pep/ 23+**	6437.841	53064	80472	60221	60245	38874	26697	20814	17103	22451	18497	18483	22901	20049	**n.d.**	4726
**antibody+0 pep/ 22+**	6730.276	20833	30343	23027	23290	14654	10271	7919	6392	8844	7135	7131	9055	8250	**n.d.**	2093
**antibody+0 pep/ 21+**	7053.124	4041	5713	4333	4516	2843	1959	1544	1325	1781	1554	1495	1942	1744	**n.d.**	443
**antibody+0 pep/ 20+**	7405.272	80	695	528	558	316	223	193	163	243	215	259	324	309	**n.d.**	58
**antibody+1 pep/ 28+**		0	0	0	0	0	0	0	0	0	0	0	0	0		0
**antibody+1 pep/ 27+**		80	80	80	80	80	80	80	80	80	80	80	80	80		80
**antibody+1 pep/ 26+**	5842.668	1145	2053	1578	1520	983	623	481	369	442	312	349	438	332	**n.d.**	74
**antibody+1 pep/ 25+**	6002.336	4008	6462	5131	5034	3346	2209	1718	1392	1712	1366	1306	1479	1295	**n.d.**	325
**antibody+1 pep/ 24+**	6252.907	5958	9323	7523	7515	5071	2502	2767	2112	2731	2210	2142	2536	2218	**n.d.**	585
**antibody+1 pep/ 23+**	6524.756	4146	6021	5074	5100	3311	2372	1864	1552	1954	1588	1503	1861	1665	**n.d.**	432
**antibody+1 pep/ 22+**	6822.381	1422	2036	1714	1760	1176	872	653	508	689	591	588	712	638	**n.d.**	172
**antibody+1 pep/ 21+**	7147.156	80	466	377	397	257	192	148	114	169	140	151	180	180	**n.d.**	47
**antibody+1 pep/ 20+**		0	0	0	0	0	0	0	0	0	0	0	0	0		0
**antibody+2 peps/ 28+**		**n.d.**	**n.d.**	**n.d.**	**n.d.**	**n.d.**	**n.d.**	**n.d.**	**n.d.**	**n.d.**	**n.d.**	**n.d.**	**n.d.**	**n.d.**	**n.d.**	**n.d.**
**antibody+2 peps/ 27+**		**n.d.**	**n.d.**	**n.d.**	**n.d.**	**n.d.**	**n.d.**	**n.d.**	**n.d.**	**n.d.**	**n.d.**	**n.d.**	**n.d.**	**n.d.**	**n.d.**	**n.d.**
**antibody+2 peps/ 26+**		**n.d.**	**n.d.**	**n.d.**	**n.d.**	**n.d.**	**n.d.**	**n.d.**	**n.d.**	**n.d.**	**n.d.**	**n.d.**	**n.d.**	**n.d.**	**n.d.**	**n.d.**
**antibody+2 peps/ 25+**		**n.d.**	**n.d.**	**n.d.**	**n.d.**	**n.d.**	**n.d.**	**n.d.**	**n.d.**	**n.d.**	**n.d.**	**n.d.**	**n.d.**	**n.d.**	**n.d.**	**n.d.**
**antibody+2 peps/ 24+**		**n.d.**	**n.d.**	**n.d.**	**n.d.**	**n.d.**	**n.d.**	**n.d.**	**n.d.**	**n.d.**	**n.d.**	**n.d.**	**n.d.**	**n.d.**	**n.d.**	**n.d.**
**antibody+2 peps/ 23+**		**n.d.**	**n.d.**	**n.d.**	**n.d.**	**n.d.**	**n.d.**	**n.d.**	**n.d.**	**n.d.**	**n.d.**	**n.d.**	**n.d.**	**n.d.**	**n.d.**	**n.d.**
**antibody+2 peps/ 22+**		**n.d.**	**n.d.**	**n.d.**	**n.d.**	**n.d.**	**n.d.**	**n.d.**	**n.d.**	**n.d.**	**n.d.**	**n.d.**	**n.d.**	**n.d.**	**n.d.**	**n.d.**
**antibody+2 peps/ 21+**		**n.d.**	**n.d.**	**n.d.**	**n.d.**	**n.d.**	**n.d.**	**n.d.**	**n.d.**	**n.d.**	**n.d.**	**n.d.**	**n.d.**	**n.d.**	**n.d.**	**n.d.**
**antibody+2 peps/ 20+**		**n.d.**	**n.d.**	**n.d.**	**n.d.**	**n.d.**	**n.d.**	**n.d.**	**n.d.**	**n.d.**	**n.d.**	**n.d.**	**n.d.**	**n.d.**	**n.d.**	**n.d.**

Table S2: continued

M11+P25, measurement 1

ion / charge state	m/z	ΔCV 2	6	10	14	18	22	26	30	34	40	46	52	58	64	0
**Peptide 25/ 0.5+**			0	0	0	0	0	0	0	0						
**peptide 25/ 1+**			400	400	400	400	400	400	400	400	0	0	0	0		
**peptide 25/ 2+**	958.912	**n.d.**	3175	20367	3539	27349	18080	17434	16168	21401	17391	10390	11181	9275	**n.d.**	**n.d.**
**peptide 25/ 3+**	639.635	**n.d.**	294	2015	4752	5676	7332	10787	11262	20208	17439	16219	17433	28955	**n.d.**	**n.d.**
**peptide 25/ 4+**	479.95	**n.d.**	0	0	0	0	0	258	646	650	318	523	1120	708	**n.d.**	**n.d.**
**peptide 25/ 5+**											0	0	0	0		
**antibody+0 pep/ 28+**																
**antibody+0 pep/ 27+**		0	0	0	0	0	0	0	0	0	0	0	0			0
**antibody+0 pep/ 26+**	5773.316	364	587	833	635	434	480	361	730	760	984	1627	2544	**n.d.**	**n.d.**	140
**antibody+0 pep/ 25+**	5921.287	3137	4353	6159	5489	3688	3960	3166	6011	5880	984	11034	15814	**n.d.**	**n.d.**	1523
**antibody+0 pep/ 24+**	6168.012	9343	11742	16666	16828	10996	12024	9410	17130	17165	20878	30173	40456	**n.d.**	**n.d.**	5109
**antibody+0 pep/ 23+**	6434.982	10119	11949	16708	18915	11944	13248	10638	18902	19104	22731	32531	40611	**n.d.**	**n.d.**	6219
**antibody+0 pep/ 22+**	6729.586	4285	4826	6724	8723	5181	5052	4839	8631	8746	10019	14141	17996	**n.d.**	**n.d.**	3016
**antibody+0 pep/ 21+**	7051.392	759	838	1240	1807	1059	1238	1096	1997	2027	2349	3598	4492	**n.d.**	**n.d.**	714
**antibody+0 pep/ 20+**	7407.709	57	67	93	154	110	172	157	296	321	435	637	833	**n.d.**	**n.d.**	93
		0	0	0	0	0	0	0	0	0	0	0	0			0
**antibody+1 pep/ 28+**		0	0	0	0	0	0	0	0	0	0	0	0			0
**antibody+1 pep/ 27+**		80	80	80	80	80	80	80	80	80	80	80	80			80
**antibody+1 pep/ 26+**	5770.848	208	297	435	314	204	218	170	315	313	367	556	656	**n.d.**	**n.d.**	80
**antibody+1 pep/ 25+**	5998.911	1113	1532	2090	1727	1095	1178	928	1537	1643	1722	2473	3242	**n.d.**	**n.d.**	536
**antibody+1 pep/ 24+**	6248.328	2270	2743	3883	3664	2311	2514	1958	3176	3188	3648	4944	6046	**n.d.**	**n.d.**	1168
**antibody+1 pep/ 23+**	6520.954	1836	2065	1884	3108	1826	2142	1646	2924	2696	3017	4277	5043	**n.d.**	**n.d.**	1000
**antibody+1 pep/ 22+**	6817.185	689	765	1022	1223	766	799	670	1159	1130	2101	1786	2148	**n.d.**	**n.d.**	426
**antibody+1 pep/ 21+**	7143.509	144	162	228	306	165	197	159	281	321	314	464	607	**n.d.**	**n.d.**	120
**antibody+1 pep/ 20+**		0	0	0	0	0	0	0	0	0	0	0	0			0
**antibody+2 peps/ 28+**		0	0	0	0	0	0	0	0	0	0	0	0			0
**antibody+2 peps/ 27+**		80	80	80	80	80	80	80	80	80	80	80	80			20
**antibody+2 peps/ 26+**		80	80	80	80	80	80	80	80	80	80	80	80			20
**antibody+2 peps/ 25+**	6075.952	273	327	439	386	248	269	196	322	328	361	508	639	**n.d.**	**n.d.**	20
**antibody+2 peps/ 24+**	6327.609	395	433	604	593	399	414	344	531	545	650	837	1041	**n.d.**	**n.d.**	222
**antibody+2 peps/ 23+**	6603.944	285	286	437	459	277	302	266	448	406	458	660	794	**n.d.**	**n.d.**	190
**antibody+2 peps/ 22+**	6905.05	112	117	144	182	125	126	113	195	180	204	291	363	**n.d.**	**n.d.**	69
**antibody+2 peps/ 21+**		80	80	80	80	80	80	80	80	80	80	80	80			20
**antibody+2 peps/ 20+**		0	0	0	0	0	0	0	0	0	0	0	0			0

Table S2: continued

M11+P25, measurement 2

ion / charge state	m/z	ΔCV														
		2	6	10	14	18	22	26	30	34	40	46	52	58	64	0
**peptide 25/ 0.5+**		0	0	0	0	0	0	0	0							
**peptide 25/ 1+**		400	400	400	400	400	400	400	400	0	0	0	0			
**peptide 25/ 2+**	958.783	1598	7496	4630	22109	13998	15913	12724	17941	18349	18302	20906	25278	**n.d.**	**n.d.**	**n.d.**
**peptide 25/ 3+**	639.527	322	491	737	3766	4372	6774	8542	17730	20562	30680	41165	39707	**n.d.**	**n.d.**	**n.d.**
**peptide 25/ 4+**	479.977	0	0	0	0	0	0	321	635	519	797	1227	1925	**n.d.**	**n.d.**	**n.d.**
**peptide 25/ 5+**										0	0	0	0			
**antibody+0 pep/ 27+**		0	0	0	0	0	0	0	0	0	0	0	0			0
**antibody+0 pep/ 26+**	5693.203	932	1481	1903	1653	1058	953	884	1150	598	516	670	919	**n.d.**	**n.d.**	80
**antibody+0 pep/ 25+**	5922.549	6985	9792	13359	11515	7485	6381	6506	8365	4640	4108	5014	6083	**n.d.**	**n.d.**	343
**antibody+0 pep/ 24+**	6168.879	19289	25860	34896	30081	19087	17272	17996	22219	13473	12406	14771	17211	**n.d.**	**n.d.**	1411
**antibody+0 pep/ 23+**	6437.42	20066	26531	36472	30941	19492	17875	19509	23029	14666	13860	17872	19526	**n.d.**	**n.d.**	2165
**antibody+0 pep/ 22+**	6729.675	8664	11166	15915	13311	8396	8183	8671	10099	6852	6384	8371	9487	**n.d.**	**n.d.**	1320
**antibody+0 pep/ 21+**	7051.229	1800	2318	3254	2821	1817	1773	2100	2347	1648	1589	2162	2493	**n.d.**	**n.d.**	328
**antibody+0 pep/ 20+**	7404.479	223	279	433	369	228	268	311	345	279	284	401	471	**n.d.**	**n.d.**	65
**antibody+0 pep/ 19+**		0	0	0	0	0	0	0	0	0	0	0	0			0
**antibody+1 pep/ 28+**		0	0	0	0	0	0	0	0	0	0	0	0			0
**antibody+1 pep/ 27+**		80	80	80	80	80	80	80	80	80	80	80	80			80
**antibody+1 pep/ 26+**	5769.84	405	592	812	677	416	336	344	436	237	218	248	268	**n.d.**	**n.d.**	80
**antibody+1 pep/ 25+**	6000.389	2055	2895	3903	3187	2048	1676	1692	2158	1206	1085	1129	1343	**n.d.**	**n.d.**	155
**antibody+1 pep/ 24+**	6248.58	3918	5235	7305	6079	3689	3453	3503	3950	2458	2195	2645	2791	**n.d.**	**n.d.**	431
**antibody+1 pep/ 23+**	6521.165	3146	4126	5718	4738	2930	2795	2981	3391	2180	1959	2441	2655	**n.d.**	**n.d.**	417
**antibody+1 pep/ 22+**	6817.783	1201	1562	2193	1777	1089	1149	1103	1301	894	858	1091	1187	**n.d.**	**n.d.**	225
**antibody+1 pep/ 21+**	7144.231	265	339	479	369	273	265	280	336	314	224	303	326	**n.d.**	**n.d.**	68
**antibody+1 pep/ 20+**		0	0	0	0	0	0	0	0	0	0	0	0			0
**antibody+2 peps/ 28+**		0	0	0	0	0	0	0	0	0	0	0	0			0
**antibody+2 peps/ 27+**		80	80	80	80	80	80	80	80	80	80	80	80			80
**antibody+2 peps/ 26+**		80	80	80	80	80	80	80	80	80	80	80	80			80
**antibody+2 peps/ 25+**	6076.013	467	582	824	722	421	385	399	440	278	258	284	311	**n.d.**	**n.d.**	**n.d.**
**antibody+2 peps/ 24+**	6330.218	687	909	1244	1068	628	579	634	692	437	427	486	535	**n.d.**	**n.d.**	91
**antibody+2 peps/ 23+**	6606.228	475	633	860	757	448	463	456	521	356	319	424	457	**n.d.**	**n.d.**	99
**antibody+2 peps/ 22+**	6907.479	204	230	340	276	175	164	191	243	166	149	195	204	**n.d.**	**n.d.**	40
**antibody+2 peps/ 21+**		80	80	80	80	80	80	80	80	80	80	80	80			80
**antibody+2 peps/ 20+**		0	0	0	0	0	0	0	0	0	0	0	0			0

Table S2: continued

M11+P26, measurement 1

ion / charge state	m/z	ΔCV 2	6	10	14	18	22	26	30	34	40	46	52	58	64	0
**peptide 26/ 0.5+**		0	0	0	0	0	0	0								
**peptide 26/ 1+**		100	400	400	400	400	400	400	0	0	0	0	0			
**peptide 26/ 2+**	634.52	406	2236	4842	3430	3768	3323	3862	3835	4367	4982	2751	2457	**n.d.**	**n.d.**	**n.d.**
**peptide 26/ 3+**	951.235	48	121	472	714	1518	1982	3144	4057	5597	7774	4950	5105	**n.d.**	**n.d.**	**n.d.**
**peptide 26/ 4+**		0	0	0	0	0	0	0	400	400	400	400	400			
**peptide 26/ 5+**									0	0	0	0	0			
**antibody+0 pep/ 27+**		0	0	0	0	0	0	0	0	0	0	0	0			0
**antibody+0 pep/ 26+**	5695.102	194	254	502	442	504	441	454	468	539	669	472	613	**n.d.**	**n.d.**	194
**antibody+0 pep/ 25+**	5922.482	2078	2700	5163	4328	4964	4344	4810	4955	5444	6486	4000	4920	**n.d.**	**n.d.**	2078
**antibody+0 pep/ 24+**	6168.772	8039	9587	18575	14410	16729	14876	16362	16740	19293	23289	14636	16027	**n.d.**	**n.d.**	8039
**antibody+0 pep/ 23+**	6437.734	11624	13240	25133	18727	21582	19274	21611	21947	25758	30342	20135	22282	**n.d.**	**n.d.**	11624
**antibody+0 pep/ 22+**	6730.645	6611	7094	13267	9409	10925	9739	11085	11709	13477	16133	11133	11903	**n.d.**	**n.d.**	6611
**antibody+0 pep/ 21+**	7051.861	1459	1643	3052	2109	2554	2356	2801	2946	3458	4352	2951	3383	**n.d.**	**n.d.**	1459
**antibody+0 pep/ 20+**	7406.581	80	166	318	239	291	311	412	431	630	819	604	674	**n.d.**	**n.d.**	80
**antibody+0 pep/ 19+**		0	0	0	0	0	0	0	0	0	0	0	0			0
**antibody+1 pep/ 28+**		0	0	0	0	0	0	0	0	0	0	0	0			0
**antibody+1 pep/ 27+**		80	80	80	80	80	80	80	80	80	80	80	80			80
**antibody+1 pep/ 26+**	5768.44	133	185	375	311	311	281	297	275	314	352	243	273	**n.d.**	**n.d.**	80
**antibody+1 pep/ 25+**	5999.04	1036	1284	2534	1925	2188	1782	2012	2014	2193	2377	1444	1593	**n.d.**	**n.d.**	233
**antibody+1 pep/ 24+**	6248.852	2781	3300	6308	4502	5149	4420	4811	4758	5151	6007	3739	3878	**n.d.**	**n.d.**	715
**antibody+1 pep/ 23+**	6520.805	2909	3219	6024	4340	4994	4305	4738	4641	5352	6058	3921	4207	**n.d.**	**n.d.**	833
**antibody+1 pep/ 22+**	6817.795	1351	1471	2660	1881	2120	1891	2092	2193	2494	2806	1950	2168	**n.d.**	**n.d.**	422
**antibody+1 pep/ 21+**	7144.076	305	347	623	408	486	465	528	551	664	768	531	614	**n.d.**	**n.d.**	109
**antibody+1 pep/ 20+**		0	0	0	0	0	0	0	0	0	0	0	0			0

Table S2: continued

M11+P26, measurement 2

ion / charge state	m/z	ΔCV														
		2	6	10	14	18	22	26	30	34	40	46	52	58	64	0
**peptide 26/ 0.5+**		0	0	0	0	0										
**peptide 26/ 1+**		100	400	400	400	400	0	0	0	0	0	0	0			
**peptide 26/ 2+**	951.32	227	1197	3223	2859	3216	4437	2262	3744	4509	3954	4046	4061	**n.d.**	**n.d.**	**n.d.**
**peptide 26/ 3+**	634.578	113	268	786	1212	2408	6688	3075	6304	8711	8654	10463	10506	**n.d.**	**n.d.**	**n.d.**
**peptide 26/ 4+**		0	0	0	0	0	400	400	400	400	400	400	400			
**Peptide 26/ 5+**							0	0	0	0	0	0	0			
**antibody+0 pep / 27+**		0	0	0	0	0	0	0	0	0	0	0	0			
**antibody+0 pep / 26+**	5695.536	407	1935	2685	2141	2611	4830	1972	2991	3503	2683	3827	4010	**n.d.**	**n.d.**	**n.d.**
**antibody+0 pep / 25+**	5922.504	3169	13835	20092	16078	18939	30664	12152	6402	21039	16323	10041	19760	**n.d.**	**n.d.**	**n.d.**
**antibody+0 pep / 24+**	6169.638	9638	38460	57659	47258	53516	75842	29725	11051	49232	38682	42709	40800	**n.d.**	**n.d.**	**n.d.**
**antibody+0 pep / 23+**	6438.562	3034	40576	62720	51063	56093	73118	26039	38772	41690	34057	34454	32827	**n.d.**	**n.d.**	**n.d.**
**antibody+0 pep / 22+**	6732.48	11177	15712	25225	20530	22549	29011	9384	14324	15027	13598	12030	12074	**n.d.**	**n.d.**	**n.d.**
**antibody+0 pep / 21+**	7053.495	4443	2692	4696	3765	4254	5357	1738	2887	2990	2658	2634	2749	**n.d.**	**n.d.**	**n.d.**
**antibody+0 pep / 20+**		80	80	80	80	80	80	80	80	80	80	80	80			
**antibody+0 pep / 19+**		0	0	0	0	0	0	0	0	0	0	0	0			
**antibody+1 pep/ 28+**		0	0	0	0	0	0	0	0	0	0	0	0			
**antibody+1 pep/ 27+**		80	80	80	80	80	80	80	80	80	80	80	80			
**antibody+1 pep/ 26+**	5769.297	263	1159	1675	1314	1512	2638	1042	1449	1548	1170	1347	1365	**n.d.**	**n.d.**	**n.d.**
**antibody+1 pep/ 25+**	5999.99	1381	5737	8382	6724	7427	11853	4384	6402	6659	4959	5431	5076	**n.d.**	**n.d.**	**n.d.**
**antibody+1 pep/ 24+**	6250.056	3034	11433	16896	13556	14956	21924	7615	11051	11217	8621	8895	8284	**n.d.**	**n.d.**	**n.d.**
**antibody+1 pep/ 23+**	6523.02	2417	8666	13540	10580	11517	16887	5127	7656	7434	6125	5884	5464	**n.d.**	**n.d.**	**n.d.**
**antibody+1 pep/ 22+**	6820.098	917	2938	4455	3751	4091	5712	1746	2704	2577	2086	1966	1890	**n.d.**	**n.d.**	**n.d.**
**antibody+1 pep/ 21+**	7144.721	171	549	868	736	746	1077	334	524	542	470	443	498	**n.d.**	**n.d.**	**n.d.**
**antibody+1 pep/ 20+**		0	0	0	0	0	0	0	0	0	0	0	0			

Table S2: continued

M11+P27, measurement 1

ion / charge state	m/z	ΔCV 2	6	10	14	18	22	26	30	34	40	46	52	58	64	0
**peptide 27/ 0.5+**		0	0	0	0	0										
**peptide 27/ 1+**		400	400	400	400	400	0	0	0	0	0	0	0	0		0
**peptide 27/ 2+**	975.964	4890	13494	10942	9421	14000	14987	15936	22123	21226	23679	24197	26223	400	**n.d.**	100
**peptide 27/ 3+**	650.979	1661	2603	4171	6284	11557	17789	27350	37818	40358	48292	54181	50850	1661	**n.d.**	398
**peptide 27/ 4+**	488.523	0	0	0	0	0	990	768	791	2437	2499	3303	3871	400	**n.d.**	100
**Peptide 27/ 5+**							0	0	0	0	0	0	0	0		0
**antibody+0 pep/ 27+**		80	80	80	80	80	80	80	80	80	80	80	80			80
**antibody+0 pep/ 26+**	5695.167	3024	4173	1553	1105	1208	1096	1235	1474	1257	1261	1421	1470	**n.d.**	**n.d.**	80
**antibody+0 pep/ 25+**	5924.113	15852	21493	9376	7542	8467	5921	9257	10846	9713	9703	10418	10395	**n.d.**	**n.d.**	957
**antibody+0 pep/ 24+**	6178.354	32335	44296	22722	19793	22455	22777	25568	29600	27334	28399	28278	30279	**n.d.**	**n.d.**	3925
**antibody+0 pep/ 23+**	8649.385	26852	37428	21823	20336	23302	24712	26309	30801	28686	32214	34703	35164	**n.d.**	**n.d.**	5585
**antibody+0 pep/ 22+**	6732.504	9655	13775	8821	8686	10150	10835	11130	13590	12914	14935	16014	16991	**n.d.**	**n.d.**	2975
**antibody+0 pep/ 21+**	7055.414	1791	2533	1809	1860	2115	2483	2559	3053	2983	3675	3959	4432	**n.d.**	**n.d.**	748
**antibody+0 pep/ 20+**	7409.126	169	263	80	80	251	296	80	447	468	630	764	804	**n.d.**	**n.d.**	0
**antibody+0 pep/ 19+**		0	0	0	0	0	0	0	0	0	0	0	0			0
**antibody+1 pep/ 28+**		0	0	0	0	0	0	0	0	0	0	0	0			0
**antibody+1 pep/ 27+**		80	80	80	80	80	80	80	80	80	80	80	80			80
**antibody+1 pep/ 26+**	5771.22	1377	1937	683	557	580	495	586	641	539	477	532	519	**n.d.**	**n.d.**	80
**antibody+1 pep/ 25+**	6003.224	4969	6884	3026	2451	2710	2609	2844	3204	2762	2771	2705	2540	**n.d.**	**n.d.**	447
**antibody+1 pep/ 24+**	6253.045	7728	10568	5294	4866	5418	5224	5655	6293	5641	5923	5962	5931	**n.d.**	**n.d.**	1105
**antibody+1 pep/ 23+**	6525.979	5101	7171	4011	3750	4286	4580	4465	5268	4814	5482	5413	5742	**n.d.**	**n.d.**	1141
**antibody+1 pep/ 22+**	6823.373	1666	2551	1477	1491	1629	1821	1822	2086	1981	2315	2380	2554	**n.d.**	**n.d.**	508
**antibody+1 pep/ 21+**	7146.515	80	489	305	314	363	427	429	519	458	561	638	717	**n.d.**	**n.d.**	151
**antibody+1 pep/ 20+**		0	0	0	0	0	0	0	0	0	0	0	0			0

Table S2: continued

M11+P27, measurement 2

ion / charge state	m/z	ΔCV 2	6	10	14	18	22	26	30	34	40	46	52	58	0
**peptide 27/ 0.5+**		0	0	0	0	0									
**peptide 27/ 1+**		400	400	400	400	400	0	0	0	0	0	0	0		
**peptide 27/ 2+**	975.96	3940	17542	22723	29680	23473	11095	24800	29052	30895	22573	24310	22071	5152	**n.d.**
**peptide 27/ 3+**	650.977	2133	3224	6202	13753	17407	13656	36590	45639	53691	51019	55190	43487	34502	**n.d.**
**peptide 27/ 4+**	488.484	0	0	0	0	0	400	1172	1608	2304	3158	2502	3431	44205	**n.d.**
**peptide 27/ 5+**							0	0	0	0	0	0	0	400	
**peptide 27/ 6+**														0	
**antibody+0 pep/ 27+**		0	0	0	0	0	0	0	0	0	0	0	0		0
**antibody+0 pep/ 26+**	5695.417	1670	2335	1944	2098	1514	1433	1662	1690	1816	1552	1614	1475	**n.d.**	80
**antibody+0 pep/ 25+**	5924.046	11607	16177	13470	15144	10749	10394	11539	12377	12855	10806	10801	9832	**n.d.**	1046
**antibody+0 pep/ 24+**	6170.465	31881	43650	34924	41830	30062	27595	31098	33459	35709	29846	29438	27958	**n.d.**	4611
**antibody+0 pep/ 23+**	6439.437	35118	46883	37283	46805	34141	29694	33463	35510	38418	31984	32259	32591	**n.d.**	7053
**antibody+0 pep/ 22+**	6733.197	15960	21528	16586	21738	15869	13665	15293	16354	17567	14798	14490	16125	**n.d.**	4242
**antibody+0 pep/ 21+**	7054.464	3578	4608	3578	4797	3672	3086	3553	3899	4305	3581	3681	4203	**n.d.**	1228
**antibody+0 pep/ 20+**		80	80	80	80	80	80	80	80	80	80	80	80		80
**antibody+0 pep/ 19+**		0	0	0	0	0	0	0	0	0	0	0	0		0
**antibody+1 pep/ 28+**		0	0	0	0	0	0	0	0	0	0	0	0		0
**antibody+1 pep/ 27+**		80	80	80	80	80	80	80	80	80	80	80	80		80
**antibody+1 pep/ 26+**	5771.672	780	1108	898	965	665	616	679	666	706	555	533	513	**n.d.**	80
**antibody+1 pep/ 25+**	6002.719	3678	5261	4220	5762	3311	2917	3426	3383	3510	2842	2703	2442	**n.d.**	472
**antibody+1 pep/ 24+**	6253.01	7529	10517	8120	9814	7083	6121	6646	7014	7101	5833	5602	5357	**n.d.**	1356
**antibody+1 pep/ 23+**	6525.495	6413	8963	6812	8514	6169	5359	5749	5970	6144	5079	4941	5176	**n.d.**	1479
**antibody+1 pep/ 22+**	6822.834	2543	3617	2719	3522	2628	2181	2369	2540	2721	2215	2178	2329	**n.d.**	745
**antibody+1 pep/ 21+**	7148.695	587	770	786	793	590	497	568	627	643	557	577	720	**n.d.**	197
**antibody+1 pep/ 20+**		0	0	0	0	0	0	0	0	0	0	0	0		0

Table S2: continued

M11+P28, measurement 1

ion / charge state	m/z	ΔCV													
		2	6	10	14	18	22	26	30	34	40	46	52	58	0
**peptide/ 0.5+**		0	0	0	0	0	0	0	0	0	0	0	0		
**peptide 28/ 1+**		400	400	400	400	400	400	400	400	400	400	400	400		
**peptide 28/ 2+**	1000.5	1338	12740	17980	23746	24140	27820	28580	39776	32450	30180	18020	16780	**n.d.**	**n.d.**
**peptide 28/ 3+**	667.67	401	747	2385	4365	13300	21800	18140	47170	40600	48370	17790	18400	**n.d.**	**n.d.**
**peptide 28/ 4+**	500.33	0	0	0	0	0	364	688	1218	1330	1366	1768	1500	**n.d.**	**n.d.**
**peptide 28/ 5+**															
**antibody+0 pep/ 28+**		0	0	0	0	0	0	0	0	0	0				0
**antibody+0 pep/ 27+**		80	80	80	80	80	80	80	80	80	80	0	0		80
**antibody+0 pep/ 26+**	5694.749	1711	4807	6730	6706	6106	6361	5936	6827	5023	4951	3070	3177	**n.d.**	540
**antibody+0 pep/ 25+**	5922.282	10980	29840	39490	38450	36030	37840	34930	41410	31940	31150	20060	21700	**n.d.**	4268
**antibody+0 pep/ 24+**	6169.391	27010	72050	89050	86330	80050	84550	78570	96830	76500	78160	51380	56990	**n.d.**	12950
**antibody+0 pep/ 23+**	6438.202	25320	66740	80590	76030	70600	75830	70600	89770	72720	77730	52910	61000	**n.d.**	12220
**antibody+0 pep/ 22+**	6731.838	9434	24680	28650	27090	25410	27650	26650	34300	28340	31110	21800	26850	**n.d.**	5618
**antibody+0 pep/ 21+**	7053.354	1637	4195	4916	4835	4643	5245	5015	6815	5767	6839	4948	6263	**n.d.**	1081
**antibody+0 pep/ 20+**		0	0	0	0	0	0	0	0	0	0	80	80		0
**antibody+0 pep/ 19+**												0	0		
**antibody+1 pep/ 28+**		0	0	0	0	0	0	0	0	0	0	0	0		0
**antibody+1 pep/ 27+**		80	80	80	80	80	80	80	80	80	80	80	80		80
**antibody+1 pep/ 26+**		80	80	80	80	80	80	80	80	80	80	80	80		80
**antibody+1 pep/ 25+**	6004.09	2140	5632	7059	7062	6329	6531	5988	7143	5300	5160	3106	3341	**n.d.**	899
**antibody+1 pep/ 24+**	6254.218	3954	10280	12540	12290	11180	11410	10630	12880	10120	10110	6457	7009	**n.d.**	1968
**antibody+1 pep/ 23+**	6526.554	2897	7466	8961	8601	8079	8474	7849	9687	7787	8231	5515	6405	**n.d.**	1638
**antibody+1 pep/ 22+**	6824.277	928	2455	2855	2832	2678	2868	2668	3487	2940	3121	2073	2573	**n.d.**	640
**antibody+1 pep/ 21+**		80	80	80	80	80	80	80	80	80	80	80	80		80
**antibody+1 pep/ 20+**		0	0	0	0	0	0	0	0	0	0	0	0		0

Table S2: continued

M11+P28, measurement 2

ion / charge state	m/z	ΔCV 2	6	10	14	18	22	26	30	34	40	46	52	58	64	0
**peptide 28/ 0.5+**		0	0	0	0	0	0	0	0	0	0	0	0			0
**peptide 28/ 1+**		400	400	400	400	400	400	400	400	400	400	400	400			100
**peptide 28/ 2+**	1000.5	16780	15950	22940	41796	36240	35465	36190	28510	29564	14120	7712	148	**n.d.**	**n.d.**	194
**peptide 28/ 3+**	667.67	1840	1720	2945	8356	5966	9100	8610	12550	9161	11210	11050	11250	**n.d.**	**n.d.**	81
**peptide 28/ 4+**	500.33	0	0	0	0	146	222	0	205	0	459	561	898	**n.d.**	**n.d.**	0
**peptide 28/ 5+**																
**antibody+0 pep/ 28+**									0	0	0	0	0			
**antibody+0 pep/ 27+**		0	0	0	0	0	0	0	80	80	80	80	80			0
**antibody+0 pep/ 26+**	5694.678	1457	3661	4651	6142	2501	2686	1760	1360	981	1124	1193	1522	**n.d.**	**n.d.**	154
**antibody+0 pep/ 25+**	5921.944	9724	22920	28200	36470	15050	16840	11110	8870	6728	7053	7210	8497	**n.d.**	**n.d.**	1267
**antibody+0 pep/ 24+**	6168.669	24900	56710	70130	87260	36760	42360	28510	22580	17110	18590	18070	20710	**n.d.**	**n.d.**	4029
**antibody+0 pep/ 23+**	6436.664	26270	58990	71880	87680	36820	43420	28970	22340	16490	17750	17270	20070	**n.d.**	**n.d.**	4239
**antibody+0 pep/ 22+**	6730.169	11220	24720	29950	36220	15160	18420	12130	9145	6521	6963	6773	8114	**n.d.**	**n.d.**	1840
**antibody+0 pep/ 21+**	7051.425	2417	5151	6160	7451	3245	4030	2615	2020	1407	1552	1512	1854	**n.d.**	**n.d.**	446
**antibody+0 pep/ 20+**		80	80	80	80	80	80	80	0	0	0	0	0			80
**antibody+0 pep/ 19+**		0	0	0	0	0	0	0								0
**antibody+1 pep/ 28+**		0	0	0	0	0	0	0	0	0	0	0	0			0
**antibody+1 pep/ 27+**		80	80	80	80	80	80	80	80	80	80	80	80			80
**antibody+1 pep/ 26+**		80	80	80	80	80	80	80	80	80	80	80	80			80
**antibody+1 pep/ 25+**	6002.572	1970	4562	5566	7668	3450	3778	2385	1786	1266	1255	1224	1436	**n.d.**	**n.d.**	337
**antibody+1 pep/ 24+**	6253.674	3843	8671	10780	14640	6744	7818	4756	3446	2427	2552	2384	2752	**n.d.**	**n.d.**	754
**antibody+1 pep/ 23+**	6525.699	3234	7137	8574	11440	5462	6376	3963	2945	1913	1953	1928	2181	**n.d.**	**n.d.**	581
**antibody+1 pep/ 22+**	6823.509	1197	2584	3166	4101	2028	2529	1508	1070	717	728	729	863	**n.d.**	**n.d.**	230
**antibody+1 pep/ 21+**		80	80	80	80	80	80	80	80	80	80	80	80			80
**antibody+1 pep/ 20+**		0	0	0	0	0	0	0	0	0	0	0	0			0

Table S2: continued

M11+P29, measurement 1

ion / charge state	m/z	ΔCV														
		2	6	10	14	18	22	26	30	34	40	46	52	58	64	0
**peptide 29/ 0.5+**		0	0	0	0	0	0	0	0	0	0	0	0	0		
**peptide 29/ 1+**		400	400	400	400	400	400	400	400	400	400	400	400	400		
**peptide 29/ 2+**	971.49	4881	28893	28352	30282	20715	14273	16852	13993	13957	14464	7805	7086	7906	**n.d.**	**n.d.**
**peptide 29/ 3+**	647.999	270	1604	2567	4141	5473	4743	7811	11778	13550	22767	17933	14610	16023	**n.d.**	**n.d.**
**peptide 29/ 4+**	486.247	0	0	0	185	555	1046	1126	1001	1198	185	555	1046	1126	**n.d.**	**n.d.**
**antibody+0 pep/ 28+**		0	0	0	0	0	0	0	0	0	0	0	0	0		0
**antibody+0 pep/ 27+**		80	80	80	80	80	80	80	80	80	80	80	80	80		80
**antibody+0 pep/ 26+**	5696.969	501	1819	1195	1196	753	475	573	597	516	715	573	668	610	**n.d.**	64
**antibody+0 pep/ 25+**	5924.363	3854	10604	7326	7536	4959	3246	4034	3992	3929	4794	3879	3984	3820	**n.d.**	525
**antibody+0 pep/ 24+**	6170.216	11077	25287	17925	19018	12713	8532	10270	10381	10346	13076	10215	10296	10124	**n.d.**	1699
**antibody+0 pep/ 23+**	6439.271	11919	24855	17797	19448	13148	8689	10022	10382	10307	13297	10636	10571	10203	**n.d.**	2011
**antibody+0 pep/ 22+**	6733.109	5252	10494	7566	8396	5753	3678	4146	4295	4239	5733	4466	4478	3334	**n.d.**	928
**antibody+0 pep/ 21+**	7054.979	1094	2269	1620	1877	1239	785	934	940	989	1372	1068	1115	1094	**n.d.**	242
**antibody+0 pep/ 20+**	7407.943	114	283	262	245	201	170	135	154	158	244	208	226	211	**n.d.**	39
**antibody+1 pep/ 28+**		0	0	0	0	0	0	0	0	0	0	0	0	0		0
**antibody+1 pep/ 27+**		80	80	80	80	80	80	80	80	80	80	80	80	80		80
**antibody+1 pep/ 26+**	5772.156	245	880	543	514	291	189	223	235	216	230	184	201	190	**n.d.**	80
**antibody+1 pep/ 25+**	5994.77	1282	3796	2431	2469	1435	897	1089	1027	1028	1209	872	927	940	**n.d.**	193
**antibody+1 pep/ 24+**	6252.598	2700	6943	4545	4750	2855	1799	2137	2042	1880	2403	1812	1787	1740	**n.d.**	402
**antibody+1 pep/ 23+**	6524.721	2349	5619	3719	3974	2333	1493	1663	1680	1544	2038	1520	1523	1514	**n.d.**	377
**antibody+1 pep/ 22+**	6822.594	933	2208	1415	1487	952	541	641	633	630	780	603	650	626	**n.d.**	148
**antibody+1 pep/ 21+**	7150.253	83	460	307	345	220	129	140	171	153	211	171	174	160	**n.d.**	0
**antibody+1 pep/ 20+**		0	0	0	0	0	0	0	0	0	0	0	0	0		0

Table S2: continued

M11+P29, measurement 2

ion / charge state	m/z	ΔCV 2	6	10	14	18	22	26	30	34	40	46	52	58	64	0
**peptide 29/ 0.5+**		0	0	0	0	0	0	0	0	0	0	0	0	0		
**peptide 29/ 1+**		400	400	400	400	400	400	400	400	400	400	400	400	400		
**peptide 29/ 2+**	971.5	3999	17532	28460	25038	23800	26730	25567	26624	21987	17763	13748	12507	11861	**n.d.**	**n.d.**
**peptide 29/ 3+**	648	361	781	3234	9836	7731	15138	21358	21407	24175	28577	31271	31707	27944	**n.d.**	**n.d.**
**peptide 29/ 4+**	486.25	0	0	0	0	0	0	632	633	758	926	1123	1189	1333	**n.d.**	**n.d.**
**antibody+0 pep/ 28+**																
**antibody+0 pep/ 27+**		0	0	0	0	0	0	0	0	0	0	0	0	0		0
**antibody+0 pep/ 26+**	5695.049	447	632	831	743	729	720	732	756	696	651	730	895	786	**n.d.**	92
**antibody+0 pep/ 25+**	5922.939	4149	5338	6879	5950	5741	6257	6417	6392	6288	5884	5972	6594	6383	**n.d.**	1113
**antibody+0 pep/ 24+**	6169.603	14437	17205	21494	18352	17793	19045	19173	19768	19438	18187	18514	19946	19384	**n.d.**	4642
**antibody+0 pep/ 23+**	6438.422	19257	21491	26150	22372	21475	22862	23085	24489	22966	21578	21477	24323	23412	**n.d.**	6681
**antibody+0 pep/ 22+**	6731.567	10151	10614	12988	11080	10593	11239	11293	12126	11320	19524	10815	12023	11931	**n.d.**	3835
**antibody+0 pep/ 21+**	7051.692	2451	2408	2895	2518	2439	2681	2785	2967	2677	2671	2803	3088	3028	**n.d.**	1015
**antibody+0 pep/ 20+**	7407.209	274	291	373	342	321	405	411	402	458	470	516	567	584	**n.d.**	156
**antibody+0 pep/ 19+**		0	0	0	0	0	0	0	0	0	0	0	0	0		0
**antibody+1 pep/ 28+**		0	0	0	0	0	0	0	0	0	0	0	0	0		0
**antibody+1 pep/ 27+**		80	80	80	80	80	80	80	80	80	80	80	80	80		80
**antibody+1 pep/ 26+**	5772.254	202	288	342	290	268	280	263	275	258	254	256	260	259	**n.d.**	80
**antibody+1 pep/ 25+**	6001.519	1327	1641	1989	1653	1560	1546	1605	1610	1491	1371	1426	1372	1346	**n.d.**	416
**antibody+1 pep/ 24+**	6252.661	3348	3688	4654	3928	3839	3790	3705	3787	3595	3278	3226	3471	3228	**n.d.**	1116
**antibody+1 pep/ 23+**	6523.899	3500	3581	4411	3711	3574	3715	3519	3798	3430	3319	3204	3320	3253	**n.d.**	1160
**antibody+1 pep/ 22+**	6820.606	1590	1490	1919	1599	1576	1641	1582	1731	1520	1437	1381	1454	1560	**n.d.**	572
**antibody+1 pep/ 21+**	7147.797	420	392	389	385	371	377	404	428	382	336	382	426	444	**n.d.**	149
**antibody+1 pep/ 20+**		0	0	0	0	0	0	0	0	0	0	0	0	0		0

Table S2: continued

M11+P30, measurement 1

ion / charge state	m/z	ΔCV 2	6	10	14	18	22	26	30	34	40	46	52	58	64	0
**peptide 30/ 0.5+**		0	0	0	0	0	0	0	0	0	0	0	0	0		
**peptide 30/ 1+**		400	400	400	400	400	400	400	400	400	400	400	400	400		
**peptide 30/ 2+**	1000.511	2360	10685	15722	16241	12287	11580	9716	9838	8654	10146	5780	2419	2038	**n.d.**	**n.d.**
**peptide 30/ 3+**	667.34	400	400	400	579	744	966	1637	1989	2169	3379	3264	2080	2022	**n.d.**	**n.d.**
**peptide 30/ 4+**	500.76	0	0	0	0	0	0	0	0	0	866	292	0	0	**n.d.**	**n.d.**
**antibody+0 pep/ 28+**		0	0	0	0	0	0	0	0	0	0	0	0	0		0
**antibody+0 pep/ 27+**		80	80	80	80	80	80	80	80	80	80	80	80	80		80
**antibody+0 pep/ 26+**	5696.092	2407	2582	2312	1989	2042	1429	1691	1287	1750	1976	2631	2255	2473	**n.d.**	115
**antibody+0 pep/ 25+**	5924.557	14721	17440	15488	14473	12994	10392	11990	10005	11981	15215	17396	14151	16086	**n.d.**	917
**antibody+0 pep/ 24+**	6171.409	36744	46005	36437	37667	33121	28560	30558	28019	30562	41545	44359	35411	40656	**n.d.**	2723
**antibody+0 pep/ 23+**	6440.084	34868	44586	34453	36584	30288	28954	28046	27992	28082	40047	42311	33062	38140	**n.d.**	2975
**antibody+0 pep/ 22+**	7240.001	13202	16921	12339	13826	11383	11355	10445	11141	10282	15490	15650	12189	14809	**n.d.**	1315
**antibody+0 pep/ 21+**	7055.835	2443	2984	2922	2465	1987	2159	2002	2016	1809	2799	2829	2242	2838	**n.d.**	288
**antibody+0 pep/ 20+**	7408.629	225	283	261	255	224	249	204	231	260	348	347	237	331	**n.d.**	265
**antibody+1 pep/ 28+**		0	0	0	0	0	0	0	0	0	0	0	0	0		0
**antibody+1 pep/ 27+**		80	80	80	80	80	80	80	80	80	80	80	80	80		80
**antibody+1 pep/ 26+**	5696.092	2407	2582	2312	1989	2042	1429	1691	1287	1750	1976	2631	2255	2473	**n.d.**	115
**antibody+1 pep/ 25+**	5924.557	14721	17440	15488	14473	12994	10392	11990	10005	11981	15215	17396	14151	16086	**n.d.**	917
**antibody+1 pep/ 24+**	6171.409	36744	46005	36437	37667	33121	28560	30558	28019	30562	41545	44359	35411	40656	**n.d.**	2723
**antibody+1 pep/ 23+**	6440.084	34868	44586	34453	36584	30288	28954	28046	27992	28082	40047	42311	33062	38140	**n.d.**	2975
**antibody+1 pep/ 22+**	6733.612	13202	16921	12339	13826	11383	11355	10445	11141	10282	15490	15650	12189	14809	**n.d.**	1315
**antibody+1 pep/ 21+**	7055.835	2443	2984	2922	2465	1987	2159	2002	2016	1809	2799	2829	2242	2838	**n.d.**	288
**antibody+1 pep/ 20+**	7408.628	225	283	261	255	224	249	204	231	260	348	347	237	331	**n.d.**	265

Table S2: continued

M11+P30, measurement 2

ion / charge state	m/z	ΔCV 2	6	10	14	18	22	26	30	34	40	46	52	58	64	0
**peptide 30/ 0.5+**		0	0	0	0	0	0	0	0	0	0	0	0	0		
**peptide 30/ 1+**		400	400	400	400	400	400	400	400	400	400	400	400	400		
**peptide 30/ 2+**	1000.51	5367	31470	43273	33776	17640	8884	10271	11263	13124	6487	3771	2559	1188	**n.d.**	**n.d.**
**peptide 30/ 3+**	667.34	774	2132	2147	803	689	1147	1133	2887	1963	1331	2188	601	774	**n.d.**	**n.d.**
**peptide 30/ 4+**	500.76	0	0	0	0	0	0	0	0	587	385	291	488	188	**n.d.**	**n.d.**
**antibody+0 pep/ 28+**		0	0	0	0	0	0	0	0	0	0	0	0	0		0
**antibody+0 pep/ 27+**		80	80	80	80	80	80	80	80	80	80	80	80	80		80
**antibody+0 pep/ 26+**	5696.29	6878	7480	4222	1485	549	657	669	936	738	793	1313	915	261	**n.d.**	2519
**antibody+0 pep/ 25+**	5924.787	41178	42776	24196	8063	3434	4251	4538	6402	4494	4478	7496	5027	1955	**n.d.**	22819
**antibody+0 pep/ 24+**	6171.319	99202	99646	56647	17107	8584	11251	11817	17022	11613	11234	17967	11034	5578	**n.d.**	56226
**antibody+0 pep/ 23+**	6439.772	96186	94856	53107	14661	8013	10780	11303	17064	11399	10674	17687	10223	5529	**n.d.**	55565
**antibody+0 pep/ 22+**	6733.017	36619	36218	20220	5341	3058	4259	4414	6762	4434	3999	6912	3748	2232	**n.d.**	21562
**antibody+0 pep/ 21+**	7055.07	6800	6968	3906	968	580	896	886	1440	902	842	1420	750	440	**n.d.**	4097
**antibody+0 pep/ 20+**	7411.223	822	831	491	116	65	116	114	110	132	124	111	109	55	**n.d.**	488
**antibody+1 pep/ 28+**		0	0	0	0	0	0	0	0	0	0	0	0	0		0
**antibody+1 pep/ 27+**		80	80	80	80	80	80	80	80	80	80	80	80	80		80
**antibody+1 pep/ 26+**	5777.298	1494	1659	809	266	80	80	80	80	80	80	80	80	80	**n.d.**	739
**antibody+1 pep/ 25+**	6004.675	6628	7240	3476	1026	469	593	668	1006	660	619	938	691	335	**n.d.**	3358
**antibody+1 pep/ 24+**	6256.27	13012	13906	6618	1752	973	1256	1299	2074	1370	1295	1974	1264	719	**n.d.**	6657
**antibody+1 pep/ 23+**	6526.794	10634	11054	5280	1279	753	993	1095	1807	1101	1049	1701	984	646	**n.d.**	5529
**antibody+1 pep/ 22+**	6828.359	3739	3972	1863	469	251	418	388	678	418	371	661	386	231	**n.d.**	1953
**antibody+1 pep/ 21+**	7164.265	746	80	80	80	80	80	80	80	80	80	80	80	80	**n.d.**	433
**antibody+1 pep/ 20+**		0	0	0	0	0	0	0	0	0	0	0	0	0		0

Table S2: continued

M11+P31, measurement 1

ion / charge state	m/z	ΔCV 2	6	10	14	18	22	26	30	34	40	46	52	58	64	0
**peptide 31/ 0.5+**		0	0	0	0	0	0	0	0	0	0	0	0	0		
**peptide 31/ 1+**		400	400	400	400	400	400	400	400	400	400	400	400	400		
**peptide 31/ 2+**	971.467	3242	26192	21330	17406	13742	13435	13216	11405	11105	9635	5006	2465	1422	**n.d.**	**n.d.**
**peptide 31/ 3+**	648	400	400	400	682	988	1547	1531	2724	3352	3532	3649	3833	2442	**n.d.**	**n.d.**
**peptide 31/ 4+**	486.253	0	0	0	0	0	0	0	0	0	0	940	759	575	**n.d.**	**n.d.**
**antibody+0 pep/ 27+**		0	0	0	0	0	0	0	0	0	0	0	0	0		0
**antibody+0 pep/ 26+**	5694.664	1425	3231	1932	2050	1606	1403	1721	1255	1579	1487	1573	1801	1594	**n.d.**	174
**antibody+0 pep/ 25+**	5923.351	10385	22013	13872	13739	10984	10294	11213	9591	11338	10522	10784	12222	10935	**n.d.**	1744
**antibody+0 pep/ 24+**	6169.686	28439	57125	37761	34289	28564	27891	28870	27308	29967	29306	31407	32682	30833	**n.d.**	6359
**antibody+0 pep/ 23+**	6437.047	29734	59695	39733	34582	29752	29696	29919	30171	31554	31551	35794	35790	34440	**n.d.**	8567
**antibody+0 pep/ 22+**	6729.882	12655	24944	17420	14249	12821	12905	12817	13668	13246	13819	16142	15878	15868	**n.d.**	4567
**antibody+0 pep/ 21+**	7052.197	2476	4940	3583	3905	2551	2660	2748	2939	2739	2879	3515	3325	3467	**n.d.**	1149
**antibody+0 pep/ 20+**	7406.829	213	311	452	365	331	380	388	407	358	402	528	488	528	**n.d.**	195
**antibody+0 pep/ 19+**		0	0	0	0	0	0	0	0	0	0	0	0	0		0
**antibody+1 pep/ 28+**		0	0	0	0	0	0	0	0	0	0	0	0	0		0
**antibody+1 pep/ 27+**		80	80	80	80	80	80	80	80	80	80	80	80	80		80
**antibody+1 pep/ 26+**		80	80	80	80	80	80	80	80	80	80	80	80	80		80
**antibody+1 pep/ 25+**	6000.162	1053	2208	1355	1240	1012	917	987	863	951	938	936	1021	981	**n.d.**	240
**antibody+1 pep/ 24+**	6251.463	2292	4473	2900	2569	2122	2047	2106	1921	2191	2142	2278	2281	2033	**n.d.**	544
**antibody+1 pep/ 23+**	6484.098	2069	3986	2672	2225	1897	1889	1898	1845	1983	1970	2133	2113	2067	**n.d.**	601
**antibody+1 pep/ 22+**	6820.1	564	1620	1090	891	784	760	747	794	770	803	905	929	879	**n.d.**	303
**antibody+1 pep/ 21+**	7157.655	100	182	552	80	80	80	80	80	80	80	80	80	80	**n.d.**	80
**antibody+1 pep/ 20+**		0	0	0	0	0	0	0	0	0	0	0	0	0		0

Table S2: continued

M11+P31, measurement 2

ion / charge state	m/z	ΔCV 2	6	10	14	18	22	26	30	34	40	46	52	58	64	0
**peptide 31/ 0.5+**		0	0	0	0	0	0	0	0	0	0	0				
**peptide 31/ 1+**		400	400	400	400	400	400	400	400	400	400	400	0	0		
**peptide 31/ 2+**	971.507	9543	25166	41711	49357	40672	34406	24674	26430	21439	21859	10146	3715	1635	**n.d.**	**n.d.**
**peptide 31/ 3+**	648.01	400	400	400	1258	2358	3197	2841	4100	4570	6981	7507	5326	3104	**n.d.**	**n.d.**
**peptide 31/ 4+**	486.246	0	0	0	0	0	0	0	0	667	713	945	585	981	**n.d.**	**n.d.**
**peptide 31/ 5+**													0	0		
**antibody+0 pep / 27+**		0	0	0	0	0	0	0	0	0	0	0	0	0		0
**antibody+0 pep / 26+**	5693.995	1522	1657	1485	1927	1638	1626	1102	1403	1079	1547	1646	1532	1257	**n.d.**	91
**antibody+0 pep / 25+**	5922.651	10419	11358	10987	14224	12805	12004	8603	5921	8881	11784	11729	10486	8695	**n.d.**	924
**antibody+0 pep / 24+**	6169.686	35847	30476	31290	39856	36569	32937	25240	30517	25271	34253	32885	27889	23374	**n.d.**	3097
**antibody+0 pep / 23+**	6438.219	44968	33251	37451	44414	41915	36473	29005	34196	27631	38727	37806	31881	24974	**n.d.**	4126
**antibody+0 pep / 22+**	6731.45	23077	14376	17750	20205	19604	16495	13895	15603	12626	17565	17199	14447	11562	**n.d.**	2081
**antibody+0 pep / 21+**	7053.096	5370	2898	3739	4469	4249	3483	2973	3378	2738	2846	3858	3158	2613	**n.d.**	1254
**antibody+0 pep / 20+**	7405.672	235	327	390	580	598	452	387	434	397	534	543	458	352	**n.d.**	76
**antibody+0 pep / 19+**		0	0	0	0	0	0	0	0	0	0	0	0	0		0
**antibody+1 pep/ 28+**		0	0	0	0	0	0	0	0	0	0	0	0	0		0
**antibody+1 pep/ 27+**		80	80	80	80	80	80	80	80	80	80	80	80	80		80
**antibody+1 pep/ 26+**	5770.53	493	373	320	458	371	371	210	282	310	349	357	276	228	**n.d.**	80
**antibody+1 pep/ 25+**	6000.63	2983	1910	1854	2364	2033	1744	1260	1532	1256	1916	1910	1400	1132	**n.d.**	177
**antibody+1 pep/ 24+**	6251.626	7178	4154	4347	5226	4685	3956	2834	3412	2799	4485	4342	3276	2573	**n.d.**	463
**antibody+1 pep/ 23+**	6523.802	7637	3719	4344	5046	4621	3711	2792	3357	2604	4371	4227	3212	2386	**n.d.**	450
**antibody+1 pep/ 22+**	6822.059	3486	1527	1918	2122	1948	1586	1245	1383	1125	1812	1919	1318	1077	**n.d.**	226
**antibody+1 pep/ 21+**	7145.191	80	80	425	478	479	365	305	325	254	446	489	351	267	**n.d.**	65
**antibody+1 pep/ 20+**		0	0	0	0	0	0	0	0	0	0	0	0	0		0

Table S2: continued

M11+P32, measurement 1

ion / charge state	m/z	ΔCV 2	6	10	14	18	22	26	30	34	40	46	52	58	64	0
**peptide 32/ 0.5+**		0	0	0	0											
**peptide 32/ 1+**		400	400	400	400	0	0	0	0	0	0	0	0	0		0
**peptide 32/ 2+**	985.042	4782	14043	12236	19734	19590	18358	11406	10674	9792	9066	7724	6544	7819	**n.d.**	100
**peptide 32/ 3+**	657.042	1479	3380	4398	17407	28903	40951	38376	51505	64106	86312	91882	64556	82539	**n.d.**	162
**peptide 32/ 4+**	493.023	1080	1894	3501	12534	19218	25342	24486	30553	45273	51843	65232	62813	71977	**n.d.**	100
**peptide 32/ 5+**						0	0	0	0	0	0	0	0	0		0
**antibody+0 pep/ 27+**		0	0	0	0	0	0	0	0	0	0	0	0	0		0
**antibody+0 pep/ 26+**	5696.27	830	1035	858	1079	1105	822	691	544	629	716	686	582	656	**n.d.**	112
**antibody+0 pep/ 25+**	5923.234	7786	7749	7280	8561	8170	6846	5078	4569	4868	4977	4939	4161	4594	**n.d.**	1172
**antibody+0 pep/ 24+**	6169.934	24748	23009	21596	25096	23952	20836	14478	13881	14879	14933	14963	12591	14757	**n.d.**	4479
**antibody+0 pep/ 23+**	6438.548	32352	26739	25942	29666	27625	24331	16513	16658	16662	17877	17956	15158	17535	**n.d.**	6164
**antibody+0 pep/ 22+**	6731.879	16857	13127	12860	14132	13194	12004	8108	8315	8237	8925	8911	7937	9464	**n.d.**	3382
**antibody+0 pep/ 21+**	7053.062	4120	2962	2917	3283	3198	2979	2057	2287	2414	2896	2907	2576	3134	**n.d.**	870
**antibody+0 pep/ 20+**	7408.251	536	410	397	430	434	445	354	464	566	746	817	786	987	**n.d.**	124
**antibody+0 pep/ 19+**		0	0	0	0	0	0	0	0	0	0	0	0	0		0
**antibody+1 pep/ 27+**		0	0	0	0	0	0	0	0	0	0	0	0	0		0
**antibody+1 pep/ 26+**	5773.124	1616	1690	1388	1617	1452	1122	821	672	656	580	589	466	527	**n.d.**	193
**antibody+1 pep/ 25+**	6003.052	7714	7295	6674	7315	6666	5308	3599	3151	3316	2788	2762	2174	2555	**n.d.**	1132
**antibody+1 pep/ 24+**	6253.61	14260	12046	11264	12623	11459	9579	6232	5824	5713	5279	5267	4313	4879	**n.d.**	2333
**antibody+1 pep/ 23+**	6526.137	11444	9074	8476	9513	8554	7524	4781	4770	4531	4562	4564	3808	4391	**n.d.**	1912
**antibody+1 pep/ 22+**	6824.153	4719	3389	3192	3591	3201	2825	1930	1943	1946	1988	1973	1757	2035	**n.d.**	866
**antibody+1 pep/ 21+**	7147.642	1072	723	726	795	727	673	458	501	521	583	628	578	697	**n.d.**	225
**antibody+1 pep/ 20+**	7521.15	1616	1690	1388	1617	1452	1122	821	672	656	580	589	466	527	**n.d.**	193
**antibody+1 pep/ 19+**		0	0	0	0	0	0	0	0	0	0	0	0	0		0
**antibody+2 peps/ 28+**		0	0	0	0	0	0	0	0	0	0	0	0	0		0
**antibody+2 peps/ 27+**		80	80	80	80	80	80	80	80	80	80	80	80	80		80
**antibody+2 peps/ 26+**	5854.352	681	694	597	580	522	411	283	233	235	182	171	80	681	**n.d.**	94
**antibody+2 peps/ 25+**	6083.602	2064	1763	1592	1659	1438	1167	740	800	625	556	501	390	2064	**n.d.**	306
**antibody+2 peps/ 24+**	6338.769	2557	1951	1733	1869	1658	1376	848	800	759	769	664	549	2557	**n.d.**	368
**antibody+2 peps/ 23+**	6614.343	1563	1123	1020	1122	995	816	547	545	548	519	506	411	1563	**n.d.**	248
**antibody+2 peps/ 22+**	6917.188	628	416	360	373	357	302	216	225	233	252	237	80	628	**n.d.**	105
**antibody+2 peps/ 21+**		80	80	80	80	80	80	80	80	80	80	80	80	80		80
**antibody+2 peps/ 20+**		0	0	0	0	0	0	0	0	0	0	0	0	0		0

Table S2: continued

M11+P32, measurement 2

ion / charge state	m/z	ΔCV 2	6	10	14	18	22	26	30	34	40	46	52	58	64	0
**peptide 32/ 0.5**		0	0	0	0											
**peptide 32/ 1+**		400	400	400	400	0	0	0	0	0	0	0	0	0		
**peptide 32/ 2+**	985.052	2818	9695	11968	12238	6609	8614	6663	7986	9000	4693	4249	5405	5152	**n.d.**	**n.d.**
**peptide 32/ 3+**	657.041	821	1695	3515	6075	7103	16030	21145	35495	48041	41708	38990	37138	34502	**n.d.**	**n.d.**
**peptide 32/ 4+**	439.029	629	1403	1945	4346	5554	10811	14855	27540	40080	37205	32816	43678	44205	**n.d.**	**n.d.**
**peptide 32/ 5+**						0	0	0	0	0	0	0	0	0		
**antibody+0 pep/ 27+**		0	0	0	0	0	0	0	0	0	0	0	0	0		0
**antibody+0 pep/ 26+**	5693.712	565	520	463	449	305	374	311	404	404	296	269	360	380	**n.d.**	94
**antibody+0 pep/ 25+**	5922.591	4741	4042	4426	4092	2500	3325	2723	3791	4063	2751	2524	2847	2982	**n.d.**	803
**antibody+0 pep/ 24+**	6169.063	14302	12053	12649	13198	7963	10513	9035	12054	13649	9285	8258	9266	9530	**n.d.**	3673
**antibody+0 pep/ 23+**	6437.764	17339	13725	14672	15406	9310	12427	11226	14347	16785	11451	10388	11794	12040	**n.d.**	5513
**antibody+0 pep/ 22+**	6731.044	8575	6294	6725	7250	4495	5976	5469	7130	8487	6026	5379	6436	6530	**n.d.**	3163
**antibody+0 pep/ 21+**	7051.524	1978	1350	1485	1650	1000	1503	1486	2019	2472	1753	1708	2147	2204	**n.d.**	869
**antibody+0 pep/ 20+**	7405.945	274	172	176	194	141	224	260	387	569	504	501	646	693	**n.d.**	142
**antibody+0 pep/ 19+**		0	0	0	0	0	0	0	0	0	0	0	0	0		0
**antibody+1 pep/ 28+**		0	0	0	0	0	0	0	0	0	0	0	0	0		0
**antibody+1 pep/ 27+**		80	80	80	80	80	80	80	80	80	80	80	80	80		80
**antibody+1 pep/ 26+**	5772.889	1616	1690	1388	1617	1452	1122	821	672	656	580	589	466	527	**n.d.**	193
**antibody+1 pep/ 25+**	6001.806	7714	7295	6674	7315	6666	5308	3599	3151	3316	2788	2762	2174	2555	**n.d.**	1132
**antibody+1 pep/ 24+**	6252.667	14260	12046	11264	12623	11459	9579	6232	5824	5713	5279	5267	4313	4879	**n.d.**	2333
**antibody+1 pep/ 23+**	6524.688	11444	9074	8476	9513	8554	7524	4781	4770	4531	4562	4564	3808	4391	**n.d.**	1912
**antibody+1 pep/ 22+**	6821.477	4719	3389	3192	3591	3201	2825	1930	1943	1946	1988	1973	1757	2035	**n.d.**	866
**antibody+1 pep/ 21+**	7148.078	1072	723	726	795	727	673	458	501	521	583	628	578	697	**n.d.**	225
**antibody+1 pep/ 20+**		0	0	0	0	0	0	0	0	0	0	0	0	0		0
**antibody+2 peps/ 28+**		0	0	0	0	0	0	0	0	0	0	0	0	0		0
**antibody+2 peps/ 27+**		80	80	80	80	80	80	80	80	80	80	80	80	80		80
**antibody+2 peps/ 26+**	5851.67	395	345	331	264	165	207	178	211	185	115	266	266	266	**n.d.**	100
**antibody+2 peps/ 25+**	6083.315	1102	888	873	836	495	567	478	555	612	367	267	284	268	**n.d.**	251
**antibody+2 peps/ 24+**	6336.181	1170	923	930	945	558	705	592	707	768	516	430	417	402	**n.d.**	295
**antibody+2 peps/ 23+**	6610.65	714	531	522	538	312	415	369	472	527	343	270	307	321	**n.d.**	201
**antibody+2 peps/ 22+**	6915.062	271	171	185	206	124	179	143	197	221	167	217	160	183	**n.d.**	82
**antibody+2 peps/ 21+**		80	80	80	80	80	80	80	80	80	80	80	80	80		80
**antibody+2 peps/ 20+**		0	0	0	0	0	0	0	0	0	0	0	0	0		0

Footnote: Imputed values are necessary for optimized Gauss-fit (red) and are equal to the background intensity at the given m/z and/or 0. n.d.: value not determined/ion signal not present

Table S3: Apex heights (h) and mean charge ($\bar{z}$) states of precursors and product ion signals upon gas phase dissociation of M7 antibody immune complex and assigned peptide.

M7+P11 Experiment 1

ΔCV	Epitope peptide		Antibody		Antibody complex+1 peptide		Antibody complex+2 peptides	
[V]	$\bar{z}_{(1)}$+	$h_{(1)}$	$\bar{z}_{(2)}$+	$h_{(2)}$	$\bar{z}_{(3)}$+	$h_{(3)}$	$\bar{z}_{(4)}$+	$h_{(4)}$
0	3,117	63,156	24,208	21274,594	23,845	13941,291	25,244	2552,648
2	3,135	2208,286	24,245	22304,413	24,773	14058,537	25,283	2662,240
6	3,052	80,009	24,134	18723,202	24,660	11828,604	25,267	2261,886
10	3,245	926,982	24,221	14770,314	24,766	9052,543	25,291	1707,306
14	3,264	2121,499	24,188	13539,099	24,724	8434,187	25,234	1565,358
18	3,238	3950,073	24,173	13076,594	24,685	8034,494	25,232	1501,207
22	3,235	3964,325	24,172	13186,761	24,684	8092,332	25,225	1517,951
26	3,241	7482,016	24,216	13686,419	24,720	8110,103	25,214	1502,316
30	3,227	10958,558	24,199	13328,112	24,709	7821,897	25,230	1374,349
34	3,194	16690,708	24,134	13067,856	24,659	7158,609	25,177	1235,543
40	3,174	23406,670	24,144	14110,451	24,571	6891,400	25,168	1188,130
46	3,150	12780,796	24,033	6531,591	24,528	3259,595	25,062	479,893
52	3,145	14318,299	24,112	7198,900	24,580	3427,132	25,388	504,905
58	3,120	13753,985	24,075	7145,230	24,537	3343,004	24,990	334,784

Missing data at predefined collision voltage intervals were supplemented by interpolating from corresponding data points within the same experimental dataset and are indicated in red. n.a.: data was not produced for both replicates.

M7+P11 Experiment 2

ΔCV	Epitope peptide		Antibody		Antibody complex+1 peptide		Antibody complex+2 peptides	
[V]	$\bar{z}_{(1)}$	$h_{(1)}$	$\bar{z}_{(2)}$	$h_{(2)}$	$\bar{z}_{(3)}$	$h_{(3)}$	$\bar{z}_{(4)}$	$h_{(4)}$
0	3,117	63,156	24,030	3975,562	24,578	2481,262	25,132	486,562
2	3,135	2208,286	24,108	3218,688	24,626	1846,937	25,440	319,881
6	3,052	80,009	24,061	7633,362	24,458	5104,242	25,096	981,615
10	3,222	545,009	24,137	8865,039	24,684	5736,488	25,218	1075,292
14	3,218	612,657	24,169	9006,602	24,690	5720,094	25,235	1089,508
18	3,255	3216,812	24,211	9813,388	24,734	5972,086	25,260	1134,865
22	3,234	5653,234	24,243	9771,690	24,763	5887,366	25,274	1070,565
26	3,228	8606,511	24,264	9982,139	23,819	4217,545	25,257	1037,332
30	3,213	10085,402	24,151	9356,011	24,679	5293,890	25,191	949,518
34	3,195	13526,623	24,170	10205,560	24,651	5609,546	25,200	965,149
40	3,191	10268,569	23,954	7613,258	24,473	4190,304	24,990	684,808
46	3,147	6796,895	23,904	3920,958	26,628	22303,440	24,879	295,395
52	3,138	6678,911	23,769	3801,031	24,242	1799,963	24,778	243,027
58	3,126	9444,868	24,054	5463,658	24,519	2500,501	24,990	334,784

Missing data at predefined collision voltage intervals were supplemented by interpolating from corresponding data points within the same experimental dataset and are indicated in red. n.a.: data was not produced for both replicates.

M7+P12 Experiment 1

ΔCV	Epitope peptide		Antibody		Antibody complex+1 peptide		Antibody complex+2 peptides	
[V]	$\bar{z}_{(1)}$	$h_{(1)}$	$\bar{z}_{(2)}$	$h_{(2)}$	$\bar{z}_{(3)}$	$h_{(3)}$	$\bar{z}_{(4)}$	$h_{(4)}$
0	n.d.	n.d.	23,994	2242,135	24,228	243,984	n.d.	n.d.
2	n.d.	n.d.	24,023	4515,913	24,920	452,228	n.d.	n.d.
6	n.d.	n.d.	23,965	5586,423	24,354	654,947	n.d.	n.d.
10	3,000	22,998	24,004	8153,534	24,419	949,831	n.d.	n.d.
14	3,000	227,660	23,973	16035,743	24,404	1862,600	n.d.	n.d.
18	3,250	354,168	23,992	8437,850	24,399	959,846	n.d.	n.d.
22	3,303	396,380	24,014	8370,098	24,415	948,986	n.d.	n.d.
26	3,226	595,706	24,024	8129,492	24,396	902,849	n.d.	n.d.
30	3,251	313,311	24,024	3930,332	24,342	408,406	n.d.	n.d.
34	3,228	358,786	24,003	3967,310	24,319	420,133	n.d.	n.d.
40	3,159	451,826	23,995	4396,379	24,368	453,215	n.d.	n.d.
46	3,181	436,612	23,960	2983,568	24,304	326,675	n.d.	n.d.
52	3,219	536,314	23,974	4093,532	24,330	387,331	n.d.	n.d.
58	3,202	347,188	23,963	3647,980	24,278	332,231	n.d.	n.d.

Missing data at predefined collision voltage intervals were supplemented by interpolating from corresponding data points within the same experimental dataset and are indicated in red. n.a.: data was not produced for both replicates.

Table S3: continued

M7+P12 Experiment 2

ΔCV	Epitope peptide		Antibody		Antibody complex+1 peptide		Antibody complex+2 peptides	
[V]	$\bar{z}_{(1)}$	$h_{(1)}$	$\bar{z}_{(2)}$	$h_{(2)}$	$\bar{z}_{(3)}$	$h_{(3)}$	$\bar{z}_{(4)}$	$h_{(4)}$
0	n.d.	n.d.	23,994	2242,135	24,228	243,984	n.d.	n.d.
2	n.d.	n.d.	23,951	4260,893	24,354	654,947	n.d.	n.d.
6	n.d.	n.d.	23,948	5759,576	24,390	689,745	n.d.	n.d.
10	2,850	21,886	23,965	6629,365	24,372	780,768	n.d.	n.d.
14	2,605	37,135	23,947	4179,515	24,344	463,070	n.d.	n.d.
18	3,255	139,304	23,978	5112,068	24,446	551,116	n.d.	n.d.
22	3,301	302,297	24,036	6685,816	24,401	742,667	n.d.	n.d.
26	3,265	412,106	24,061	6033,589	24,392	646,732	n.d.	n.d.
30	3,227	385,333	24,004	5038,549	24,381	508,567	n.d.	n.d.
34	3,259	314,562	23,975	3017,716	24,331	272,229	n.d.	n.d.
40	3,181	260,867	23,974	2303,662	24,231	207,494	n.d.	n.d.
46	3,072	191,538	23,915	2271,286	24,336	161,520	n.d.	n.d.
52	3,222	315,204	23,925	3899,187	24,255	352,170	n.d.	n.d.
58	3,194	253,797	23,924	3756,426	24,339	339,989	n.d.	n.d.

Missing data at predefined collision voltage intervals were supplemented by interpolating from corresponding data points within the same experimental dataset and are indicated in red. n.a.: data was not produced for both replicates.

M7+P13 Experiment 1

ΔCV	Epitope peptide		Antibody		Antibody complex+1 peptide		Antibody complex+2 peptides	
[V]	$\bar{z}_{(1)}$	$h_{(1)}$	$\bar{z}_{(2)}$	$h_{(2)}$	$\bar{z}_{(3)}$	$h_{(3)}$	$\bar{z}_{(4)}$	$h_{(4)}$
0	n.d.	n.d.	n.d.	n.d.	n.d.	n.d.	n.d.	n.d.
2	3,000	101,290	24,273	4656,158	24,679	5091,966	25,089	1589,296
6	2,936	190,043	24,301	5222,444	24,730	5589,610	25,119	1666,166
10	3,024	685,972	24,315	5732,554	24,707	6142,708	25,111	1826,614
14	3,073	1862,387	24,329	6202,357	24,734	6600,044	25,107	1972,419
18	3,041	12712,326	24,365	21474,945	24,758	22678,075	25,145	6447,097
22	3,029	4856,035	24,302	5078,937	24,677	5300,516	25,073	1491,475
26	3,008	17850,324	24,342	12300,754	24,739	12544,188	25,133	3571,151
30	2,992	23448,388	24,341	11460,420	24,765	11381,218	25,152	3149,574
34	2,973	29758,871	24,336	11586,104	24,748	11222,152	25,116	3022,434
40	2,947	34679,797	24,233	9723,252	24,678	8591,384	25,117	2181,367
46	2,921	41840,942	24,216	9686,374	24,648	8052,631	25,050	1850,453
52	2,899	25712,135	24,110	5868,735	24,525	4922,148	24,958	1130,173
58	2,864	35787,397	24,081	8069,053	24,506	6285,146	24,942	1361,941

Missing data at predefined collision voltage intervals were supplemented by interpolating from corresponding data points within the same experimental dataset and are indicated in red. n.a.: data was not produced for both replicates.

M7+P13 Experiment 2

ΔCV	Epitope peptide		Antibody		Antibody complex+1 peptide		Antibody complex+2 peptides	
[V]	$\bar{z}_{(1)}$	$h_{(1)}$	$\bar{z}_{(2)}$	$h_{(2)}$	$\bar{z}_{(3)}$	$h_{(3)}$	$\bar{z}_{(4)}$	$h_{(4)}$
0	n.d.	n.d.	n.d.	n.d.	n.d.	n.d.	n.d.	n.d.
2	3,000	218,978	24,110	5962,226	24,530	6765,513	24,932	2227,398
6	3,058	693,596	24,167	9264,753	24,594	10719,691	24,995	3406,981
10	3,035	1110,813	24,144	5671,652	24,534	6342,471	24,956	1993,521
14	3,044	2058,462	24,084	5333,582	24,492	5953,605	24,899	1877,194
18	3,038	2723,593	24,078	3586,823	24,467	3910,543	24,782	1157,931
22	3,022	6110,790	24,022	4733,022	24,446	5210,855	24,827	1590,885
26	3,007	5610,729	24,052	2998,031	24,918	33136,068	24,855	918,693
30	2,987	5702,584	24,005	2296,211	24,455	2328,647	24,899	672,573
34	2,977	7031,113	24,035	2381,039	24,472	2316,309	24,906	615,422
40	2,927	6068,690	23,987	1663,792	24,443	1525,354	24,802	366,726
46	2,909	6234,969	24,740	14890,429	24,363	1390,291	24,809	334,578
52	2,880	6438,338	23,948	1682,129	24,374	1403,364	24,758	290,963
58	2,851	5981,536	23,960	1624,863	24,375	1395,230	24,810	321,900

Missing data at predefined collision voltage intervals were supplemented by interpolating from corresponding data points within the same experimental dataset and are indicated in red. n.a.: data was not produced for both replicates.

Table S3: continued

M7+P14 Experiment 1

ΔCV [V]	Epitope peptide $\bar{z}_{(1)}$	Epitope peptide $h_{(1)}$	Antibody $\bar{z}_{(2)}$	Antibody $h_{(2)}$	Antibody complex+1 peptide $\bar{z}_{(3)}$	Antibody complex+1 peptide $h_{(3)}$	Antibody complex+2 peptides $\bar{z}_{(4)}$	Antibody complex+2 peptides $h_{(4)}$
0	2,966	184,302	23,986	1062,585	24,487	760,059	24,918	161,379
2	2,944	595,778	24,107	6510,695	24,575	4887,279	25,038	1003,272
6	2,950	4384,702	24,229	18214,098	24,715	14526,844	25,179	3340,791
10	2,908	6957,462	24,277	20456,327	24,772	16990,195	25,224	3933,693
14	2,934	28275,809	24,267	19622,420	24,745	16295,223	25,191	3751,415
18	2,929	21384,759	24,255	13984,310	24,741	11382,232	25,213	2715,372
22	2,837	51192,690	24,263	19408,921	24,733	15318,381	25,183	3404,968
26	2,946	120243,589	24,317	19804,579	24,778	14853,700	25,269	3279,730
30	2,945	106753,902	24,308	13831,585	24,802	9898,825	25,285	2102,672
34	2,877	118435,093	24,314	12404,919	24,813	8584,443	25,277	1725,371
40	2,702	201232,167	24,324	25922,233	24,796	16691,623	25,263	3171,243
46	2,668	109215,074	24,311	12924,588	24,799	7847,622	25,223	1343,269
52	2,598	93299,940	24,321	11478,330	24,772	6686,176	25,262	1103,768
58	2,668	61373,829	24,314	5520,242	24,779	3115,227	25,191	480,948

Missing data at predefined collision voltage intervals were supplemented by interpolating from corresponding data points within the same experimental dataset and are indicated in red. n.a.: data was not produced for both replicates.

M7+P14 Experiment 2

ΔCV [V]	Epitope peptide $\bar{z}_{(1)}$	Epitope peptide $h_{(1)}$	Antibody $\bar{z}_{(2)}$	Antibody $h_{(2)}$	Antibody complex+1 peptide $\bar{z}_{(3)}$	Antibody complex+1 peptide $h_{(3)}$	Antibody complex+2 peptides $\bar{z}_{(4)}$	Antibody complex+2 peptides $h_{(4)}$
0	2,921	1320,120	24,287	985,609	24,726	713,320	24,873	175,743
2	2,894	2492,792	24,362	11208,649	24,868	9179,784	25,368	2131,742
6	2,970	3468,004	24,400	9356,647	24,882	7646,083	25,354	1791,497
10	2,896	5600,711	24,383	14034,691	24,880	11227,631	25,319	2515,654
14	2,954	9167,595	24,360	11012,834	24,833	8694,376	25,291	1902,723
18	2,857	17917,939	24,313	10535,411	24,782	8184,472	25,247	1770,123
22	2,919	57443,842	24,348	11907,754	24,806	8952,690	25,255	1914,755
26	2,943	45376,526	24,338	7167,545	24,786	5181,427	25,259	1036,499
30	2,897	69804,203	24,372	8803,529	24,850	6175,035	25,279	1220,039
34	2,866	65169,850	24,386	7347,420	24,850	4965,754	25,403	1002,450
40	2,721	33828,019	24,345	4556,600	24,833	2850,916	25,239	512,671
46	2,787	66687,495	24,325	5552,721	24,787	3242,025	25,226	538,888
52	2,598	93299,940	24,321	11478,330	24,772	6686,176	25,262	1103,768
58	2,668	61373,829	24,314	5520,242	24,779	3115,227	25,191	480,948

Missing data at predefined collision voltage intervals were supplemented by interpolating from corresponding data points within the same experimental dataset and are indicated in red. n.a.: data was not produced for both replicates.

M7+P15 Experiment 1

ΔCV [V]	Epitope peptide $\bar{z}_{(1)}$	Epitope peptide $h_{(1)}$	Antibody $\bar{z}_{(2)}$	Antibody $h_{(2)}$	Antibody complex+1 peptide $\bar{z}_{(3)}$	Antibody complex+1 peptide $h_{(3)}$	Antibody complex+2 peptides $\bar{z}_{(4)}$	Antibody complex+2 peptides $h_{(4)}$
0	n.d.	n.d.	n.d.	n.d.	n.d.	n.d.	n.d.	n.d.
2	3,000	13,505	24,130	2913,020	24,675	1164,095	n.d.	n.d.
6	3,139	49,307	24,134	3221,928	24,676	1373,447	n.d.	n.d.
10	3,025	157,158	24,155	3991,920	24,633	1578,656	n.d.	n.d.
14	3,050	73,359	24,205	932,922	24,759	372,823	n.d.	n.d.
18	3,037	467,859	23,874	2403,288	24,641	1028,162	n.d.	n.d.
22	3,050	301,019	24,201	1262,199	24,612	452,762	n.d.	n.d.
26	2,985	367,070	24,194	952,088	24,584	363,804	n.d.	n.d.
30	2,994	1160,622	24,213	2206,281	24,653	776,511	n.d.	n.d.
34	2,926	2324,523	24,168	3941,056	24,661	1333,839	n.d.	n.d.
40	2,890	995,156	24,135	1379,743	24,560	444,514	n.d.	n.d.
46	2,846	1169,556	24,092	1591,303	24,601	489,210	n.d.	n.d.
52	2,742	1587,454	24,066	2708,847	24,585	797,269	n.d.	n.d.
58	2,673	745,233	24,109	1659,774	24,546	457,074	n.d.	n.d.

Missing data at predefined collision voltage intervals were supplemented by interpolating from corresponding data points within the same experimental dataset and are indicated in red. n.a.: data was not produced for both replicates.

Table S3: continued

M7+P15 Experiment 2

ΔCV	Epitope peptide		Antibody		Antibody complex+1 peptide		Antibody complex+2 peptides	
[V]	$\bar{z}_{(1)}$	$h_{(1)}$	$\bar{z}_{(2)}$	$h_{(2)}$	$\bar{z}_{(3)}$	$h_{(3)}$	$\bar{z}_{(4)}$	$h_{(4)}$
0	n.d.	n.d.	n.d.	n.d.	n.d.	n.d.	n.d.	n.d.
2	3,000	97,431	24,233	6164,939	24,735	2544,912	n.d.	n.d.
6	3,180	156,796	24,208	7809,744	24,730	3262,328	n.d.	n.d.
10	3,026	280,051	24,168	5602,418	24,662	2313,727	n.d.	n.d.
14	3,066	503,443	24,088	5151,134	24,578	2080,199	n.d.	n.d.
18	3,006	615,663	24,106	4206,591	24,617	1716,264	n.d.	n.d.
22	3,011	994,857	24,119	3779,147	24,643	1476,396	n.d.	n.d.
26	3,006	2056,799	24,165	6384,757	24,604	2410,921	n.d.	n.d.
30	2,959	2433,359	24,202	3928,938	24,636	1679,114	n.d.	n.d.
34	2,937	2080,537	24,172	3386,206	24,654	1167,033	n.d.	n.d.
40	2,891	2777,640	24,174	3780,182	24,593	1171,483	n.d.	n.d.
46	2,842	3075,071	24,153	3783,096	24,628	1199,927	n.d.	n.d.
52	2,754	2389,553	24,096	3596,930	24,527	1093,192	n.d.	n.d.
58	2,694	1902,617	24,086	3497,990	24,523	988,927	n.d.	n.d.

Missing data at predefined collision voltage intervals were supplemented by interpolating from corresponding data points within the same experimental dataset and are indicated in red. n.a.: data was not produced for both replicates.

M7+P16 Experiment 1

ΔCV	Epitope peptide		Antibody		Antibody complex+1 peptide		Antibody complex+2 peptides	
[V]	$\bar{z}_{(1)}$	$h_{(1)}$	$\bar{z}_{(2)}$	$h_{(2)}$	$\bar{z}_{(3)}$	$h_{(3)}$	$\bar{z}_{(4)}$	$h_{(4)}$
0	3,092	1650,399	23,101	1456,813	23,611	1663,662	24,226	522,686
2	2,976	1898,168	23,127	2192,283	23,661	2226,849	24,256	650,164
6	3,026	7333,546	23,344	4636,235	23,928	5437,504	24,531	1817,166
10	3,036	17597,141	23,408	5788,612	23,991	6852,371	24,612	2298,634
14	3,076	29207,059	23,583	9603,957	24,158	11394,006	24,706	3598,968
18	3,043	57565,517	23,670	12486,292	24,224	13854,494	24,797	4396,511
22	3,017	149050,436	23,703	12852,177	24,204	14399,561	24,753	4313,530
26	2,999	389491,845	23,579	13142,214	24,127	13282,245	24,701	3948,269
30	2,973	384037,593	23,382	19292,072	23,922	19361,749	24,514	5557,300
34	2,869	425586,720	23,344	19985,840	23,821	19242,210	24,445	5171,286
40	2,859	426899,300	23,406	14387,904	23,938	12235,946	24,569	3045,644
46	2,832	444147,521	23,364	13868,247	23,941	11494,543	24,544	2710,543
52	2,714	250766,091	23,418	11051,319	23,998	8743,535	24,621	1931,652
58	2,792	266665,471	23,401	8527,026	23,975	6805,529	24,642	1446,313

Missing data at predefined collision voltage intervals were supplemented by interpolating from corresponding data points within the same experimental dataset and are indicated in red. n.a.: data was not produced for both replicates.

M7+P16 Experiment 2

ΔCV	Epitope peptide		Antibody		Antibody complex+1 peptide		Antibody complex+2 peptides	
[V]	$\bar{z}_{(1)}$	$h_{(1)}$	$\bar{z}_{(2)}$	$h_{(2)}$	$\bar{z}_{(3)}$	$h_{(3)}$	$\bar{z}_{(4)}$	$h_{(4)}$
0	2,814	3362,464	23,308	3669,207	23,847	4480,027	24,403	1628,905
2	2,970	2743,469	23,335	3858,898	23,959	4809,739	24,497	1735,304
6	2,831	6676,658	23,438	4687,964	24,019	5636,052	24,659	2015,771
10	2,955	23900,365	23,525	9599,653	24,116	12005,095	24,736	4246,117
14	3,096	30848,374	23,546	12109,199	24,117	15204,581	24,714	5306,299
18	3,058	81505,177	23,461	14850,758	24,027	17682,545	24,629	6156,469
22	3,067	120016,685	23,343	13824,289	23,920	15946,241	24,532	5231,922
26	3,087	166397,285	23,268	12357,967	23,831	13849,556	24,438	4236,819
30	3,082	188674,495	23,171	10418,501	23,739	10882,826	24,420	3190,181
34	3,087	234312,577	23,062	9189,389	23,590	9059,421	24,215	2572,895
40	3,148	223708,095	22,977	6951,667	23,477	6216,118	24,114	1605,977
46	3,295	92238,616	22,855	3201,412	23,366	2638,874	23,982	611,030
52	3,222	123078,177	22,805	3108,639	23,352	2511,785	23,990	572,239
58	3,332	80141,311	22,826	2791,689	23,364	2264,111	24,083	485,978

Missing data at predefined collision voltage intervals were supplemented by interpolating from corresponding data points within the same experimental dataset and are indicated in red. n.a.: data was not produced for both replicates.

Table S3: continued

M7+P17 Experiment 1

ΔCV [V]	Epitope peptide $\bar{z}_{(1)}$	Epitope peptide $h_{(1)}$	Antibody $\bar{z}_{(2)}$	Antibody $h_{(2)}$	Antibody complex+1 peptide $\bar{z}_{(3)}$	Antibody complex+1 peptide $h_{(3)}$	Antibody complex+2 peptides $\bar{z}_{(4)}$	Antibody complex+2 peptides $h_{(4)}$
0	3,110	112,007	23,985	4530,479	24,519	2868,356	25,026	550,415
2	3,000	25,081	24,138	1921,936	24,652	1267,307	25,166	290,153
6	2,987	171,925	24,200	3124,683	24,698	2082,805	25,201	409,680
10	3,101	252,010	24,324	6354,764	24,850	4156,275	25,315	803,657
14	3,037	238,705	24,215	2913,663	24,781	1844,311	25,300	358,196
18	3,045	754,383	24,312	3841,824	24,807	2520,743	25,274	482,397
22	3,048	1024,656	24,263	3263,379	24,773	2091,131	25,268	397,229
26	3,032	1222,966	24,169	2530,239	24,650	1620,417	25,175	342,101
30	3,024	1786,882	24,057	2519,178	24,601	1537,660	25,100	295,482
34	3,004	3053,803	24,274	3076,935	24,758	1793,448	25,474	387,538
40	3,004	3801,363	24,288	3278,438	24,811	1887,532	25,198	317,772
46	2,964	3676,647	24,282	2494,038	24,786	1279,703	25,314	230,336
52	2,813	1632,036	24,198	1379,456	24,718	668,525	25,232	115,204
58	2,734	1359,076	24,150	1310,783	24,731	613,237	25,032	117,200

Missing data at predefined collision voltage intervals were supplemented by interpolating from corresponding data points within the same experimental dataset and are indicated in red. n.a.: data was not produced for both replicates.

M7+P17 Experiment 2

ΔCV [V]	Epitope peptide $\bar{z}_{(1)}$	Epitope peptide $h_{(1)}$	Antibody $\bar{z}_{(2)}$	Antibody $h_{(2)}$	Antibody complex+1 peptide $\bar{z}_{(3)}$	Antibody complex+1 peptide $h_{(3)}$	Antibody complex+2 peptides $\bar{z}_{(4)}$	Antibody complex+2 peptides $h_{(4)}$
0	3,110	112,007	23,985	4530,479	24,519	2868,356	25,026	550,415
2	3,188	216,140	24,171	10058,191	24,704	6460,979	25,218	1301,771
6	3,147	564,032	24,227	12223,838	24,751	7755,999	25,311	1478,128
10	3,079	2529,709	24,222	20454,129	24,759	12940,300	25,271	2532,435
14	3,096	1844,542	24,172	9190,779	24,958	65418,994	25,247	1122,886
18	3,071	3235,644	24,085	7886,588	24,604	4765,748	25,143	932,387
22	3,040	5697,907	24,201	8036,559	24,708	4717,572	25,207	864,845
26	3,028	8103,166	24,201	7619,806	24,672	4311,940	25,230	756,495
30	3,010	21857,063	24,257	16317,975	24,754	8922,498	25,274	1491,043
34	2,973	7150,304	24,278	4440,440	24,782	2364,328	25,270	364,513
40	2,925	17593,056	24,224	9329,733	24,665	3968,732	25,232	718,462
46	2,868	10245,170	24,191	5043,212	24,698	2407,868	25,135	324,046
52	2,827	11472,915	24,188	5650,107	24,671	2628,858	25,139	335,996
58	2,807	12041,899	24,169	6013,327	24,644	2659,376	25,161	330,985

Missing data at predefined collision voltage intervals were supplemented by interpolating from corresponding data points within the same experimental dataset and are indicated in red. n.a.: data was not produced for both replicates.

Table S4: Apex heights (h) and mean charge ($\bar{z}$) states of precursors and product ion signals upon gas phase dissociation of M11.7 antibody immune complex and assigned peptide.

M11.7+P21 Experiment 1

ΔCV	Epitope peptide		Antibody		Antibody complex+1 peptide		Antibody complex+2 peptides	
[V]	$\bar{z}_{(1)}$	$h_{(1)}$	$\bar{z}_{(2)}$	$h_{(2)}$	$\bar{z}_{(3)}$	$h_{(3)}$	$\bar{z}_{(4)}$	$h_{(4)}$
0	2,371	1752,039	23,662	3252,899	24,112	2894,096	24,523	905,692
2	2,380	1906,152	23,714	9093,265	24,156	8400,737	24,530	2832,001
6	2,205	35772,153	23,732	14826,351	24,167	14171,953	24,558	4532,257
10	2,432	29512,976	23,736	12535,107	24,149	11674,053	24,561	3798,055
14	2,651	44750,397	23,753	12728,197	24,165	11551,224	24,552	3691,081
18	3,254	93091,541	23,729	17230,019	24,109	15637,129	24,507	4767,255
22	3,242	105223,205	23,721	12238,673	24,121	10457,974	24,509	3263,555
26	3,267	173197,770	23,734	15013,205	24,107	12059,078	24,496	3562,696
30	3,452	376937,378	23,696	19706,342	24,070	15563,827	24,472	4417,054
34	3,290	270022,214	23,681	15775,449	24,071	11954,506	24,442	3248,449
40	3,306	486368,175	23,665	26068,546	24,054	18439,296	24,376	4769,158
46	3,332	446663,339	23,677	20663,437	24,020	13818,235	24,354	3439,399
52	3,354	414518,743	23,629	19019,328	23,999	128374,116	24,270	3003,718
58	n.d.	n.d.	23,496	23763,180	23,826	14870,536	24,149	3477,457

Missing data at predefined collision voltage intervals were supplemented by interpolating from corresponding data points within the same experimental dataset and are indicated in red. n.a.: data was not produced for both replicates.

M11.7+P21 Experiment 2

ΔCV	Epitope peptide		Antibody		Antibody complex+1 peptide		Antibody complex+2 peptides	
[V]	$\bar{z}_{(1)}$	$h_{(1)}$	$\bar{z}_{(2)}$	$h_{(2)}$	$\bar{z}_{(3)}$	$h_{(3)}$	$\bar{z}_{(4)}$	$h_{(4)}$
0	2,826	3479,532	23,510	5697,547	23,955	4927,754	24,365	1582,447
2	2,831	3286,057	23,796	10513,872	24,227	9676,026	24,637	3142,154
6	2,452	18212,064	23,841	16160,010	24,288	14835,326	24,676	4793,267
10	2,757	30772,226	23,828	16666,717	24,255	15158,767	24,550	4860,879
14	3,247	61511,819	23,803	17472,019	24,225	15729,252	24,615	5055,635
18	3,242	116297,780	23,763	21970,913	24,204	19237,315	24,572	5996,897
22	3,259	137954,519	23,770	16418,002	24,168	13757,848	24,526	4097,162
26	3,265	184968,221	23,747	16463,152	24,130	13258,034	24,507	3842,628
30	3,271	305144,945	23,709	21099,142	24,092	16341,227	24,470	4540,018
34	3,286	400155,008	23,677	23488,088	24,041	17630,578	24,422	4807,282
40	3,297	372808,572	23,612	19214,323	23,969	13824,374	24,315	3554,315
46	3,317	421019,722	23,562	19595,285	23,920	13204,115	24,250	3326,254
52	3,345	587130,781	23,547	27290,090	23,891	17810,349	24,220	4296,920
58	n.d.	n.d.	23,496	23763,180	23,826	14870,536	24,149	3477,457

Missing data at predefined collision voltage intervals were supplemented by interpolating from corresponding data points within the same experimental dataset and are indicated in red. n.a.: data was not produced for both replicates.

M11.7+P22 Experiment 1

ΔCV	Epitope peptide		Antibody		Antibody complex+1 peptide		Antibody complex+2 peptides	
[V]	$\bar{z}_{(1)}$	$h_{(1)}$	$\bar{z}_{(2)}$	$h_{(2)}$	$\bar{z}_{(3)}$	$h_{(3)}$	$\bar{z}_{(4)}$	$h_{(4)}$
0	3,000	957,910	23,765	7898,498	24,147	1167,170	n.d.	n.d.
2	3,000	817,070	23,701	14836,621	24,038	2634,401	n.d.	n.d.
6	3,375	3627,412	23,792	39589,131	24,173	6616,502	n.d.	n.d.
10	3,331	9813,212	23,782	65716,887	24,158	10812,761	n.d.	n.d.
14	3,315	15350,429	23,800	48545,799	24,172	7865,596	n.d.	n.d.
18	3,294	28067,154	23,817	58876,778	24,171	9213,398	n.d.	n.d.
22	3,304	61190,855	23,838	84066,798	24,187	12917,240	n.d.	n.d.
26	3,315	61305,015	23,855	70591,926	24,212	10442,026	n.d.	n.d.
30	3,282	56020,119	23,860	51089,696	24,203	7480,255	n.d.	n.d.
34	3,302	56805,701	23,811	41337,801	24,151	5828,886	n.d.	n.d.
40	3,336	88827,569	23,801	45914,231	24,063	4987,402	n.d.	n.d.
46	3,304	262815,981	23,808	50534,564	24,145	6675,095	n.d.	n.d.
52	3,366	107111,578	23,804	107680,263	24,070	11822,596	n.d.	n.d.
58	3,368	164210,165	23,817	44488,540	24,059	4923,565	n.d.	n.d.

Missing data at predefined collision voltage intervals were supplemented by interpolating from corresponding data points within the same experimental dataset and are indicated in red. n.a.: data was not produced for both replicates.

Table S4: continued

M11.7+P22 Experiment 2

ΔCV	Epitope peptide		Antibody		Antibody complex+1 peptide		Antibody complex+2 peptides	
[V]	$\bar{z}_{(1)}$	$h_{(1)}$	$\bar{z}_{(2)}$	$h_{(2)}$	$\bar{z}_{(3)}$	$h_{(3)}$	$\bar{z}_{(4)}$	$h_{(4)}$
0	3,000	699,381	23,626	8531,074	24,040	1381,522	n.d.	n.d.
2	3,213	1428,796	23,838	14848,556	24,211	2431,311	n.d.	n.d.
6	3,392	5408,819	23,950	49107,267	24,367	7926,257	n.d.	n.d.
10	3,313	6452,194	23,998	32171,465	24,366	5349,865	n.d.	n.d.
14	3,284	12011,006	23,994	33767,789	24,357	5583,400	n.d.	n.d.
18	3,322	25882,699	23,996	47755,901	24,348	7609,732	n.d.	n.d.
22	3,312	40881,562	24,006	56401,692	24,365	8780,390	n.d.	n.d.
26	3,294	57111,686	24,010	59531,310	24,365	8830,311	n.d.	n.d.
30	3,297	62461,692	24,013	65664,839	24,367	9762,711	n.d.	n.d.
34	3,296	60464,388	23,971	41620,172	24,558	7407,231	n.d.	n.d.
40	3,331	101560,272	23,936	56003,420	24,233	7582,505	n.d.	n.d.
46	3,333	113645,421	23,878	51647,929	24,166	6395,322	n.d.	n.d.
52	3,374	112879,652	23,901	45391,503	24,166	5191,458	n.d.	n.d.
58	3,421	125839,869	23,885	55189,367	24,142	5903,953	n.d.	n.d.

Missing data at predefined collision voltage intervals were supplemented by interpolating from corresponding data points within the same experimental dataset and are indicated in red. n.a.: data was not produced for both replicates.

M11.7+P23 Experiment 1

ΔCV	Epitope peptide		Antibody		Antibody complex+1 peptide		Antibody complex+2 peptides	
[V]	$\bar{z}_{(1)}$	$h_{(1)}$	$\bar{z}_{(2)}$	$h_{(2)}$	$\bar{z}_{(3)}$	$h_{(3)}$	$\bar{z}_{(4)}$	$h_{(4)}$
0	n.d.	n.d.	23,252	3575,884	23,671	1436,039	23,695	196,188
2	3,307	9200,843	23,304	47038,742	23,684	20590,298	24,019	3872,211
6	3,288	17063,605	23,391	171307,906	23,779	74670,369	24,133	13511,322
10	3,326	16808,047	23,333	158783,926	23,747	68106,763	24,099	11732,475
14	3,253	21083,780	23,404	77393,026	23,808	33152,222	24,145	5699,580
18	3,293	37758,502	23,276	62372,189	23,786	25686,210	24,146	4117,959
22	3,414	17752,116	23,402	91966,622	23,794	37434,968	24,133	5955,640
26	3,287	18939,100	23,483	26761,437	23,947	11168,516	24,037	1569,588
30	3,283	24022,127	23,429	24403,445	23,849	9408,274	24,172	1328,595
34	3,299	33339,133	23,440	25504,570	23,824	9690,319	24,198	1417,989
40	3,363	31468,804	23,346	18533,064	23,675	5661,539	23,879	737,216
46	3,404	35348,704	23,324	15862,636	23,614	4671,470	23,769	545,963
52	3,427	30616,180	23,289	18975,243	23,575	5369,489	23,695	672,799
58	3,512	3079,133	23,277	18722,534	23,565	5209,303	23,718	599,698

Missing data at predefined collision voltage intervals were supplemented by interpolating from corresponding data points within the same experimental dataset and are indicated in red. n.a.: data was not produced for both replicates.

M11.7+P23 Experiment 2

ΔCV	Epitope peptide		Antibody		Antibody complex+1 peptide		Antibody complex+2 peptides	
[V]	$\bar{z}_{(1)}$	$h_{(1)}$	$\bar{z}_{(2)}$	$h_{(2)}$	$\bar{z}_{(3)}$	$h_{(3)}$	$\bar{z}_{(4)}$	$h_{(4)}$
0	n.d.	n.d.	23,243	2858,401	23,561	1241,056	23,695	196,188
2	2,987	889,764	23,449	14761,401	23,614	6692,455	24,141	1256,960
6	2,445	4550,418	23,484	23653,046	23,636	10515,472	24,210	1578,941
10	2,473	4023,133	23,503	15299,471	23,654	6570,667	24,258	879,040
14	2,694	4805,423	23,508	10697,917	23,650	4424,955	24,227	586,795
18	3,330	5613,495	23,527	8525,249	23,635	3422,674	24,273	446,721
22	3,288	9076,535	23,530	10585,501	23,636	4317,776	24,138	537,203
26	3,304	17444,933	23,546	13110,702	23,631	4973,563	24,279	595,153
30	3,301	18085,285	23,524	11677,933	23,630	4032,323	24,171	521,863
34	3,350	26501,247	23,517	15702,858	23,622	5616,755	24,128	662,365
40	3,383	37347,773	23,479	19037,023	23,592	6275,055	24,060	761,402
46	3,453	25589,134	23,448	12770,541	23,567	3874,394	24,002	441,268
52	3,453	39263,088	23,413	21467,088	23,512	6230,879	23,956	711,012
58	3,548	28462,759	23,401	17678,044	23,517	4956,230	23,966	550,506

Missing data at predefined collision voltage intervals were supplemented by interpolating from corresponding data points within the same experimental dataset and are indicated in red. n.a.: data was not produced for both replicates.

Table S4: continued

M11.7+P24 Experiment 1

ΔCV [V]	Epitope peptide $\bar{z}_{(1)}$	Epitope peptide $h_{(1)}$	Antibody $\bar{z}_{(2)}$	Antibody $h_{(2)}$	Antibody complex+1 peptide $\bar{z}_{(3)}$	Antibody complex+1 peptide $h_{(3)}$	Antibody complex+2 peptides $\bar{z}_{(4)}$	Antibody complex+2 peptides $h_{(4)}$
0	n.d.	n.d.	23,299	7598,833	23,708	800,132	n.d.	n.d.
2	2,000	639,493	23,792	40722,866	24,148	4707,334	n.d.	n.d.
6	2,076	2092,820	23,708	45656,495	24,081	5411,917	n.d.	n.d.
10	2,102	4013,061	23,573	84303,770	23,917	11177,883	n.d.	n.d.
14	2,338	4750,837	23,466	82345,921	23,796	10595,152	n.d.	n.d.
18	2,762	3775,700	23,540	74875,779	23,897	7023,774	n.d.	n.d.
22	2,703	4757,286	23,484	77255,593	23,824	9313,771	n.d.	n.d.
26	3,200	6926,016	23,477	87503,720	23,855	9839,609	n.d.	n.d.
30	3,346	11424,117	23,449	117264,503	23,744	13474,102	n.d.	n.d.
34	3,520	19195,225	23,416	98043,547	23,713	10840,805	n.d.	n.d.
40	3,573	18695,150	23,377	89913,589	23,658	9735,589	n.d.	n.d.
46	3,616	21926,077	23,323	101955,731	23,619	10984,771	n.d.	n.d.
52	3,716	17493,331	23,341	88885,320	23,608	9085,173	n.d.	n.d.
58	3,676	9992,353	23,344	57919,657	23,618	5622,911	n.d.	n.d.

Missing data at predefined collision voltage intervals were supplemented by interpolating from corresponding data points within the same experimental dataset and are indicated in red. n.a.: data was not produced for both replicates.

M11.7+P24 Experiment 2

ΔCV [V]	Epitope peptide $\bar{z}_{(1)}$	Epitope peptide $h_{(1)}$	Antibody $\bar{z}_{(2)}$	Antibody $h_{(2)}$	Antibody complex+1 peptide $\bar{z}_{(3)}$	Antibody complex+1 peptide $h_{(3)}$	Antibody complex+2 peptides $\bar{z}_{(4)}$	Antibody complex+2 peptides $h_{(4)}$
0	n.d.	n.d.	23,386	5101,944	23,809	562,122	n.d.	n.d.
2	2,000	639,493	23,567	61220,966	23,965	6031,046	n.d.	n.d.
6	2,091	6938,336	23,631	95822,239	24,029	9271,310	n.d.	n.d.
10	2,100	12665,194	23,627	71512,205	23,994	7514,400	n.d.	n.d.
14	2,226	12142,039	23,616	71012,783	23,976	7485,370	n.d.	n.d.
18	2,303	12492,283	23,628	46224,446	23,986	5001,458	n.d.	n.d.
22	2,529	9734,595	23,584	31012,813	23,906	2841,803	n.d.	n.d.
26	2,787	8132,018	23,597	24564,413	23,937	2715,093	n.d.	n.d.
30	3,145	7709,547	23,605	20248,020	23,920	2128,980	n.d.	n.d.
34	3,284	13538,601	23,576	26375,374	23,900	2715,409	n.d.	n.d.
40	3,518	17080,298	23,556	21490,208	23,878	2194,663	n.d.	n.d.
46	3,628	16856,535	23,547	21099,251	23,886	2095,363	n.d.	n.d.
52	3,708	19846,602	23,540	26078,620	23,846	2493,024	n.d.	n.d.
58	3,791	15655,051	23,516	22544,940	23,824	2195,212	n.d.	n.d.

Missing data at predefined collision voltage intervals were supplemented by interpolating from corresponding data points within the same experimental dataset and are indicated in red. n.a.: data was not produced for both replicates.

M11.7+P25 Experiment 1

ΔCV [V]	Epitope peptide $\bar{z}_{(1)}$	Epitope peptide $h_{(1)}$	Antibody $\bar{z}_{(2)}$	Antibody $h_{(2)}$	Antibody complex+1 peptide $\bar{z}_{(3)}$	Antibody complex+1 peptide $h_{(3)}$	Antibody complex+2 peptides $\bar{z}_{(4)}$	Antibody complex+2 peptides $h_{(4)}$
0	n.d.	n.d.	23,270	6458,805	23,617	1191,676	23,598	230,839
2	2,092	1497,406	23,402	11013,669	23,705	2308,042	23,940	365,274
6	2,028	7196,223	23,470	13332,328	23,800	2749,747	24,030	406,649
10	2,087	4515,339	23,479	18765,430	23,999	3605,520	23,960	596,458
14	2,106	22164,946	23,349	20200,429	23,651	3776,431	23,839	578,695
18	2,215	15143,159	23,392	12933,388	23,689	2316,799	23,892	357,215
22	2,288	18592,894	23,410	14276,414	23,657	2596,574	23,889	381,033
26	2,400	17365,872	23,352	11289,080	23,653	2001,726	23,798	306,630
30	2,497	29239,160	23,372	20194,711	23,605	3369,844	23,735	521,104
34	2,529	31907,388	23,362	20341,645	23,667	3263,918	23,815	514,477
40	2,629	40224,280	23,299	25424,405	23,508	3723,912	23,815	609,994
46	2,670	51056,077	23,407	34908,480	23,648	5102,760	23,767	833,605
52	2,614	53421,052	23,462	44873,018	23,688	6176,554	23,783	1039,906
58	n.d.	n.d.	n.d.	n.d.	n.d.	n.d.	n.d.	n.d.

Missing data at predefined collision voltage intervals were supplemented by interpolating from corresponding data points within the same experimental dataset and are indicated in red. n.a.: data was not produced for both replicates.

Table S4: continued

M11.7+P25 Experiment 2

ΔCV	Epitope peptide		Antibody		Antibody complex+1 peptide		Antibody complex+2 peptides	
[V]	$\bar{z}_{(1)}$	$h_{(1)}$	$\bar{z}_{(2)}$	$h_{(2)}$	$\bar{z}_{(3)}$	$h_{(3)}$	$\bar{z}_{(4)}$	$h_{(4)}$
0	n.d.	n.d.	23,032	2152,621	23,427	436,350	23,458	95,592
2	2,034	2998,209	23,433	22043,619	23,730	3983,999	23,916	663,915
6	2,054	19909,654	23,457	29276,100	23,764	5312,869	23,900	895,158
10	2,575	6668,003	23,438	39874,797	23,754	7384,765	23,901	1244,185
14	2,136	27938,171	23,451	34064,705	23,754	6137,778	23,908	1072,776
18	2,276	20862,582	23,460	21512,594	23,763	3742,380	23,907	607,258
22	2,380	23101,340	23,424	19586,811	23,685	3493,415	23,846	572,688
26	2,410	22449,656	23,396	20940,199	23,669	3616,409	23,862	606,923
30	2,486	34132,775	23,436	25213,229	23,699	4097,467	23,811	673,375
34	2,566	25245,465	23,370	15727,678	23,635	2596,137	23,773	416,275
40	2,612	21773,098	23,352	14720,741	23,609	2283,233	23,815	391,164
46	2,613	23431,998	23,299	18384,031	23,539	2783,526	23,686	476,537
52	2,781	31641,446	23,333	20473,652	23,550	2983,507	23,703	525,503
58	n.d.	n.d.	n.d.	n.d.	n.d.	n.d.	n.d.	n.d.

Missing data at predefined collision voltage intervals were supplemented by interpolating from corresponding data points within the same experimental dataset and are indicated in red. n.a.: data was not produced for both replicates.

M11.7+P26 Experiment 1

ΔCV	Epitope peptide		Antibody		Antibody complex+1 peptide		Antibody complex+2 peptides	
[V]	$\bar{z}_{(1)}$	$h_{(1)}$	$\bar{z}_{(2)}$	$h_{(2)}$	$\bar{z}_{(3)}$	$h_{(3)}$	$\bar{z}_{(4)}$	$h_{(4)}$
0	n.d.	n.d.	22,953	3139,540	23,295	844,795	n.d.	n.d.
2	2,055	385,632	23,102	11730,810	23,412	3164,809	n.d.	n.d.
6	2,008	2107,494	23,156	13362,848	23,472	3611,509	n.d.	n.d.
10	2,061	4747,297	23,172	25483,319	23,502	6849,920	n.d.	n.d.
14	2,166	3554,777	23,221	19104,389	23,517	4904,949	n.d.	n.d.
18	2,308	4633,555	23,220	22048,409	23,515	5636,516	n.d.	n.d.
22	2,391	4700,948	23,216	19650,625	23,491	4831,629	n.d.	n.d.
26	2,457	6301,136	23,198	21890,195	23,489	5286,120	n.d.	n.d.
30	2,512	7128,444	23,188	22294,338	23,479	5199,468	n.d.	n.d.
34	2,551	9035,984	23,180	26029,473	23,440	5806,380	n.d.	n.d.
40	2,592	11583,759	23,185	30812,928	23,445	6670,732	n.d.	n.d.
46	2,622	6897,057	23,137	20214,480	23,392	4226,877	n.d.	n.d.
52	2,652	6743,569	23,153	22115,104	23,368	4460,164	n.d.	n.d.
58	2,645	7869,204	n.d.	n.d.	n.d.	n.d.	n.d.	n.d.

Missing data at predefined collision voltage intervals were supplemented by interpolating from corresponding data points within the same experimental dataset and are indicated in red. n.a.: data was not produced for both replicates.

M11.7+P26 Experiment 2

ΔCV	Epitope peptide		Antibody		Antibody complex+1 peptide		Antibody complex+2 peptides	
[V]	$\bar{z}_{(1)}$	$h_{(1)}$	$\bar{z}_{(2)}$	$h_{(2)}$	$\bar{z}_{(3)}$	$h_{(3)}$	$\bar{z}_{(4)}$	$h_{(4)}$
0	n.d.	n.d.	22,953	3139,540	23,295	844,795	n.d.	n.d.
2	2,083	128,383	22,961	28652,283	23,691	3075,834	n.d.	n.d.
6	2,136	1143,883	23,451	44655,956	23,769	11541,350	n.d.	n.d.
10	2,198	3432,234	23,417	68062,315	23,735	17349,418	n.d.	n.d.
14	2,316	3530,580	23,418	55641,488	23,740	13764,536	n.d.	n.d.
18	2,439	5016,307	23,443	61801,668	23,750	15129,277	n.d.	n.d.
22	2,585	10085,473	23,526	83492,946	23,789	22252,511	n.d.	n.d.
26	2,565	4749,582	23,608	31497,674	23,897	7508,379	n.d.	n.d.
30	2,608	9070,269	22,942	36926,291	23,876	10959,886	n.d.	n.d.
34	2,635	11952,799	23,640	51423,223	23,916	11056,773	n.d.	n.d.
40	2,661	11352,473	23,594	40815,320	23,864	8574,258	n.d.	n.d.
46	2,695	13031,327	23,584	44625,350	23,936	8781,490	n.d.	n.d.
52	2,695	13084,026	23,708	41606,628	23,939	8124,867	n.d.	n.d.
58	2,698	13775,504	n.d.	n.d.	n.d.	n.d.	n.d.	n.d.

Missing data at predefined collision voltage intervals were supplemented by interpolating from corresponding data points within the same experimental dataset and are indicated in red. n.a.: data was not produced for both replicates.

Table S4: continued

M11.7+P27 Experiment 1

ΔCV [V]	Epitope peptide $\bar{z}_{(1)}$	Epitope peptide $h_{(1)}$	Antibody $\bar{z}_{(2)}$	Antibody $h_{(2)}$	Antibody complex+1 peptide $\bar{z}_{(3)}$	Antibody complex+1 peptide $h_{(3)}$	Antibody complex+2 peptides $\bar{z}_{(4)}$	Antibody complex+2 peptides $h_{(4)}$
0	2,000	287,434	23,128	5617,374	23,445	1222,400	23,249	173,589
2	2,228	5282,509	23,695	33466,100	23,974	7727,604	23,123	1020,220
6	2,122	13613,002	23,678	46004,510	23,955	10485,093	23,120	1461,274
10	2,260	12357,165	23,529	24856,984	23,811	5325,572	23,042	646,863
14	2,398	12779,622	23,453	22433,965	23,739	4863,050	22,997	602,995
18	2,452	20896,476	23,440	25569,098	23,726	5473,165	22,952	680,372
22	2,544	26858,465	23,360	26863,894	23,646	5461,411	22,912	681,711
26	2,635	35530,572	23,444	29053,109	23,715	5689,897	22,893	691,174
30	2,634	49262,336	23,428	33716,867	23,684	6464,150	22,919	794,124
34	2,662	50648,141	23,411	31282,365	23,651	5828,100	22,572	857,562
40	2,679	59241,107	23,345	33975,499	23,574	6321,239	22,547	890,416
46	2,703	64577,542	23,308	35437,137	23,569	6272,648	22,560	881,748
52	2,668	63436,153	23,316	36636,759	23,506	6413,263	22,548	918,725
58	n.d.	n.d.	n.d.	n.d.	n.d.	n.d.	n.d.	n.d.

Missing data at predefined collision voltage intervals were supplemented by interpolating from corresponding data points within the same experimental dataset and are indicated in red. n.a.: data was not produced for both replicates.

M11.7+P27 Experiment 2

ΔCV [V]	Epitope peptide $\bar{z}_{(1)}$	Epitope peptide $h_{(1)}$	Antibody $\bar{z}_{(2)}$	Antibody $h_{(2)}$	Antibody complex+1 peptide $\bar{z}_{(3)}$	Antibody complex+1 peptide $h_{(3)}$	Antibody complex+2 peptides $\bar{z}_{(4)}$	Antibody complex+2 peptides $h_{(4)}$
0	2,000	287,434	23,026	7027,196	23,344	1563,090	23,701	203,777
2	2,343	4844,640	23,382	37451,613	23,661	7778,117	23,564	1385,778
6	2,116	17663,243	23,399	50586,479	23,666	10888,502	23,567	1950,411
10	2,187	24069,771	23,419	40295,603	23,688	8317,395	23,575	1297,657
14	2,309	35704,454	23,365	49669,477	23,700	10327,337	23,566	1645,885
18	2,425	33525,398	23,352	35992,193	23,617	7364,336	23,571	1151,069
22	2,553	20237,873	23,399	31965,750	23,630	6378,525	23,558	961,184
26	2,598	50445,539	23,398	35996,799	23,659	6905,270	23,574	1064,656
30	2,613	61418,392	23,403	38418,890	23,640	7221,564	23,578	1057,916
34	2,639	69597,705	23,392	41301,771	23,628	7352,787	23,560	1128,479
40	2,706	60629,543	23,392	34413,920	23,622	6048,169	23,572	836,831
46	2,706	65518,386	23,388	34334,472	23,607	5826,179	23,569	789,718
52	2,672	53938,141	23,309	33843,086	23,519	5761,064	23,531	794,034
58	n.d.	n.d.	n.d.	n.d.	n.d.	n.d.	n.d.	n.d.

Missing data at predefined collision voltage intervals were supplemented by interpolating from corresponding data points within the same experimental dataset and are indicated in red. n.a.: data was not produced for both replicates.

M11.7+P28 Experiment 1

ΔCV [V]	Epitope peptide $\bar{z}_{(1)}$	Epitope peptide $h_{(1)}$	Antibody $\bar{z}_{(2)}$	Antibody $h_{(2)}$	Antibody complex+1 peptide $\bar{z}_{(3)}$	Antibody complex+1 peptide $h_{(3)}$	Antibody complex+2 peptides $\bar{z}_{(4)}$	Antibody complex+2 peptides $h_{(4)}$
0	2,005	278,192	23,415	14165,600	23,653	2045,113	n.d.	n.d.
2	2,167	1296,374	23,558	29450,851	23,805	4068,032	n.d.	n.d.
6	2,027	12294,507	23,572	78151,996	23,809	10637,091	n.d.	n.d.
10	2,077	17741,903	23,611	95598,523	23,830	12983,064	n.d.	n.d.
14	2,117	23958,837	23,629	91597,348	23,846	12674,633	n.d.	n.d.
18	2,352	30712,910	23,629	84949,203	23,822	11584,513	n.d.	n.d.
22	2,439	40730,914	23,614	90237,451	23,807	11899,143	n.d.	n.d.
26	2,387	38354,127	23,605	83795,333	23,804	11051,240	n.d.	n.d.
30	2,543	71502,676	23,572	104600,395	23,781	13417,824	n.d.	n.d.
34	2,556	60052,878	23,545	83555,521	23,742	10539,047	n.d.	n.d.
40	2,618	64599,317	23,499	87105,491	23,698	10694,972	n.d.	n.d.
46	2,498	29370,808	23,465	58253,506	23,654	6912,763	n.d.	n.d.
52	2,524	28847,392	23,417	65926,080	23,597	7695,718	n.d.	n.d.
58	n.d.	n.d.	n.d.	n.d.	n.d.	n.d.	n.d.	n.d.

Missing data at predefined collision voltage intervals were supplemented by interpolating from corresponding data points within the same experimental dataset and are indicated in red. n.a.: data was not produced for both replicates.

Table S4: continued

M11.7+P28 Experiment 2

ΔCV	Epitope peptide		Antibody		Antibody complex+1 peptide		Antibody complex+2 peptides	
[V]	$\bar{z}_{(1)}$	$h_{(1)}$	$\bar{z}_{(2)}$	$h_{(2)}$	$\bar{z}_{(3)}$	$h_{(3)}$	$\bar{z}_{(4)}$	$h_{(4)}$
0	1,930	89,146	23,400	4638,617	23,703	730,626	n.d.	n.d.
2	2,061	16430,544	23,441	28599,078	23,685	4080,530	n.d.	n.d.
6	2,059	15603,592	23,463	64609,707	23,708	9223,187	n.d.	n.d.
10	2,075	22639,171	23,473	79293,742	23,720	11339,905	n.d.	n.d.
14	2,131	42607,712	23,497	97559,444	23,740	15366,098	n.d.	n.d.
18	2,103	36330,824	23,496	41027,900	23,706	7109,810	n.d.	n.d.
22	2,176	37281,480	23,465	47848,786	23,675	8234,087	n.d.	n.d.
26	2,161	37632,741	23,470	32096,472	23,679	5037,431	n.d.	n.d.
30	2,297	33767,086	23,494	25087,597	23,682	3676,641	n.d.	n.d.
34	2,215	32106,780	23,519	18811,663	23,731	2504,295	n.d.	n.d.
40	2,442	20714,782	23,520	20346,324	23,732	2607,226	n.d.	n.d.
46	2,591	15303,960	23,530	19744,844	23,709	2476,017	n.d.	n.d.
52	3,037	10895,081	23,521	22697,895	23,718	2846,137	n.d.	n.d.
58	n.d.	n.d.	n.d.	n.d.	n.d.	n.d.	n.d.	n.d.

Missing data at predefined collision voltage intervals were supplemented by interpolating from corresponding data points within the same experimental dataset and are indicated in red. n.a.: data was not produced for both replicates.

M11.7+P29 Experiment 1

ΔCV	Epitope peptide		Antibody		Antibody complex+1 peptide		Antibody complex+2 peptides	
[V]	$\bar{z}_{(1)}$	$h_{(1)}$	$\bar{z}_{(2)}$	$h_{(2)}$	$\bar{z}_{(3)}$	$h_{(3)}$	$\bar{z}_{(4)}$	$h_{(4)}$
0	2,000	337,590	23,305	2095,132	23,601	394,758	23,622	387,572
2	2,019	4636,932	23,399	12918,225	23,643	2836,417	23,955	1063,754
6	2,028	28011,742	23,507	27837,502	23,740	7075,752	23,979	655,380
10	2,049	27702,148	23,492	19838,951	23,729	4644,004	23,609	811,409
14	2,082	30011,760	23,461	21394,156	23,704	4890,760	23,617	384,838
18	2,180	21812,141	23,447	14400,029	23,692	2896,896	23,720	176,393
22	2,229	15647,817	23,461	9600,485	23,702	1835,911	n.d.	n.d.
26	2,309	20203,655	23,500	11297,900	23,740	2139,972	n.d.	n.d.
30	2,457	21074,019	23,480	11580,087	23,705	2067,731	23,569	469,545
34	2,493	22510,126	23,480	11510,357	23,720	1907,132	23,569	394,904
40	2,615	30534,529	23,449	14677,081	23,677	2469,807	23,525	373,876
46	2,711	21102,060	23,446	11605,260	23,665	1840,361	n.d.	n.d.
52	2,684	17745,095	23,457	11566,848	23,666	1822,482	23,548	423,130
58	2,680	19586,167	23,514	11364,359	23,672	1796,958	23,622	387,572

Missing data at predefined collision voltage intervals were supplemented by interpolating from corresponding data points within the same experimental dataset and are indicated in red. n.a.: data was not produced for both replicates.

M11.7+P29 Experiment 2

ΔCV	Epitope peptide		Antibody		Antibody complex+1 peptide		Antibody complex+2 peptides	
[V]	$\bar{z}_{(1)}$	$h_{(1)}$	$\bar{z}_{(2)}$	$h_{(2)}$	$\bar{z}_{(3)}$	$h_{(3)}$	$\bar{z}_{(4)}$	$h_{(4)}$
0	2,000	337,590	23,087	6712,791	23,397	1242,869	0,000	0,000
2	2,037	3804,788	23,182	19523,759	23,429	3785,715	23,622	387,572
6	2,021	16917,192	23,254	22107,577	23,531	4009,658	23,955	1063,754
10	2,065	27986,620	23,272	27091,478	23,530	5031,980	23,582	692,474
14	2,269	28708,249	23,273	23128,318	23,528	4224,602	23,576	546,868
18	2,225	26070,008	23,280	22267,596	23,524	4103,029	23,601	518,742
22	2,358	34360,133	23,285	23712,165	23,491	4149,457	23,552	536,279
26	2,455	38513,385	23,286	23881,449	23,519	3985,406	n.d.	n.d.
30	2,446	39425,044	23,261	25148,207	23,473	4184,316	n.d.	n.d.
34	2,524	37886,407	23,292	23963,033	23,510	3877,137	23,569	469,545
40	2,619	38043,442	23,028	24378,046	23,479	3642,723	23,569	394,904
46	2,706	37033,270	23,293	22466,497	23,504	3534,222	23,525	373,876
52	2,733	36424,584	23,273	24982,101	23,499	3733,174	n.d.	n.d.
58	2,716	32737,264	23,267	24152,089	23,448	3551,338	23,548	423,130

Missing data at predefined collision voltage intervals were supplemented by interpolating from corresponding data points within the same experimental dataset and are indicated in red. n.a.: data was not produced for both replicates.

Table S4: continued

M11.7+P30 Experiment 1

ΔCV	Epitope peptide		Antibody		Antibody complex+1 peptide		Antibody complex+2 peptides	
[V]	$\bar{z}_{(1)}$	$h_{(1)}$	$\bar{z}_{(2)}$	$h_{(2)}$	$\bar{z}_{(3)}$	$h_{(3)}$	$\bar{z}_{(4)}$	$h_{(4)}$
0	n.d.	n.d.	23,383	3156,776	23,710	6854,016	n.d.	n.d.
2	2,000	2179,870	23,544	40171,976	23,703	3979,652	n.d.	n.d.
6	2,000	10209,699	23,518	50981,123	23,702	4516,835	n.d.	n.d.
10	2,000	15068,107	23,567	39695,753	23,775	3151,209	n.d.	n.d.
14	2,016	15638,915	23,520	41793,899	23,705	3209,368	n.d.	n.d.
18	2,028	11857,757	23,565	35699,029	23,720	2720,223	n.d.	n.d.
22	2,042	11224,107	23,474	32354,181	23,654	2518,840	n.d.	n.d.
26	2,101	9656,828	23,562	33004,843	23,725	2542,632	n.d.	n.d.
30	2,128	9931,176	23,477	31538,127	23,627	2261,663	n.d.	n.d.
34	2,166	8950,808	23,565	33065,997	23,735	2394,751	n.d.	n.d.
40	2,229	11088,144	23,510	45966,322	23,680	3388,081	n.d.	n.d.
46	2,355	7295,667	23,540	48747,271	23,700	3563,007	n.d.	n.d.
52	2,461	3542,751	23,559	38494,783	23,714	2756,709	n.d.	n.d.
58	2,498	3180,964	23,543	44185,470	23,667	3090,961	n.d.	n.d.

Missing data at predefined collision voltage intervals were supplemented by interpolating from corresponding data points within the same experimental dataset and are indicated in red. n.a.: data was not produced for both replicates.

M11.7+P30 Experiment 2

ΔCV	Epitope peptide		Antibody		Antibody complex+1 peptide		Antibody complex+2 peptides	
[V]	$\bar{z}_{(1)}$	$h_{(1)}$	$\bar{z}_{(2)}$	$h_{(2)}$	$\bar{z}_{(3)}$	$h_{(3)}$	$\bar{z}_{(4)}$	$h_{(4)}$
0	n.d.	n.d.	23,513	62807,224	23,710	6854,016	n.d.	n.d.
2	2,000	5080,254	23,537	109348,812	23,725	13362,524	n.d.	n.d.
6	2,011	30353,701	23,557	108675,730	23,754	14319,157	n.d.	n.d.
10	2,025	41959,766	23,566	61362,164	23,757	6806,097	n.d.	n.d.
14	2,033	32819,109	23,660	17886,653	23,854	1735,229	n.d.	n.d.
18	2,021	17025,121	23,552	9297,511	23,742	968,802	n.d.	n.d.
22	2,037	8571,965	23,516	12318,828	23,684	1260,252	n.d.	n.d.
26	2,060	10014,954	23,522	12948,030	23,697	1346,178	n.d.	n.d.
30	2,053	10960,500	23,484	19100,924	23,647	2200,394	n.d.	n.d.
34	2,144	13420,581	23,509	12856,673	23,697	1393,620	n.d.	n.d.
40	2,204	6891,261	23,544	12236,400	23,700	1321,379	n.d.	n.d.
46	2,235	4075,125	23,523	19874,129	23,642	2079,842	n.d.	n.d.
52	2,461	3752,859	23,598	11864,993	23,752	1274,976	n.d.	n.d.
58	2,498	3180,964	23,476	6229,076	23,630	737,522	n.d.	n.d.

Missing data at predefined collision voltage intervals were supplemented by interpolating from corresponding data points within the same experimental dataset and are indicated in red. n.a.: data was not produced for both replicates.

M11.7+P31 Experiment 1

ΔCV	Epitope peptide		Antibody		Antibody complex+1 peptide		Antibody complex+2 peptides	
[V]	$\bar{z}_{(1)}$	$h_{(1)}$	$\bar{z}_{(2)}$	$h_{(2)}$	$\bar{z}_{(3)}$	$h_{(3)}$	$\bar{z}_{(4)}$	$h_{(4)}$
0	n.d.	n.d.	23,166	8661,325	23,381	602,129	n.d.	n.d.
2	2,000	3030,597	23,436	32645,118	23,648	2472,362	n.d.	n.d.
6	2,000	25166,884	23,451	65428,012	23,605	4731,811	n.d.	n.d.
10	2,000	20477,270	23,424	43372,853	23,584	3161,245	n.d.	n.d.
14	2,018	16778,929	23,480	38217,587	23,637	2727,092	n.d.	n.d.
18	2,036	13307,045	23,445	32595,587	23,608	2269,261	n.d.	n.d.
22	2,064	13151,625	23,420	32245,530	23,576	2216,725	n.d.	n.d.
26	2,064	12937,903	23,451	32772,654	23,607	2261,213	n.d.	n.d.
30	2,158	11763,662	23,376	32266,277	23,537	2112,975	n.d.	n.d.
34	2,206	11899,721	23,441	34434,229	23,587	2358,694	n.d.	n.d.
40	2,250	10755,274	23,406	34104,158	23,570	2317,858	n.d.	n.d.
46	2,422	6979,811	23,354	37795,855	23,536	2486,937	n.d.	n.d.
52	2,619	5033,129	23,399	38311,851	23,555	2476,523	n.d.	n.d.
58	2,650	3024,223	23,365	36584,883	23,519	2303,840	n.d.	n.d.

Missing data at predefined collision voltage intervals were supplemented by interpolating from corresponding data points within the same experimental dataset and are indicated in red. n.a.: data was not produced for both replicates.

Table S4: continued

M11.7+P31 Experiment 2

ΔCV	Epitope peptide		Antibody		Antibody complex+1 peptide		Antibody complex+2 peptides	
[V]	$\bar{z}_{(1)}$	$h_{(1)}$	$\bar{z}_{(2)}$	$h_{(2)}$	$\bar{z}_{(3)}$	$h_{(3)}$	$\bar{z}_{(4)}$	$h_{(4)}$
0	n.d.	n.d.	23,139	4002,918	23,460	474,063	n.d.	n.d.
2	2,000	9108,190	23,231	46334,524	23,447	8387,610	n.d.	n.d.
6	2,000	24177,261	23,408	35728,863	23,610	4429,271	n.d.	n.d.
10	2,000	40135,643	23,312	38894,392	23,488	4810,735	n.d.	n.d.
14	2,012	47676,571	23,371	47263,437	23,540	5680,249	n.d.	n.d.
18	2,030	39498,039	23,342	44173,420	23,512	5141,357	n.d.	n.d.
22	2,051	33663,809	23,381	38927,580	23,550	4228,633	n.d.	n.d.
26	2,066	24257,973	23,329	30582,002	23,504	3099,947	n.d.	n.d.
30	2,095	26357,668	23,308	37034,454	23,529	3738,202	n.d.	n.d.
34	2,141	21935,655	23,379	29679,489	23,562	2965,180	n.d.	n.d.
40	2,221	23849,413	23,362	41285,489	23,520	4897,081	n.d.	n.d.
46	2,425	14395,357	23,351	39713,060	23,505	4717,364	n.d.	n.d.
52	2,594	7292,433	23,365	33495,712	23,519	3572,724	n.d.	n.d.
58	2,683	3745,158	23,400	26961,349	23,542	2723,381	n.d.	n.d.

Missing data at predefined collision voltage intervals were supplemented by interpolating from corresponding data points within the same experimental dataset and are indicated in red. n.a.: data was not produced for both replicates.

M11.7+P32 Experiment 1

ΔCV	Epitope peptide		Antibody		Antibody complex+1 peptide		Antibody complex+2 peptides	
[V]	$\bar{z}_{(1)}$	$h_{(1)}$	$\bar{z}_{(2)}$	$h_{(2)}$	$\bar{z}_{(3)}$	$h_{(3)}$	$\bar{z}_{(4)}$	$h_{(4)}$
0	2,000	141,454	23,138	6220,823	23,651	2354,608	24,096	346,733
2	2,207	5065,702	23,207	32809,708	23,728	14491,714	24,139	2598,932
6	2,162	14542,524	23,311	28037,048	23,837	12164,213	24,278	2042,082
10	2,249	13663,548	23,287	26918,515	23,822	11361,392	24,275	1827,197
14	2,766	21312,703	23,311	30901,067	23,814	12700,310	24,235	1956,515
18	2,990	32660,409	23,325	28973,758	23,822	11508,553	24,220	1711,566
22	3,110	43344,562	23,300	25437,700	23,768	9710,799	24,206	1400,540
26	3,378	51760,412	23,326	17329,288	23,791	6255,950	24,188	860,653
30	3,362	67570,319	23,276	17170,026	23,717	5931,825	24,204	856,462
34	3,409	90100,779	23,320	17537,691	23,752	5779,123	24,066	765,228
40	3,370	113811,194	23,273	18248,515	23,653	5442,361	23,985	738,304
46	3,413	129417,319	23,272	18308,621	23,649	5420,835	23,940	657,683
52	3,492	104895,116	23,249	15427,092	23,605	4447,002	23,974	547,957
58	3,464	127283,464	23,235	17939,616	23,600	5076,357	24,072	443,146

Missing data at predefined collision voltage intervals were supplemented by interpolating from corresponding data points within the same experimental dataset and are indicated in red. n.a.: data was not produced for both replicates.

M11.7+P32 Experiment 2

ΔCV	Epitope peptide		Antibody		Antibody complex+1 peptide		Antibody complex+2 peptides	
[V]	$\bar{z}_{(1)}$	$h_{(1)}$	$\bar{z}_{(2)}$	$h_{(2)}$	$\bar{z}_{(3)}$	$h_{(3)}$	$\bar{z}_{(4)}$	$h_{(4)}$
0	2,000	141,454	23,059	5499,185	23,651	2354,608	24,155	272,171
2	2,186	2886,816	23,279	17938,091	23,728	14491,714	24,262	1232,651
6	2,107	9664,909	23,346	14535,323	23,837	12164,213	24,336	975,237
10	2,202	12781,043	23,339	15421,704	23,822	11361,392	24,319	968,477
14	2,328	14995,941	23,311	16211,309	23,814	12700,310	24,239	962,130
18	2,863	8811,507	23,304	9778,197	23,822	11508,553	24,261	551,061
22	3,139	18280,292	23,296	12958,464	23,768	9710,799	24,170	680,677
26	3,403	29583,301	23,254	11502,267	23,791	6255,950	24,157	571,431
30	3,432	51890,446	23,276	14854,932	23,717	5931,825	24,089	689,459
34	3,451	72583,079	23,242	17201,182	23,752	5779,123	24,051	761,846
40	3,470	64934,398	23,224	11703,421	23,653	5442,361	23,981	478,999
46	3,455	59069,949	23,218	10497,506	23,649	5420,835	24,073	450,218
52	3,539	66513,022	23,186	11840,722	23,605	4447,002	24,144	425,619
58	3,560	64770,491	23,194	12087,765	23,600	5076,357	24,072	443,146

Missing data at predefined collision voltage intervals were supplemented by interpolating from corresponding data points within the same experimental dataset and are indicated in red. n.a.: data was not produced for both replicates.

Table S5: Solvent accessible surface areas of hcTnT amino acid residues and epitope peptides.

residue			epitope / total
name	no.	area [$Å^2$]	area [$Å^2$]
MET	1	211.01	
SER	2	77.98	
ASP	3	113.67	
ILE	4	128.79	
GLU	5	129.22	
GLU	6	120.64	
VAL	7	67	
VAL	8	77.44	
GLU	9	123.71	
GLU	10	117.32	
TYR	11	150.72	
GLU	12	107.25	
GLU	13	127.44	
GLU	14	118.76	
GLU	15	136.96	
GLN	16	131.76	
GLU	17	133.05	
GLU	18	139.52	
ALA	19	54.94	
ALA	20	54.74	
VAL	21	89.83	
GLU	22	134.88	
GLU	23	111.54	
GLU	24	97.82	
GLU	25	115.18	
ASP	26	98.56	
TRP	27	168.18	
ARG	28	136.5	
GLU	29	123.22	
ASP	30	85.42	
GLU	31	127.4	
ASP	32	105.19	
GLU	33	134.91	
GLN	34	141.38	
GLU	35	102.51	
GLU	36	135.7	
ALA	37	53.78	
ALA	38	58.68	
GLU	39	129.98	
GLU	40	138.19	
ASP	41	109.42	
ALA	42	59.87	
GLU	43	131.21	
ALA	44	51.94	
GLU	45	136.97	
ALA	46	54.88	
GLU	47	129.44	
THR	48	86.34	
GLU	49	138.03	
GLU	50	121.87	
THR	51	80.34	
ARG	52	162.51	
ALA	53	50.59	
GLU	54	120.8	
GLU	55	135.45	
ASP	56	91.15	
GLU	57	140.07	
GLU	58	140.11	
GLU	59	104.4	
GLU	60	127.7	
GLU	61	142.67	
ALA	62	63.48	
LYS	63	122.06	
GLU	64	158.24	
ALA	65	88.52	
GLU	66	159.07	
ASP	67	141.53	
GLY	68	53.86	
PRO	69	128.31	
MET	70	185.21	
GLU	71	178.79	
GLU	72	152.32	
SER	73	115.31	
LYS	74	176.27	
PRO	75	114.29	
LYS	76	195.44	
PRO	77	122.85	
ARG	78	222.15	
SER	79	98.6	
PHE	80	189.8	
MET	81	177.91	
PRO	82	101.98	
ASN	83	138.35	
LEU	84	173.91	
VAL	85	103.72	
PRO	86	98.26	
PRO	87	111.28	
LYS	88	170.68	
ILE	89	142.69	
PRO	90	109.34	
ASP	91	153.91	
GLY	92	71.5	
GLU	93	170.88	
ARG	94	200.77	
VAL	95	89.38	
ASP	96	90.96	
PHE	97	164	
ASP	98	120.91	
ASP	99	70.45	
ILE	100	93.01	
HIS	101	106	
ARG	102	144.87	
LYS	103	134.6	
ARG	104	162.05	
MET	105	110.55	
GLU	106	94.69	
LYS	107	126.89	
ASP	108	63.01	
LEU	109	94.99	
ASN	110	92.85	
GLU	111	95.58	
LEU	112	103.51	
GLN	113	88.88	
ALA	114	54.78	
LEU	115	111.22	
ILE	116	94.17	
GLU	117	119.28	
ALA	118	53.75	
HIS	119	104.29	
PHE	120	130.37	
GLU	121	116.69	
ASN	122	80.25	
ARG	123	123.74	
LYS	124	121.51	
LYS	125	121.54	
GLU	126	92.49	
GLU	127	103.81	
GLU	128	110.5	
GLU	129	127.08	
LEU	130	99.12	
VAL	131	81.89	
SER	132	70.82	
LEU	133	73.11	
LYS	134	138.76	
ASP	135	75.75	
ARG	136	121.61	
ILE	137	77.54	
GLU	138	125.76	
ARG	139	149.67	
ARG	140	134.96	
ARG	141	162.2	
ALA	142	59.37	
GLU	143	99.19	
ARG	144	126.1	**1595.85**
ALA	145	52.28	
GLU	146	110.27	
GLN	147	83.19	
GLN	148	88.9	
ARG	149	183.17	
ILE	150	67.74	
ARG	151	92.86	
ASN	152	66.98	
GLU	153	127.57	

Table S5: continued

residue			epitope / total
name	no.	area [Å^2]	area [Å^2]
ARG	154	156.28	
GLU	155	105.94	
LYS	156	135.35	
GLU	157	88.59	
ARG	158	174.97	
GLN	159	112.56	**1646.65**
ASN	160	102.83	
ARG	161	151.7	
LEU	162	109.56	
ALA	163	55.17	
GLU	164	129.49	
GLU	165	97.86	
ARG	166	171.14	
ALA	167	43.01	
ARG	168	154.62	
ARG	169	151.59	
GLU	170	105.9	
GLU	171	130.23	
GLU	172	78.75	
GLU	173	89.23	
ASN	174	79.45	
ARG	175	144.96	
ARG	176	163.34	
LYS	177	98.85	
ALA	178	58.81	
GLU	179	93.18	
ASP	180	53.56	
GLU	181	125.68	
ALA	182	46.07	
ARG	183	156.28	
LYS	184	134.83	
LYS	185	157.83	
LYS	186	152.97	
ALA	187	46.19	
LEU	188	116.11	
SER	189	90.79	
ASN	190	120.82	
MET	191	85.04	
MET	192	160.71	
HIS	193	158.52	
PHE	194	155.2	
GLY	195	78.4	
GLY	196	68.58	
TYR	197	219.81	
ILE	198	138.08	
GLN	199	160.45	
LYS	200	175.92	
GLN	201	161.18	
ALA	202	74.87	
GLN	203	148.11	
THR	204	110.23	
GLU	205	165.17	
ARG	206	218.75	
LYS	207	192.7	
SER	208	115.09	
GLY	209	68.9	
LYS	210	186.85	
ARG	211	220.1	
GLN	212	77.98	
THR	213	76.06	
GLU	214	151.81	
ARG	215	207.05	
GLU	216	92.66	
LYS	217	99.5	
LYS	218	138.58	
LYS	219	154.31	
LYS	220	102.21	
ILE	221	78.08	
LEU	222	80.7	
ALA	223	67.36	
GLU	224	120.98	
ARG	225	183.64	
ARG	226	184.12	
LYS	227	174.67	
VAL	228	136.25	
LEU	229	93.06	
ALA	230	61.2	
ILE	231	69.57	
ASP	232	155.65	
HIS	233	143.37	
LEU	234	45.5	
ASN	235	83.43	
GLU	236	121.39	
ASP	237	102.02	
GLN	238	81.46	
LEU	239	70.98	
ARG	240	115.26	
GLU	241	128.95	
LYS	242	57.74	
ALA	243	56.85	
LYS	244	141.36	
GLU	245	98.45	
LEU	246	71.22	
TRP	247	165.81	
GLN	248	109.87	
SER	249	56.12	
ILE	250	94.16	
TYR	251	132.52	
ASN	252	70.14	
LEU	253	104.11	
GLU	254	112.07	
ALA	255	47.1	
GLU	256	67.35	
LYS	257	122.08	
PHE	258	122.85	
ASP	259	79.52	
LEU	260	114.8	
GLN	261	89.89	
GLU	262	78.27	
LYS	263	116.78	
PHE	264	115.18	
LYS	265	126.65	
GLN	266	93.14	
GLN	267	83.59	
LYS	268	79.26	
TYR	269	143.02	
GLU	270	89.95	
ILE	271	86.42	
ASN	272	79.34	
VAL	273	67.23	
LEU	274	101.64	
ARG	275	158.34	
ASN	276	96.83	
ARG	277	160.22	
ILE	278	97.26	
ASN	279	70.37	
ASP	280	84.66	
ASN	281	97.23	
GLN	282	100.6	
LYS	283	114.99	
VAL	284	92.06	
SER	285	77.8	
LYS	286	159.76	
THR	287	103.81	
ARG	288	197.4	
GLY	289	69.71	
LYS	290	178.82	
ALA	291	80.49	
LYS	292	176.04	
VAL	293	134.71	
THR	294	119.35	
GLY	295	51.1	
ARG	296	222.08	
TRP	297	240.74	
LYS	298	254.66	**34657.73**
